# Dual Piperidine-Based Histamine H_3_ and Sigma-1 Receptor Ligands in the Treatment of Nociceptive and Neuropathic Pain

**DOI:** 10.1021/acs.jmedchem.3c00430

**Published:** 2023-07-07

**Authors:** Katarzyna Szczepańska, Tadeusz Karcz, Maria Dichiara, Szczepan Mogilski, Justyna Kalinowska-Tłuścik, Bogusław Pilarski, Arkadiusz Leniak, Wojciech Pietruś, Sabina Podlewska, Katarzyna Popiołek-Barczyk, Laura J. Humphrys, M. Carmen Ruiz-Cantero, David Reiner-Link, Luisa Leitzbach, Dorota Łażewska, Steffen Pockes, Michał Górka, Adam Zmysłowski, Thierry Calmels, Enrique J. Cobos, Agostino Marrazzo, Holger Stark, Andrzej J. Bojarski, Emanuele Amata, Katarzyna Kieć-Kononowicz

**Affiliations:** † Department of Technology and Biotechnology of Drugs, Faculty of Pharmacy, Jagiellonian University Medical College, Medyczna 9, 30-688 Kraków, Poland; ‡ Department of Medicinal Chemistry, Maj Institute of Pharmacology, Polish Academy of Sciences, Smętna 12, 31-343 Kraków, Poland; § Department of Drug and Health Sciences, University of Catania, V.le A. Doria, 95125 Catania, Italy; ∥ Department of Pharmacodynamics, Faculty of Pharmacy, Jagiellonian University Medical College, Medyczna 9, 30-688 Kraków, Poland; ⊥ Department of Crystal Chemistry and Crystal Physics, Faculty of Chemistry, Jagiellonian University, Gronostajowa 2, 30-387 Kraków, Poland; # Cerko Sp. z o.o. Sp.k, Al. Zwycięstwa 96/98, 81-451 Gdynia, Poland; ∇ Celon Pharma S.A., R&D Centre, Marymoncka 15, 05-152 Kazuń Nowy, Poland; ○ Department of Neurochemistry, Maj Institute of Pharmacology, Polish Academy of Sciences, Smętna 12, 31-343 Kraków, Poland; ◆ Institute of Pharmacy, Faculty of Chemistry and Pharmacy, University of Regensburg, Universitätsstraße 31, D-93053 Regensburg, Germany; ¶ Department of Pharmacology and Neurosciences Institute (Biomedical Research Center), University of Granada, and Biosanitary Research Institute ibs. Granada, Avenida de la Investigación 11, 18016 Granada, Spain; & Institute of Pharmaceutical and Medicinal Chemistry, Heinrich Heine University Düsseldorf, Universitaetsstr. 1, 40225 Düsseldorf, Germany; ● Bioprojet-Biotech, 4rue du Chesnay Beauregard, 35762 Saint-Gregoire Cedex, France

## Abstract

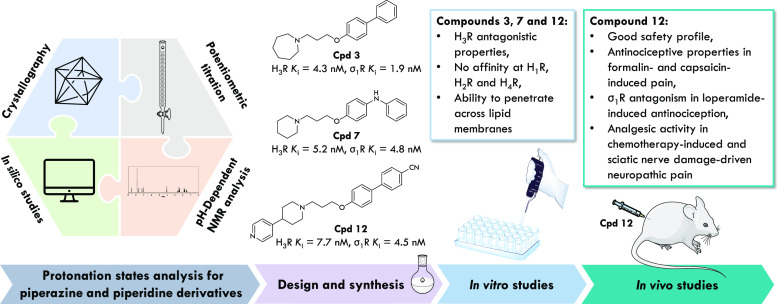

In search of new dual-acting histamine H_3_/sigma-1 receptor ligands, we designed a series of compounds structurally based on highly active *in vivo* ligands previously studied and described by our team. However, we kept in mind that within the previous series, a pair of closely related compounds, **KSK67** and **KSK68**, differing only in the piperazine/piperidine moiety in the structural core showed a significantly different affinity at sigma-1 receptors (σ_1_Rs). Therefore, we first focused on an in-depth analysis of the protonation states of piperazine and piperidine derivatives in the studied compounds. In a series of 16 new ligands, mainly based on the piperidine core, we selected three lead structures (**3**, **7**, and **12**) for further biological evaluation. Compound **12** showed a broad spectrum of analgesic activity in both nociceptive and neuropathic pain models based on the novel molecular mechanism.

## 1 Introduction

The treatment of complex, multifactorial diseases by single target-oriented therapies rarely results in good efficacy. For this reason, the approach based on the simultaneous modulation of multiple targets’ activity captured the interest of the pharmaceutical industry and academia. The increasing number of evidence indicates improvement in both the therapeutic safety and efficacy of multitarget-directed ligands (MTDLs) compared with single-target drugs.^1,2^

The histamine H_3_ receptor (H_3_R) is a G protein-coupled receptor (GPCR) highly expressed in the central nervous system (CNS), where it acts as an auto- and heteroreceptor to regulate neurotransmission.^3^ Thus, it has been considered a relevant target in the treatment of varied disorders, such as Alzheimer’s disease, schizophrenia, and attention deficit hyperactivity disorder.^4^ Furthermore, being localized in several CNS regions responsible for nociception, H_3_Rs are also associated with pain^5^ through the involvement in the central sensitization of pain.

**Figure 1:**
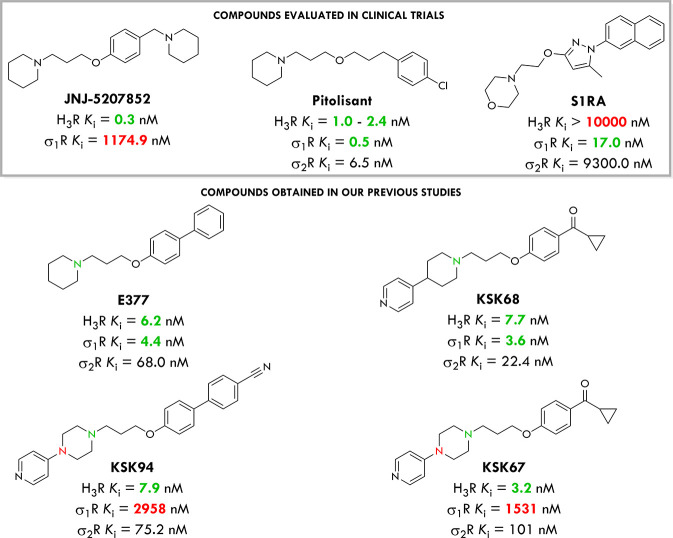
Structures of selective and dual-targeting compounds evaluated in clinical trials and obtained in our previous work.^23^

A range of competitive antagonists/inverse agonists have been discovered and progressed into clinical trials, among which pitolisant was approved in 2016 for the treatment of narcolepsy (Figure 1).^6,7^ As a consequence, the interest in the clinical application of novel H_3_R antagonists, especially those with multifunctional profiles, has clearly increased.^8−12^

Importantly, recent studies have shown that some clinically evaluated H_3_R antagonists possess additional affinity at sigma-1 receptors, which may play an important role in their pharmacology and underlie the differences in the reported preclinical and clinical efficacy.^13,14^ For example, JNJ-5207852 was highly selective for H_3_R compared to the sigma-1 receptor, and the approved agent pitolisant displayed similar affinity toward both receptors (Figure 1).^13^ Therefore, sigma-1 affinity should be always considered when interpreting the *in vivo* efficacy of novel H_3_R ligands.

Sigma (σ) receptors, initially described as a subtype of opioid receptors, are now considered a separate class. Pharmacological studies have distinguished two types of σ receptors, namely, σ_1_ and σ_2_.^15^ The sigma-1 receptor (σ_1_R) is a ligand-regulated chaperone protein that modulates the signaling of proteins (receptors, enzymes) with which it interacts.^16^ It is a unique and poorly understood biological target engaged in physiological mechanisms of learning and memory, depression, anxiety, and schizophrenia.^17^ Sigma-1 antagonists are reported to be effective agents in neuropathic pain,^18^ and although the mechanism of action is not well understood, several studies suggest the involvement of σ_1_Rs in the regulation of ion channel function (including NMDA/GluN receptors and K^+^/Ca^2+^ channels), which are involved in the pathogenesis of pain.^17^ Many ion channels are located at the nociceptor peripheral terminal, affecting neuron excitability after injury and as a result affecting pain sensation.^17^ In this context, the highly selective σ_1_ antagonist **S1RA** (Figure 1) is in phase II clinical trials for pain treatment, with an intended indication for enhancing opioid analgesia and amelioration of neuropathic pain.^18^

**Figure 2:**
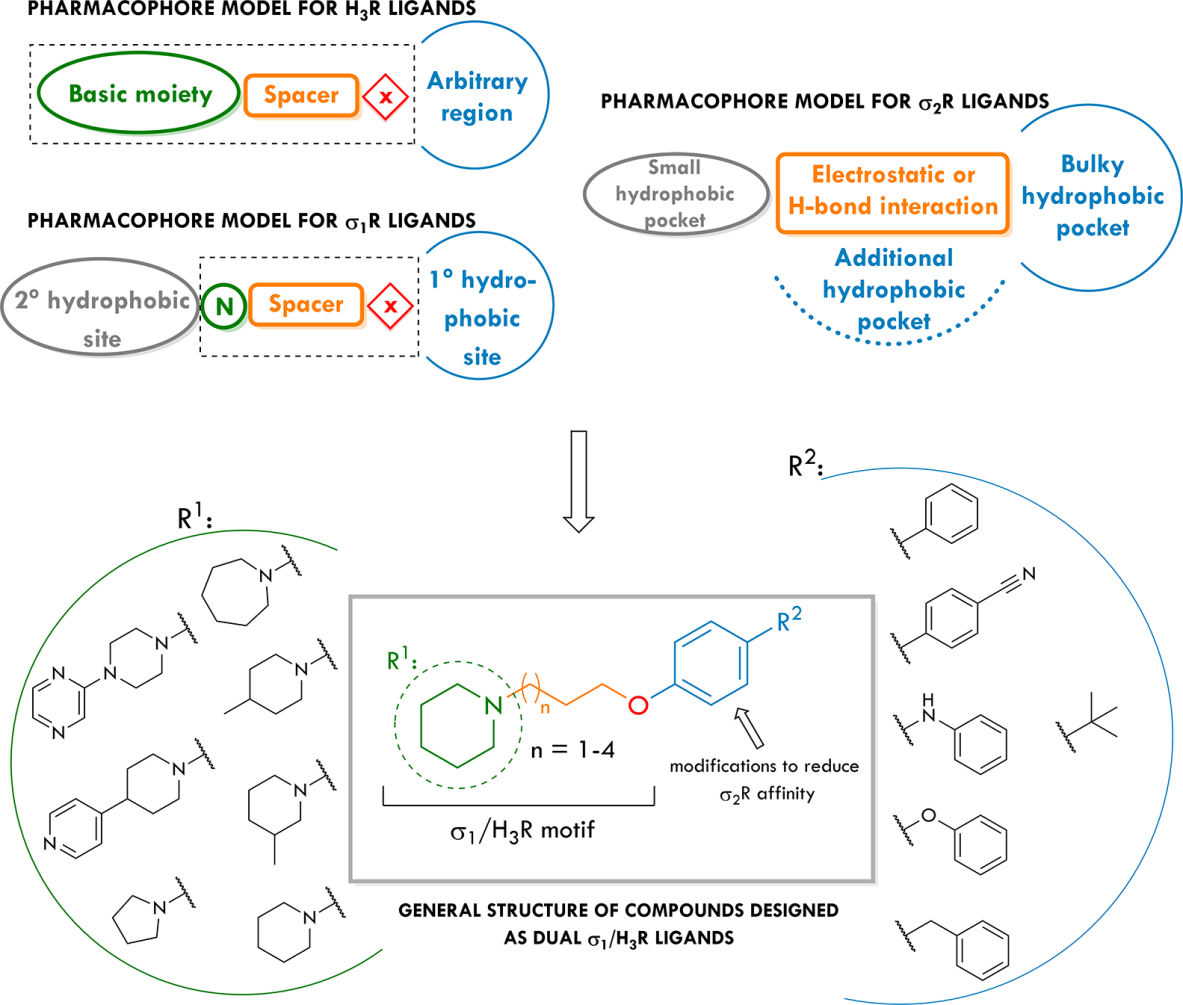
Pharmacophore models for H_3_R, σ_1_R, and σ_2_R ligands. General structures of compounds described in this work.

Considering the clear relation between H_3_R and σ_1_R, along with the fact that dual-targeting compounds can lead to several improvements when compared to selective drugs, great efforts should be made to develop such ligands for the treatment of various pain conditions. Answering this challenge, in our recent studies, we investigated 20 previously reported H_3_R ligands for their affinity toward σRs to check whether their high preclinical *in vivo* efficacy is related to a synergistic effect of the dual H_3_R and σ_1_R modulation.^19−22^ According to the obtained results, compounds **E377** and **KSK68** (Figure 1) turned out to be high-affinity histamine H_3_ and σ_1_ receptor antagonists with negligible affinity at the other histamine receptor subtypes and promising antinociceptive *in vivo* activity.^23^ Moreover, the piperidine moiety in the basic part of those compounds has been established as a critical structural feature for dual H_3_/σ_1_ receptor activity as can be seen by comparing the data for compounds **KSK67** and **KSK68** (Figure 1). The piperidine derivatives in protonated form are involved in the essential salt bridge interaction with Glu172 in the σ_1_R binding pocket, being responsible for the high biological activity of the studied ligands. As discussed in our previous work, this phenomenon can be attributed to a change in the protonation state or states at physiological pH.^23^ Therefore, in the initial step of designing the next series of ligands, we focused on an in-depth analysis of the protonation states of piperazine vs piperidine derivatives using crystallography, potentiometric titration, and NMR spectroscopy measurements in a pH-controlled environment. Next, we designed a series of 16 new compounds, mainly based on the piperidine core, with the general structure presented in Figure 2 and performed their pharmacological characterization using *in vitro* methods. Finally, lead compounds were tested in animal models of nociceptive and neuropathic pain.

## 2 Results and Discussion

### 2.1 Chemistry

**Scheme 1:**
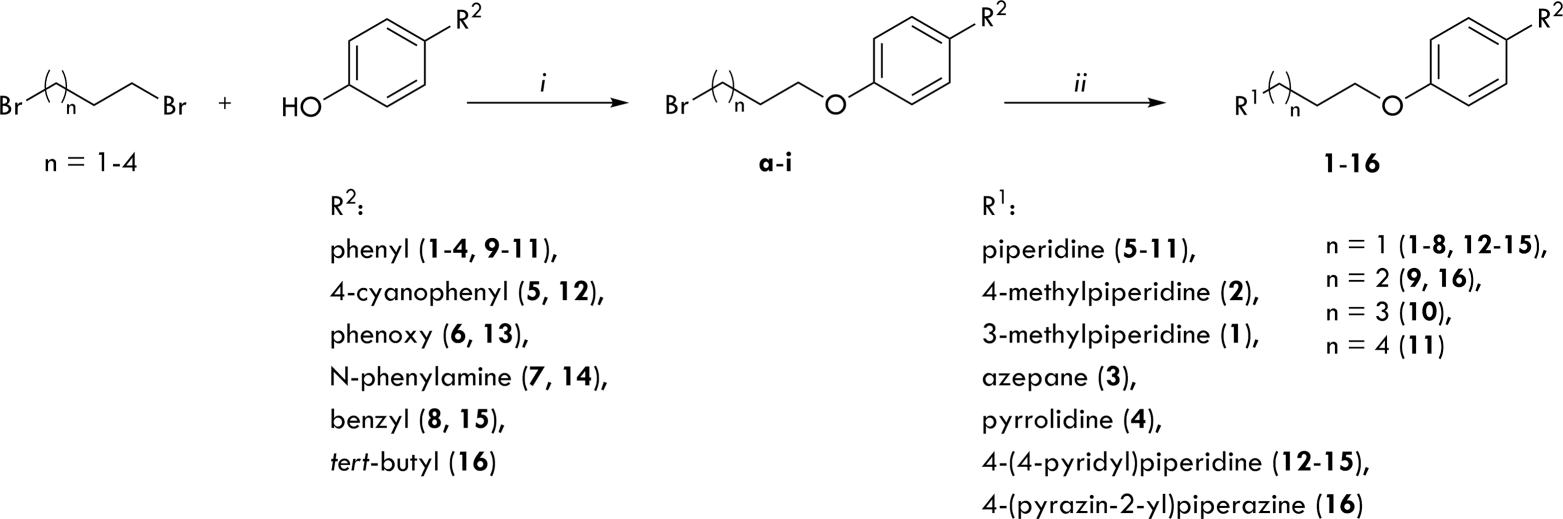
General Synthetic Pathway for Starting Ethers **a**–**i** and Compounds **1**–**16** Reagents and conditions: (i) proper α,ω-dibromoalkane, CH_3_CH_2_CH_2_ONa, 60 °C: 3 h, reflux: 3 h; (ii) proper cycloalkylamine, K_2_CO_3_, KI, EtOH/H_2_O (5:1), reflux: 8–12 h.

The synthesis of the desired final compounds **1**–**16** was achieved through the synthetic route presented in Scheme 1. According to the previously described procedure,^19,21,22,24,25^ the phenoxy alkyl bromides **a**–**i** were obtained mainly by one-step alkylation of commercially available phenols with 1,3-dibromopropane (**a**–**e**), 1,4-dibromobutane (**f**, **i**), 1,5-dibromopentane (**g**), or 1,6-dibromohexane (**h**) in propan-1-ol under reflux conditions. Obtained precursor bromides were then coupled with 3-methylpiperidine (**1**), 4-methylpiperidine (**2**), azepane (**3**), pyrrolidine (**4**), piperidine (**5**–**11**), 4-(piperidin-4-yl)pyridine (**12**–**15**), or 2-(piperazin-1-yl)pyrazine (**16**) in a mixture of ethanol/water with powdered potassium carbonate and a catalytic amount of potassium iodide. The final products were obtained as free bases and isolated as oxalate or hydrogen oxalate salts.

### 2.2 Protonation Investigation Based on Crystal Structure Analysis

**Figure 3:**
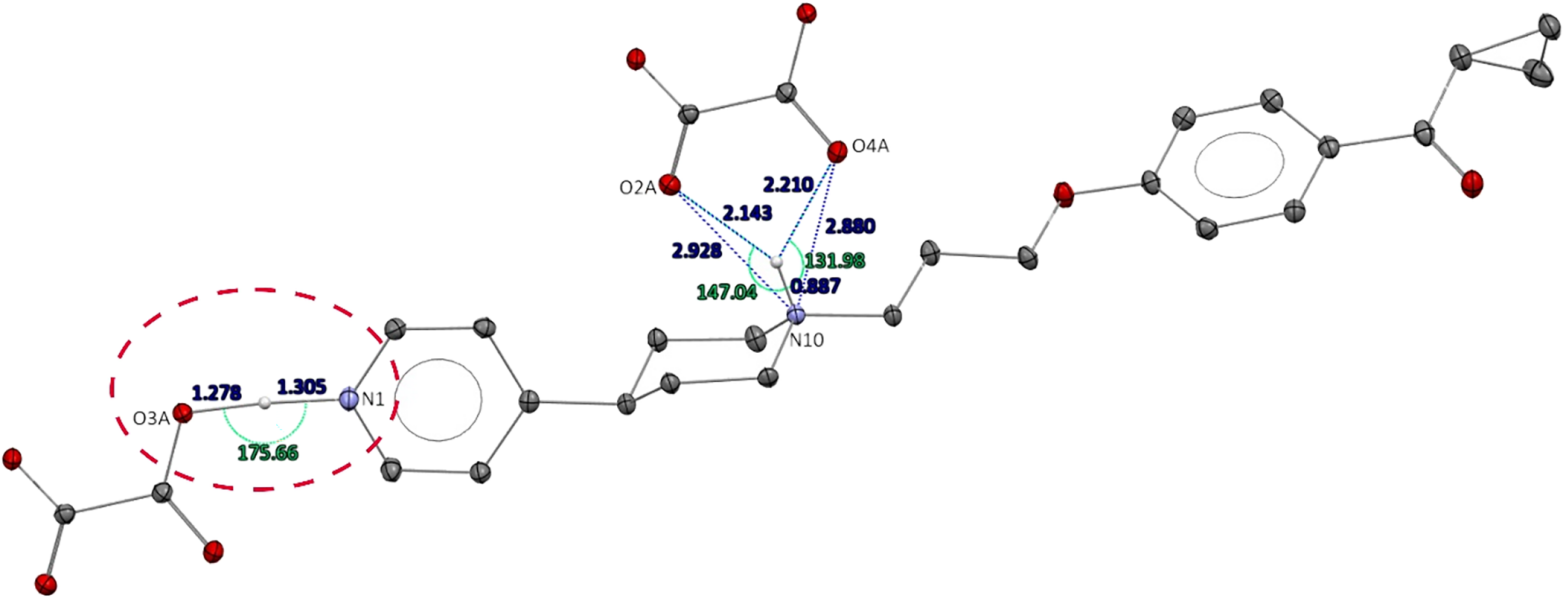
The geometrical parameters of the strongest interactions observed in the crystal structure of **KSK68** (charge-assisted hydrogen bonds N^+^-H···O^–^ type; distances in dark blue, angles in light green). The weaker basicity of the N1 atom in the pyridine ring is manifested by the hydrogen shift toward oxalate anion (discussed fragment is depicted with a dashed-line ellipsoid). Displacement ellipsoids of nonhydrogen atoms are drawn at the 30% probability level. H atoms not involved in the described interactions were removed for figure clarity.

To determine the potential differences in the protonation of piperidine and piperazine derivatives, we selected two pairs of compounds for crystallographic studies, differing only in the basic part. The first contains the cyclopropylmethanone derivatives **KSK67** and **KSK68** obtained in our previous work^22,23^ (Figure 1), whereas the second contains two nitrile derivatives: **KSK94** (also published earlier,^22,23^Figure 1) and compound **12** (structure in Pharmacology, Table 2). In the **KSK67** and **KSK68** oxalate crystals, the total charge of the main organic compound is +2, with protonation centers at N1 (pyridine) and N10 (piperazine/piperidine) nitrogen atoms. The resulting positive charge is compensated by oxalate anions that form charge-assisted hydrogen bonds with the mentioned protonated amine (Figures S1 and S2). An interesting geometry is observed for N1-H-O3A interaction in structure **KSK68** (distances: N1···O3A 2.581 Å, N1-H 1.305 Å, O3A-H 1.278 Å; angle: N1-H-O3A 175.66°), suggesting possible proton transfer between pyridine nitrogen N1 and O3A of the carboxylic group (Figure 3). That may imply weaker basic properties of N1 compared to the piperidine N10 protonation center for **KSK68**. This can be deduced also based on the obtained **KSK68_OH** crystal structure, where increased pH led to +1 cation, with sole protonation at N10 (Figure S3).

The crystal structures obtained for the free bases **KSK67_fb**, **KSK68_fb**, **KSK94_fb**, and **12_fb** from water/organic solvents revealed peculiar properties of molecules with 4-pyridylpiperazine moiety. Surprisingly, molecules containing the mentioned fragment (**KSK67_fb** and **KSK94_fb**) did not crystalize in the neutral form. The pyridine nitrogen of those compounds is a very strong base and easily protonates in the solution (Figures S4 and S6). Thus, in the crystal, an iodide salt is observed. The counter ion I^–^ was introduced during the synthesis, where the catalytic amount of potassium iodide was applied (see Chemistry Section 2.1.). An analogous basic property of N1 is not observed for **KSK68_fb** and **12_fb** with 4-pyridylpiperidine moiety. For these samples, crystals with the neutral form of the compound were obtained (Figures S5 and S7).

The observed strong basic character of N1 in structures **KSK67_fb** and **KSK94_fb** is a consequence of the increased availability of lone pair electrons correlated with the electron source at the para position (lone pair of N7 nitrogen atom of the piperazine ring). The N7-lone pair shift manifests in N7-C4 bond shortening to ∼1.35 Å (Table 1), which implies a partial double bond character and decreasing pyramidality of N7 atom. The last observation is confirmed by angles C4-N7-C8 and C4-N7-C12 being ∼120° (Table 1) and defining nearly flat geometry around N7. The observed change of N7 hybridization from sp^3^ to sp^2^ leads to the quasi-planar mutual orientation of pyridine and piperazine (torsion angles C3-C4-N7-C8 and C5-C4-N7-C12 closer to 0/180°; Table 1). Such effect and geometry are often observed for arylpiperazine fragments. However, it may strongly depend on substituents or heteroatoms in the aromatic fragment.^26^ Nevertheless, the observed phenomenon is responsible for the rigidity of the 4-pyridylpiperazine fragment that determines the highly basic character of N1 as well as the orientation of π-electrons of the aromatic fragment. This last characteristic may be a strong geometrical feature responsible for effective ligand–protein recognition and may explain the observed selectivity of the investigated compounds containing the 4-pyridylpiperazine fragment for H_3_R.

**Table 1:**
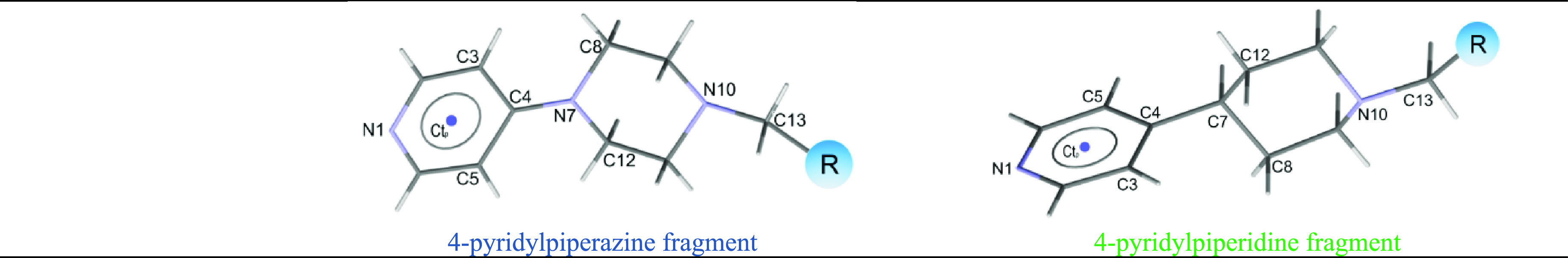

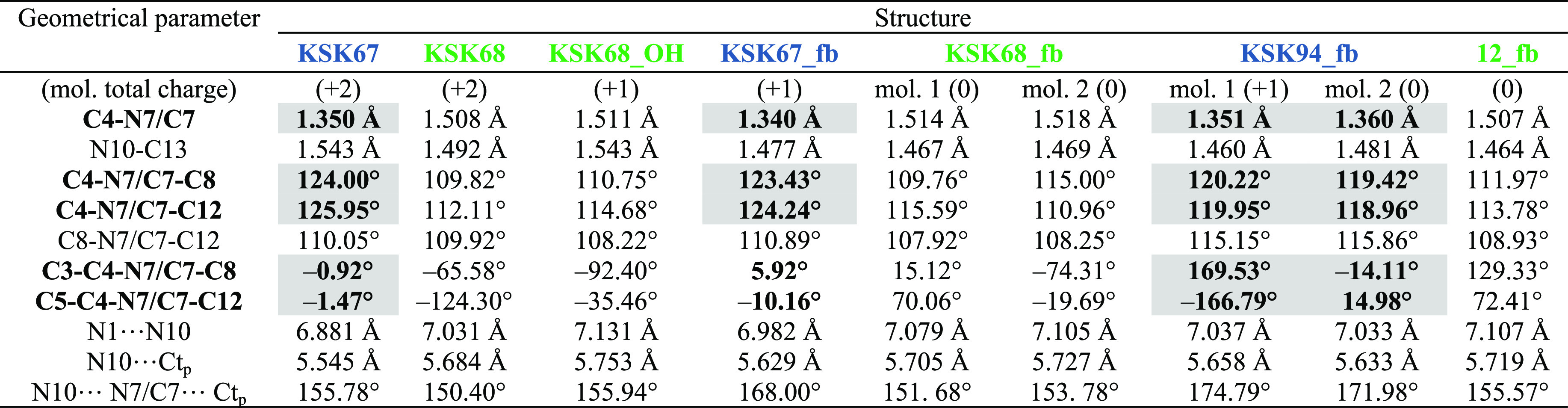
Selected Geometrical Parameters Defined for the Determined Crystal Structures^a^

a Ct_p_: geometrical center of the pyridine ring.

### 2.3 Determination of the Basicity of Selected Piperidine and Piperazine Derivatives by Potentiometric Titration

Several key physicochemical properties that influence absorption and distribution processes, such as lipophilicity and solubility, depend on p*K*_a_. The ionization state is a key parameter not only in ADME profiling but also during ligand–receptor mutual recognition and interaction, as it usually occurs in an aqueous environment at physiological pH.

**Figure 4:**
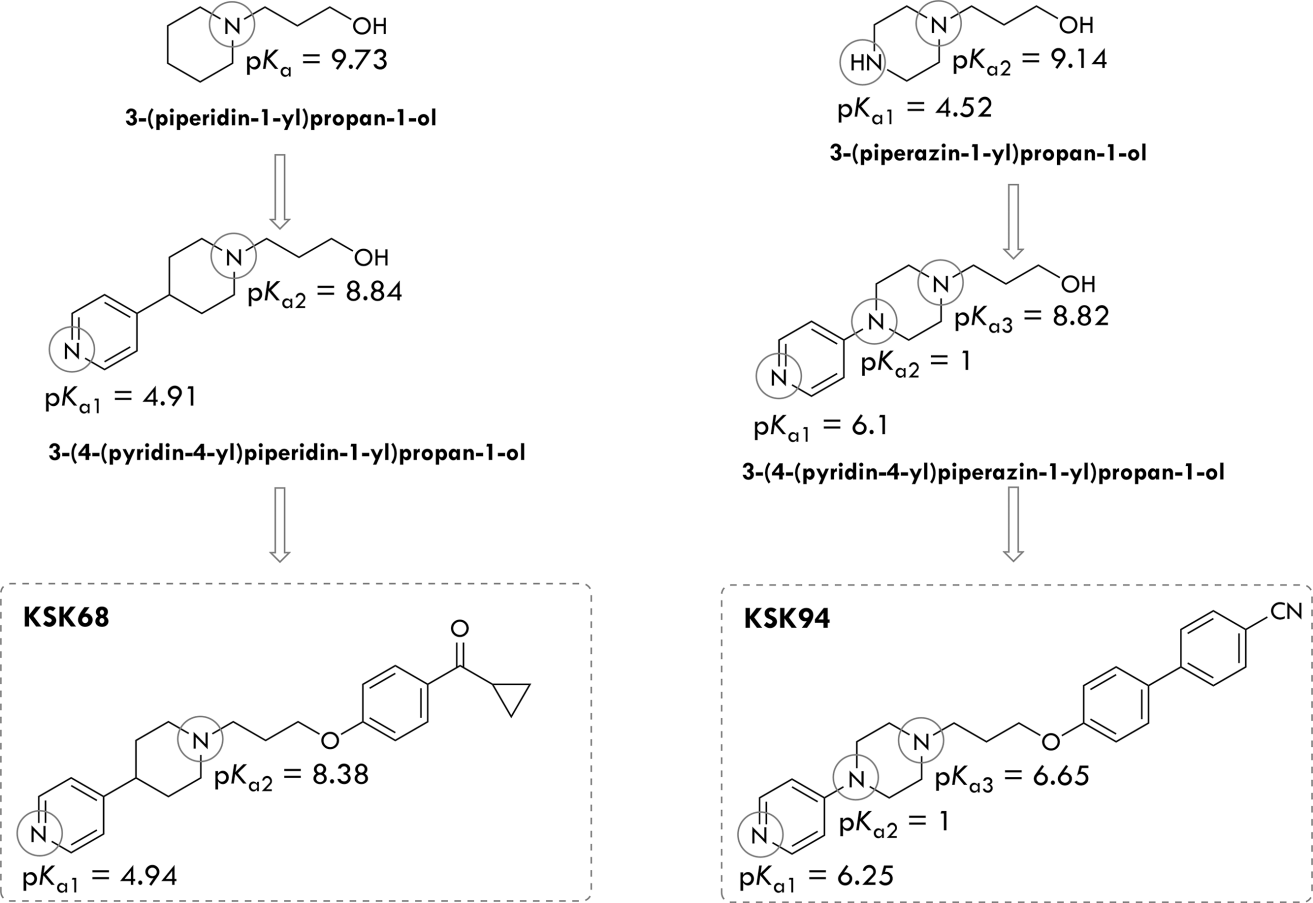
Structure and experimental p*K*_a_ values of KSK compounds and corresponding amino alcohols.

**Figure 5:**
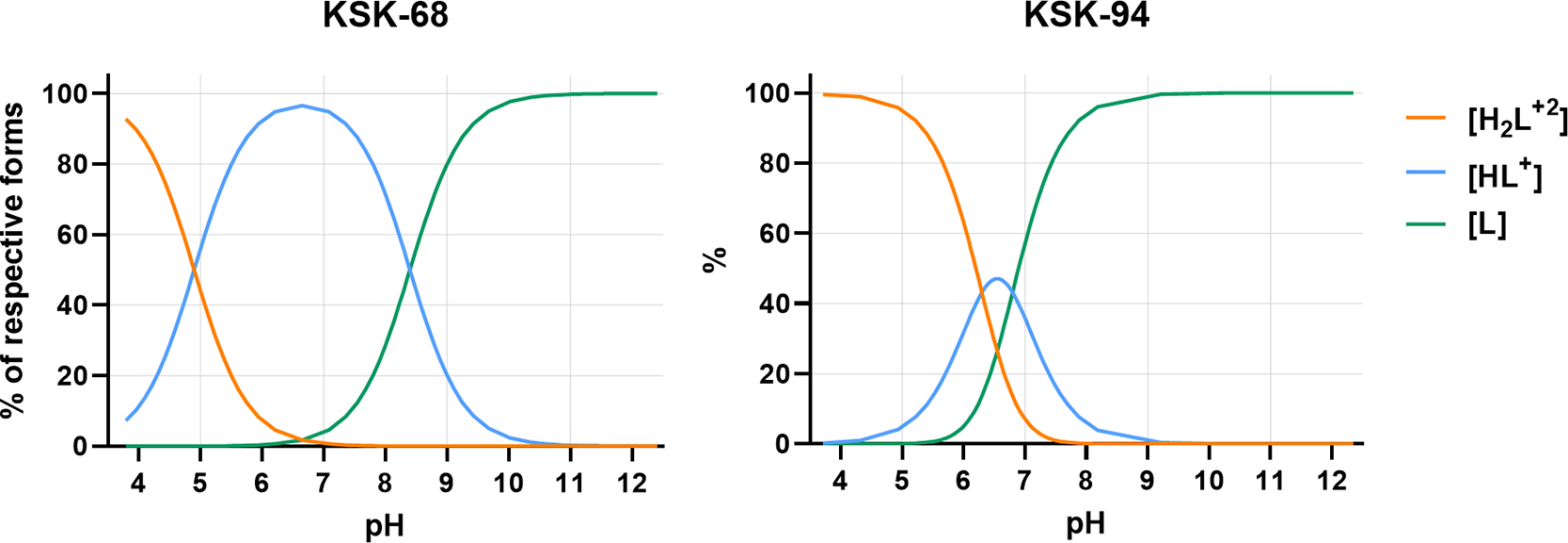
The protonation states of **KSK68** and **KSK94** as a function of pH. The species distribution was generated from the CerkoLab software based on titration curves of investigated compounds (see Figure S8). The equilibria between the species are as follows: **KSK68**: H_2_L^+2^ = H^+^ + HL^+^, p*K*_1_ = 4.94 and HL^+^ = H^+^ + L^0^, p*K*_2_ = 8.38; **KSK94**: H_2_L^+2^ = H^+^ + HL^+^, p*K*_2_ = 6.25 and HL^+^ = H^+^ + L^0^, p*K*_2_ = 6.65.

Thus, we evaluated two representative compounds, **KSK68** and **KSK94**, containing a piperidine or piperazine substituted with a 4-pyridyl moiety in p*K*_a_ studies. In addition, we tested the amino alcohol fragments of these compounds to carefully monitor the influence of individual protonated nitrogen atoms on the obtained p*K*_a_ values (Figure 4). Considering the structural differences between **KSK68** and **KSK94**, it seems obvious that their different binding potency toward σ_1_R should be attributed to the change in protonation state at physiological pH. Based on a previously modified potentiometric procedure,^27,28^ a series of titrations were performed, and the p*K*_a_ values were determined using the Kostrowicki and Liwo algorithm.^29,30^ Experimental p*K*_a_ data show that **KSK68** exists exclusively in the monoprotonated form in an aqueous solution at physiological pH (approximately 90% of [HL^+^] form, Figure 5). That explains the surprising result of free base crystallization attempts where iodide salt was obtained instead of the neutral form. On the other hand, in the same environment, **KSK94** exists in two forms: the monoprotonated form [HL^+^] (20.56%) and the free ligand [L] (77.72%, Figure 5). Experimental data show how replacing the piperidine system with a piperazine ring drastically affects the acid–base properties of the molecule. **KSK68** can be characterized as a basic ligand with p*K*_a1_ = 4.9 (pyridine N) and p*K*_a2_ = 8.4 (piperidine N). The p*K*_a_ values are consistent with those determined by the same procedure for the corresponding amino alcohol 3-(4-(pyridin-4-yl)piperidin-1-yl)propan-1-ol (Figure 4). In contrast to **KSK68**, the **KSK94** compound belongs to the group of ligands with an acidic center located at the N7 nitrogen of the piperazine ring with a p*K*_a2_ value of approximately 1 (Figure 4). A similarly low p*K*_a2_ value for the N7 piperazine nitrogen atom was also determined for the corresponding amino alcohol 3-(4-(pyridin-4-yl)piperazin-1-yl)propan-1-ol (Figure 4), which is in agreement with the value obtained for 1-phenyl-4-methyl piperazine reported in the literature.^31^ Moreover, the literature p*K*_a_ values of 1-aryl-4-propylpiperazines range from 7.59 to 8.39 for the N-CH_2_-CH_2_ moiety. The lowest value was determined for 4-nitrophenyl, and the highest was for 2-methylphenyl derivatives.^32^

### 2.4 Protonation Investigation Based on NMR Spectroscopy Measurements in the pH-Controlled Environment

Another attempt to determine the order of protonation of nitrogen atoms in studied molecules was carried out using NMR spectroscopy.

All signals in the ^1^H and ^13^C spectra were assigned based on 2D correlation spectra for compounds **KSK68** and **KSK94** (piperidine and piperazine derivatives, respectively) to follow the course of changes in their chemical shifts during titration with trifluoromethanesulfonic acid (triflic acid or TfOH), one of the strongest organic acids (p*K*_a_ ∼(−15)). A detailed description of the chemical shifts upon the addition of triflic acid is described in the Supporting Information.

**Figure 6:**
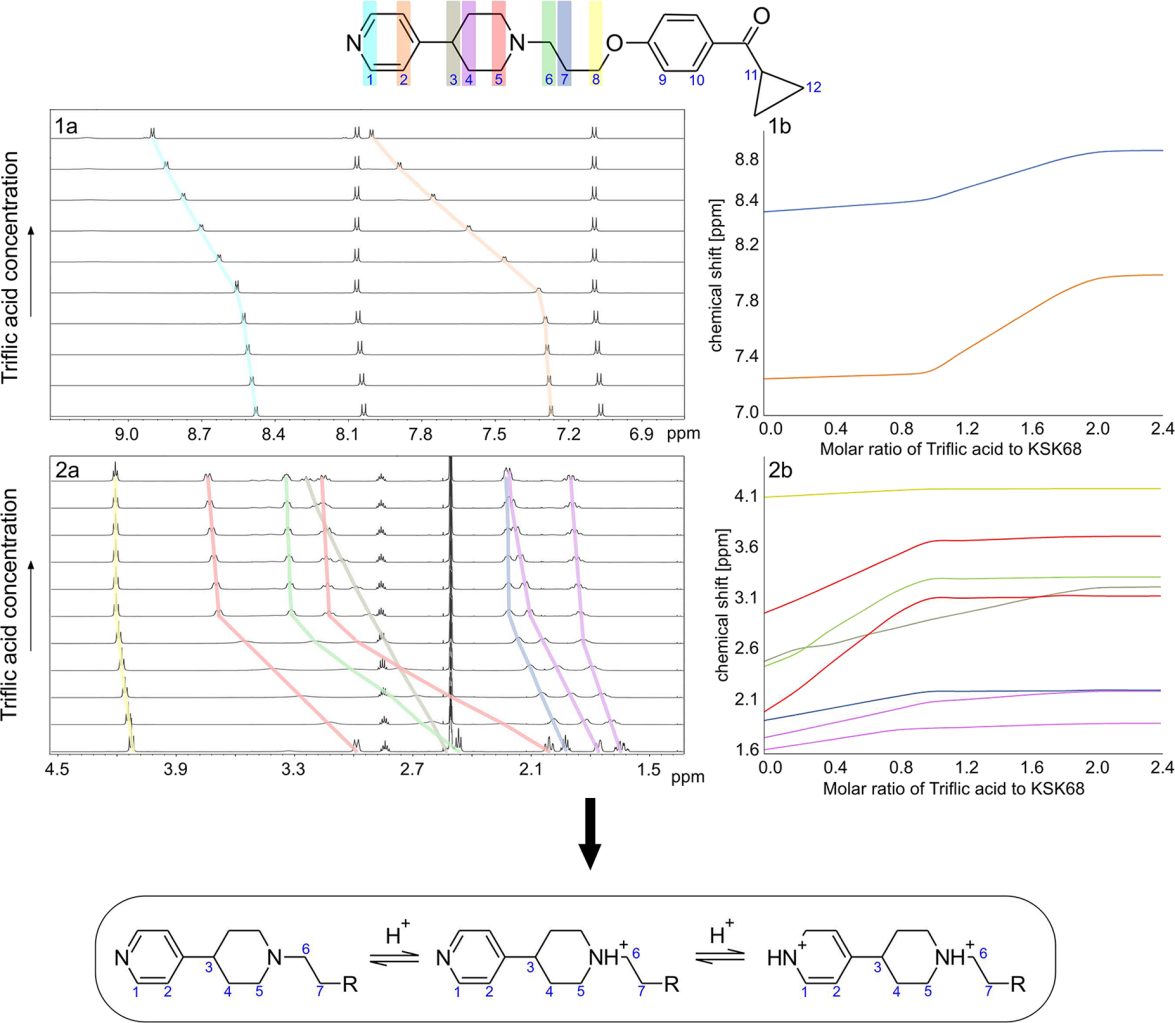
NMR titration experiment for **KSK68** presents stacked ^1^H NMR spectra of (1a) the aromatic region of chemical shifts and (2a) the aliphatic one. The concentration of acid is rising in an upward direction by 0.2 molar equivalents. (1b, 2b) Graphs that represent the change in the chemical shift of the individual signals on the ^1^H NMR spectra as the concentration of triflic acid in the solution increases. Panel 1b corresponds to the aromatic region of chemical shits and 2b to the aliphatic one. The colors on the top structure of **KSK68** refer to the signals marked on the spectra with exact color lines and to the colors of the lines on the graphs. The largest changes in the difference in chemical shifts ΔΔδ are observed for signals of protons that are adjacent to the nitrogen atoms, which undergo protonation. Tracking these changes in ΔΔδ while increasing the concentration of triflic acid in the solution allows one to assess the order of protonation of individual nitrogen atoms in the molecule (when nitrogen functions possess significant difference in p*K*_a_) and determine the integer number of nitrogen atoms that undergo protonation. For a detailed analysis, see the Supporting Information.

In the case of **KSK68**, the nitrogen atom in the piperidine ring is protonated first (Figure 6). Protonation of the nitrogen atom in the pyridine ring begins after full saturation to a molar ratio of 1.0:1.0. This is clearly visible in the shifts of protons in the vicinity of individual nitrogen atoms. The sole signal on the spectrum that shifts toward higher frequencies during the titration process comes from the H3 proton, which is located near both nitrogen atoms and links the two rings. The observed effect is characteristic for systems in which the p*K*_a_ values for the two basic centers differ significantly. Here, the p*K*_a_ for piperidine nitrogen is higher in value than that for pyridine nitrogen, which is in line with the theoretical data.

**Figure 7:**
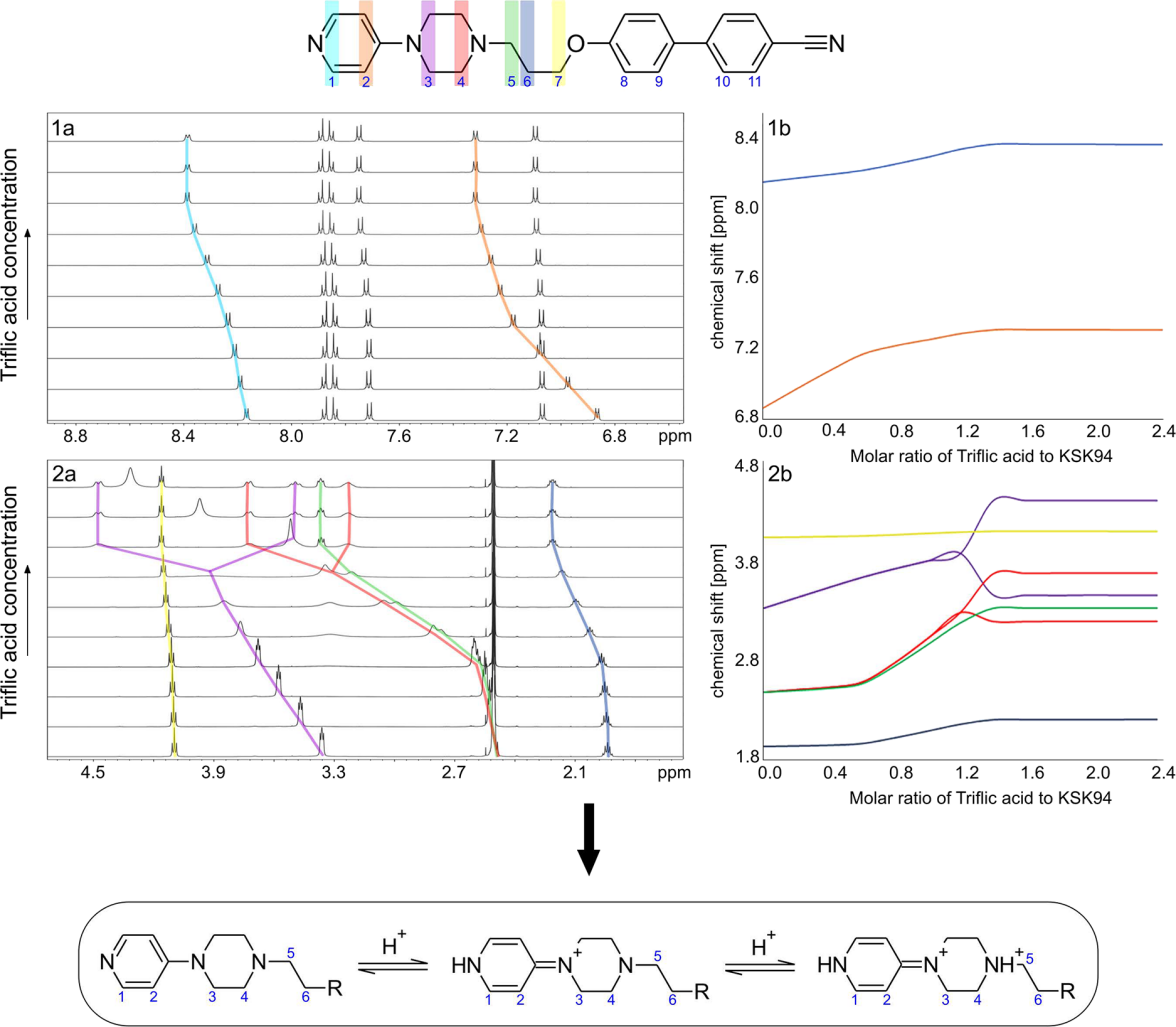
NMR titration experiment for **KSK94** presents stacked ^1^H NMR spectra of (1a) the aromatic region of chemical shifts and (2a) the aliphatic one. The concentration of acid is rising in an upward direction by 0.2 molar equivalents. (1b, 2b) Graphs that represent the change in the chemical shift of the individual signals on the ^1^H NMR spectra as the concentration of triflic acid in the solution increases. Panel 1b corresponds to the aromatic region of chemical shits and 2b to the aliphatic one. The colors on the top structure of **KSK94** refer to the signals marked on the spectra with exact color lines and to the colors of the lines on the graphs. The largest changes in the difference in chemical shifts ΔΔδ are observed for signals of protons that are adjacent to the nitrogen atoms, which undergo protonation. Tracking these changes in ΔΔδ while increasing the concentration of triflic acid in the solution allows one to assess the order of protonation of individual nitrogen atoms in the molecule (when nitrogen functions possess significant difference in p*K*_a_) and determine the integer number of nitrogen atoms that undergo protonation. For a detailed analysis, see the Supporting Information.

For **KSK94**, the model is more complicated than for **KSK68** (Figure 7). H1, H2, and H3 protons are affected by a change in the chemical environment at first, with H2 protons being the least affected. Thus, the proton environment is mostly changed in the proximity of the pyridine nitrogen atom and near the piperazine nitrogen closer to the pyridine. Taking further into account the difference of 94 ppm in ^15^N chemical shift for pyridine nitrogen, it can be concluded that the nature of the pyridine nitrogen atom changes dramatically from purely aromatic to an intermediate form between heterocyclic and amine. As a consequence, the pyridine nitrogen atom is protonated first, but the positive charge is transferred to the piperazine nitrogen atom by appropriate resonance structures. The second piperazine nitrogen atom is initially unprotonated, as indicated by no change in the chemical shift of proton signals from its immediate vicinity. From an acid molar ratio of 0.6:1.0, protonation of the second nitrogen atom in the piperazine ring can be observed. The proton signals in the vicinity of this nitrogen atom broaden by losing multiplicity and start to move toward higher frequencies. This is analogous to the first protonation step of **KSK68**. At this point, both nitrogen functions protonate at the same time, as both the aromatic and aliphatic proton signals shift toward higher frequencies, indicating similar p*K*_a_ values, with the value of the first stage being higher. Because of the loss of one symmetry element and likely conformational stiffening of the piperazine ring, the signals from the H3 and H4 protons, which were integrated as four protons, split into axial and equatorial components, and now each is integrated as two. At a ratio of 1.4:1.0, saturation occurs, and no further changes were observed despite reaching the molar ratio of 3:0:1.0.

### 2.5 *In Vitro* Pharmacology

#### 2.5.1 Affinity at H_3_R and σRs

*In vitro* affinity data for the newly synthesized ligands are assembled in Table 2. Interestingly, almost all compounds (except **16**) showed high affinity at histamine H_3_ receptors with *K*_i_ values below 100 nM. Moreover, all the described ligands showed more or less significant activity to both sigma receptors but with different binding affinities. In the case of compound **16**, the only piperazine derivative in this series, the lack of activity toward H_3_R is related to the pyrazine-2-yl group in the basic part of the molecule, as discussed in our previous work.^19,20^ Nevertheless, **16** was still very active on both sigma receptors (σ_1_R *K*_i_ = 7.6 nM, σ_2_R *K*_i_ = 27 nM). The unsubstituted piperidine ring in the basic part of the compounds seems to be the most influential on affinity at human H_3_R (hH_3_R), as can be seen by comparing compounds **5**–**7** with their 4-pyridyl analogues **12**–**14** (hH_3_R *K*_i_ = 6.2, 2.7, and 5.2 nM vs 7.7, 24.2, and 69 nM, respectively). Moreover, this structural relationship is also observed in the case of ligands **1** and **2**, which are methyl analogues of the lead compound **E377** (Figure 1) published in our previous paper^23^ (hH_3_R *K*_i_ = 31 and 10.3 nM vs 6.2 nM, respectively). However, for both sigma receptors, 4-pyridylpiperidine derivatives **12**–**14** were more potent than unsubstituted piperidines (**5**–**7**), especially when comparing affinity values toward sigma-1 receptors (σ_1_R *K*_i_ = 4.5, 5.6, and 3.3 nM vs 28, 18, and 4.8 nM, respectively; σ_2_R *K*_i_ = 10, 4, and 29 nM vs 47, 103, and 116 nM, respectively). For all the described piperidine derivatives, the effect of the alkyl chain can only be observed at the H_3_R, where the extension of the linker length decreased the affinity of biphenyl analogues **9**, **10**, and **11** (hH_3_R *K*_i_ = 22, 21.7, and 88.9 nM, respectively). In our previous work, we found a critical structural feature that distinguishes the dual H_3_/σ_1_Rs ligands and selective H_3_R-targeting compounds.^23^ One of the lead structures, **KSK68** with the 4-pyridylpiperidine moiety as the fundamental part of the molecule, showed high affinity at both histamine H_3_ and σ_1_ receptors, whereas its piperazine analogue **KSK67** was highly selective for H_3_R (Figure 1). Interestingly, this time, we also confirmed this structural phenomenon, as can be seen by comparing the nitrile derivative **12** with its piperazine analogue **KSK94** described in our previous work (Figure 1, hH_3_R *K*_i_ = 7.7 nM and σ_1_R *K*_i_ = 4.5 nM vs hH_3_R *K*_i_ = 7.9 nM and σ_1_R *K*_i_ = 2958 nM, respectively). Again, the piperidine derivative **12** showed dual H_3_/σ_1_Rs activity, whereas the piperazine-based compound **KSK94** was highly selective for H_3_R. Taking into account the selectivity toward sigma-2 receptors in the group of biphenyl derivatives with three-carbon chains, the highest values were observed for compounds **1** and **3** (σ_2_/σ_1_ ratio 11.3 and 13.2, respectively). Furthermore, extending the carbon chain from four to six methylene groups significantly reduced this parameter (ligands **9**–**11**). Aniline derivative **7** showed the highest σ_2_/σ_1_ ratio among all described derivatives (24.2).

**Table 2:**
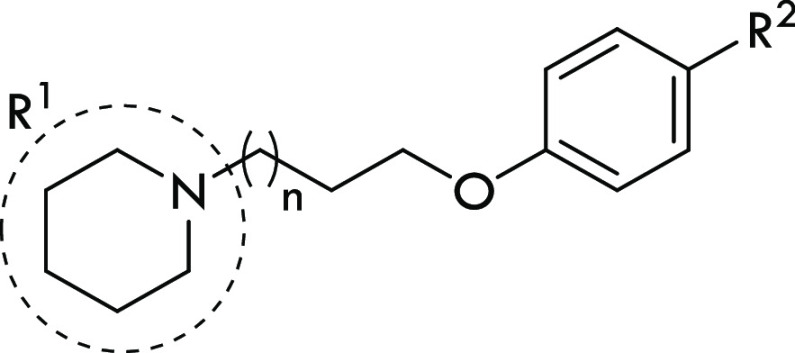

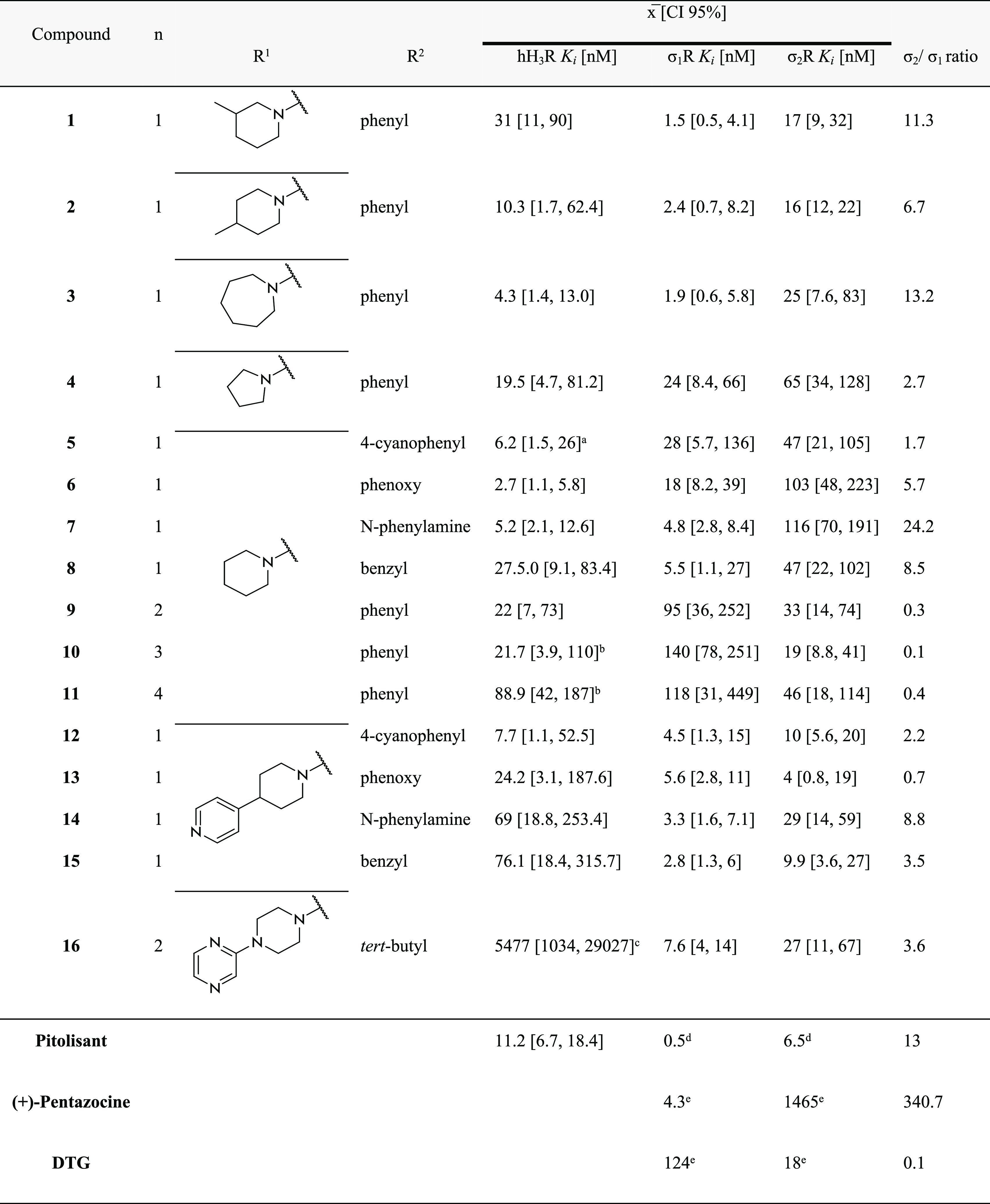
Structures of Compounds **1***–***16** and Their *in Vitro* Binding Affinities at the Human Histamine H_3_ Receptor (hH_3_R) and Rat Sigma-1 (σ_1_R) and Sigma-2 (σ_2_R) Receptors^f^

a Data published in ref (25).

b Recalculated from data published in ref (24).

c Data published in ref (19).

d Data published in ref (7).

e Data published in ref (61).

f Given data represent mean values within the 95% confidence interval (CI).

As lead structures for further evaluation, we selected two piperidine derivatives, **3** and **7**, with high affinity at both H_3_R and σ_1_R as well as the highest σ_2_/σ_1_ selectivity factor. In this choice, we were guided by the fact that the highly selective σ_1_R antagonist **S1RA**, so far the only one tested in clinical trials for pain treatment, showed no affinity at σ_2_R.^18^ On the other hand, bearing in mind recently reported studies with novel σ_2_R-selective ligands, which suggest that they may also modulate nociception,^33^ we included nitrile derivative **12** exhibiting activity toward σ_2_R at a comparable level as toward H_3_R and σ_1_R. Moreover, its piperazine analogue, compound **KSK94**, was extensively tested in the obesity model in our previous studies and showed a good safety profile.^22^

#### 2.5.2 Affinity at Other Histamine Receptors

To check the selectivity profile of our lead structures, radioligand binding studies at other histamine receptor subtypes were carried out. Compounds **3**, **7**, and **12** in their oxalate forms were tested at human recombinant histamine H_1_, H_2_, and H_4_ receptor subtypes stably expressed in HEK293T cells. Obtained results clearly indicate the high selectivity of the tested derivatives toward human H_3_R (Table 3).

**Table 3:** Radioligand Binding Assay at Human Histamine H_1_ (hH_1_R), H_2_ (hH_2_R), and H_4_ (hH_4_R) Receptors and Functional Study at the Human Histamine H_3_ Receptor (hH_3_R)^a^

compound	hH_1_R (*K*_i_ [nM] x̅ [CI 95%])	hH_2_R (*K*_i_ [nM] x̅ [CI 95%])	hH_4_R (*K*_i_ [nM] x̅ [CI 95%])	hH_3_R (IC_50_ [nM] ± SD)
**3**	7615 [4024; 14,407]	>10,000	>10,000	83.4 ± 15.9
**7**	>10,000	>10,000	>10,000	83.7 ± 2.1
**12**	653 [259; 1647]	>10,000	>10,000	215 ± 108
thioperamide				375 ± 30
pitolisant				1.96 ± 0.80

a Given data represent the mean *K*_i_ (nM) values within the 95% confidence interval (CI) (*n* = 3, triplicate) or the mean IC_50_ values of tested compounds in the cAMP assay ± standard deviation (*n* = 2, triplicate).

#### 2.5.3 Intrinsic Activity toward H_3_R

To identify the lead compounds’ functional profile, their intrinsic activity was tested in a 3′,5′-cyclic adenosine monophosphate (cAMP) accumulation assay in HEK cells expressing human recombinant H_3_R.^34^ All tested compounds reversed the *R*-α-methylhistamine inhibition of cAMP production in forskolin-stimulated cells and were therefore classified as H_3_R antagonists/inverse agonists. The IC_50_ values obtained in the H_3_R functional assay are assembled in Table 3.

### 2.6 *In Silico* Studies. Molecular Modeling: Docking Studies and Molecular Dynamics Simulations

**Figure 8:**
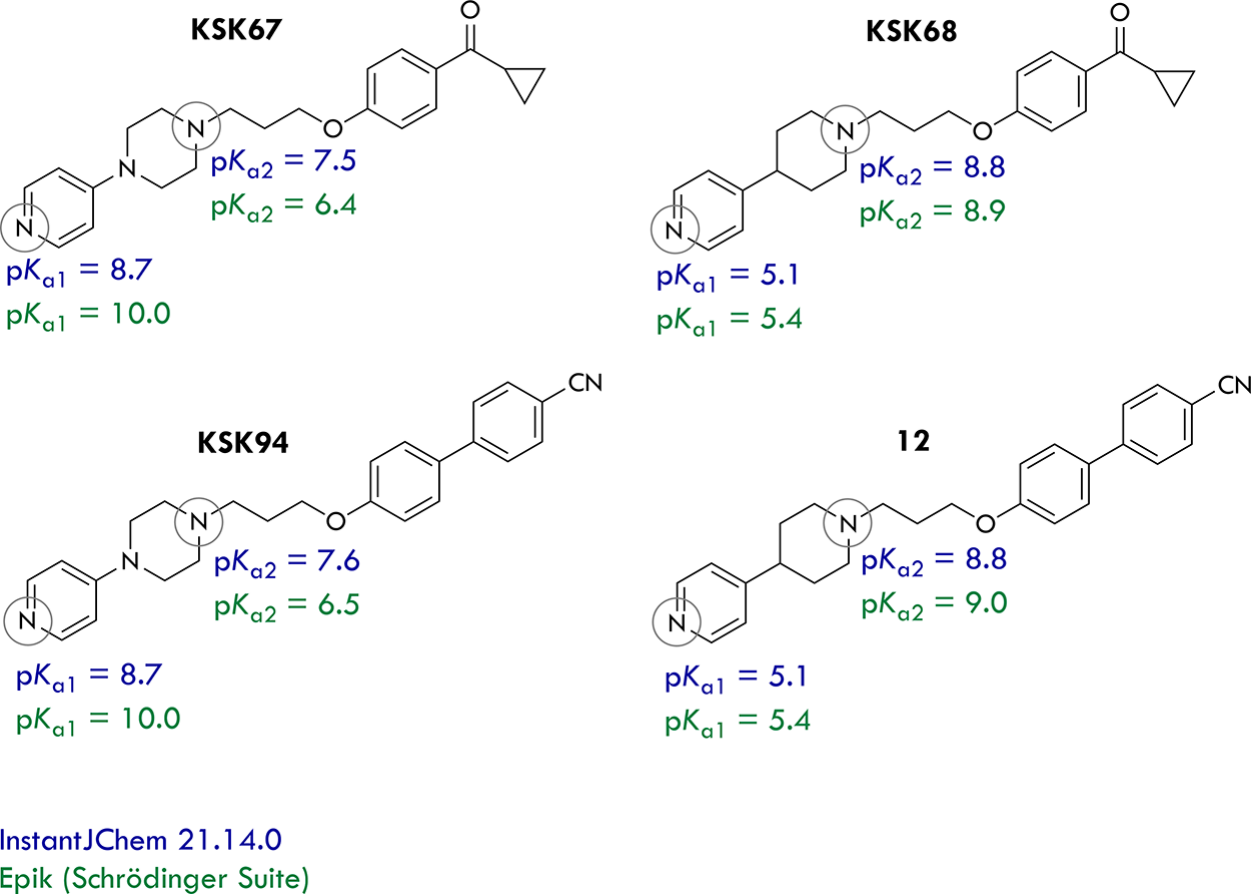
Results of *in silico* protonation studies of compounds **KSK67**, **KSK68**, **KSK94**, and **12**.

In addition to experimental studies, the protonation states of compounds were also evaluated *in silico* using two software packages: InstantJChem and Epik (Figure 8). Although there are slight differences in the numerical values of predicted p*K*_a_, the overall tendencies observed for the performed experiments are preserved: nitrogen atoms belonging to the piperidine moieties are characterized by higher p*K*_a_ values than those of piperazine, and therefore, they are less likely to protonate at physiological pH.

**Figure 9:**
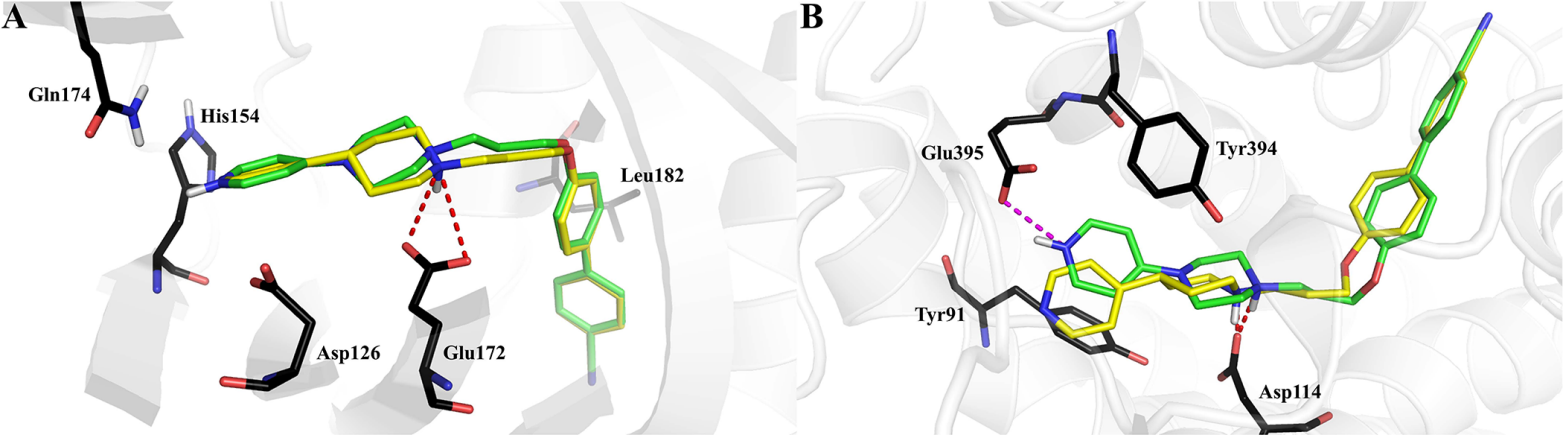
Comparison of the binding mode of **KSK94** (green) in tautomeric monoprotonated state and **12** (yellow) in the σ_1_R (PDB ID: 6DJZ) (A). Comparison of the binding mode of **KSK67** (green) in the tautomeric monoprotonated state and **KSK68** (yellow) in the H_3_R binding site (homology model) (B). The corresponding salt bridges are marked in red and magenta.

To further investigate the influence of structural differences between the 4-pyridylpiperazine and 4-pyridylpiperidine moieties on their molecular mechanism of action, the binding with sigma-1 and histamine H_3_ receptors was evaluated using molecular modeling methods. Analysis of the binding mode of **KSK94** and **12** to σ_1_R shows coherent binding modes of 4-pyridylpiperidine/piperazine fragment in comparison to our previous studies^22,23^ (Figure 9A). However, because of the use of induced-fit docking, which allows relaxing the binding pocket and fitting of adjacent amino acids to the molecular core of compounds, we observed that the 4-cyanophenyl fragment occupies a hydrophobic pocket with the stabilizing hydrogen bond (HB) between the nitrile group and the main chain of Ser99. Then, on the basis of both NMR analysis and crystallographic results, we decided to dock all protonation and tautomeric states of **KSK94** and **12** using *ab initio* docking (quantum polarized ligand docking, QPLD) and energy calculations applying the Molecular Mechanics Generalized Born Surface Area (MM-GBSA) method (Table S5, SI). First, we estimated the energy loss of piperazine derivatives to piperidine ones for σ_1_R. The result showed that the tautomeric monoprotonated pyrimidine structure (the most populated protonation state) of **KSK94** has a higher Δ*G* value vs **12** (Table 4), indicating a loss of ligand–receptor interaction energy, which is also reflected in the *in vitro* results (Table 2). In the case of the monoprotonated tautomeric form, a loss of the essential salt bridge interaction with Glu172 (Figure 9A) can explain the decrease in biological activity. A similar correlation between *in vitro* results and Δ*G* value can be noticed for **KSK67** and **KSK68** compounds (Table 4). When analyzing the interactions of lead compounds, it is worth noting that ligands **3** and **7** occupy exactly the same place as **12** despite the lack of a heteroaromatic ring connected to the saturated basic fragment (Figure S25). Notably, enriched by the assembled experimental evidence and observations, in this study, more complex and sophisticated molecular modeling methods were applied compared to the previous studies,^23^ resulting in different binding modes observed. Analysis of the previously described **KSK68** binding mode showed significant changes compared to the results presented here, where a salt bridge with Asp114 (D3.32) was formed. However, the two compound poses are flipped with respect to each other. In our earlier studies, the conformation of **KSK68** was almost linear (Figure S24), whereas in the current study, the conformation resembles the one observed for the sigma-1 receptor (Figure 9A,B). In fact, four distinct binding modes of H_3_R ligands have been reported so far in the literature;^35−44^ however, we decided to focus on the one that does not involve Glu206. This orientation was also similar to the alternative binding modes of ciproxifan in the H_3_R binding pocket.^37,45^ Because of the expansion of the tertiary amine fragment compared to ciproxifan (replacement of imidazole with 4-pyridylpiperazine), compound **12** additionally forms contacts with Tyr91 (TM3), Tyr394 (TM7), as well as Glu395 (TM7). To explain the slight difference in the biological activity of **KSK94** and **12** toward H_3_R (Table 2), *ab initio* molecular docking of all protonation and tautomeric forms was performed (Table S5, SI). The results showed that for the monoprotonated tautomeric form of **KSK94**, the loss of the ligand–receptor interaction energy (Δ*G*) is insignificant (Table 4), which is related to formation of an additional stabilizing salt bridge with Glu395, which also correlates with the biological activity observed. Moreover, the compounds occupy the same region of the binding pocket, and because of the nature of the homology modeling procedure applied to generate the H_3_R model, alternative binding mechanisms cannot be excluded. For the monoprotonated tautomeric form of **KSK67**, compared to **KSK68**, the Δ*G* values are close, which correlate well with the experimental results (Table 4). The observed salt bridge with D3.32 (the strongest interaction in ligand–receptor interactions)^46^ fixes the position occupied by the ligands, which can be seen by comparing lead compounds **3** and **7** (Figure S26). The *N*-phenylamine (**7**) or phenyl (**3**) fragment penetrates deep into the binding pocket because of its hydrophobic environment and, as with compound **12**, interacts through hydrophobic effects or π–π stacking interactions (Figure S26).

**Table 4:**
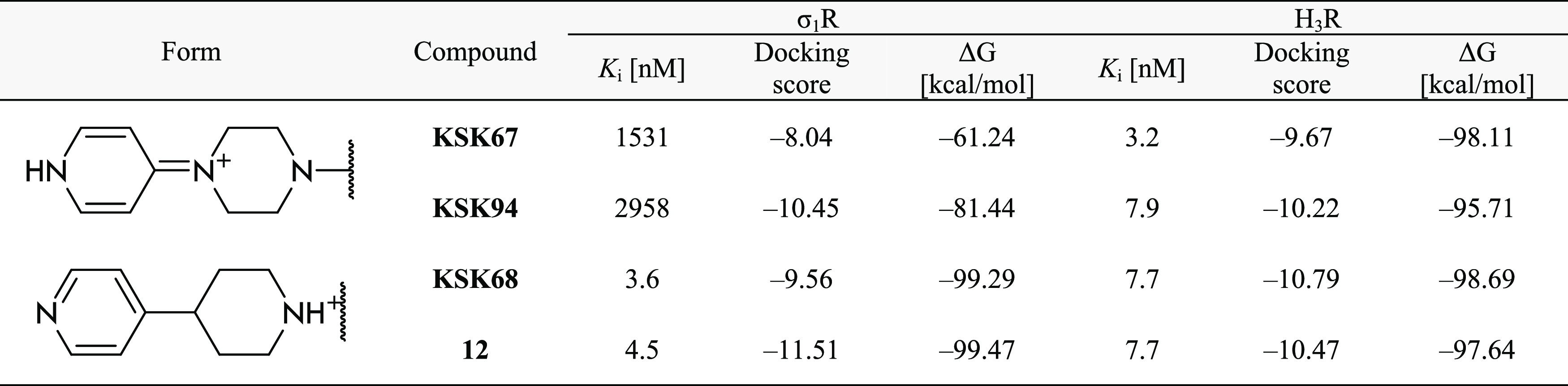
Comparison of Free Binding Energies and Docking Scores of the Most Populated Protonation States with the *In Vitro* Activity of **KSK67**, **KSK68**, **KSK94**, and **12** with σ_1_ and H_3_ Receptors

To confirm the validity of the docking poses discussed above, the molecular dynamics (MD) simulations were carried out. The obtained data were analyzed both in terms of the variation in the ligand-protein contacts as well as *via* the examination of ligands’ RMSDs. Both approaches confirmed the stability of the obtained docking poses: the compounds occupied the same region of the binding pocket during the whole simulation process, and the patterns of ligand–protein interactions in general remain the same (Figures S27 and S28).

We are aware of the H_3_R crystal structure, which was released in late October 2022 in the PDB repository (PDB ID: 7F61).^47^ The crystalized complex contains an antagonist in the binding pocket, PF03654746, as well as the unexpected cholesterol binding at the allosteric site. According to the authors, the H_3_R orthosteric binding pocket is shallow, and PF03654746 occupies an extended part of it (EBD). To evaluate the quality of our homology model, we docked PF03654746 to the model, and we obtained a similar pose to that observed in the crystal structure (Figure S29). The RMSD (root-mean-square deviation) of ligands was 1.48 Å, and it is less than the crystal resolution (2.6 Å), which indicated that our model was coherent with the crystal structure. It is worth to stress that the proposed binding mode of KSK ligands engaged the same protein helices (especially TM7) and crucial amino acids.^47^ For this reason, we decided not to perform *in silico* calculations once again using an experimental H_3_R structure.

### 2.7 Determination of Selected ADMET Parameters

#### 2.7.1 Permeability Profile

During the research and development of new drugs directed to the central nervous system, there is a considerable attrition rate caused by their hampered access to the brain by the blood–brain barrier. Throughout the years, several *in vitro* models have been developed in an attempt to mimic critical functionalities of the blood–brain barrier and reliably predict the permeability of drug candidates. Therefore, the ability of selected compounds (**3**, **7**, **12**, and **S1RA**) to penetrate across lipid membranes was estimated by a parallel artificial membrane permeability assay (PAMPA). In medicinal chemistry, PAMPA is a method that determines the permeability of substances from a donor compartment through a lipid-infused artificial membrane into an acceptor compartment; passive diffusion is the predominant absorption mechanism of most commercial drugs.^48^ By using UPLC–MS with an internal standard, the exact quantity of molecules that penetrated from donor to acceptor wells through the phospholipid membrane was measured. The results were expressed as permeability coefficient *P*_e_ calculated according to the formulas described in the literature.^49^ Taking into account the collected data and comparing them with two standards: well-permeable caffeine (*P*_e_ = 10.49 × 10^–6^ cm/s) and low-permeable sulpiride (*P*_e_ = 0.05 × 10^–6^ cm/s), it seems justified to consider the tested molecules as substances with highly passive transport through biological membranes (Table 5). In general, compounds with a *P*_e_ value above 4 × 10^–6^ cm/s are considered permeable, whereas those with a lower value are considered impermeable. All tested ligands showed *P*_e_ values greater than the threshold value.

**Table 5:** PAMPA Results (Permeability Coefficient *P*_e_) for Tested Compounds

compound	*P*_e_ (10^–6^ cm/s)
**S1RA**	5.26
**3**	6.57
**7**	10.63
**12**	12.00
caffeine	10.49
sulpiride	0.05

Moreover, taking into account an intracellular localization of sigma receptors, the high ability of tested compounds to penetrate lipid membranes and, at the same time, the possibility of interacting with σ_1_R seem to be an important feature of dual histamine H_3_/σ_1_R ligands.

#### 2.7.2 Serum Metabolic Stability

**Figure 10:**
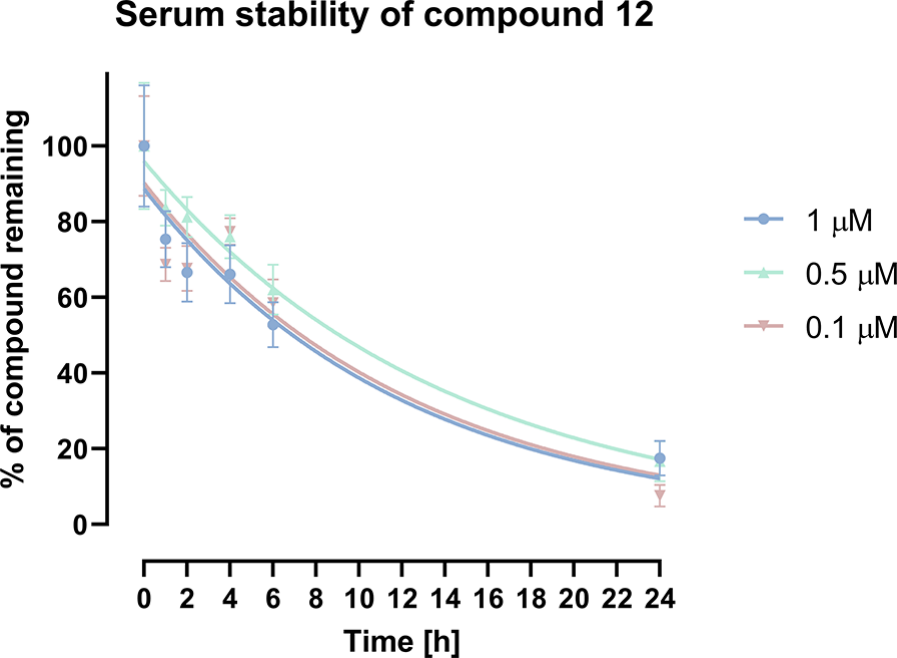
Serum stability of compound **12**. The % of test compound remaining in the serum is plotted vs time, and the curve is fitted using a one phase decay model.

A successful drug-lead candidate must possess favorable characteristics, including, among others, good serum stability; therefore, we decided to test this parameter for compound **12**. Using LC–MS with imipramine as an internal standard, the exact amount of molecule remaining in the serum was measured at six time points (0, 1, 2, 4, 6, and 24 h) for three initial concentrations (0.1, 0.5, and 1 μM). The response ratio was calculated by dividing the test peak area by the internal standard peak area and converted to % of test compound remaining in the serum. The % of test compound remaining was then plotted vs time (Figure 10). Upon the curve fitting using a one phase decay model, the half-life parameter was calculated to characterize the serum stability of compound **12** (*t*_1/2_ = 7.7 ± 1.4 h). As a reference drug, we used highly stable atenolol at an initial concentration of 1 μM. The % of atenolol remaining in the serum after 24 h of incubation was significantly higher than compound **12** (82.3 vs 17.5%); thus, **12** can be considered a moderately stable ligand.

### 2.8 *In Vivo* Pharmacological Activity

#### 2.8.1 Safety Pharmacology: Influence of Lead Compounds on Spontaneous Locomotor Activity and Motor Coordination in the Rotarod Test

The high sedative activity of analgesics is considered an undesirable property, which may lead to an incorrect or ambiguous interpretation of the *in vivo* results. Moreover, it may limit the potential clinical application of new drug candidates. Therefore, we tested the influence of compounds **3**, **7**, and **12** on spontaneous locomotor activity to investigate their sedative properties. The data presented in Figure S30 indicate that compounds **3** and **7** at the dose of 15 mg/kg showed high and significant sedative effects (*F*_(2,21)_ = 28.36; *p* < 0.0001). In contrast, **12** at the same dose decreased spontaneous locomotor activity, but the effect was not statistically significant, which proved the less pronounced sedative property of this compound. It significantly decreased locomotor activity at the dose of 30 mg/kg (*F*_(3,32)_ = 4.97; *p* < 0.01). The estimated ED_50_ value for the ligand in this test was 21.97 mg/kg (Figure S31).

The neurotoxicity of the new compounds can result in impaired motor functions of the experimental animals, which may subsequently cause false interpretation of the results of *in vivo* tests. Understandably, neurotoxicity is also the frequent reason for eliminating the tested compound from further studies. To minimize that risk, we investigated the influence of our lead structures on motor coordination in the rotarod test, which is the classical test used for assessing motor coordination and balance in rodents. It provides a quick and simple estimation of neuromuscular coordination. During our test, the vehicle-treated mice did not demonstrate any signs of impaired motor coordination. The time spent on the rotarod apparatus was 60 s for each control mouse. Compound **7** at the dose of 15 mg/kg significantly decreased the time spent on the rod at 18 and 24 rpm (*F*_(3,20)_ = 31.32; *p* < 0.0001). Although the effect of **3** was less pronounced, the compound also significantly impaired the motor coordination of mice at the administrated dose (15 mg/kg) and two tested speeds (18 and 24 rpm) (*F*_(3,20)_ = 3.33; *p* < 0.05). Compound **12** at the same conditions slightly affected motor coordination, but the effect was not statistically significant (*F*_(3,20)_ = 2.31; *p* = 0.107) (Figure S32). The results showed that **12** is characterized by a better safety profile than the other two tested compounds.

#### 2.8.2 Antinociceptive Activity in the Formalin Test

**Figure 11:**
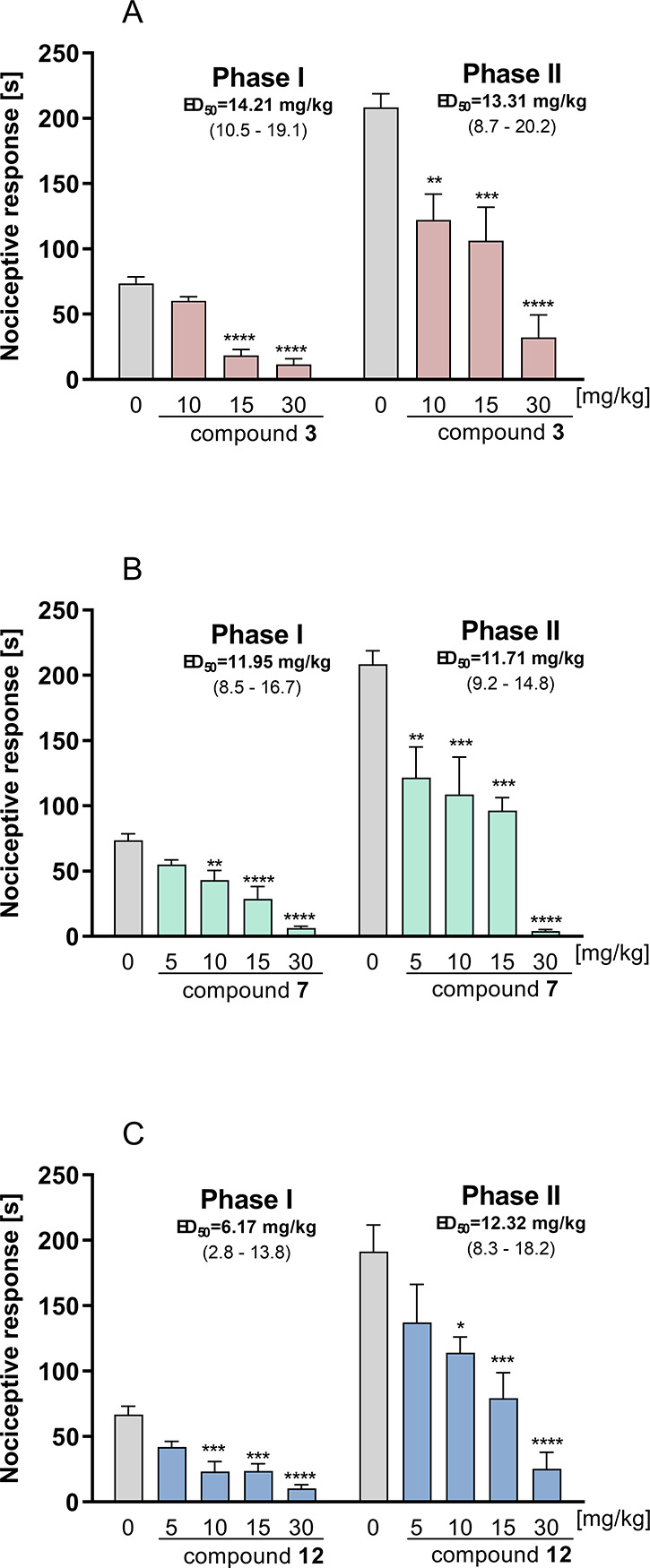
Antinociceptive activity of **3** (A), **7** (B), and **12** (C) in the formalin test. Results are shown as time of licking in phase I (0–5 min after intraplantar injection of formalin) and in phase II (15–30 min after formalin injection). Each value represents the mean ± S.E.M. for 8–10 animals. 0: vehicle (1% Tween 80). Statistical analysis: one-way ANOVA followed by *post hoc* Dunnett’s test. Statistical significance compared to vehicle-treated animals: *p < 0.05, **p < 0.01, ***p < 0.001, and ****p < 0.0001.

The formalin test is one of the most useful screening models for testing potentially clinically relevant antinociceptive molecules because of its ease of administration, standardization, and validation with reference drugs.^50^ The subcutaneous injection of formalin results in a focal injury that stimulates and then damages sensory endings. Two distinct phases of nociceptive response are associated with the immediate activation of nociceptors and sensitization of spinal reflex circuits during phase I and II responses, respectively.^51^ Moreover, it has been recently suggested that formalin injection results in pathological changes that resemble those observed in nerve injury.^52^ The intraperitoneal (i.p.) administration of compound **7** prior to the subcutaneous injection of formalin significantly affected the duration of the nociceptive response in the acute phase of the formalin test (*F*_(4,40)_ = 21.36; *p* < 0.0001). This ligand also significantly attenuated the late phase of the test (*F*_(4,40)_ = 16.50; *p* < 0.0001). The calculated ED_50_ value in phase I was found to be 11.9 mg/kg, whereas the ED_50_ value in phase II was found to be 11.7 mg/kg (Figure 11b). The treatment with **3** resulted in a significant decrease in nociceptive response in both the acute phase (*F*_(3,34)_ = 43.20; *p* < 0.0001) and the late phase (*F*_(3,32)_ = 17.63; *p* < 0.0001). The potency of the compound expressed as the ED_50_ value was slightly lower than that obtained for the previous compound and was 14.21 and 13.31 mg/kg for the acute and late phases, respectively (Figure 11A). Ligand **12** showed high analgesic activity in the acute phase (*F*_(4,40)_ = 13.73; *p* < 0.0001) and late phase (*F*_(4,40)_ = 9.51; *p* < 0.0001) of the test (Figure 11C). Interestingly, the obtained ED_50_ values (6.17 and 12.32 mg/kg for the acute and late phase, respectively) reveal the particularly high potency of this compound in the acute, neurogenic phase, which suggests that it may affect the mechanisms of neurotransmitter release from neuronal cells.

Taking all data from preliminary studies, we decided to select compound **12** for further analysis of its analgesic activity. Although all three compounds showed significant activity in the formalin test, the safety profile of **12** was better than the other ligands. The chosen compound did not affect motor coordination significantly, and its ED_50_ value in the formalin test was almost two times lower than that obtained in the evaluation of the sedative activity. We used other models of pain to assess the range of analgesic activity of the compound with particular emphasis on neuropathic pain of different origins.

#### 2.8.3 Effects of **12** on Capsaicin-Induced Nociception

Because compound **12** had a strong effect on the acute phase of the formalin test, we decided to evaluate its activity in capsaicin-induced pain, which is another model of neurogenic pain. Formalin-induced pain is mainly dependent on the chemical stimulation of the TRPA1 receptor on somatosensory nerve endings, whereas capsaicin is an agonist of TRPV1 receptors.^53^

**Figure 12:**
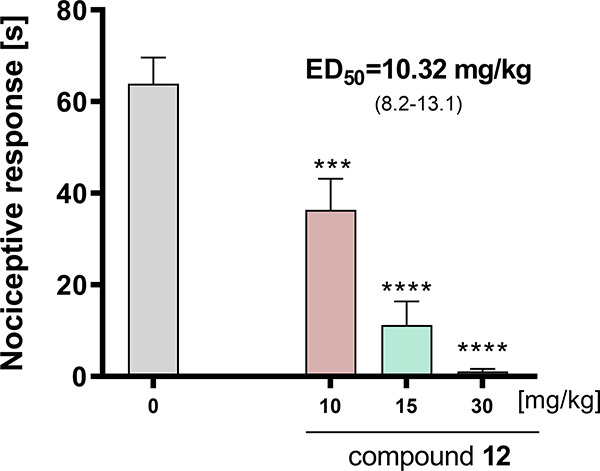
Antinociceptive activity of compound **12** in the capsaicin test. Results are shown as time of nociceptive response in 5 min period after intraplantar injection of capsaicin. Each value represents the mean ± S.E.M. for 8–10 animals. Statistical analysis: one-way ANOVA followed by *post hoc* Dunnett’s test. Statistical significance compared to vehicle-treated animals (Tween): ^****^p < 0.0001. 0: vehicle treated mice (1% Tween 80).

As shown in Figure 12, the selected compound significantly decreased the paw licking or biting behavior in that test at all administrated doses (10, 15, and 30 mg/kg) (*F*_(3,34)_ = 28.48, *p* < 0.0001). The ED_50_ value calculated for **12** in the capsaicin test was 10.32 mg/kg.

The high potency of **12** in both formalin- and capsaicin-induced pain shows that this compound has the potential to attenuate neurogenic pain independent of the mechanism of its induction. This suggests a broad spectrum of activity of this ligand.

#### 2.8.4 Effects of **12** on Loperamide-Induced Antinociception

**Figure 13:**
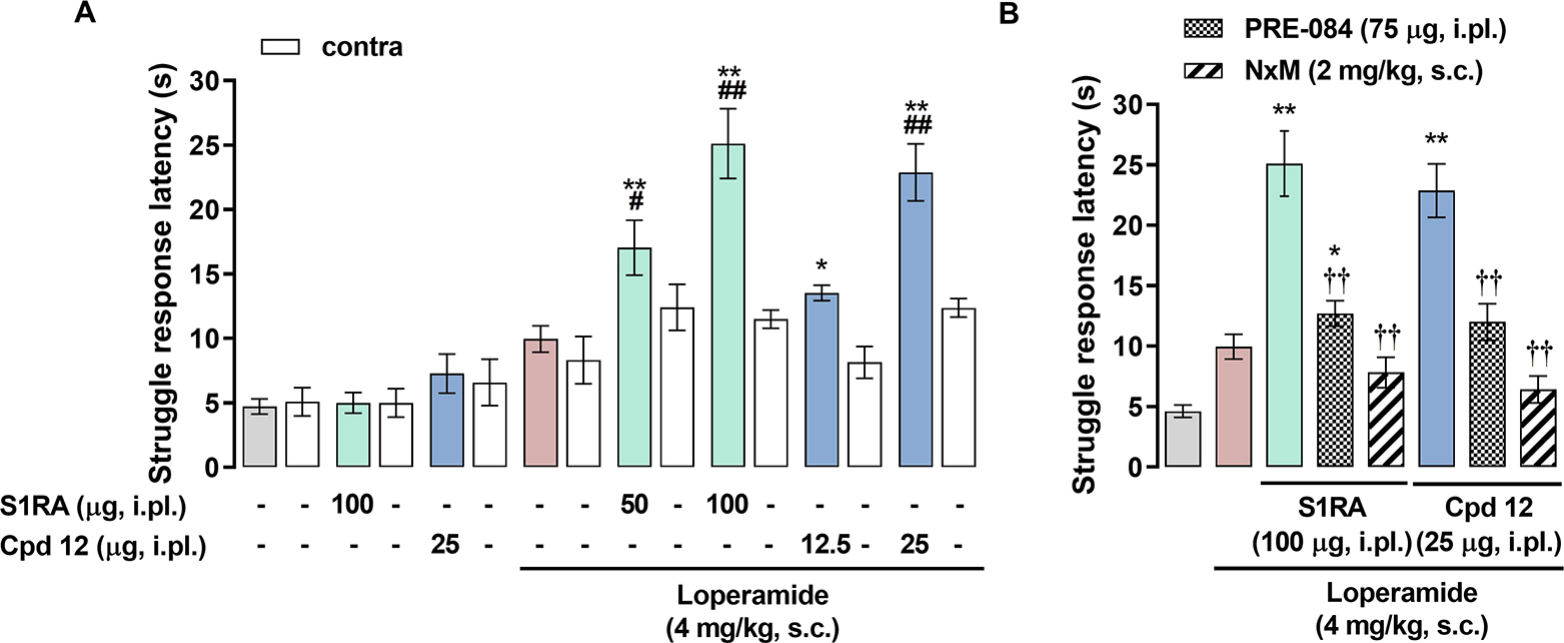
Effects of **S1RA** and **12** on loperamide-induced antinociception. The results represent the struggle response latency during stimulation with 450 g pressure in mice intraplantarly (i.pl.) administered **S1RA** (50–100 μg), **12** (12.5–25 μg), or saline and treated subcutaneously (s.c.) with loperamide (4 mg/kg) or its solvent (1% DMSO in ultrapure water). (A) Effect of treatments on the response latency to mechanical stimulation in the paw i.pl. injected with the σ_1_R ligands (ipsi) and in the contralateral paw (contra). (B) Effect of the i.pl. administration of PRE-084 (75 μg) and the s.c. administration of naloxone-methiodide (NxM, 2 mg/kg) on the potentiation of loperamide-induced antinociception by **S1RA** and **12**. Each bar and vertical line represent the mean ± SEM of values obtained in six to eight animals. Two-way analysis of variance followed by the Bonferroni test was used to determine statistically significant differences (A and B) between the values obtained in the group treated with the solvent of the drugs and the rest of the groups (*p < 0.05, **p < 0.01), (A) between the ipsi and the contra paws (##*p* < 0.01), and (B) between the values of the ipsi paw from loperamide-treated mice injected with **S1RA** or **12** alone and coadministered with PRE-084 or with the association with NxM (††*p* < 0.01).

As it is well known that σ_1_R antagonism enhances opioid analgesia, we tested the effects of compound **12** on antinociception induced by the opioid agonist loperamide. **S1RA** was used as a σ_1_R reference antagonist and administered intraplantarly (i.pl.) at two doses of 50 and 100 μg. Compound **12** was tested at 12.5 and 25 μg. The antinociceptive effect of the treatments was tested in mice by monitoring the latency to struggle in response to a nociceptive mechanical stimulus applied to the paw. The subcutaneous (s.c.) administration of loperamide (4 mg/kg) induced a minimal (nonsignificant) increase in the struggle response latency in comparison to the values from mice treated with its solvent (Figure 13). The administration of **S1RA** alone (100 μg) did not change the response to the mechanical stimulus but significantly and dose-dependently (50–100 μg) increased the antinociceptive effect induced by loperamide and only in the paw injected with the σ_1_R antagonist (Figure 13). The administration of compound **12** (25 μg) did not have any effect *per se* but dose-dependently (12.5–25 μg) increased the antinociceptive effect of loperamide at the injected paw, mirroring the effects induced by **S1RA** (Figure 13). Hence, the potency of compound **12** to enhance loperamide-induced antinociception was much higher than **S1RA**. Notably, the coadministration of the σ_1_R agonist PRE-084 (75 μg) with **S1RA**, and also with **12**, was able to markedly reverse the effect of these compounds on loperamide-induced antinociception (Figure 13). These data strongly support that not only **S1RA** but also compound **12** is a σ_1_ receptor antagonists. Moreover, the effect of **S1RA** and **12** was also reversed by naloxone-methiodide that is a peripheral opioid antagonist. This means that the observed pharmacological effect is due to the peripheral potentiation of opioid antinociception by sigma-1 receptor antagonism.

In further studies, we used two different models of neuropathic pain to test the influence of **12** on pain associated with neuronal tissue damage: the oxaliplatin-induced model and the chronic constriction injury (CCI) model. The mechanism of neuron impairment is different in both, which may relate to the different efficacy of potential treatments. In the oxaliplatin-induced model, neurons are directly affected by this chemotherapeutic agent. That leads to mechanical allodynia resulting from the impaired regulation of ion channels and destruction of mitochondrial DNA, among others.^54^ In CCI, neuropathy impairs peripheral nerve function, and neuronal damage results from a persistent mechanical stimulus and its complications such as vascular and metabolic dysfunctions.

#### 2.8.5 Oxaliplatin (OXPT)-Induced Neuropathy

The neuropathy manifests as a decrease in the pain threshold and can be observed in hours or in days after oxaliplatin administration, which is called the early or late phase of neuropathic pain, respectively. We tested the influence of compound **12** on tactile allodynia using the von Frey method 3 h and 7 days after the induction of neuropathy.^12^

**Figure 14:**
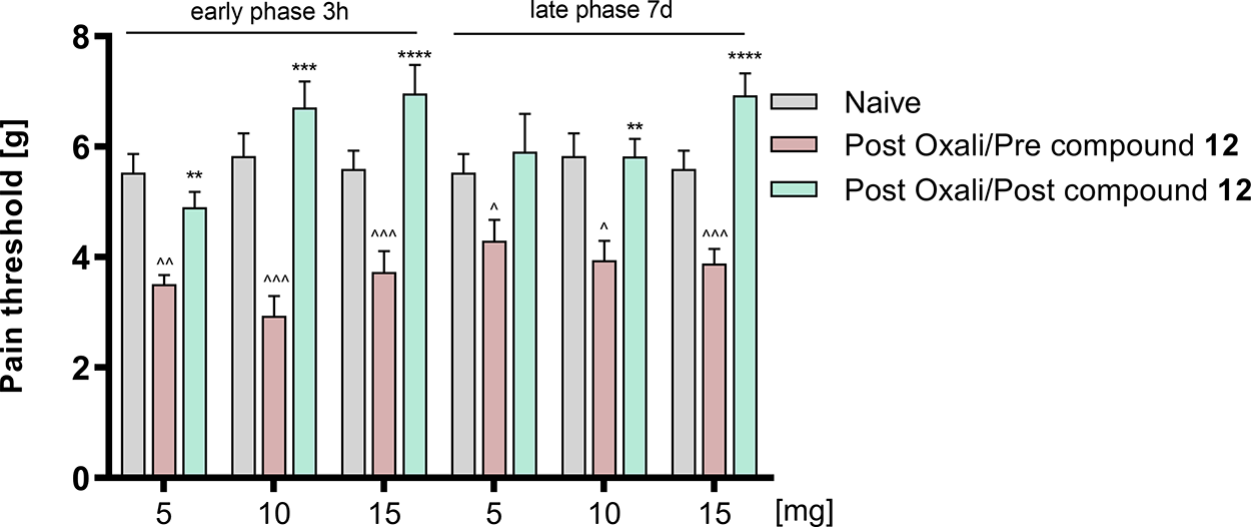
Antiallodynic effects of compound **12** in the tactile allodynia evaluated in the von Frey test in oxaliplatin-induced peripheral neuropathy. Each experimental group consisted of 8–10 animals. **p < 0.01, ***p < 0.001, and ****p < 0.0001 (repeated measures analysis of variance (ANOVA) followed by Dunnett’s *post hoc* test) when the groups were compared to oxaliplatin treatment; ^*p* < 0.05, ^^*p* < 0.01, and ^^^*p* < 0.001 (repeated measures analysis of variance ANOVA followed by *post hoc* Dunnett’s test) when the groups were compared to naive animals.

In the group treated with **12** at the dose of 5 mg/kg (Figure 14), the initial value of 5.53 ± 0.34 (baseline) was decreased to the value of 3.51 ± 0.17 (63.5% of the baseline) and 4.30 ± 0.08 (77.7% of the baseline) in the early and late phase, respectively. The single administration of **12** partially reversed the effect of OXPT in the early phase (88.6% of the baseline; *F*_(2.136, 19.22)_ = 7.66, *p* < 0.01) but not in the late phase. In the group treated with **12** at the dose of 10 mg/kg, the initial value of 5.83 ± 0.41 was decreased to the value of 2.94 ± 0.36 (50.4% of the baseline) and 3.94 ± 0.36 (67.6% of the baseline) in the early and the late phase, respectively. The single administration of **12** reversed the effect of OXPT in both the early phase (115.0% of the baseline) and the late phase (99.8% of the baseline) (*F*_(2.574, 20.59)_ = 17.84, *p* < 0.0001). In the group treated with **12** at the dose of 15 mg/kg, the initial value of 5.60 ± 0.33 was decreased to the value of 3.73 ± 0.38 (66.6% of the baseline) and 3.88 ± 0.27 (69.3% of the baseline) in the early and late phase, respectively. The single administration of **12** completely reversed the effect of OXPT and even elevated the pain threshold above the baseline in both the early phase (124.3% of the baseline) and the late phase (123.7% of the baseline) (*F*_(2.317, 41.71)_ = 21.67, *p* < 0.0001).

These results indicate that compound **12** exhibits potency for relieving chemotherapy-induced neuropathic pain (all the tested doses were significantly active), which confirms its wide range of analgesic activity and encourages further research.

#### 2.8.6 Effects of **12** on Neuropathic Pain Symptoms in CCI-Exposed Mice

**Figure 15:**
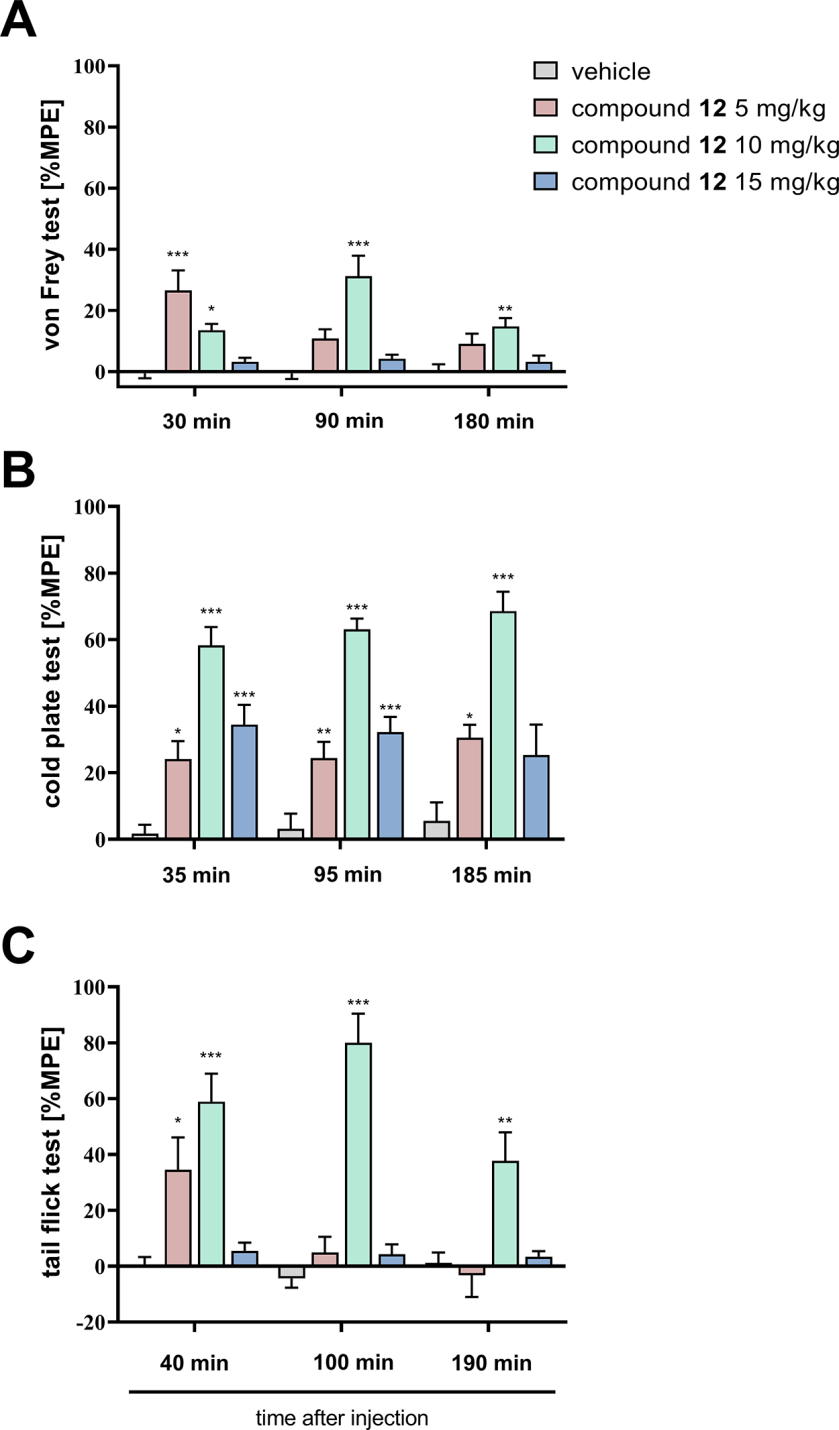
The effects of single i.p. administration of **12** (5, 10 and 15 mg/kg) on mechanical (A, von Frey test) and thermal (B, cold plate test; C, tail flick test) stimulus on day 14 following CCI to the sciatic nerve were evaluated (*n* = 7–8 animals per group). The results are presented as percentages of the maximal possible effect (% MPE; means ± SEM). Intergroup differences were analyzed by one-way ANOVA with Bonferroni’s multiple comparison *post hoc* test. *p < 0.05, **p < 0.01, and ***p < 0.001 vs vehicle-treated group.

Behavioral assessment of **12** was performed 14 days after the sciatic nerve injury. Mice were randomly assigned to four treatment groups: vehicle-treated (i.p.) and **12**-treated (5, 10, and 15 mg/kg, i.p.). Baseline measurements (basal) for all tests were performed before compound injections, and the results are presented as percentages of the maximal possible effect (% MPE). We observed a significant effect of the tested compound in the von Frey test at all tested time points (30 min: *F*_(3, 26)_ = 12.38, *p* < 0,0001; 90 min: *F*_(3, 26)_ = 12.65, *p* < 0.0001; 180 min: *F*_(3, 26)_ = 6.464, *p* = 0.0020). In CCI-exposed mice, **12** exhibited significant analgesic effects 30 min after injection of 5 (*p* < 0.0001) and 10 mg/kg (*p* = 0.0201) in the von Frey test compared with vehicle-treated animals (Figure 15A). However, a single injection of compound **12** only at a dose of 10 mg/kg increased the withdrawal thresholds in the von Frey test at 90 (*p* < 0.0001) and 180 (*p* = 0.0014) min after administration (Figure 15A). We also observed a significant effect of treatment on the contralateral paw 90 min after injection (*F*_(3, 26)_ = 5.741, *p* = 0.0037). Further analysis showed no effects of compound **12** (for doses 5 and 10 mg/kg) on tactile allodynia in the von Frey test at all time points after treatment. However, **12** at dose 15 mg/kg reduced the withdrawal threshold 30 and 180 min after injection, although this effect was not statistically significant at those time points. But 90 min after injection, we observed a significant effect (*p* = 0.0056) (Figure S33). We have also observed a significant effect of treatment on thermal stimuli as measured in a cold plate test at all tested time points (35 min: *F*_(3, 26)_ = 20.77, *p* < 0.0001; 95 min: *F*_(3, 26)_ = 32.42, *p* < 0.0001; 185 min: *F*_(3, 26)_ = 16.11, *p* < 0.0001). Here, analgesic effects of **12** single injection were observed at 35 min for all tested doses as compared with control animals (5 mg/kg: *p* = 0.019; 10 mg/kg: *p* < 0.0001; 15 mg/kg: *p* = 0.0004) (Figure 15B). This antihyperalgesic effect was also observed 95 min after injection (5 mg/kg: *p* = 0.0072; 10 mg/kg: *p* < 0.0001; 15 mg/kg: *p* = 0.0002) (Figure 15B). However, 185 min after treatment, the analgesic effect was significant only for doses 5 (*p* = 0.0482) and 10 mg/kg (*p* < 0.0001). The measurements of spinal responses assessed by the tail flick test also revealed a significant effect of treatments (40 min: *F*_(3, 25)_ = 11.74, *p* < 0.0001; 100 min: *F*_(3, 25)_ = 35.93, *p* < 0.0001; 190 min: *F*_(3, 25)_ = 7.479, *p* = 0.001). The analysis of the tail flick test results (Figure 15C) showed significant analgesic action of **12** at 40 min (for doses 5 (*p* = 0.021) and 10 mg/kg (*p* < 0.0001)) after injection as compared with vehicle-treated mice. At the further time points (100 and 190 min), only the 10 mg/kg dose had significant analgesic effects (*p* < 0.0001 and *p* = 0.0033, respectively) (Figure 15C).

## 3 Conclusions

Comprehensive and rational drug discovery requires a multidisciplinary approach involving many techniques and methods. A deeper understanding of the molecule’s physicochemical properties and its conformational preferences may be very helpful, especially in the case of selective or dual (multi)-target therapeutic agent design.

In our study, we decided to combine chemical, biological, and computational methods to reveal molecular properties responsible for histamine H_3_R and σ_1_R selective or dual-target binding of the studied compounds. Considering the different abilities of piperidine and piperazine derivatives substituted with a 4-pyridyl moiety to form a salt bridge with Glu172 in the σ_1_R binding pocket, which is a crucial interaction for ligand–protein complex stability and thus the high biological activity, we performed an in-depth analysis of their protonation states with three different methods. In the case of piperidine derivatives, the crystal structure confirmed the presence of two protonation centers with higher basic properties of the piperidine vs pyridine nitrogen atom. In contrast, for the piperazine derivatives, we observed a strong basic character of the pyridine nitrogen atom as a consequence of the increased availability of the lone pair electrons. The former is correlated with the source of electrons located at the para position, originated in the piperazine ring’s nitrogen atom with its lone pair of electrons being involved in the π-electron resonance. The observed rigidity of the 4-pyridylpiperazine fragment not only determines the highly basic character of pyridine nitrogen atom but also strongly defines the spatial orientation of π-electrons of the aromatic fragment. This geometrical feature is most likely responsible for the effective ligand–protein recognition and may explain the observed selectivity of the investigated compounds containing 4-pyridylpiperazine fragment for H_3_R. Moreover, the order of protonation of individual nitrogen atoms based on NMR spectroscopy measurements in a pH-controlled environment stays in agreement with the crystallographic data for both piperidines and piperazines. Interestingly, for the piperazine derivative **KSK94**, we observed a transfer of the positive charge from the pyridine nitrogen atom, which was protonated first, to the piperazine nitrogen through appropriate resonance structures. This observation is fully consistent with the crystallographic data and explains the inability of 4-pyridylpiperazine derivatives to form a salt bridge with Glu172 in the σ_1_R binding pocket. This is caused by the low basicity of the second piperazine nitrogen atom, which was initially unprotonated. A series of titrations allowed determining p*K*_a_ values of individual nitrogen atoms and recognizing piperidine derivative **KSK68** as a basic ligand with p*K*_a1_ = 4.9 (pyridine N) and p*K*_a2_ = 8.4 (piperidine N). In turn, the **KSK94** compound belongs to ligands with an acidic center located on the piperazine nitrogen atom to which the pyridine ring is directly attached (p*K*_a2_ = 1). The protonation states of the compounds were also assessed *in silico*, and the obtained results were consistent with the experimental data.

In the next step, we designed and synthesized a series of 16 novel compounds, mainly piperidine derivatives, and determined their affinity at H_3_R, σ_1_R, and σ_2_R. We confirmed the previously described phenomenon and identified a second pair of similar compounds, differing only in the 4-pyridylpiperazine/piperidine core with dual H_3_/σ_1_R activity of the piperidine derivative **12** and no affinity toward σ_1_R of its piperazine analogue **KSK94**. To further investigate the impact of structural differences between these moieties on their molecular mode of action, the binding to σ_1_R and H_3_R was evaluated using molecular modeling techniques and molecular dynamics simulations. Both the free binding energies and the docking scores of the most abundant protonation states correlated with the *in vitro* activity of the tested ligands.

As the lead structures for further evaluation, we selected compounds **3** and **7** with the highest σ_2_/σ_1_ selectivity factor (13.2 and 24.2, respectively) and ligand **12** with the same high σ_2_R activity as for major biological targets. It is well known that sigma receptors modulate nociception, offering a potential therapeutic target for pain management, but relatively little is known about the role of σ_2_R in the pathomechanism of this condition. Interestingly, recent studies with the highly σ_2_R-selective compound CM-398 showed that it ameliorates inflammatory and chronic neuropathic pain in established mouse models, suggesting possible mediation of nociception by σ2R.^33^ Therefore, it seems reasonable to test triple-targeting H_3_/σ_1_/σ_2_R ligand **12** alongside the dual-acting compounds **3** and **7**.

All lead structures had no affinity at other histamine receptor subtypes and were characterized as potent H_3_R antagonists in the cAMP accumulation assay. Moreover, we checked their ability to penetrate lipid membranes by parallel artificial membrane permeability assay. All tested molecules can be considered substances with high passive transport through biological membranes. Taking into account an intracellular localization of σ_1_R, the satisfactory level of lipid membranes penetration and thus the ability of studied compounds to reach this biological target seem to be an important feature of dual H_3_/σ_1_R ligands. Furthermore, we also tested the serum stability of compound **12**, and the obtained data clearly indicate that it can be considered as a moderately stable ligand, which is relatively rapidly degraded by serum enzymes. This is associated with the presence of a hydrolysis-susceptible nitrile group within its structure. On the other hand, this moiety was one of the key structural element responsible for the pharmacological activity of **12**. Thus, the identification of other groups in this region, less sensitive to serum enzymes activity, will be the subject of our further studies. Knowledge of the permeability profile and stability in plasma of tested ligand is a very preliminary step in predicting its exposure required for *in vivo* efficacy.

Finally, we tested the efficacy of compounds **3**, **7**, and **12** in animal models of pain. We started by testing the safety pharmacology and checked their influence on spontaneous locomotor activity and motor coordination in the rotarod test. Compounds **3** and **7** showed high and significant sedative effects and significantly impaired motor coordination in mice at the administrated doses. In contrast, the obtained results showed that **12** is characterized by a better safety profile than the other two tested compounds; hence, we chose this ligand for further analysis of its analgesic activity. The high potency of **12** in both formalin- and capsaicin-induced pain shows that this compound has the potential to attenuate neurogenic pain regardless of the mechanism of its induction. We then tested the effect of **12** on antinociception induced by the opioid agonist loperamide. Administration of compound **12** enhanced the effect of loperamide, which was markedly reversed by co-administration of the σ_1_R agonist PRE-084. These data strongly support that **12** is a potent σ_1_R antagonist. Finally, we used two different models of neuropathic pain to test the influence of **12** on pain associated with neuronal tissue damage. The obtained results indicate that compound **12** has the potential to alleviate both chemotherapy-induced neuropathic pain and pain resulting from sciatic nerve damage. This confirms its broad spectrum of analgesic activity, which may be related to the dual H_3_/σ_1_R modulation as well as the additional interaction with σ_2_R. Determining the exact mechanism of action of compound **12** will be the subject of our further studies.

## 4 Experimental Protocols

### 4.1 Chemistry

All reagents were purchased from commercial suppliers and were used without further purification. Melting points (m.p.) were determined on a MEL-TEMP melting point apparatus II (LD Inc., USA) and are uncorrected. HPLC analysis were performed on an Agilent 1290 UHPLC system coupled with an Agilent QTOF 6545 mass spectrometer (a Phenomenex Kinetex 1.7 μm EVO C18 (100 Å, 50 × 2.1 mm) reversed-phase column was used). Mass spectra (LC/MS) were carried out on a system consisting of a Waters Acquity UPLC coupled to a Waters TQD mass spectrometer. Retention times (*t*_R_) are given in minutes. High-resolution mass spectrometry (HRMS) was performed on Thermo Scientific Orbitrap Exploris 240 mass spectrometer. ^1^H NMR spectra were recorded on a Varian Mercury 300 MHz PFG spectrometer in DMSO-*d*_6_. Chemical shifts were expressed in parts per million (ppm) using the solvent signal as an internal standard. Data are reported in the following order: multiplicity (s, singlet; d, doublet; dd, double of doublets; t, triplet; tt, triplet of triplets; quin, quintet; qd, quartet of doublets; m, multiplet; br, broad), approximate coupling constants *J* expressed in hertz (Hz), and number of protons. ^13^C NMR spectra were recorded on a Varian-Mercury-VX 300 MHz PFG or Bruker 400 MHz spectrometer at 75 MHz in DMSO-*d*_6_. Elemental analyses (C, H, N) were performed on a Vario El III elemental analyzer (Hanau, Germany). For CC purification (column chromatography using silica gel 60 (0.063–0.20 mm); Merck), the following solvent systems were used: I: petroleum ether/EtOAc (9:1); II: CH_2_Cl_2_/MeOH (9:1). Chemical compounds’ names were generated using Chem Draw Professional 18.0.0.231 (4029).

All final compounds are >95% pure by HPLC analysis.

#### 4.1.1 General Synthetic Procedure for Compounds **a**–**i**

All compounds were obtained using methods described previously.^19,21,22,24,25^ To a solution of freshly prepared sodium 1-propanolate (0.1 mol, 100 mL), proper substituted phenols (0.1 mol) were added and stirred in room temperature for 5 min. α,ω-Dibromoalkanes (0.2 mol) were then added dropwise in the time of 1 h. The reaction mixture was stirred in 60 °C for 3 h and then refluxed for another 3 h. After cooling down to RT, the mixture was filtrated and evaporated. To a rough product, 100 mL of 10% NaOH was added and left overnight in the cold. To a resulting white oil, CH_2_Cl_2_ was added and mixed, and layers were then separated. The organic layer was dried over sodium sulfate, filtered, and evaporated. The rough product was used for further reactions after purification.

##### 4.1.1.1 4-(3-Bromopropoxy)-1,1′-biphenyl (**a**)

Compound previously described in the literature.^21^ Obtained 22.13 g of raw product, purified by CC (I), yield 76%. CAS113795-28-1.

##### 4.1.1.2 4′-(3-Bromopropoxy)-[1,1′-biphenyl]-4-carbonitrile (**b**)

Compound previously described in the literature.^22^ Obtained 17.71 g of raw product, purified by CC (I), yield 56%. CAS134880-32-3.

##### 4.1.1.3 1-(3-Bromopropoxy)-4-phenoxybenzene (**c**)

Compound previously described in the literature.^22^ Obtained 16.59 g of raw product, purified by CC (I). yield 54%. CAS63457-51-2.

##### 4.1.1.4 4-(3-Bromopropoxy)-*N*-phenylaniline (**d**)

Compound previously described in the literature.^22^ Obtained 15.92 g of raw product, purified by CC (I), yield 52%.

##### 4.1.1.5 1-Benzyl-4-(3-bromopropoxy)benzene (**e**)

Compound previously described in the literature.^22^ Obtained 17.40 g of raw product, purified by CC (I), yield 57%. CAS60859-24-7.

##### 4.1.1.6 4-(4-Bromobutoxy)-1,1′-biphenyl (**f**)

Compound previously described in the literature.^55^ Obtained 12.51 g of raw product, purified by CC (I), yield 41%. CAS53669-78-6.

##### 4.1.1.7 4-((5-Bromopentyl)oxy)-1,1′-biphenyl (**g**)

Compound previously described in the literature.^24^ Obtained 7.66 g of raw product, purified by CC (I), yield 24%. CAS52273-19-5.

##### 4.1.1.8 4-((6-Bromohexyl)oxy)-1,1′-biphenyl (**h**)

Compound previously described in the literature.^24^ Obtained 8.33 g of raw product, purified by CC (I), yield 25%. CAS158136-46-0.

##### 4.1.1.9 1-(4-Bromobutoxy)-4-(*tert*-butyl)benzene (**I**)

Compound previously described in the literature.^19^ Obtained 19.68 g of raw product, purified by CC (I), yield 69%. CAS53669-73-1.

#### 4.1.2 General Synthetic Procedure for Compounds **1**–**16**

All compounds were obtained using methods described previously.^19,21,22,24,25^ To a suspension of potassium carbonate (8.5 mmol) and a catalytic amount of potassium iodide in water (10 mL), a mixture of proper amine (5 mmol) and compound **a**–**i** (5 mmol) in ethanol (50 mL) was added as previously described.^19,21,22,24,25^ The mixture was then refluxed for 8–12 h (TLC controlled). After cooling down to room temperature, reaction mixture was filtrated, evaporated, and purified. To the resulting oil, 100 mL of CH_2_Cl_2_ was added and washed with 0.5% HCl solution, 0.5% NaOH solution, and water. After drying over anhydrous Na_2_SO_4_ and evaporation of the organic layer, the product was further purified using CC (II). Compounds **5**, **10**, **11**, and **16** were obtained as in previous studies and used in the biological assays with high purity estimated using LC/MS; details of their synthesis and analyses are described elsewhere.^19,24,25^

##### 4.1.2.1 1-(3-([1,1′-Biphenyl]-4-yloxy)propyl)-3-methylpiperidine Oxalate (**1**)

White solid, yield 30%, m.p. 169–172 °C, C_21_H_27_NO·C_2_H_2_O_4_ (MW = 399.49). RP-HPLC: 99.67% (*t*_R_ = 2.890, *k’* = 14.21). ^1^H NMR (500 MHz, DMSO-*d*_6_) δ ppm: 7.57 (dd, *J* = 2.00, 8.31 Hz, 4H), 7.39 (t, *J* = 7.73 Hz, 2H), 7.24–7.31 (m, 1H), 6.99 (d, *J* = 8.59 Hz, 2H), 4.04 (t, *J* = 6.01 Hz, 2H), 3.28–3.42 (m, 2H), 3.10 (t, *J* = 7.59 Hz, 2H), 2.71 (t, *J* = 10.74 Hz, 1H), 2.43 (br. s., 1H), 2.11 (quin, *J* = 6.80 Hz, 2H), 1.79–1.90 (m, 1H), 1.62–1.78 (m, 3H), 0.95–1.09 (m, 1H), 0.86 (d, *J* = 6.59 Hz, 3H). ^13^C NMR (126 MHz, DMSO-*d*_6_) δ ppm: 165.2, 158.4, 140.3, 133.3, 129.4, 128.3, 127.3, 126.7, 115.5, 65.7, 58.1, 54.0, 54.0, 52.2, 30.6, 29.1, 24.1, 22.8, 22.8, 19.1. LC–MS: purity 100.00%, *t*_R_ = 5.53, (ESI) *m/z* [M + H]^+^ 310.22. Anal. calcd. for C_23_H_29_NO_5_: C, 69.15; H, 7.32; N, 3.51%. Found: C, 68.94; H, 7.08; N, 3.46%.

##### 4.1.2.2 1-(3-([1,1′-Biphenyl]-4-yloxy)propyl)-4-methylpiperidine Oxalate (**2**)

White solid, yield 38%, m.p. 222–224 °C, C_21_H_27_NO·C_2_H_2_O_4_ (MW = 399.49). RP-HPLC: 97.96% (*t*_R_ = 2.888, *k’* = 14.20). ^1^H NMR (500 MHz, DMSO-*d*_6_) δ ppm: 7.57 (d, *J* = 8.59 Hz, 4H), 7.39 (t, *J* = 7.73 Hz, 2H), 7.24–7.30 (m, 1H), 6.99 (d, *J* = 8.59 Hz, 2H), 4.05 (t, *J* = 6.01 Hz, 2H), 3.37 (d, *J* = 10.60 Hz, 2H), 3.06–3.15 (m, 2H), 2.83 (br. s., 2H), 2.02–2.15 (m, 2H), 1.74 (d, *J* = 13.46 Hz, 2H), 1.57 (br. s., 1H), 1.27–1.40 (m, 2H), 0.89 (d, *J* = 6.30 Hz, 3H). ^13^C NMR (126 MHz, DMSO-*d*_6_) δ ppm: 140.28, 133.34, 129.42, 128.32, 126.71, 115.48, 100.00, 65.60, 24.28. LC–MS: purity 97.76%, *t*_R_ = 5.47, (ESI) *m/z* [M + H]^+^ 310.22. Anal. calcd. for C_23_H_29_NO_5_: C, 69.15; H, 7.32; N, 3.51%. Found: C, 68.82; H, 7.53; N, 3.38%.

##### 4.1.2.3 1-(3-([1,1′-Biphenyl]-4-yloxy)propyl)azepane Oxalate (**3**)

White solid, yield 38%, m.p. 187–190 °C, C_21_H_27_NO·C_2_H_2_O_4_ (MW = 399.49). RP-HPLC: 96.06% (*t*_R_ = 2.875, *k’* = 14.13). ^1^H NMR (500 MHz, DMSO-*d*_6_) δ ppm: 7.57 (d, *J* = 7.73 Hz, 4H), 7.39 (t, *J* = 7.30 Hz, 2H), 7.27 (t, *J* = 7.16 Hz, 1H), 7.00 (d, *J* = 8.31 Hz, 2H), 4.05 (t, *J* = 5.58 Hz, 2H), 2.93–3.40 (m, 6H), 2.10 (br. s., 2H), 1.76 (br. s., 4H), 1.40–1.63 (m, 4H). ^13^C NMR (126 MHz, DMSO-*d*_6_) δ ppm: 165.1, 158.4, 140.3, 133.3, 129.4, 128.3, 127.3, 126.7, 115.5, 65.6, 54.3, 54.2, 26.5, 24.5, 23.7. LC–MS: purity 96.08%, *t*_R_ = 5.51, (ESI) *m/z* [M + H]^+^ 310.22. Anal. calcd. for C_23_H_29_NO_5_: C, 69.15; H, 7.32; N, 3.51%. Found: C, 68.71; H, 7.08; N, 3.38%.

##### 4.1.2.4 1-(3-([1,1′-Biphenyl]-4-yloxy)propyl)pyrrolidine Oxalate (**4**)

White solid, yield 42%, m.p. 204–206 °C, C_19_H_23_NO·C_2_H_2_O_4_ (MW = 371.43). RP-HPLC: 96.58% (*t*_R_ = 2.768, *k’* = 13.57). ^1^H NMR (500 MHz, DMSO-*d*_6_) δ ppm: 7.57 (d, *J* = 8.31 Hz, 4H), 7.39 (t, *J* = 7.59 Hz, 2H), 7.23–7.31 (m, 1H), 6.99 (d, *J* = 8.59 Hz, 2H), 4.05 (t, *J* = 5.87 Hz, 2H), 3.01–3.42 (m, 6H), 2.03–2.13 (m, 2H), 1.89 (br. s., 4H). ^13^C NMR (126 MHz, DMSO-*d*_6_) δ ppm: 165.0, 158.4, 140.3, 133.3, 129.4, 128.3, 127.3, 126.7, 115.6, 115.5, 65.4, 53.7, 51.9, 25.9, 23.2. LC–MS: purity 100.00%, *t*_R_ = 5.10 (ESI) *m/z* [M + H]^+^ 282.137. HRMS (MALDI): *m/z* [M + H]^+^ 282.1856.

##### 4.1.2.5 4′-(3-(Piperidin-1-yl)propoxy)-[1,1′-biphenyl]-4-carbonitrile Oxalate (**5**)

Compound previously described in the literature.^25^ White solid, yield 37%. RP-HPLC: 95.63% (*t*_R_ = 2.731, *k’* = 13.37). LC–MS: purity 93.82%, *t*_R_ = 4.64, (ESI) *m/z* [M + H]^+^ 321.26. HRMS (MALDI): *m/z* [M + H]^+^ 321.1966.

##### 4.1.2.6 1-(3-(4-Phenoxyphenoxy)propyl)piperidine Oxalate (**6**)

White solid, yield 46%, m.p. 137–140 °C, C_20_H_25_NO_2_·C_2_H_2_O_4_ (MW = 401.46). RP-HPLC: 100.00% (*t*_R_ = 2.808, *k’* = 13.78). ^1^H NMR (500 MHz, DMSO-*d*_6_) δ ppm: 7.26–7.36 (m, 2H), 7.03 (t, *J* = 7.30 Hz, 1H), 6.90–6.98 (m, 4H), 6.87 (d, *J* = 7.73 Hz, 2H), 3.98 (t, *J* = 6.01 Hz, 2H), 2.79–3.36 (m, 6H), 2.00–2.16 (m, 2H), 1.60–1.77 (m, 4H), 1.49 (br. s., 2H). ^13^C NMR (126 MHz, DMSO-*d*_6_) δ ppm: 165.3, 158.5, 155.2, 150.1, 130.4, 123.2, 121.2, 117.9, 116.3, 66.0, 54.0, 52.6, 24.1, 23.1, 22.0. LC–MS: purity 97.09%, *t*_R_ = 5.19, (ESI) *m/z* [M + H]^+^ 312.41. Anal. calcd. for C_22_H_27_NO_6_: C, 65.82; H, 6.78; N, 3.49%. Found: C, 65.59; H, 6.61; N, 3.39%.

##### 4.1.2.7 *N*-Phenyl-4-(3-(piperidin-1-yl)propoxy)aniline Oxalate (**7**)

White solid, yield 34%, m.p. 130–133 °C, C_20_H_26_N_2_O·C_2_H_2_O_4_ (MW = 400.48). RP-HPLC: 99.41% (*t*_R_ = 2.726, *k’* = 13.35). ^1^H NMR (400 MHz, DMSO-*d*_6_) δ ppm: 7.87 (br. s., 1H), 7.12–7.22 (m, 2H), 7.04 (d, *J* = 9.00 Hz, 2H), 6.83–6.96 (m, 4H), 6.72 (t, *J* = 7.24 Hz, 1H), 3.98 (t, *J* = 5.87 Hz, 2H), 2.91–3.32 (m, 6H), 2.04–2.20 (m, 2H), 1.63–1.84 (m, 4H), 1.53 (br. s., 2H). ^13^C NMR (101 MHz, DMSO-*d*_6_) δ ppm: 165.1, 153.1, 145.4, 136.9, 129.6, 120.6, 118.8, 115.8, 115.4, 65.8, 54.1, 52.6, 24.1, 23.2, 22.0. LC–MS: purity 100%, *t*_R_ = 4.88, (ESI) *m/z* [M + H]^+^ 311.41. Anal. calcd. for C_22_H_28_N_2_O_5_: C, 65.98; H, 7.05; N, 7.00%. Found: C, 66.25; H, 6.89; N, 7.00%.

##### 4.1.2.8 1-(3-(4-Benzylphenoxy)propyl)piperidine Oxalate (**8**)

White solid, yield 39%, m.p. 152–155 °C, C_21_H_27_NO·C_2_H_2_O_4_ (MW = 399.49). RP-HPLC: 98.02% (*t*_R_ = 2.866, *k’* = 14.08). ^1^H NMR (500 MHz, DMSO-*d*_6_) δ ppm: 7.19–7.29 (m, 2H), 7.03–7.18 (m, 5H), 6.81 (d, *J* = 8.59 Hz, 2H), 3.94 (t, *J* = 6.01 Hz, 2H), 3.82 (s, 2H), 2.76–3.43 (m, 6H), 1.98–2.11 (m, 2H), 1.62–1.75 (m, 4H), 1.47 (br. s., 2H). ^13^C NMR (126 MHz, DMSO-*d*_6_) δ ppm: 165.3, 157.1, 142.3, 134.1, 130.2, 129.1, 128.9, 126.4, 115.0, 65.5, 54.0, 52.6, 24.0, 23.1, 22.0. LC–MS: purity 100.00%, *t*_R_ = 5.35, (ESI) *m/z* [M + H]^+^ 310.47. Anal. calcd. for C_23_H_29_NO_5_: C, 69.15; H, 7.32; N, 3.51%. Found: C, 68.46; H, 7.53; N, 3.36%.

##### 4.1.2.9 1-(4-([1,1′-Biphenyl]-4-yloxy)butyl)piperidine Oxalate (**9**)

White solid, yield 24%, m.p. 182–185 °C, C_21_H_27_NO·C_2_H_2_O_4_ (MW = 399.49). RP-HPLC: 97.54% (*t*_R_ = 2.863, *k’* = 14.07). ^1^H NMR (500 MHz, DMSO-*d*_6_) δ ppm: 7.56 (dd, *J* = 5.58, 7.59 Hz, 4H), 7.39 (t, *J* = 7.73 Hz, 2H), 7.18–7.31 (m, 1H), 6.88–7.06 (m, 2H), 4.00 (t, *J* = 5.87 Hz, 2H), 2.72–3.49 (m, 6H), 1.58–1.86 (m, 8H), 1.29–1.58 (m, 2H). ^13^C NMR (126 MHz, DMSO-*d*_6_) δ ppm: 165.1, 165.1, 165.0, 158.7, 140.3, 133.1, 129.4, 128.3, 127.3, 126.7, 115.5, 67.4, 56.1, 52.5, 26.5, 23.1, 22.0, 20.8. LC–MS: purity 96.17%, *t*_R_ = 5.46, (ESI) *m/z* [M + H]^+^ 310.22. Anal. calcd. for C_23_H_29_NO_5_: C, 69.15; H, 7.32; N, 3.51%. Found: C, 68.78; H, 7.47; N, 3.31%.

##### 4.1.2.10 1-(5-([1,1′-Biphenyl]-4-yloxy)pentyl)piperidine Oxalate (**10**)

Compound previously described in the literature.^24^ White solid, yield 66%. RP-HPLC: 98.88% (*t*_R_ = 2.934, *k’* = 14.44). LC–MS: purity 99.60%, *t*_R_ = 5.81, (ESI) *m/z* [M + H]^+^ 324.44.

##### 4.1.2.11 1-(6-([1,1′-Biphenyl]-4-yloxy)hexyl)piperidine Oxalate (**11**)

Compound previously described in the literature.^24^ White solid, yield 60%. RP-HPLC: 100.00% (*t*_R_ = 3.013, *k’* = 14.86). LC–MS: purity 100.00%, *t*_R_ = 6.18, (ESI) *m/z* [M + H]^+^ 338.47.

##### 4.1.2.12 4′-(3-(4-(Pyridin-4-yl)piperidin-1-yl)propoxy)-[1,1′-biphenyl]-4-carbonitrile Oxalate (**12**)

White solid, yield 32%, m.p. 157–160 °C, C_26_H_27_N_3_O·C_2_H_2_O_4_ (MW = 487.56). RP-HPLC: 100.00% (*t*_R_ = 2.431, *k’* = 11.79). ^1^H NMR (500 MHz, DMSO-*d*_6_) δ ppm: 8.40–8.43 (m, 2H), 7.79–7.86 (m, 4H), 7.65–7.69 (m, 2H), 7.22–7.24 (m, 2H), 7.01–7.04 (m, 2H), 4.04 (t, *J* = 6.44 Hz, 2H), 2.95 (d, *J* = 11.46 Hz, 2H), 2.48–2.52 (m, 1H), 2.43 (t, *J* = 7.16 Hz, 2H), 1.93–2.01 (m, 2H), 1.88 (quin, *J* = 6.73 Hz, 2H), 1.69–1.76 (m, 2H), 1.55–1.65 (m, 2H). ^13^C NMR (126 MHz, DMSO-*d*_6_) δ ppm: 159.9, 155.3, 150.1, 144.8, 133.3, 128.9, 127.4, 122.9, 115.7, 109.6, 66.6, 55.2, 54.1, 32.6, 26.9. LC–MS: purity 100.00%, *t*_R_ = 3.54, (ESI) *m/z* [M + H]^+^ 398.12. HRMS (MALDI): *m/z* [M + H]^+^ 398.2230.

##### 4.1.2.13 4-(1-(3-(4-Phenoxyphenoxy)propyl)piperidin-4-yl)pyridine Oxalate (**13**)

White solid, yield 34%, m.p. 143–146 °C, C_25_H_28_N_2_O_2_·C_2_H_2_O_4_ (MW = 478.55). RP-HPLC: 100.00% (*t*_R_ = 2.492, *k’* = 12.12). ^1^H NMR (500 MHz, DMSO-*d*_6_) δ ppm: 8.41–8.43 (m, 2H), 7.28–7.32 (m, 2H), 7.22–7.25 (m, 2H), 7.02 (tt, *J* = 7.41, 1.04 Hz, 1H), 6.90–6.96 (m, 4H), 6.86–6.89 (m, 2H), 3.96 (t, *J* = 6.30 Hz, 2H), 2.95 (d, *J* = 11.46 Hz, 2H), 2.48–2.52 (m, 1H), 2.42 (t, *J* = 7.30 Hz, 2H), 1.93–2.00 (m, 2H), 1.82–1.89 (m, 2H), 1.72 (d, *J* = 12.60 Hz, 2H), 1.60 (qd, *J* = 12.36, 3.58 Hz, 2H). ^13^C NMR (126 MHz, DMSO-*d*_6_) δ ppm: 158.6, 155.6, 149.8, 130.4, 122.9, 117.8, 116.2, 66.8, 55.2, 54.1, 32.6, 27.0. LC–MS: purity 100.00%, *t*_R_ = 3.94, (ESI) *m/z* [M + H]^+^ 389.23. HRMS (MALDI): *m/z* [M + H]^+^ 389.2227.

##### 4.1.2.14 *N*-Phenyl-4-(3-(4-(pyridin-4-yl)piperidin-1-yl)propoxy)aniline Oxalate (**14**)

White solid, yield 38%, m.p. 182–185 °C, C_25_H_29_N_3_O·C_2_H_2_O_4_ (MW = 477.56). RP-HPLC: 100.00% (*t*_R_ = 2.400, *k’* = 11.63). ^1^H NMR (500 MHz, DMSO-*d*_6_) δ ppm: 8.41–8.43 (m, 2H), 7.80 (s, 1H), 7.22–7.24 (m, 2H), 7.09–7.13 (m, 2H), 6.97–7.01 (m, 2H), 6.86–6.89 (m, 2H), 6.80–6.84 (m, 2H), 6.66 (tt, *J* = 7.30, 1.15 Hz, 1H), 3.92 (t, *J* = 6.30 Hz, 2H), 2.97 (d, *J* = 10.02 Hz, 2H), 2.48–2.54 (m, 2H), 2.00 (br. s., 2H), 1.84 (quin, *J* = 6.73 Hz, 2H), 1.73 (d, *J* = 12.03 Hz, 2H), 1.61 (qd, *J* = 12.32, 3.44 Hz, 2H). ^13^C NMR (126 MHz, DMSO-*d*_6_) δ ppm: 155.3, 153.6, 150.1, 145.6, 136.6, 129.6, 122.9, 120.8, 118.8, 115.7, 115.3, 66.6, 55.3, 54.0, 32.5, 26.9. LC–MS: purity 100.00%, *t*_R_ = 3.62, (ESI) *m/z* [M + H]^+^ 388. HRMS (MALDI): *m/z* [M + H]^+^ 388.2387.

##### 4.1.2.15 4-(1-(3-(4-Benzylphenoxy)propyl)piperidin-4-yl)pyridine Oxalate (**15**)

White solid, yield 41%, m.p. 135–138 °C, C_26_H_30_N_2_O·C_2_H_2_O_4_ (MW = 476.57). RP-HPLC: 98.95% (*t*_R_ = 2.538, *k’* = 12.36). ^1^H NMR (500 MHz, DMSO-*d*_6_) δ ppm: 8.40–8.43 (m, 2H), 7.21–7.25 (m, 4H), 7.06–7.17 (m, 5H), 6.78–6.82 (m, 2H), 3.92 (t, *J* = 6.44 Hz, 2H), 3.82 (s, 2H), 3.29 (br. s., 1H), 2.94 (d, *J* = 10.88 Hz, 2H), 2.37–2.44 (m, 2H), 1.90–2.04 (m, 2H), 1.83 (quin, *J* = 6.80 Hz, 2H), 1.71 (d, *J* = 12.03 Hz, 2H), 1.59 (qd, *J* = 12.32, 3.72 Hz, 2H). ^13^C NMR (126 MHz, DMSO-*d*_6_) δ ppm: 157.5, 155.3, 150.1, 142.3, 133.7, 130.2, 128.9, 122.9, 114.9, 66.3, 55.5, 55.2, 54.0, 32.6, 26.9. LC–MS: purity 100.00%, *t*_R_ = 4.17, (ESI) *m/z* [M + H]^+^ 387.22. HRMS (MALDI): *m/z* [M + H]^+^ 387.2434.

##### 4.1.2.16 2-(4-(4-(4-(*tert*-Butyl)phenoxy)butyl)piperazin-1-yl)pyrazine Oxalate (**16**)

Compound previously described in the literature.^19^ White solid, yield 61%. RP-HPLC: 100.00% (*t*_R_ = 2.874, *k’* = 14.13). LC–MS: purity 98.64%, *t*_R_ = 5.38, (ESI) *m/z* [M + H]^+^ 370.51.

### 4.2 pH-Dependent Crystallization and Crystal Structure Determination

Single crystals were obtained by crystallization in two sets of samples, oxalate salts of compounds **KSK67** and **KSK68**, followed by free base samples of **KSK67_fb**, **KSK68_fb**, **KSK94**_fb, and **12_fb**. The **KSK67** and **KSK68** oxalate salts were initially crystallized by slow evaporation of the solvent at ambient conditions from propan-2-ol solution with few drops of water. This procedure resulted in obtaining crystals suitable for the X-ray experiment. In the next step, the attempts to crystallize the mentioned samples in three batches were conducted: (1) from the water solution (solution pH: 4.5–5) and (2) with the addition of 1 M HCl (the solution pH: 3.5) and (3) 1 M NaOH (solution pH: 7–7.5). **KSK68** is more soluble in H_2_O than **KSK67**. The solubility of both samples increases in acidic conditions, whereas the addition of alkali leads to rapid precipitation. The controlled, dropwise addition of NaOH allowed obtaining a clear solution at equilibrium. The described crystallization procedures of the oxalate salts resulted in good quality crystals only for **KSK68** with addition of NaOH (named as **KSK68_OH**). The free base samples of **KSK67_fb**, **KSK68_fb**, **KSK94_fb**, and **12_fb** are not highly soluble in water. To solve all samples, an addition of organic solvents mixture was required: propan-2-ol and acetone (2:1, v:v; for **KSK67_fb** and **KSK68_fb**) or propan-2-ol, acetone, and THF (2:5:1, v:v:v; for **KSK94_fb** and **12_fb**). The final pH of solutions was in the range 6.5–7. All samples showed very good solubility in acidic conditions (pH 3.5) and were insoluble after adding the NaOH solution. The above described crystallization procedures of free bases resulted in good quality crystals only for the water/organic solutions. X-ray single-crystal diffraction data sets were collected using an XtaLAB Synergy-S four-circle diffractometer with a mirror monochromator and a microfocus Cu Kα radiation source (λ = 1.5418 Å). The CryoStream cryostat system was used to allow low-temperature experiments performed at 100(2) K. The obtained data sets were processed with the CrysAlisPro software (Rigaku-Oxford Diffraction; CrysAlisPro Oxford Diffraction Ltd., Abingdon, England, V 1. 171. 36. 2 (release 27-06-2012 CN) 2006). The phase problem was solved with direct methods using SHELXT.^56^ Parameters of the obtained model were refined by full-matrix least-squares on *F*^2^ using SHELXL-2014/6.^57^ Calculations were performed using the WinGX integrated system (ver. 2014.1).^58^ Figures were prepared with the Mercury 4.0 software.^59^ All nonhydrogen atoms were refined anisotropically. All hydrogen atoms attached to carbon atoms were positioned with the idealized geometry and refined using the riding model with the isotropic displacement parameter U_iso_[H] = 1.2 U_eq_[C]. The hydrogen atoms attached to nitrogens (N1 and/or N10) as a result of protonation of the basic center were located on the Fourier difference map. Hydrogen atoms of water molecules present in the crystal lattice were, in most cases, defined based on the Fourier difference map and were refined using the riding model with the isotropic displacement parameter U_iso_[H] = 1.5 U_eq_[O]. In many cases, restraints on distances and angles (DFIX and DANG commands, respectively) were required to maintain relatively good water molecule geometry. Only for structure **KSK67**, where interpretation of the Fourier difference map did not allow water molecules’ hydrogen atoms location, was the CALC-OH predictive algorithm^60^ available in the WinGX suite^58^ applied. In structures **KSK67**, **KSK68_fb**, and **KSK94_fb**, a positional disorder within the molecule was observed. This disorder was modeled based on the Fourier difference map inspection, and the site occupancies were determined during the refinement procedure. Crystal data and refinement results are shown in Tables S1 (for oxalate salts) and S2 (for free bases) in the Supporting Information file. The asymmetric units of all obtained crystal structures, presenting atom labeling as well as the positional disordered observed for tested compounds, are shown in Figures S1–S7 in the SI file. Crystallographic data have been deposited with the Cambridge Crystallographic Data Centre (CCDC) as supplementary publication nos. CCDC2245077 (**KSK67**), CCDC2245079 (**KSK68**), CCDC2245081 (**KSK68_OH**), CCDC2245080 (**KSK67_fb**), CCDC2245078 (**KSK68_fb**), CCDC2245082 (**KSK94_fb**), and CCDC2245083 (**12_fb**). Copies of the data can be obtained, free of charge, on application to CCDC (e-mail: deposit@ccdc.cam.ac.uk).

### 4.3 Potentiometric Titration

The potentiometric microtitration was performed in a thermostated 20 mL cell using a CerkoLab microtitration unit fitted with a pH electrode (Hydromet ERH-13-6). The electrode was calibrated with the use of buffer solutions: pH = 4.00, 7.00, and 10.00. Titrant T (0.1 M KOH) was standardized according to the general analytical procedure and protected from carbon dioxide. Double distilled water with a conductivity of about 0.18 mS/cm was used to prepare all aqueous solutions. DMSO (HPLC grade, 99.5%) was supplied by Sigma Aldrich. The concentration of the titrant solution (D) was 0.0025–0.005 M. The solubilization procedure was follows: The appropriate amount of KSK compound was dissolved in 1 mL 0.1 M HCl and 1 mL DMSO to prepare a 25 mL stock solution. The developed solubilization system can be recommended for poorly soluble derivatives. The analytical procedure was a follows: volume *V*_0_ = 4.5 mL of titrant D was titrated with 0.1 M titrant T using the CerkoLab System equipped with a 5 mL syringe pump. Titrant T was added to titrant D in 0.005 mL increments with 10 s intervals. p*K*_a_ values were calculated from the experimental data points {(*Vj*,pH*j*)/*j* = 1, ..., *N*} according to the Kostrowicki and Liwo algorithm.^29,30^

### 4.4 NMR Spectroscopy Measurements in the pH-Controlled Environment

NMR spectra were measured on a JEOL JNM-ECZR 600 MHz spectrometer equipped with a Royal HFX probe. All measurements were carried out at stable 303 K. DMSO-*d*_6_ of purity 99.8% was purchased from Eurisotop and stored over A3 molecular sieves for a few days. Proton spectra were collected from 16 scans and 32,768 points with an acquisition time of 2.42 s and relaxation delay of 5 s. A standard sequence with one 45° pulse on the proton channel before acquisition was used. Titrations were conducted using trifluoromethylsulfonic (triflic) acid purchased from Sigma-Aldrich with a purity of over 99%. A solution of 0.5 M concentration in DMSO-*d*_6_ was made and further used. Analysis of the results was carried out using the JEOL JASON software. Titrations were performed by adding successive portions of the TfOH acid solution to the test compound solution in portions of 0.2 molar equivalents. All spectra and chemical shift tables from the titration experiments are available in the Supporting Information.

### 4.5 *In Vitro* Pharmacology

#### 4.5.1 Affinity at σ_1_ and σ_2_ Receptors

Brain and liver homogenates for σ_1_R and σ_2_R binding assays were prepared from male Dunkin-Hartley guinea pigs and Sprague-Dawley rats, respectively (ENVIGO RMS S.R.L., Udine, Italy), as previously reported.^23,61,62^*In vitro* σ_1_R ligand binding assays were carried out in a Tris buffer (50 mM, pH 7.4) for 150 min at 37 °C. The thawed membrane preparation of the guinea pig brain cortex was incubated with increasing concentrations of test compounds and [^3^H](+)-pentazocine (2 nM) in a final volume of 0.5 mL. Unlabeled (+)-pentazocine (10 μM) was used to measure nonspecific binding. Bound and free radioligands were separated by fast filtration under reduced pressure using a Millipore filter apparatus through Whatman GF 6 glass fiber filters, which were presoaked in a 0.5% poly(ethyleneimine) water solution. Each filter paper was rinsed three times with ice-cold Tris buffer (50 mM, pH 7.4), dried at rt, and incubated overnight with scintillation fluid into pony vials. The bound radioactivity was determined using a liquid scintillation counter (Beckman LS 6500).^61,63^*In vitro* σ_2_R ligand binding assays were carried out in Tris buffer (50 mM, pH 8.0) for 120 min at rt. The thawed membrane preparation of rat liver was incubated with increasing concentrations of test compounds and [^3^H]DTG (2 nM) in the presence of (+)-pentazocine (5 μM) as σ_1_ masking agent in a final volume of 0.5 mL. Nonspecific binding was evaluated with unlabeled DTG (10 μM). Bound and free radioligands were separated by fast filtration under reduced pressure using a Millipore filter apparatus through Whatman GF 6 glass fiber filters, which were presoaked in a 0.5% poly(ethyleneimine) water solution. Each filter paper was rinsed three times with ice-cold Tris buffer (10 mM, pH 8), dried at rt, and incubated overnight with scintillation fluid into pony vials. The bound radioactivity was determined using a liquid scintillation counter (Beckman LS 6500).^64^ The *K*_i_ values were calculated with the program GraphPad Prism 7.0 (GraphPad Software, San Diego, CA, USA). The *K*_i_ values are given as mean value ± CI from at least two independent experiments performed in duplicate.

#### 4.5.2 Affinity at Histamine Receptors

Compounds (as oxalate salts) were tested in H_3_R *in vitro* binding studies using the methods described previously.^21−23^ Ligands were tested at 5 to 11 appropriate concentrations in a [^3^H]*N*^α^-methylhistamine (*K*_D_ = 3.08 nM) radioligand depletion assay to determine the affinity at human recombinant histamine H_3_R stably expressed in HEK-293 cells. Radioligand binding experiments at the H_1_R, H_2_R, and H_4_R were performed as previously described in Rosier et al.^65^ and Bartole et al.^66^ The following radioligands and concentrations were used: [^3^H]mepyramine (hH_1_R, *K*_d_ = 5.1 nM, *c* = 5 nM) (Novandi Chemistry AB, Södertälje, Sweden), [^3^H]UR-DE257 (hH_2_R, *K*_d_ = 66.9 nM, *c* = 50 nM),^67^ and [^3^H]UR-PI294 (hH_4_R, *K*_d_ = 3.6 nM, *c* = 4 nM)^68^ at HEK293T-SP-FLAG-hH_*x*_R (*x* = 1, 2, or 4) expressing the respective hHR. Data represent mean values ± CI from three independent experiments, each performed in triplicate. The normalized competition binding curves were then fitted with a four-parameter logistic fit yielding IC_50_ values using the Prism 8.4.3 software (GraphPad, San Diego, CA). The *K*_i_ values were estimated from the Cheng–Prusoff equation.^69^

#### 4.5.3 Cyclic Adenosine Monophosphate (cAMP) Accumulation Assay

The LANCE Ultra cAMP assay (Perkin Elmer), a homogeneous time-resolved fluorescence resonance energy transfer (TR-FRET) immunoassay, is designed to measure cAMP produced upon modulation of adenylyl cyclase activity by GPCRs. The assay is based on the competition between the europium (Eu) chelate-labeled cAMP tracer and sample cAMP for binding sites on cAMP-specific monoclonal antibodies labeled with the ULight dye. For measurement, HEK293 cells expressing hH_3_R^34^ were resuspended in HBSS buffer containing 0.1% BSA, HEPES (5 mM), and phosphodiesterase inhibitor RO-201724 (100 μM) as previously described.^22^ An antagonist dose–response experiment was performed in 384-well white opaque plates using 3000 cells/well stimulated with 3 μM forskolin and 30 nM (*R*)-(−)-α-methylhistamine as reference H_3_R agonist. Cell treatments were performed for 30 min at room temperature, and the cAMP production was assayed by the addition of Eu-cAMP tracer and ULight-anti-cAMP solution in detection buffer according to the manufacturer’s instructions. The TR-FRET signal was measured 60 min later using the EnVision microplate reader (Perkin Elmer), and the amount of released cAMP was calculated based on the cAMP standard curve. Experiments were conducted twice in triplicates, and IC_50_ values were determined using the Prism 8.4.3 software.

### 4.6 *In Silico* Studies. Molecular Modeling: Docking Studies and Molecular Dynamics Simulations

#### 4.6.1 Structures of the Receptors

The histamine H_3_ receptor homology model was built using the AlphaFold server,^70,71^ and we chose the model with the highest values of the predicted local-distance difference test (pLDDT). The structures of σ_1_R were retrieved from the Protein Data Bank^72,73^ (PDB ID: 5HK1,^74^5HK2,^74^6DJZ,^75^6DK0,^75^6DK1^75^); however, on the basis of the similarity of crystalized ligand and preliminary research, we choose the crystal structure of σ_1_R in complex with the antagonist haloperidol^17^ (PDB ID: 6DJZ).

#### 4.6.2 Induced Fit Docking (IFD)

The three-dimensional structures of the ligands were prepared using LigPrep v3.6,^76^ and the appropriate ionization states at pH = 7.0 ± 0.5 were assigned using Epik v3.4.^77^ The Protein Preparation Wizard^78^ was used to assign the bond orders and appropriate amino acid ionization states and to check for steric clashes for the sigma-1 crystals. The receptor grid was generated (OPLS3 force field)^79^ by centering the grid box of the sigma-1 receptor with a size of 8 Å on crystalized ligands and the grid box of histamine H_3_R with a size of 10 Å on D3.32. Automated flexible docking was performed using Glide v6.9^80,81^ at the SP level.

#### 4.6.3 The QM/MM Optimization

The L-R complexes selected in IFD procedure were next optimized using the QM/MM approach using QSite.^82^ The QM area containing the ligand and the amino acid side chains interacting with ligands was described by a combination of DFT hybrid functional B3LYP and LACVP* basis sets, whereas the rest of the system was optimized using the OPLS3 force field.

#### 4.6.4 Quantum Polarized Ligand Docking (QPLD)

The optimized complexes of **KSK68** and **12** were used as a grid for the next steps. The receptor grids were generated (OPLS3 force field) by centering the grid box with a size of 8 Å on **KSK68** and **12**. Docking was performed by the quantum-polarized ligand docking (QPLD) procedure^83^ that involves the QM-derived ligand atomic charges in the protein environment at the 3-21G/BLYP level. For each ligand, the five poses were obtained.

#### 4.6.5 Binding Free Energy Calculations

GBSA (generalized-born/surface area) was used to calculate the binding free energy based on the ligand–receptor complexes generated by the QPLD procedure. The ligand poses were minimized using the local optimization feature in Prime, the flexible residue distance was set to 6 Å from a ligand pose, and the ligand charges obtained in the QPLD stage were used. The energies of complexes were calculated with the OPLS3e force field and generalized-born/surface area continuum solvent model. To assess the influence of an appropriate tautomeric and protonation state, the ΔΔ*G* was calculated as a difference between binding free energy (Δ*G*) of the piperidine monoprotonated derivative and piperazine analogues.

#### 4.6.6 Molecular Dynamics Simulations

Selected ligand–receptor complexes underwent MD simulations to confirm the stability of docking poses obtained in previous stages. They were carried out in Desmond,^16^ with the application of the TIP3P solvent model^17^ and POPC membrane model in the case of the H_3_R. The remaining conditions were as follows: OPLS3e force-field, pressure of 1.01325 bar, temperature of 300 K, orthorhombic box shape with a size of +10 Å × +10 Å × +10 Å, application of the neutralization of the system by addition of the appropriate number of Cl– ions and relaxation before simulation, simulation length: 1000 ns. Ligand–protein contacts occurring during MD simulations were analyzed with the use of the Simulation Interaction Diagram (Schrödinger Suite).

#### 4.6.7 Protonation Studies

The protonation studies were carried out in two software packages: InstantJChem and Epik.^77^ In the latter case, water was selected as a solvent.

### 4.7 Determination of Selected ADMET Parameters

#### 4.7.1 Permeability across Lipid Membranes

The precoated PAMPA Plate System Gentest was obtained from Corning (Tewksbury, MA, USA). It consists of a 96-well receiver filter plate that has been precoated with structured layers of phospholipids and a matched donor microplate. The stock solutions of tested compounds and reference drugs were diluted in the PBS buffer (pH 7.4) to the final concentration of 100 μM according to the method described previously.^21^ The compounds were applied into the donor wells (300 μL), and PBS buffer was placed in acceptor wells (200 μL). Plates were incubated in RT for 5 h. By using the UPLC–MS spectrometry (Waters ACQUITY TQD system with the TQ Detector, Waters, Milford, USA) with an internal standard, the exact quantity of molecules that penetrated from donor to acceptor wells through phospholipid membrane was estimated. The permeability coefficients (*P*_e_, cm/s) were calculated using the formula provided by the PAMPA Plate System manufacturer.^49^

#### 4.7.2 Serum Metabolic Stability

Acetonitrile (LC–MS grade), formic acid (LC–MS grade), and DMSO (HPLC grade) were obtained from Sigma Aldrich. Imipramine was also acquired from Sigma Aldrich. Water MilliQ was used for chromatography with 18 MΩ·cm.

The plasma was warmed to 37 °C for 10 min, mixed, and centrifuged to pellet any aggregated protein. Calcium chloride (500 mM, 0.5 mL added to 25 mL of plasma) was then added to the plasma to allow clot formation. The plasma was centrifuged at 4 °C for 30 min at 14,000 rpm to remove the clot. The clear supernatant (serum) was then transferred to an assay plate (249 μL) using the Bravo Automated Liquid Handling Platform (Agilent Technologies). The serum was equilibrated to 37 °C, and biotransformation was initiated by adding 1 μL of the compound solution in DMSO to achieve final concentrations of 0.1, 0.5, and 1 mM with 250 μL final incubation volume. Samples (25 μL) were taken at 0, 1, 2, 4, 6, and 24 h and added to 100 μL acetonitrile with 100 nM imipramine as internal standard for plasma deproteinization. The samples were vortexed for 1 min and then centrifuged at 4 °C for 30 min at 14,000 rpm. The clear supernatants were analyzed by LC–MS. An Exion HPLC system consisting of a degasser (DGU-20A) and an autosampler (SIL-HTA) was used. LC was performed on an analytical column (Waters BEH C18; 1.7 μm, 50 × 2.1 mm). The mobile phase consisted of acetonitrile–water with 0.1% formic acid. The flow rate was 0.5 mL/min with a column compartment temperature of 50 °C and injection volume of 1 μL.

### 4.8 *In Vivo* Pharmacology

#### 4.8.1 Influence on Spontaneous Locomotor Activity

This test was carried out according to the procedure described elsewhere.^84^ The animals were injected (i.p.) with **12** at doses of 5, 15, and 30 mg/kg and placed in the activity cages individually (30 min before the experiment). The number of light-beam crossings was counted in each group during the next 30 min in 10 min intervals.

#### 4.8.2 Influence on Motor Coordination in the Rotarod Test

The test was carried out on the semiautomatic rotarod apparatus (Rotarod apparatus Panlab/Harvard Apparatus, LE 8200, Spain) according to the method previously described in the literature.^85^ The mice were trained for 3 days on the rod rotating at a constant speed of 18 rotations per minute (rpm). During each training session, the animals were placed on the rod for 3 min with an unlimited number of trials. On the test day (24 h after the final training trial), 30 min before the rotarod test, the mice were pretreated (i.p.) with the test compound (15 mg/kg). Then, the animals were tested on the rotarod revolving at 6, 18, and then 24 rpm. Motor impairments, defined as the inability to remain on the rotating rod for 1 min, were measured at each speed.

#### 4.8.3 Formalin Test

The procedure used was essentially the same as that described previously.^86,87^ Briefly, 20 μL of 1.5% formalin solution was injected intraplantarly (i.pl.) into the mice right hind paw using a 26-gauge needle. Immediately after formalin injection, the animals were placed individually in an observation chamber made of glass and were observed for the next 30 min. The total time (in seconds) spent on licking the injected paw during periods of 0–5 min (early phase, neurogenic) and 15–30 min (late phase, inflammatory) was measured and was considered as an indicator of nociceptive behavior. Before formalin injection, different groups of mice were treated i.p. with the vehicle (10 mL/kg, negative control) and the varying doses of **12** (5, 10, 15, and 30 mg/kg).

#### 4.8.4 Capsaicin-Induced Nociception

The procedure used was similar to that described previously.^88,89^ The pain reaction was induced by the injection of 20 μL of capsaicin solution (1.6 μg/paw made in PBS) under the skin of the dorsal surface of the mice right hindpaw. The animals were observed individually in an observation chamber for 5 min following capsaicin injection. The amount of time spent licking the injected paw was recorded by means of the stopwatch and was considered as indicative of pain. The animals were pretreated with the vehicle (10 mL/kg, negative control) and investigated compound (10, 15, and 30 mg/kg).

#### 4.8.5 Loperamide-Induced Antinociception

Female CD1 mice (Charles River, Barcelona, Spain) were used in all experiments. The experiments were performed during the light phase (from 9:00 a.m. to 3:00 p.m.). Animal care was provided in accordance with institutional (Research Ethics Committee of the University of Granada, Granada, Spain), regional (Junta de Andalucía, Spain), and international standards (European Communities Council directive 2010/63).

We aimed to test whether compound **12** behaved *in vivo* as σ_1_ antagonists or agonists. As reference σ_1_ compounds, we used **S1RA** (4-[2-[[5-methyl-1-(2-naphthalenyl)1*H*-pyraol-3-yl]oxy]ethyl]morpholine hydrochloride), a known selective σ_1_ receptor antagonist (DC Chemicals, Shanghai, China), and PRE-084 (2-[4-morpholinethyl]1-phenylcyclohexanecarboxylate hydrochloride) (Tocris Cookson Ltd., Bristol, United Kingdom), a selective σ_1_ receptor agonist.^18^**S1RA** and PRE-084 were dissolved in sterile physiologic saline (0.9% NaCl). Compound **12** was dissolved in 2% Tween 80 (Sigma-Aldrich, Madrid, Spain) in ultrapure water and heated until dissolved before injection. We previously tested that this solvent did not alter the animals’ behavioral response to the mechanical stimulation (data not shown). All these compounds (or their solvents) were administered intraplantarly (i.pl.) into the right hind paw in a volume of 20 μL using a 1710 TLL Hamilton microsyringe (Teknokroma, Barcelona, Spain) with a 30^1/2^-gauge needle. The i.pl. injection was made 5 min before nociceptive testing to minimize systemic absorption of the compounds. When PRE-084 was associated with **S1RA** or **12**, drugs were dissolved in the same solution and injected together to avoid paw lesions from multiple injections.

As it is known that σ_1_ antagonism can enhance opioid antinociception and that σ_1_ agonism reverses this effect,^90^ we tested whether our compound modulated the antinociceptive effect induced by the opioid agonist loperamide hydrochloride (Sigma-Aldrich). This drug was dissolved in 1% dimethylsulfoxide (DMSO) (Merck KGaA, Darmstadt, Germany) in ultrapure water and injected subcutaneously into the interscapular area in a volume of 5 mL/kg 30 min before behavioral testing. Naloxone methiodide (Sigma-Aldrich) was used as a peripheral opioid antagonist^91^ and was dissolved in physiological saline and s.c. administered 5 min before loperamide injection.

Nociceptive stimulation of the hind paw of the animals was made with an Analgesimeter (Model 37215, Ugo-Basile, Varese, Italy) as previously described.^23,90^ After drug administration, mice were gently pincer grasped between the thumb and index fingers by the skin above the interscapular area. Then, a blunt cone-shaped paw-presser was applied at a constant intensity of 450 g to the dorsal surface of the hind paw until the animal showed a struggle response. The struggle latency was measured with a chronometer. Evaluations were done twice alternately to each hind paw at intervals of 1 min between stimulations.

Statistical analysis was carried out with the two-way analysis of variance (ANOVA) followed by a Bonferroni *post hoc* test. ANOVA was performed with the SigmaPlot 12.0 program. The differences between values were considered significant when the *p* value was below 0.05.

#### 4.8.6 Oxaliplatin-Induced Neuropathy

Neuropathy was induced by the administration of a single dose (10 mg/kg) of oxaliplatin (OXPT) dissolved in a 5% glucose solution (Polfa Kutno, Poland). To assess the sensitivity to mechanical stimuli, the von Frey test was carried out using the electronic von Frey unit (Bioseb, France). The apparatus was supplied with a single flexible filament, which was used to apply increasing force (from 0 to 10 g) against the plantar surface of the hind paw of the mouse. The crossing of pain threshold resulted in the paw withdrawal and subsequent recording of the mechanical pressure that evoked the nocifensive response. The measurement was done before the OXPT administration and 3 h and 7 days afterward. The compounds were administrated to the animals with tactile allodynia observed as a statistically significant decrease in pain threshold. On the day of the experiment, each mouse was placed in an observation chamber with a wire mesh bottom and was allowed to habituate for 1 h. After the habituation period, the mouse pain threshold was tested three times alternately with at least 30 s gap between each measurement. The mean of these three consecutive measurements was taken as the baseline value. The mice with tactile allodynia were i.p. pretreated with the tested compound, and 30 min later, the animals were tested again according to the same procedure.^92^

#### 4.8.7 Chronic Constriction Injury of the Sciatic Nerve

All animal procedures were performed following the recommendations of the International Association for the Study of Pain^93^ and the NIH Guide for the Care and Use of Laboratory Animals. Experiments were approved by the II Local Ethics Committee Branch of the National Ethics Committee for Experiments on Animals based at the Maj Institute of Pharmacology, Polish Academy of Sciences, Krakow, Poland (approval number: 24/2022). Care was taken to minimize animal suffering and minimize the number of animals used (3R policy). Adult male albino Swiss CD-1 mice (initially weighing 18–20 g) were purchased from Charles River Laboratories (Hamburg, Germany). The animals were housed in groups of seven under controlled conditions (temp. 21 ± 2 °C; 12 h light/dark cycle, lights on at 6:00 a.m.) with *ad libitum* food and water.

The model of chronic constriction injury (CCI) to the sciatic nerve was established according to Bennett and Xie.^94^ The surgical method was performed under isoflurate anesthesia (2% isoflurane in 100% oxygen with a flow of 1.5 L/min).^95^ Below the right hipbone, a small incision was performed, and three ligatures (4/0 silk) around the sciatic nerve were made (with 1 mm spacing) until a brief twitch in the respective hind limb was observed. All mice that underwent the CCI procedure developed hypersensitivity to mechanical and thermal stimuli. Behavioral tests were performed at day 14 after injury. Compound **12** was dissolved in 10% DMSO/10% (2-hydroxypropyl)-β-cyclodextrin/water and administered i.p. at doses 5, 10, and 15 mg/kg (injection volume 10 mL/kg of body weight). The control group received the vehicle (10% DMSO/10% (2-hydroxypropyl)-β-cyclodextrin/water).

##### 4.8.7.1 Behavioral Tests

Behavioral experiments were performed between 8:00 a.m. and 2:00 p.m. Across the experiments, we compared vehicle-treated mice with **12**-treated animals. Experiments were performed 30, 90, and 180 min (von Frey test); 35, 95, and 185 min (cold plate test); and 40, 100, and 190 min (tail flick test) after the vehicle or **12** administration.

The von Frey test was used to measure mechanical hypersensitivity as described in a previous publication.^95^ The animals were placed in cages with a wire net floor. The reaction (paw withdrawal, shaking, or licking) of the ipsilateral (Figure 15) and contralateral (Figure S33) paw of CCI-exposed mice to the mechanical stimulator (calibrated nylon monofilaments (0.6–6 g; Stoelting)) in serial increments was observed.

Sensitivity to noxious thermal stimuli was assessed with the cold plate test (Cold/Hot Plate Analgesia Meter, Columbus Instruments) as previously described.^95^ The temperature of the plate was kept at 2 °C, and the cutoff latency was 30 s. The mice were placed on the cold plate, and the time until lifting of the injured paw was recorded.

The responsiveness to thermally induced pain was determined by a tail flick analgesic meter (Tail-Flick Unit; Ugo Basile) as previously described.^95^ Tail flick latency was measured on the dorsal part of the tail at two-thirds of its length by applying a focused beam of light (thermal stimulus). The time interval between onset of the thermal stimulus and withdrawal of the tail from the beam was recorded; the cutoff latency was 9 s to prevent tissue damage.

##### 4.8.7.2 Data and Statistical Analysis

The behavioral data in Figure 14 are presented as a percentage of the maximal possible effect [% MPE = 100% × (measured response – basal)(cutoff value – basal)] of drug action. In the Supporting Information, the results from the von Frey test are expressed as pressure (g) applied with the filament. The behavioral data are presented as mean ± SEM. The one-way ANOVA was performed, and the differences between the groups were further analyzed with Bonferroni’s *post hoc* test. Significant differences between group are indicated when *p < 0.05, **p < 0.01, and ***p < 0.001. The analysis and charts were prepared using GraphPad Prism v.9.1.2 (GraphPad Software, La Jolla, CA, USA).

## Supporting Information Available

Protonation investigation based on crystal structure analysis; determination of the basicity of selected piperidine and piperazine derivatives by potentiometric titration; protonation investigation based on NMR spectroscopy measurements in pH-controlled environment; *in vivo* pharmacological activity; *in silico* studies; molecular modeling: docking studies and molecular dynamics simulations; and ^1^H and ^13^C NMR spectra HRMS analysis and HPLC traces (PDF)
Molecular formula strings (CSV)
3_in_H3 (PDB)
3_in_sigma1 (PDB)
7_in_H3 (PDB)
7_in_sigma1 (PDB)
H3_compound_3 (PDB)
H3_compound_7 (PDB)
KSK67_in_H3_pose1 (PDB)
KSK67_in_H3_pose2 (PDB)
KSK68_in_H3 (PDB)
KSK94_in_sigma1_pose1 (PDB)
KSK94_in_sigma1_pose2 (PDB)
KSK100_in_sigma1 (PDB)
sigma_compound_3 (PDB)
sigma_compound_7 (PDB)

## Supplementary Material

jm3c00430_si_002.csv

jm3c00430_si_003.pdb

jm3c00430_si_004.pdb

jm3c00430_si_005.pdb

jm3c00430_si_006.pdb

jm3c00430_si_007.pdb

jm3c00430_si_008.pdb

jm3c00430_si_009.pdb

jm3c00430_si_010.pdb

jm3c00430_si_011.pdb

jm3c00430_si_012.pdb

jm3c00430_si_013.pdb

jm3c00430_si_014.pdb

jm3c00430_si_015.pdb

jm3c00430_si_016.pdb

## Abbreviations

ADME: absorption, distribution, metabolism, excretion
br: broad
cAMP: 3′,5′-cyclic adenosine monophosphate
CC: column chromatography
CCDC: Cambridge Crystallographic Data Centre
CCI: chronic constriction injury
CI: confidence interval
CNS: central nervous system
d: doublet
dd: doublet of doublets
DMSO: dimethyl sulfoxide
GPCR: G protein-coupled receptor
H_1_R: histamine H_1_ receptor
H_2_R: histamine H_2_ receptor
H_3_R: histamine H_3_ receptor
HB: hydrogen bond
HEK: human embryonic kidney cells
HPLC: high-performance liquid chromatography
HRMS: high-resolution mass spectra
i.p.: intraperitoneal
IFD: induced fit docking
m.p.: melting point
m: multiplet
MD: molecular dynamics
MM-GBSA: molecular mechanics generalized born surface area
MPE: maximum possible effect
MTDLs: multitarget-directed ligands
NMDA: *N*-methyl-d-aspartate receptor
NMR: nuclear magnetic resonance spectroscopy
OXPT: oxaliplatin
PAMPA: parallel artificial membrane permeability assay
PDB: Protein Data Bank
*P*_e_: permeability coefficient
ppm: parts per million
qd: quartet of doublets
QPLD: quantum polarized ligand docking
quin: quintet
RMSD: root-mean-square deviation
RT: room temperature
s.c.: subcutaneous
s: singlet
SD: standard deviation
SEM: standard error of the mean
t: triplet
TfOH: trifluoromethanesulfonic acid (triflic acid)
TLC: thin-layer chromatography
*t*_R_: retention time
TR-FRET: time-resolved fluorescence resonance energy transfer
TRPV1: transient receptor potential vanilloid type 1
tt: triplet of triplets
UPLC–MS: ultra-performance liquid chromatography–mass spectrometry
σ_1_R: sigma-1 receptor
σ_2_R: sigma-2 receptor
