# Supplementary material for: Dual Piperidine-Based Histamine H3 and Sigma-1 Receptor Ligands in the Treatment of Nociceptive and Neuropathic Pain
Source: J Med Chem. 2023 Jul 7;66(14):9658–83. doi: 10.1021/acs.jmedchem.3c00430 (PMC10388327; doi:10.1021/acs.jmedchem.3c00430)
Supplement: Supplementary file 1 — jm3c00430_si_001.pdf [file jm3c00430_si_001.pdf]

Supplementary Information

Dual piperidine-based histamine H_3 and sigma-1 receptor ligands in the treatment of nociceptive and neuropathic pain

Katarzyna Szczepańska[+ a,b], Tadeusz Karcz[+ a,*], Maria Dichiara [c], Szczepan Mogilski [d], Justyna Kalinowska-Tłuścik [e], Bogusław Pilarski [f], Arkadiusz Leniak [g] , Wojciech Pietruś [b,g], Sabina Podlewska [b], Katarzyna Popiołek-Barczyk [h], Laura J. Humphrys [i], M. Carmen Ruiz-Cantero [j], David Reiner-Link [k], Luisa Leitzbach [k], Dorota Łażewska [a], Steffen Pockes [i], Michał Górka [g], Adam Zmysłowski [g], Thierry Calmels [l], Enrique J. Cobos [j], Agostino Marrazzo [c], Holger Stark [k], Andrzej J. Bojarski [b], Emanuele Amata [c,*], Katarzyna Kieć-Kononowicz [a,*]

[a] *Department of Technology and Biotechnology of Drugs, Faculty of Pharmacy, Jagiellonian University Medical College, Medyczna 9, Kraków 30-688, Poland*
[b] *Maj Institute of Pharmacology, Polish Academy of Sciences, Department of Medicinal Chemistry, Smętna 12, Kraków 31-343, Poland*
[c] *Department of Drug and Health Sciences, University of Catania, V.le A. Doria, 95125 Catania, Italy*
[d] *Department of Pharmacodynamics, Faculty of Pharmacy, Jagiellonian University Medical College, Medyczna 9, Kraków 30-688, Poland*
[e] *Department of Crystal Chemistry and Crystal Physics, Faculty of Chemistry Jagiellonian University, Gronostajowa 2, 30-387 Kraków, Poland*
[f] *Cerko Sp. z o.o. Sp.k, Al. Zwycięstwa 96/98, 81-451 Gdynia, Poland*
[g] *Celon Pharma S.A., R&D Centre, Marymoncka 15, 05-152, Kazuń Nowy, Poland*
[h] *Maj Institute of Pharmacology, Polish Academy of Sciences, Department of Neurochemistry, Smętna 12, Kraków 31-343, Poland*
[i] *Institute of Pharmacy, Faculty of Chemistry and Pharmacy, University of Regensburg, Universitätsstraße 31, D-93053 Regensburg, Germany*
[j] *Department of Pharmacology and Neurosciences Institute (Biomedical Research Center), University of Granada, and Biosanitary Research Institute ibs.Granada, Avenida de la Investigación 11, 18016 Granada, Spain*
[k] *Institute of Pharmaceutical and Medicinal Chemistry, Heinrich Heine University Düsseldorf, Universitaetsstr. 1, 40225 Duesseldorf, Germany*
[l] *Bioprojet-Biotech, 4rue du Chesnay Beauregard, 35762 Saint-Gregoire Cedex, France*

* Corresponding authors. E-mail addresses: t.karcz@uj.edu.pl (Tadeusz Karcz), eamata@unict.it (Emanuele Amata), mfkonono@cyf-kr.edu.pl (Katarzyna Kieć-Kononowicz).
[+] These authors contributed equally to this work.

Contents

1. Protonation investigation based on crystal structure analysis 2
2. Determination of the basicity of selected piperidine and piperazine derivatives by potentiometric titration 11
3. Protonation investigation based on NMR spectroscopy measurements in pH-controlled environment....... 12
4. *In silico* studies. Molecular modeling: docking studies and molecular dynamics simulations........ 27
5. *In vivo* pharmacological activity 32
6. ^{1}H-, ^{13}C-NMR spectra, HRMS analysis and HPLC traces 34

1. Protonation investigation based on crystal structure analysis

Table S1. Crystal data and final refinement results for structures of oxalate salts.

	**KSK67**	**KSK68**	**KSK68_OH**
Empirical moiety formula	$C_{22}H_{29}N_3O_{12}$,<br>2 C_2HO_4, 2 H_2 O	$C_{25}H_{30}N_2O_6$,<br>0.5 $C_2H_2O_4$	$C_{23}H_{29}N_2O_2$,<br>0.5 C_2O_4, 4 H_2O
Formula weight [g/mol]	581.57	499.53	481.55
Crystal system	Monoclinic	Monoclinic	Triclinic
Space group	C2	C2/*c*	$P\bar{1}$
Unite cell dimensions	a = 15.0969(8) Å<br>b = 5.7291(3) Å<br>c = 30.7622(8) Å<br>α=90°<br>β=94.626(3)°<br>γ=90°	a = 21.5545(6) Å<br>b = 8.4549(2) Å<br>c = 28.2827(8) Å<br>α=90°<br>β=111.415(3)°<br>γ=90°	a = 8.5168(5) Å<br>b = 9.6493(4) Å<br>c = 16.1458(9) Å<br>α=89.241(4)°<br>β=89.175(5)°<br>γ=72.737(5)°
Volume [Å^3]	2652.0(2)	4798.4(2)	1266.92(12)
Z	4	8	2
D_{calc} [Mg/m^3]	1.457	1.383	1.262
μ [mm^{-1}]	0.985	0.856	0.783
F(000)	1232	2120	518
Crystal size [mm^3]	0.2 x 0.15 x 0.02	0.3 x 0.2 x 0.02	0.3 x 0.2 x 0.1
Θ range	2.882° to 75.520°	3.357° to 75.541°	2.737° to 77.567°
Index ranges	-18 ≤ h ≤ 17,<br>-7 ≤ k ≤ 5,<br>-36 ≤ l ≤ 38	-26 ≤ h ≤ 26,<br>-10 ≤ k ≤ 8,<br>-35 ≤ l ≤ 34	-10 ≤ h ≤ 10,<br>-12 ≤ k ≤ 9,<br>-20 ≤ l ≤ 20
Refl. collected	12977	23308	12391
Independent reflections	4718<br>[R(int) = 0.0785]	4842<br>[R(int) = 0.0684]	4996<br>[R(int) = 0.0553]
Completeness [%] to Θ	99.9 (Θ 67.684°)	97.2 (Θ 75.541°)	92.6 (Θ 77.567°)
Absorption correction	Multi-scan	Multi-scan	Multi-scan
Tmin. and Tmax.	0.688 and 1.000	0.778 and 1.000	0.787 and 1.000
Data/ restraints/parameters	4718 / 16 / 504	4847 / 1 / 338	4996 / 4 / 316
GooF on F2	1.101	1.125	1.078
Final R indices [I>2sigma(I)]	R1= 0.0947,<br>wR2= 0.2510	R1= 0.0694,<br>wR2= 0.2063	R1= 0.0727,<br>wR2= 0.2021
R indices (all data)	R1= 0.0947,<br>wR2= 0.2517	R1= 0.0733,<br>wR2= 0.2104	R1= 0.0809,<br>wR2= 0.2131
$\Delta\rho_{max}$, $\Delta\rho_{min}$ [e·Å^{-3}]	0.632 and -0.585	0.469 and -0.434	0.765 and -0.533
Absolute structure parameter	0.07(14)	-	-

Table S2. Crystal data and final refinement results for free bases.

	**KSK67_fb**	**KSK68_fb**	**KSK94_fb**	**KSK100_fb**
Empirical moiety formula	$C_{22}H_{27}N_3O_2$, HI, 2 H_2O	$C_{23}H_{28}N_2O_2$	2 $C_{25}H_{26}N_4O$, HI, 2 H_2O	$C_{26}H_{27}N_3O$
Formula weight [g/mol]	529.40	364.47	960.93	397.50
Crystal system	Monoclinic	Monoclinic	Monoclinic	Triclinic
Space group	$P2_1/n$	$P2_1$	$P2_1/c$	$P\bar{1}$
Unite cell dimensions	a = 10.9621(1) Å<br>b = 8.9179(1) Å<br>c = 23.5157(1) Å<br>α=90°<br>β=94.814(1)°<br>γ=90°	a = 10.3351(1) Å<br>b = 11.6636(1) Å<br>c = 16.9247(1) Å<br>α=90°<br>β=105.438(1)°<br>γ=90°	a = 10.4940(1) Å<br>b = 17.3517(2) Å<br>c = 25.4093(3) Å<br>α=90°<br>β=91.703(1)°<br>γ=90°	a = 7.5156(1) Å<br>b = 10.7481(1) Å<br>c = 13.7353(1) Å<br>α=104.711(1)°<br>β=96.758(1)°<br>γ=96.212(1)°
Volume [Å^3]	2290.76(3)	1966.57(3)	4624.7(9)	1054.62(2)
Z	4	4	4	2
D_{calc} [Mg/m^3]	1.535	1.231	1.380	1.252
μ [mm^{-1}]	11.251	0.618	5.867	0.603
F(000)	1080	784	1992	424
Crystal size [mm^3]	0.3 x 0.15 x 0.07	0.3 x 0.15 x 0.1	0.1 x 0.05 x 0.02	0.3 x 0.2 x 0.01
Θ range	3.773° to 80.481°	2.709° to 80.458°	3.084° to 80.621°	3.365° to 80.401°
Index ranges	$-14 \le h \le 13$,<br>$-10 \le k \le 11$,<br>$-29 \le l \le 30$	$-13 \le h \le 13$,<br>$-14 \le k \le 13$,<br>$-21 \le l \le 21$	$-13 \le h \le 13$,<br>$-20 \le k \le 22$,<br>$-32 \le l \le 31$	$-9 \le h \le 9$,<br>$-13 \le k \le 13$,<br>$-17 \le l \le 17$
Refl. collected	65243	68587	64583	33862
Independent reflections	4983<br>[R(int) = 0.0614]	8403<br>[R(int) = 0.0423]	10037<br>[R(int) = 0.0761]	4538<br>[R(int) = 0.0506]
Completeness [%] to Θ	99.4 (Θ 80.481°)	98.0 (Θ 80.458°)	98.9 (Θ 80.621°)	98.1 (Θ 80.401°)
Absorption correction	Multi-scan	Multi-scan	Multi-scan	Multi-scan
Tmin. and Tmax.	0.633 and 1.000	0.830 and 1.000	0.722 and 1.000	0.548 and 1.000
Data/ restraints/parameters	4983 / 2 / 291	8403 / 1 / 553	10037 / 4 / 612	4538 / 0 / 271
GooF on F2	1.085	1.051	1.0896	1.071
Final R indices [I>2sigma(I)]	R1= 0.0219,<br>wR2= 0.0583	R1= 0.0454,<br>wR2= 0.1235	R1= 0.0578,<br>wR2= 0.1420	R1= 0.0397,<br>wR2= 0.1070
R indices (all data)	R1= 0.0222,<br>wR2= 0.0586	R1= 0.0464,<br>wR2= 0.1248	R1= 0.0634,<br>wR2= 0.1454	R1= 0.0422,<br>wR2= 0.1096
$\Delta\rho_{max}$, $\Delta\rho_{min}$ [e·Å^{-3}]	0.533 and -0.832	0.391 and -0.213	1.211 and -1.014	0.196 and -0.255
Absolute structure parameter	-	0.2(2)	-	-

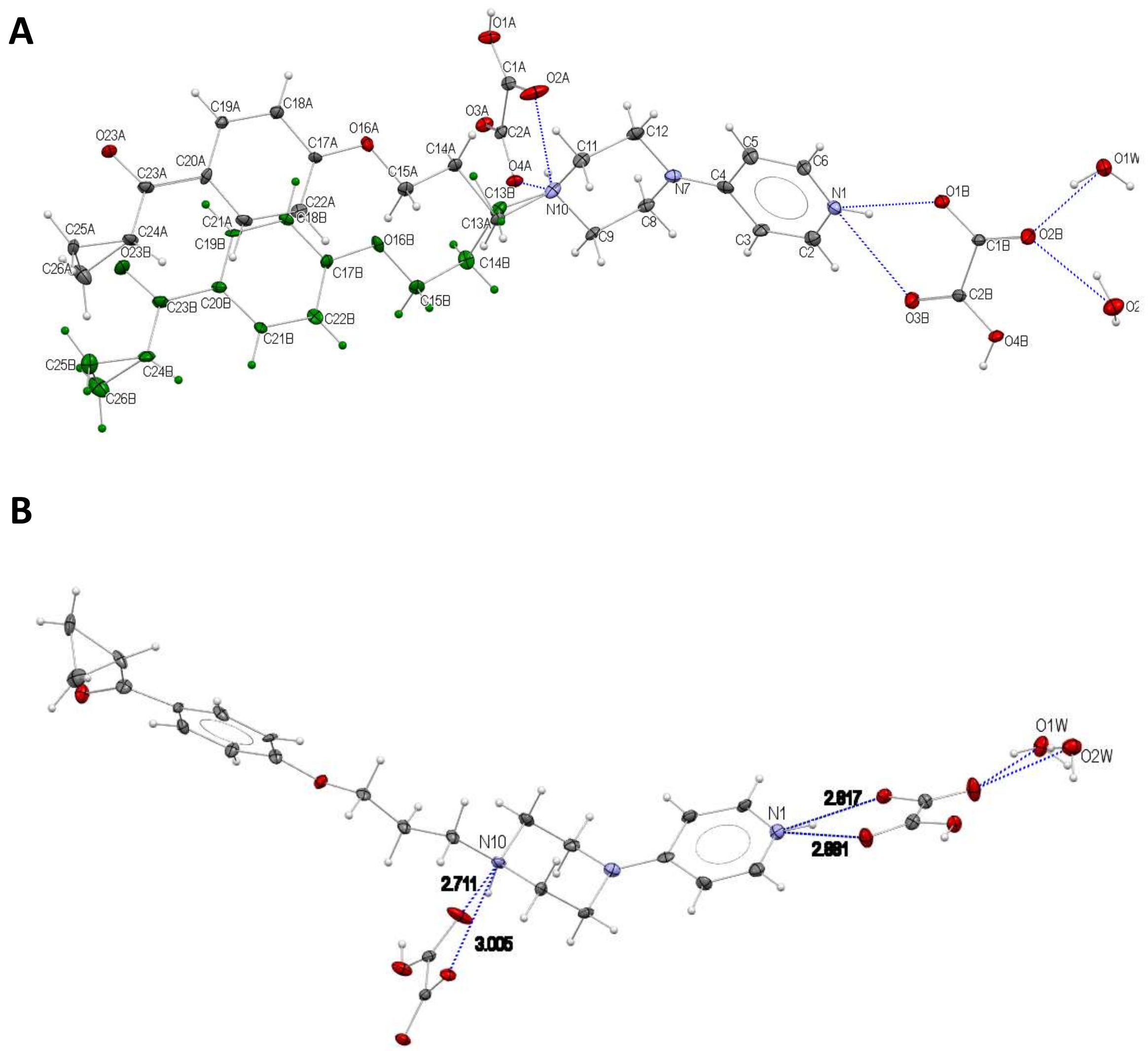

Figure S1. **A**: The asymmetric unit of **KSK67** crystal structure with the atom labeling scheme. A positional disorder is observed – the molecule adopts two alternative conformations in the crystal lattice with site occupancies of 60% and 40% (the less abundant conformation is marked with C and H-atoms in green and labels with B letters at the end of a symbol). **B**: The strongest interactions stabilizing crystal lattice (charge-assisted hydrogen bonds between protonated nitrogen atoms of the main organic compound and oxalate anion & hydrogen bonds involving water molecules incorporated in the crystal structure). Displacement ellipsoids of non-hydrogen atoms are drawn at the 30% probability level. H atoms are presented as small spheres with an arbitrary radius .

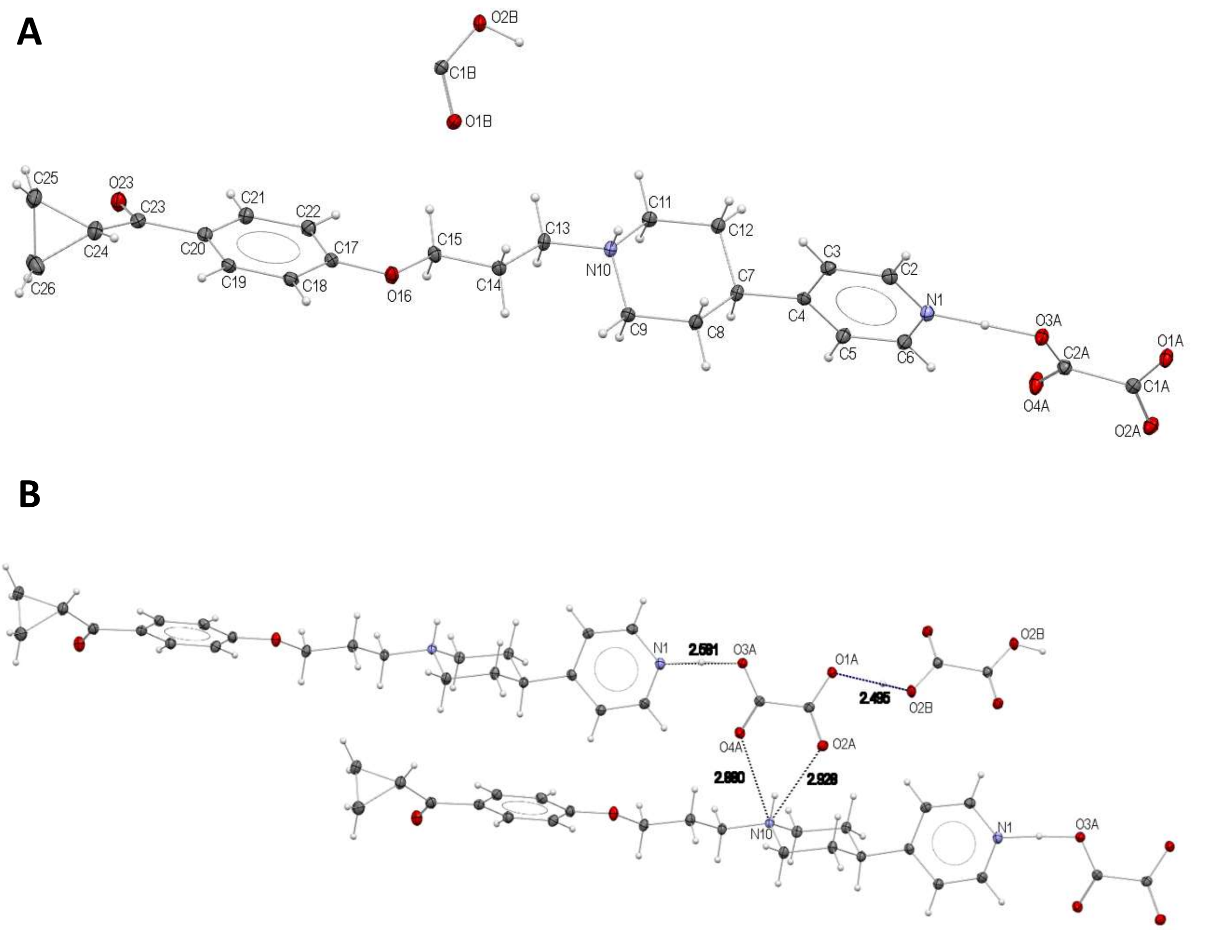

Figure S2. **A**: The asymmetric unit of **KSK68** crystal structure showing the atom labeling scheme. **B**: The strongest interactions stabilizing crystal lattice (charge-assisted hydrogen bonds between protonated nitrogen atoms of the main organic compound and oxalate anion & hydrogen bonds involving neutral oxalic acid molecules as an H-bond donor. Displacement ellipsoids of non-hydrogen atoms are drawn at the 30% probability level. H atoms are presented as small spheres with an arbitrary radius.

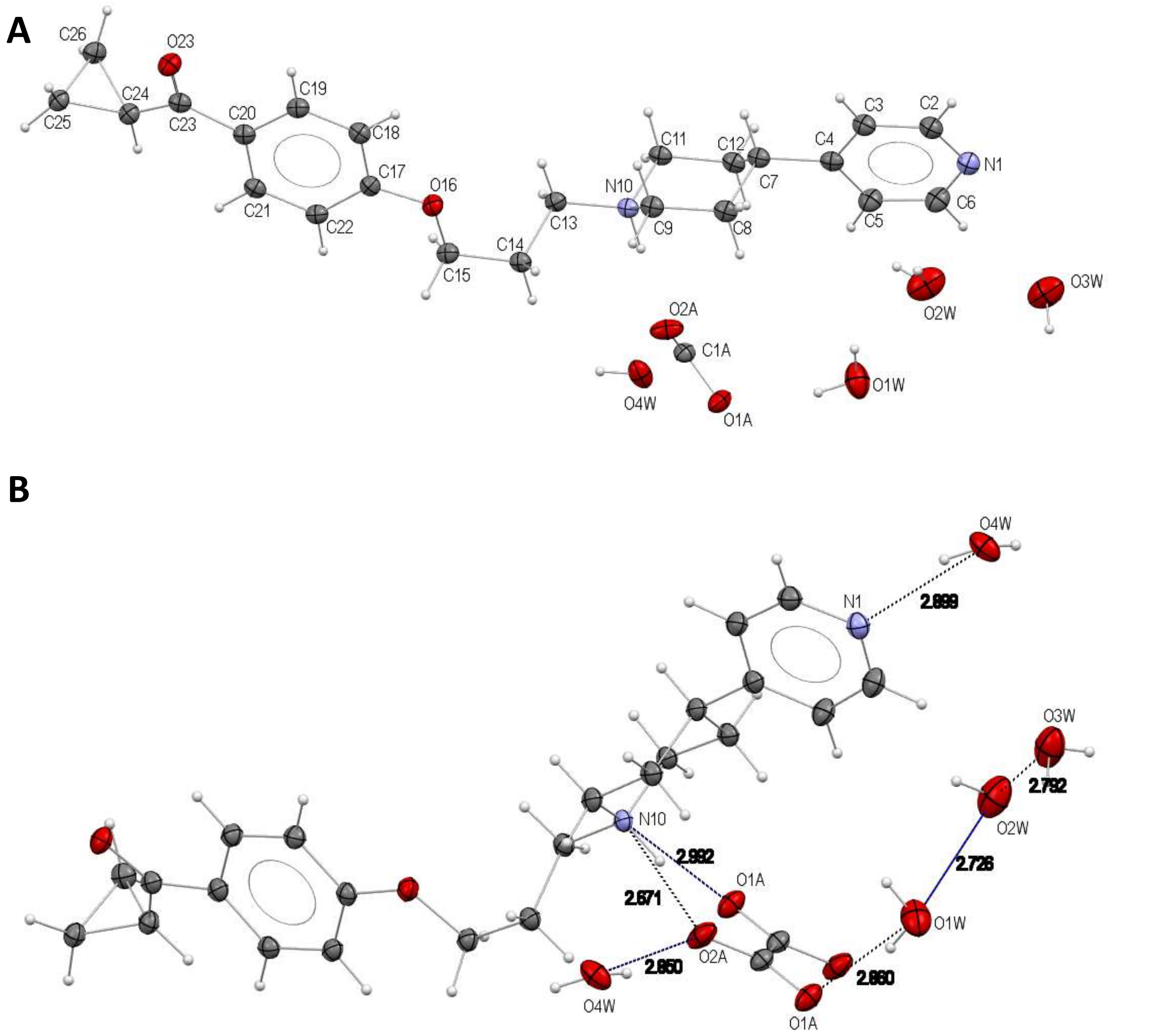

Figure S3. **A**: The asymmetric unit of **KSK68_OH** crystal structure presenting the atom labeling scheme. **B**: The strongest interactions stabilizing crystal lattice (charge-assisted hydrogen bonds between the protonated nitrogen atom of the piperidine moiety and oxalate anion & hydrogen bonds involving water molecules incorporated in the crystal structure, forming expanded H-bonds motifs propagating along [100] axis. Displacement ellipsoids of non-hydrogen atoms are drawn at the 30% probability level. H atoms are presented as small spheres with an arbitrary radius.

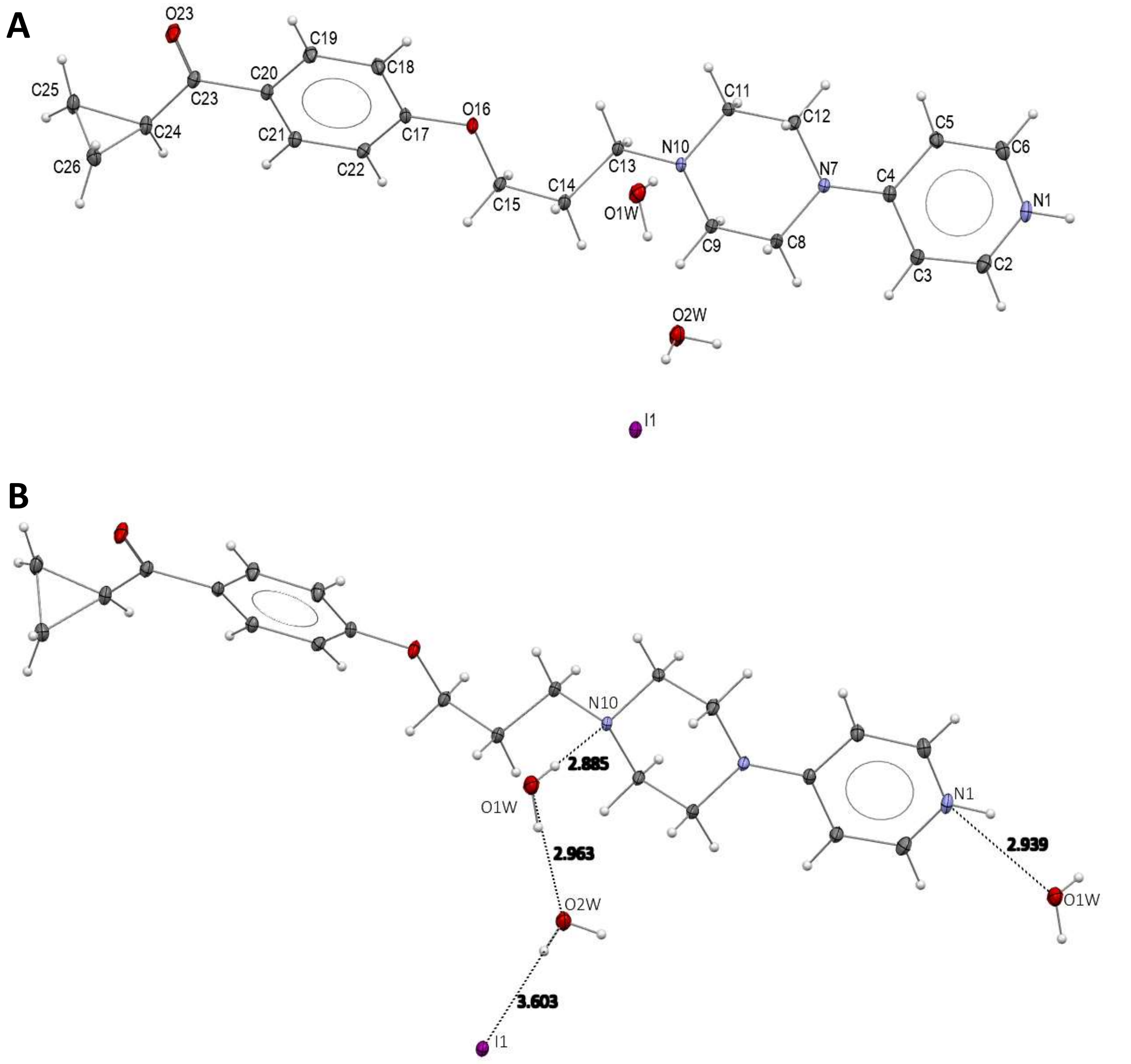

Figure S4. **A**: The asymmetric unit of **KSK67_fb** crystal structure with the atom labeling scheme. The postulated free base was not observed in the neutral form. Instead, an iodide salt was present, with the protonation center at N1 (pyridine) nitrogen atom, suggesting a strongly basic character of this molecular fragment. **B**: The strongest interactions stabilizing crystal lattice. Interestingly, the charge-assisted interaction between counter-ions was not observed. Both cation and anion are involved in hydrogen bonds with water molecules incorporated in the crystal structure. Displacement ellipsoids of non-hydrogen atoms are drawn at the 30% probability level. H atoms are presented as small spheres with an arbitrary radius.

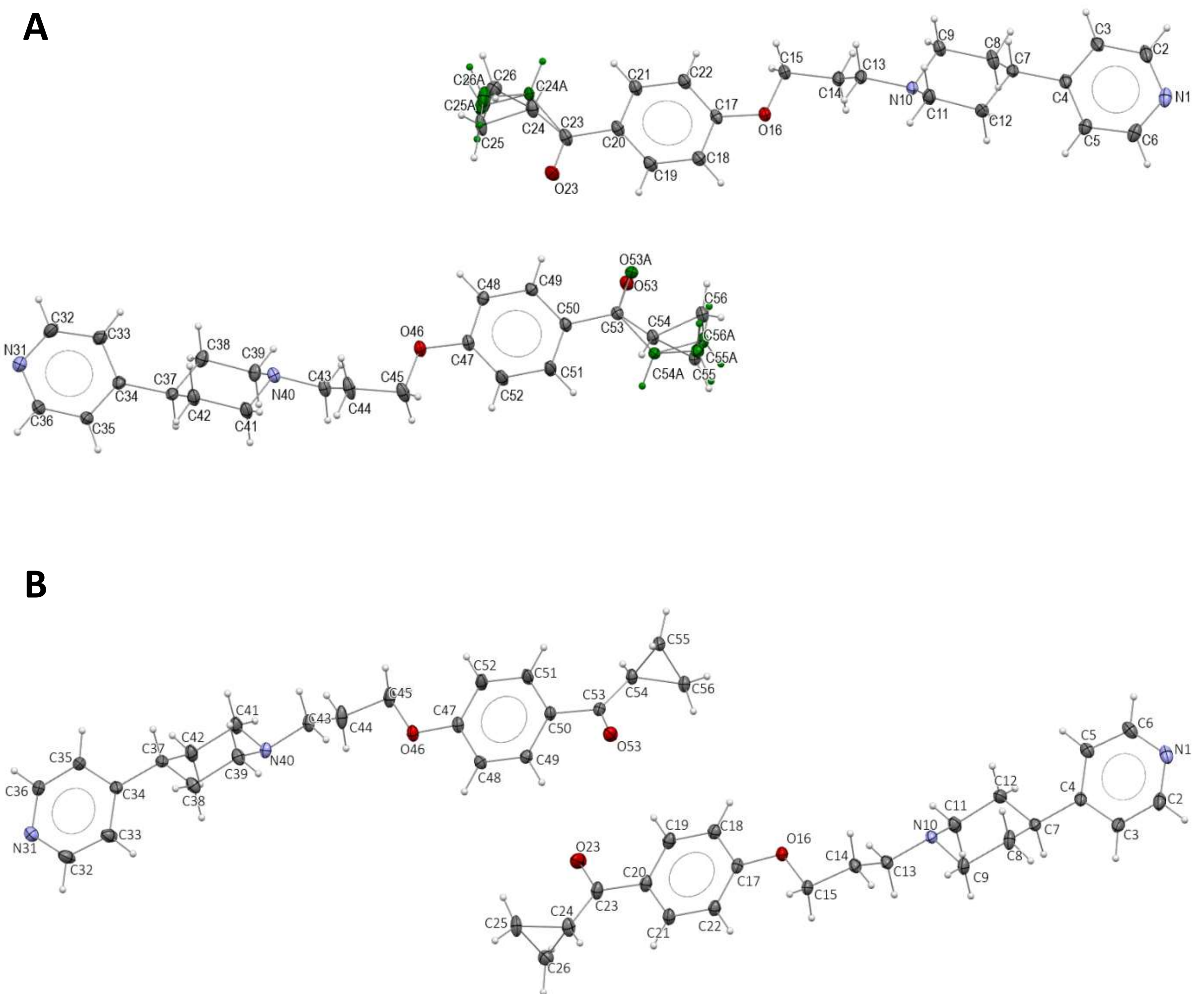

Figure S5. **A**: The asymmetric unit of **KSK68_fb** crystal structure presenting the atom labeling scheme. The asymmetric unit consists of two independent molecules adopting alternative conformations in the crystal lattice. Additionally, a positional disorder is observed for both molecules within the cyclopropyl fragment with site occupancies of 53% and 47% for molecules 1 (with label numbers 1-26), and 51% and 49% for molecule 2 (with label numbers 31-56) - the less abundant conformation for each molecule is marked with C and H-atoms in green and labels with an additional A letter at the end of a symbol. **B**: The asymmetric unit presenting only the most abundant conformations. Displacement ellipsoids of non-hydrogen atoms are drawn at the 30% probability level. H atoms are presented as small spheres with an arbitrary radius.

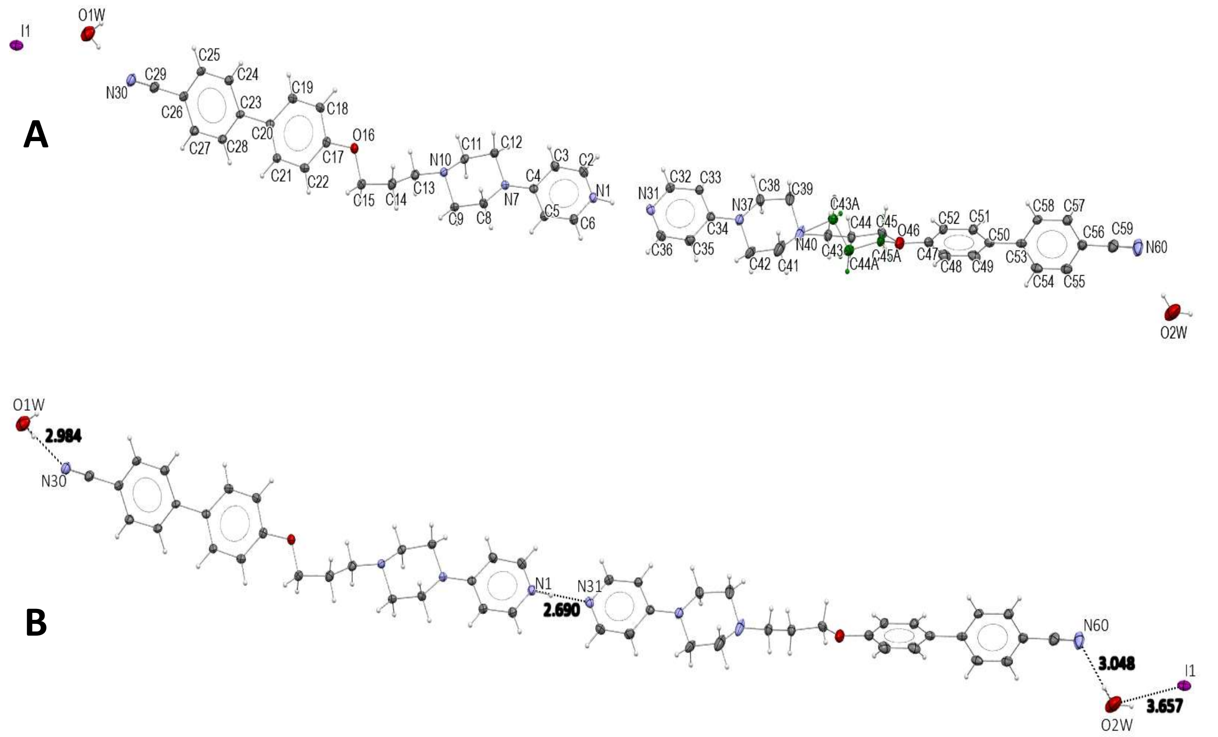

Figure S6. **A**: The asymmetric unit of **KSK94_fb** crystal structure with the atom labeling scheme. The asymmetric unit consists of two independent molecules adopting alternative conformations in the crystal lattice. For one of them (molecule 2 with label numbers 31-60) a positional disorder is observed for the propyl chain, existing in two alternative conformations (site occupancies 63% and 37%; the less abundant conformation is marked with C and H-atoms in green and labels with A letters at the end of a symbol). **B**: The strongest interactions stabilizing crystal lattice. Similarly to **KSK67_fb**, also here a free base form was expected. However, the strongest base (N1 of the pyridine ring in molecule 1) is protonated and forms an interaction with the corresponding basic center N31 in molecule 2. No charge-assisted interaction between counter-ions was observed. The iodide anion is involved in hydrogen bonds with water molecules incorporated in the crystal structure. Water molecules form additional hydrogen bonds with a nitrile nitrogen atom as an acceptor. Displacement ellipsoids of non-hydrogen atoms are drawn at the 30% probability level. H atoms are presented as small spheres with an arbitrary radius.

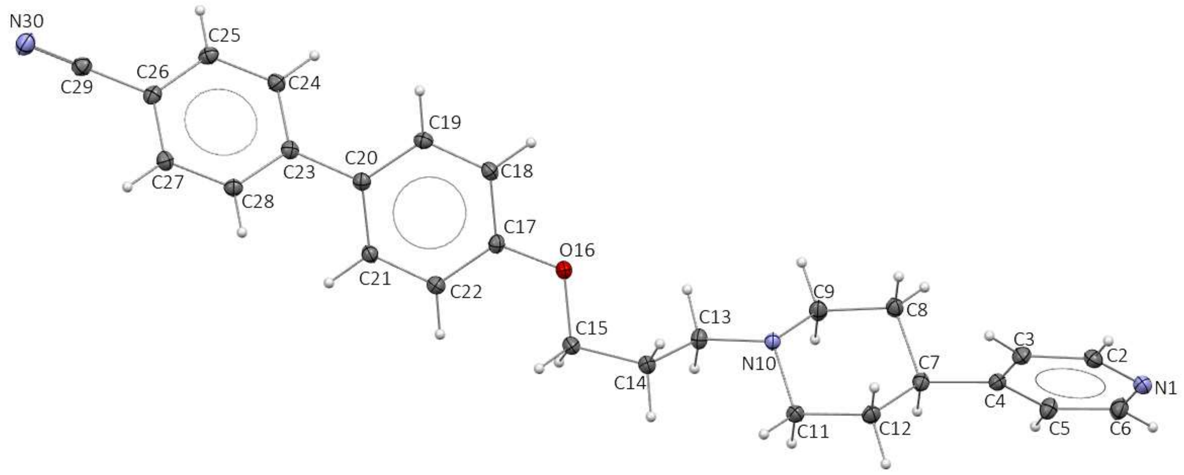

Figure S7. The asymmetric unit of **12_fb** crystal structure showing the atom labeling scheme. The crystal is stabilized only by weak interactions with the predominant number of weak hydrogen bonds with C-H donor. Displacement ellipsoids of non-hydrogen atoms are drawn at the 30% probability level. H atoms are presented as small spheres with an arbitrary radius.

2. Determination of the basicity of selected piperidine and piperazine derivatives by potentiometric titration

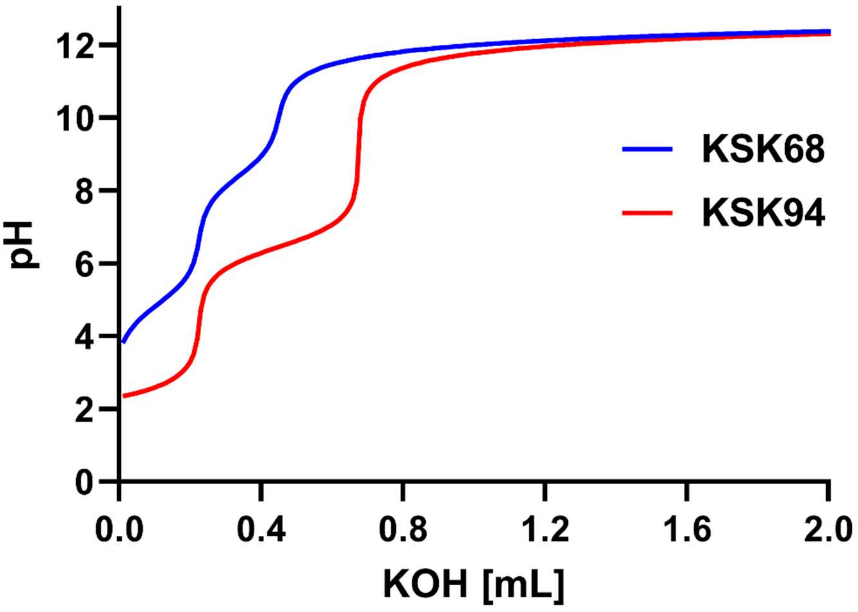

Figure S8. Titration curves of compounds **KSK68** and **KSK94** used to generate their protonation states.

3. Protonation investigation based on NMR spectroscopy measurements in pH-controlled environment

All signals in the ^{1}H and ^{13}C spectra were assigned based on 2D correlation spectra for compounds **KSK68** and **KSK94** to follow the course of changes in their chemical shifts during titration with trifluoromethanesulfonic acid (TfOH).

The spectrum of **KSK68** consists two main groups of signals: aromatic and alkyl. The signals of the methylene groups of the piperidine ring differentiate into equatorial and axial positions on the spectrum due to the different chemical environment in the ring's chair conformation. The H3 proton signal is covered by the DMSO-d_6 signal. All other signals are separable and have a distinct multiplet structure.

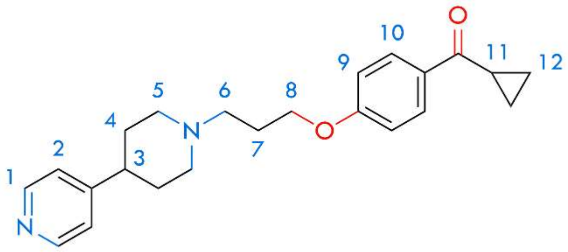

Figure S9. Structure of **KSK68** with numbered hydrogen atoms.

The addition of the first portion of TfOH in the amount of 0.2 molar equivalents does not cause any fundamental changes in the chemical shifts and multiplet structures of the signals in the aromatic part of the spectrum and for signals from H8 to H12. In contrast, the proton signals of the piperidine ring and the adjacent proton signals of H6, H7 shift toward higher frequencies. This signals undergo significant broadening and loss of multiplet structure. That behaviour can be explained to the occurrence of the dynamics of the transition of the piperidine ring from the protonated form to the free form of the nitrogen function, which entails conformational changes that are too slow on the NMR time scale. The signals of protons closer to the piperidine nitrogen undergo stronger chemical shift changes. As the titration continues, the protons H1, H1' also shift slightly towards higher frequencies. This trend is observed until a molar ratio of 1.0 : 1.0 is reached. With this acid concentration the piperidine ring proton signals and adjacent signals of H6, H7 protons recover the multiplet structure. It is associated with a shift in exchange dynamics toward the protonated form only. Further addition of acid does not cause any change in the structure and chemical shift of the aliphatic signals except for the proton H3 signal, which is the nodal proton between the pyridine and piperidine ring.

From a ratio of 1.2 : 1.0, the aromatic pyridine signals begin to shift toward higher frequencies, with shift change values for protons H1, H1' being larger than for H2, H2'. The change in the chemical shifts of the signals for the pyridine protons and H3 continues up to an acid content of 2.0 : 1.0. Above this value, there is no longer any change in the signal structure

of compound **KSK68** in the NMR spectrum. Only increasing concentrations of free triflic acid can be observed.

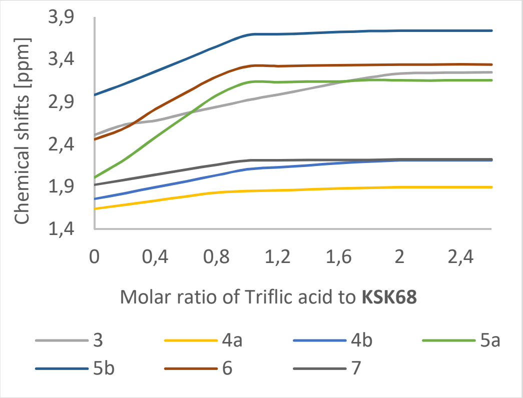

Figure S10. Proton chemical shifts in increasing molar ratio of triflic acid to **KSK68** for the significant aliphatic signals.

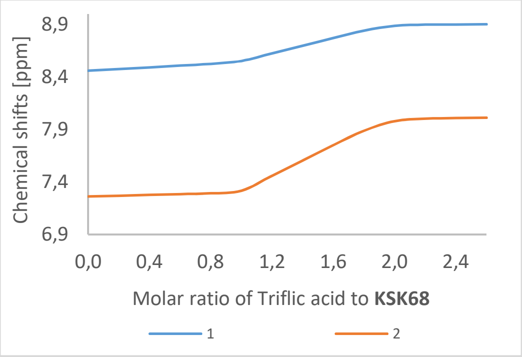

Figure S11. Proton chemical shifts in increasing molar ratio of triflic acid to **KSK68** for the significant aromatic signals.

Similar to **KSK68**, the spectrum of **KSK94** contains two major groups of signals. The aromatic part, which is enriched with two signals of an additional ring, and the aliphatic part with a simplified structure in comparison to the above case of **KSK68**. In contrast to the piperidine ring, the signals of protons of H3, H3' and H4, H4' in piperazine ring do not differentiate into equatorial and axial positions - they appear as single signals with integration referred as equal to 4. The signals of protons H4, H4' and H5 are covered by DMSO-d_6 signal.

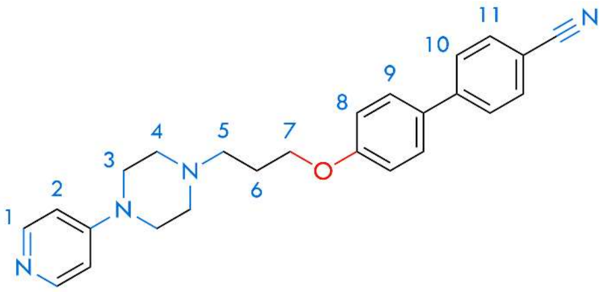

Figure S12. Structure of **KSK94** with numbered hydrogen atoms.

Titration with triflic acid to a molar ratio of 0.6 : 1.0 results in a strong shift of the aromatic signals H1, H1' towards higher frequencies. Identical behaviour is observed for H3, H3' aliphatic protons of the piperazine ring. The aromatic protons H2, H2' also shift in the same direction but with lower step difference values. The remaining aliphatic proton signals show no change in structure or chemical shift. Further titration with acid cause a decrease in the dynamics of changes in chemical shifts of aromatic protons, which still occurs but is much more subtle. On the other hand, significant changes could be observed in the aliphatic range of the spectrum. The signals of H3, H4, H5 and H6 protons have broadened and lost their multiplet structure. The situation is analogous to the case of compound **KSK68**. At a molar ratio of 1.2 : 1.0, the signals of protons H3, H3' and H4, H4' start to split into signals originating from protons in axial and equatorial positions in the piperazine ring. At this acid concentration all aliphatic signals have recovered

their multiplet structure. Above a ratio of 1:4 : 1.0, there are no more changes in the NMR spectral image except for increasing TfOH acid content.

As the titration progresses, a very broad signal associated with protonated nitrogen functions begins to grow out of the baseline in the range of 12 ppm to 14 ppm. At a molar ratio of 0.6 : 0.1 its chemical shift is about 13 ppm. Up to a ratio of 1.0 : 1.0 it shifts toward lower frequencies resulting in a very broad signal. At 1.2 : 1.0 signal splits into two independent signals at 9.86 ppm and 13.60 ppm, each one corresponds to different protonated nitrogen atom.

For compound **KSK94**, $^{1}H\{^{15}N\}$ HMBC nitrogen spectra were collected for the free base form and after the titration was completed. The chemical shift for pyridinic nitrogen before the addition of acid is -115 ppm relative to nitromethane (0 ppm). The same pyridinic nitrogen after the titration showed a chemical shift value of -209 ppm. The difference in shifts is 94 ppm.

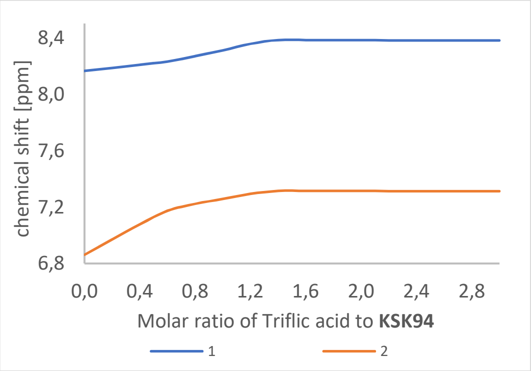

Figure S13. Proton chemical shifts in increasing molar ratio of triflic acid to **KSK94** for the significant aromatic signals.

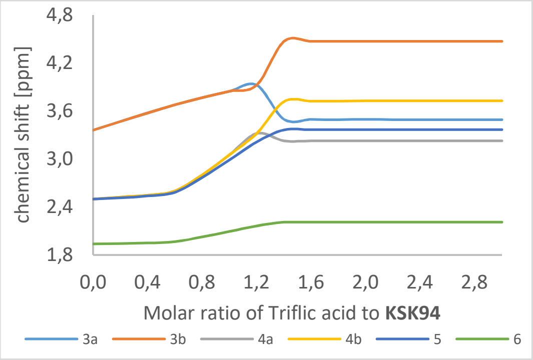

Figure S14. Proton chemical shifts in increasing molar ratio of triflic acid to **KSK94** for the significant aliphatic signals.

All spectra were measured on a JEOL JNM_ECZR 600 MHz spectrometer equipped with a Royal HFX probe. Full structural analysis with assignment of ^{1}H and ^{13}C signals performed using JASON software.

KSK68

Spectra and structural analysis

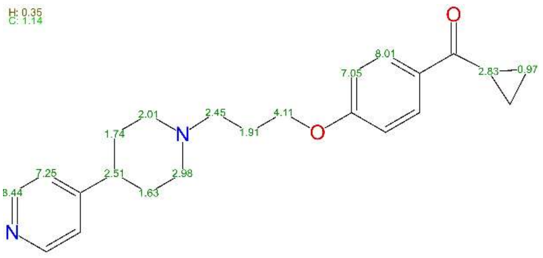

Figure S15. Structure of **KSK68** with ^{1}H chemical shifts applied on.

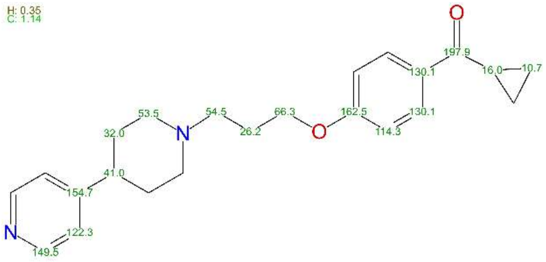

Figure S16. Structure of **KSK68** with ^{13}C chemical shifts applied on.

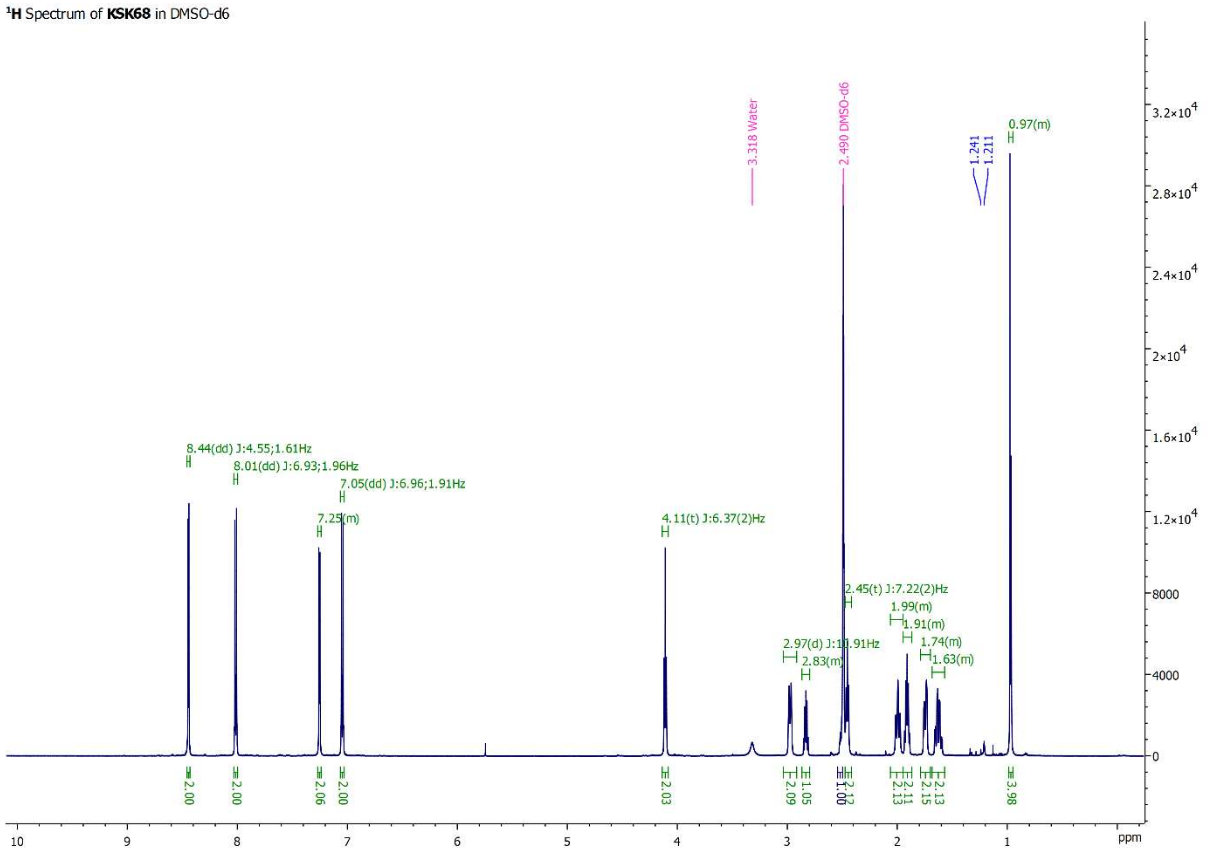

^{13}C Spectrum of **KSK68** in DMSO-d6

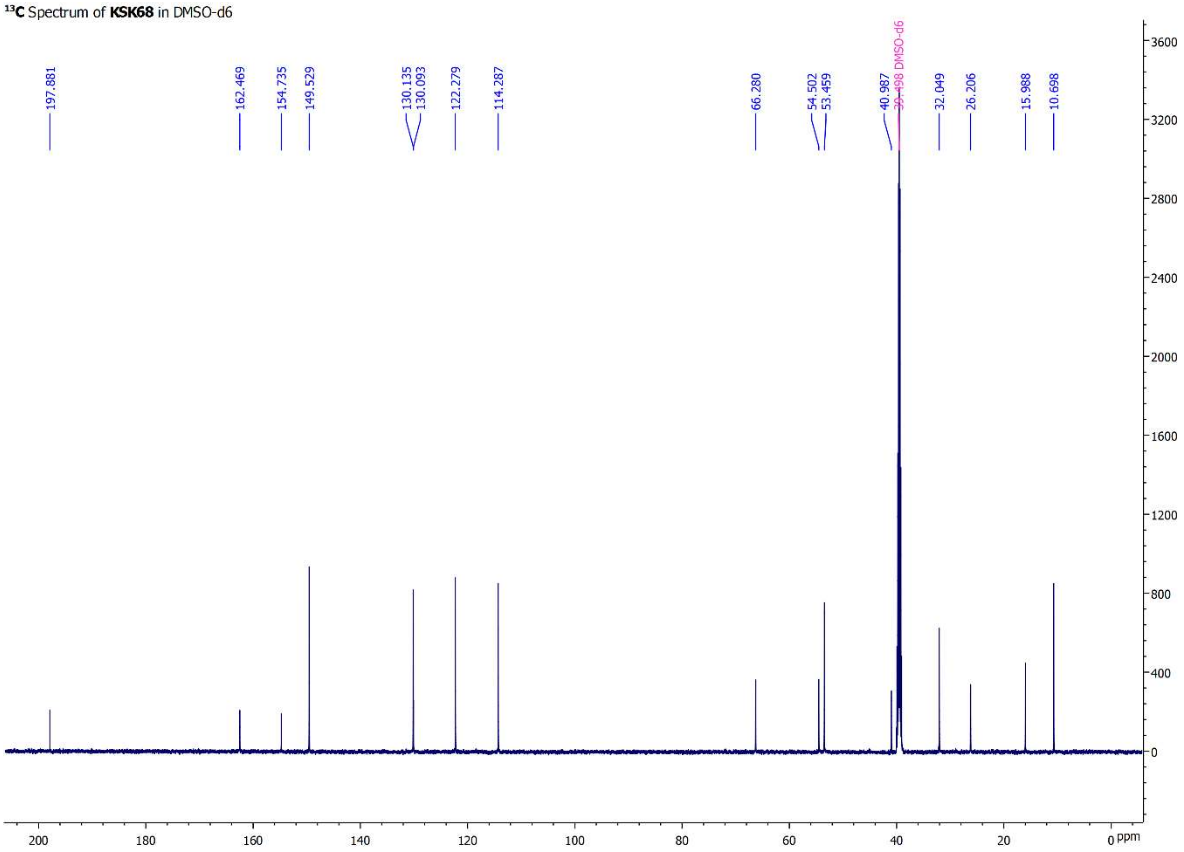

DQF-COSY Spectrum of **KSK68** in DMSO-d6

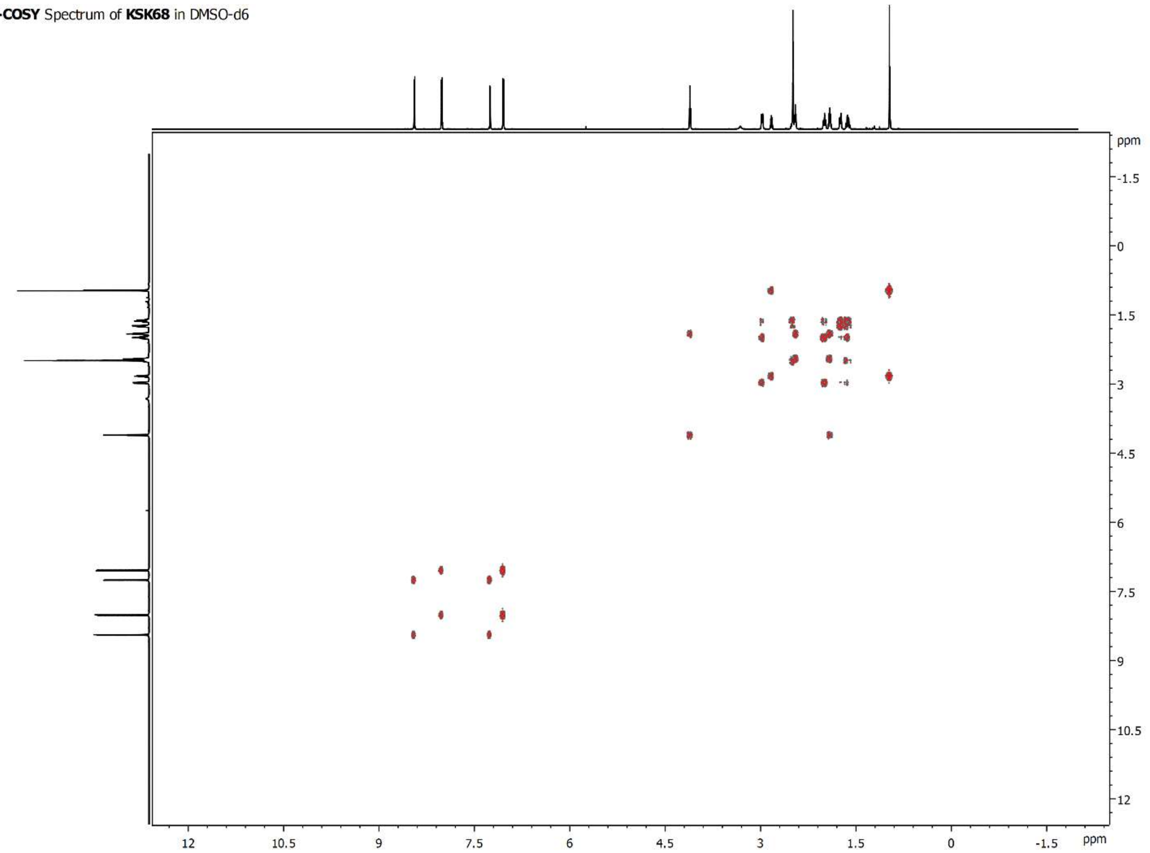

HSQC Spectrum of **KSK68** in DMSO-d6

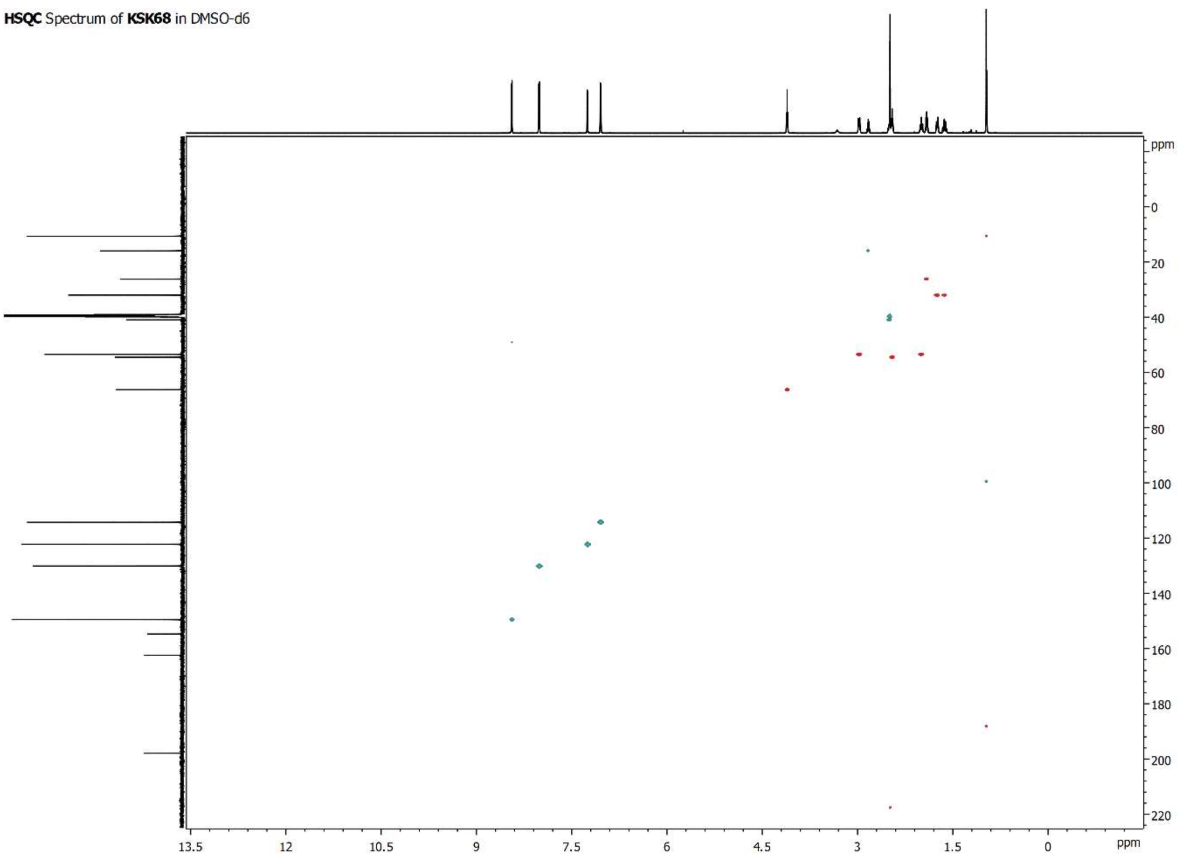

HMBC Spectrum of **KSK68** in DMSO-d6

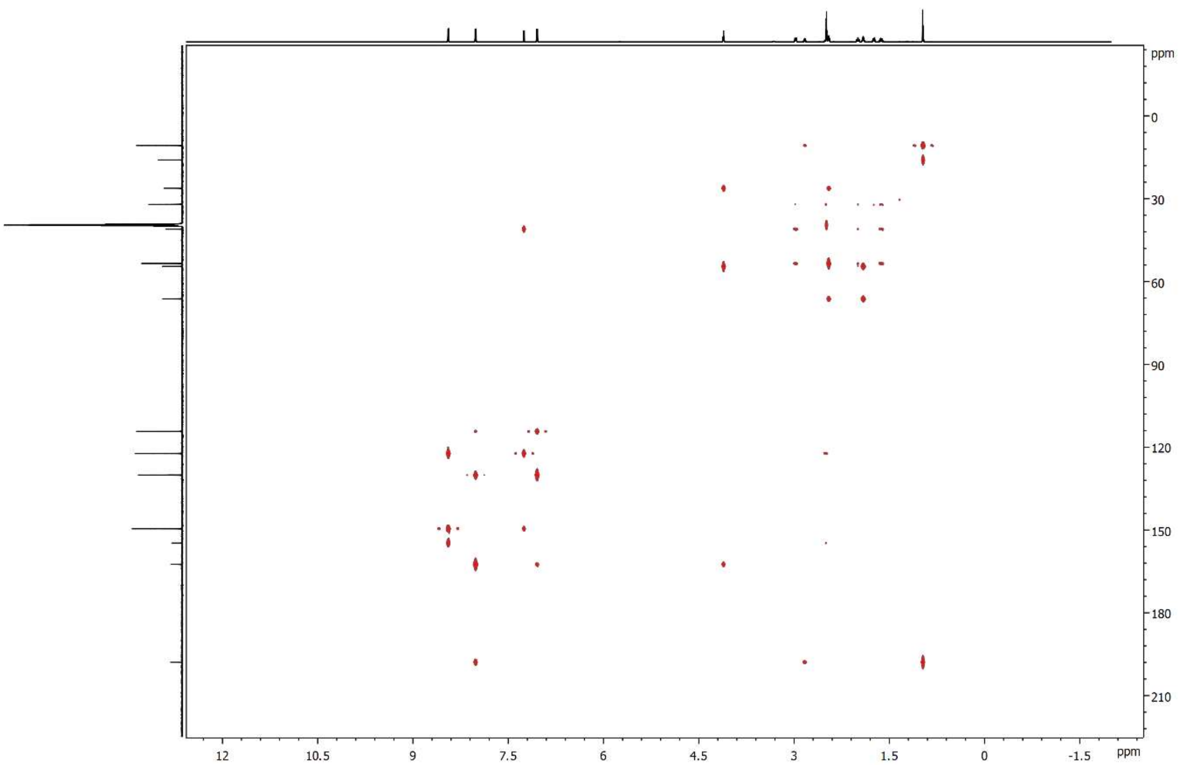

KSK68

Titration experiment

Table S3. Proton chemical shifts of signals of compound **KSK68** with increasing TfOH concentration during titration.

	Chemical shifts [ppm] in reference to TMS in DMSO-d_6													
**Atom label**	1	2	3	4a	4b	5a	5b	6	7	8	9	10	11	12
**Molarity of acid**														
0.0	8.462	7.264	2.513	1.642	1.760	2.011	2.985	2.460	1.927	4.127	7.061	8.030	2.841	0.994
0.2	8.478	7.272	2.634	1.690	1.827	2.226	3.117	2.595	1.985	4.143	7.068	8.038	2.845	0.997
0.4	8.495	7.280	2.680	1.739	1.898	2.487	3.261	2.817	2.045	4.160	7.075	8.045	2.849	1.001
0.6	8.512	7.288	2.766	1.789	1.967	2.737	3.407	3.012	2.106	4.176	7.082	8.053	2.854	1.003
0.8	8.528	7.295	2.841	1.835	2.039	2.979	3.554	3.200	2.164	4.192	7.089	8.061	2.858	1.005
1.0	8.556	7.321	2.920	1.852	2.108	3.128	3.686	3.318	2.213	4.204	7.094	8.066	2.861	1.007
1.2	8.629	7.463	2.987	1.860	2.132	3.133	3.698	3.323	2.215	4.205	7.094	8.066	2.861	1.007
1.4	8.702	7.608	3.055	1.871	2.155	3.139	3.709	3.328	2.218	4.206	7.094	8.066	2.861	1.006
1.6	8.775	7.754	3.127	1.882	2.180	3.141	3.723	3.333	2.219	4.207	7.095	8.066	2.861	1.006
1.8	8.845	7.892	3.188	1.891	2.199	3.159	3.734	3.338	2.219	4.208	7.095	8.065	2.861	1.006
2.0	8.890	7.983	3.234	1.897	2.213	3.155	3.739	3.341	2.227	4.209	7.095	8.065	2.861	1.006
2.2	8.901	8.006	3.242	1.900	2.215	3.153	3.741	3.341	2.227	4.208	7.095	8.065	2.861	1.006
2.4	8.903	8.011	3.245	1.900	2.216	3.155	3.741	3.342	2.226	4.208	7.095	8.065	2.861	1.005
2.6	8.904	8.014	3.247	1.900	2.216	3.155	3.741	3.341	2.225	4.208	7.095	8.064	2.861	1.005

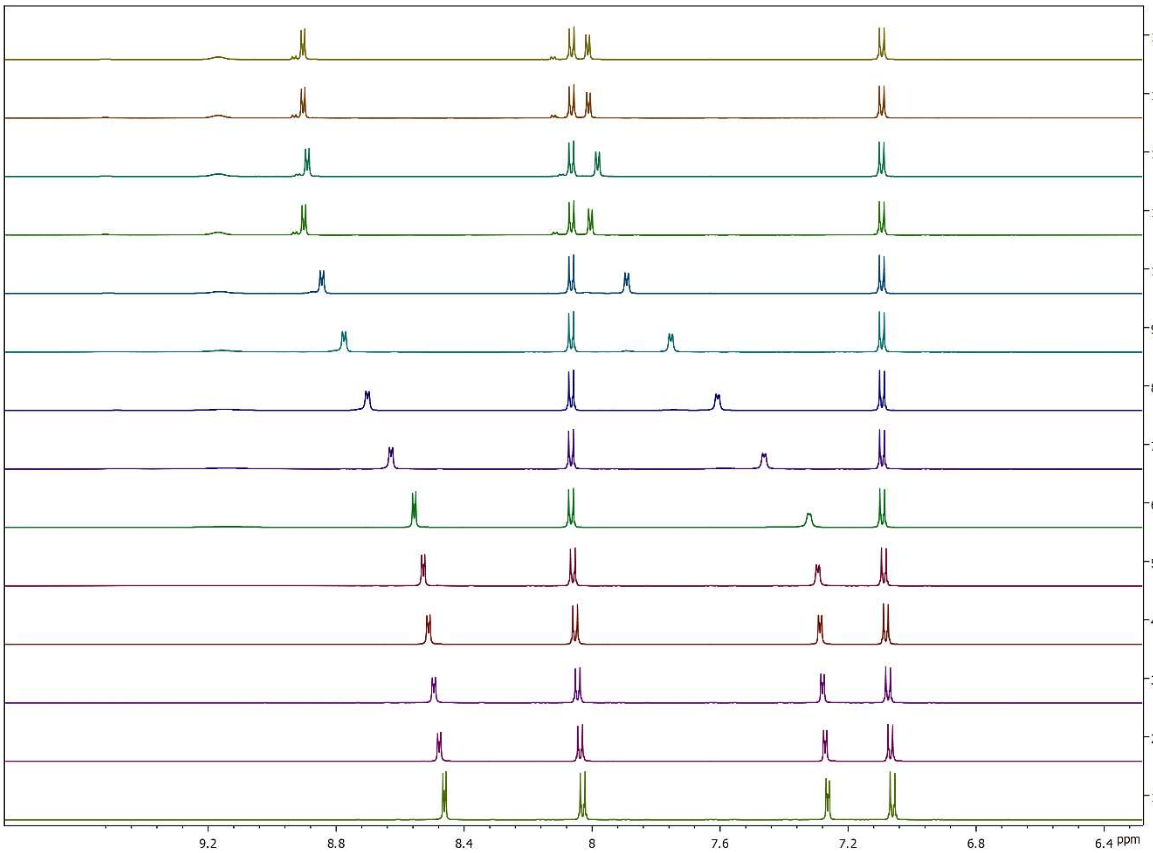

Figure S17. Stacked ^{1}H spectra of **KSK68** with increasing TfOH concentration. Aromatic region of spectrum. Acid concentration is increasing from the bottom spectrum (no acid) in steps of molar equivalents equal to 0.2.

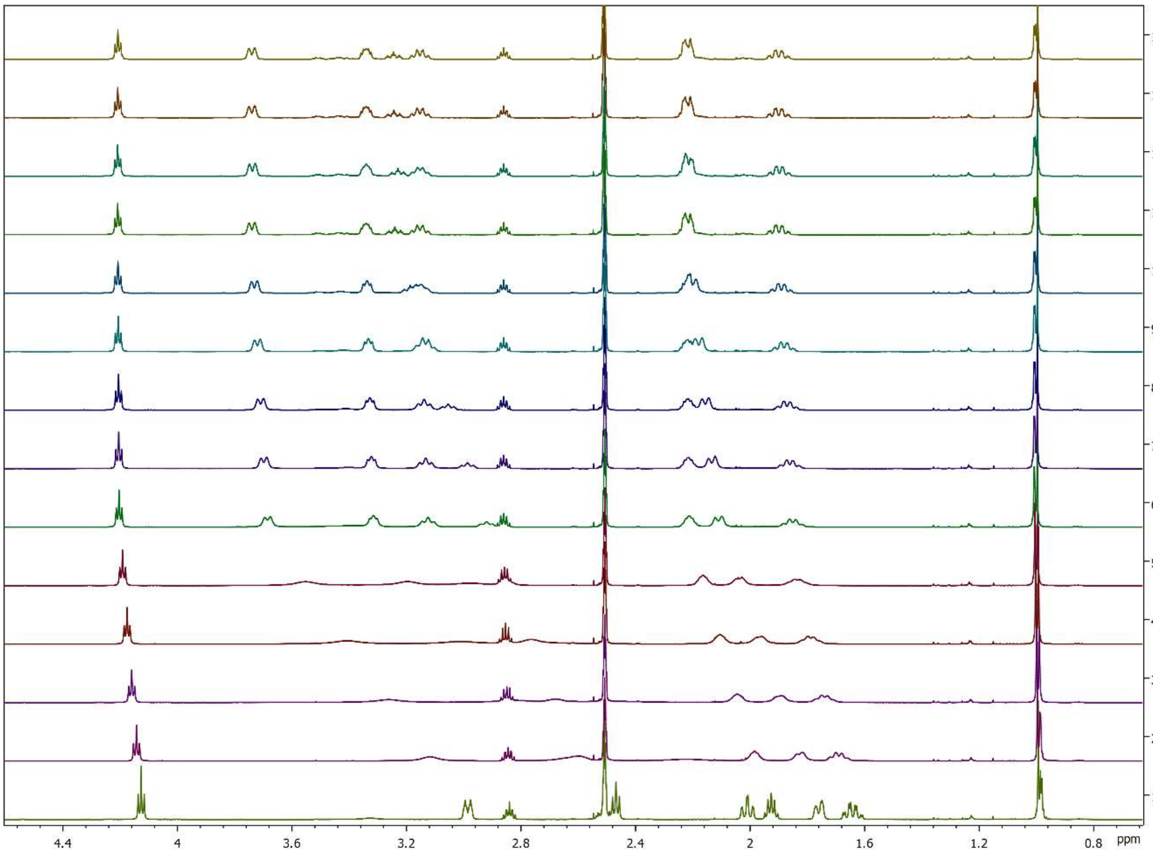

Figure S18. Stacked ^{1}H spectra of **KSK68** with increasing TfOH concentration. Aliphatic region of spectrum. Acid concentration is increasing from the bottom spectrum (no acid) in steps of molar equivalents equal to 0.2.

KSK94

Spectra and structural analysis

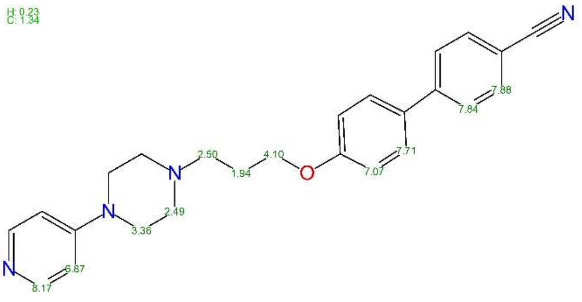

Figure S19. Structure of **KSK94** with ^{1}H chemical shifts applied on.

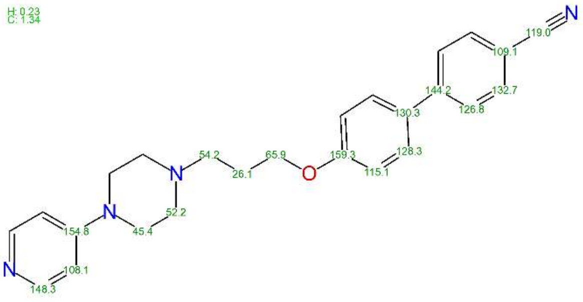

Figure S20. Structure of **KSK94** with ^{13}C chemical shifts applied on.

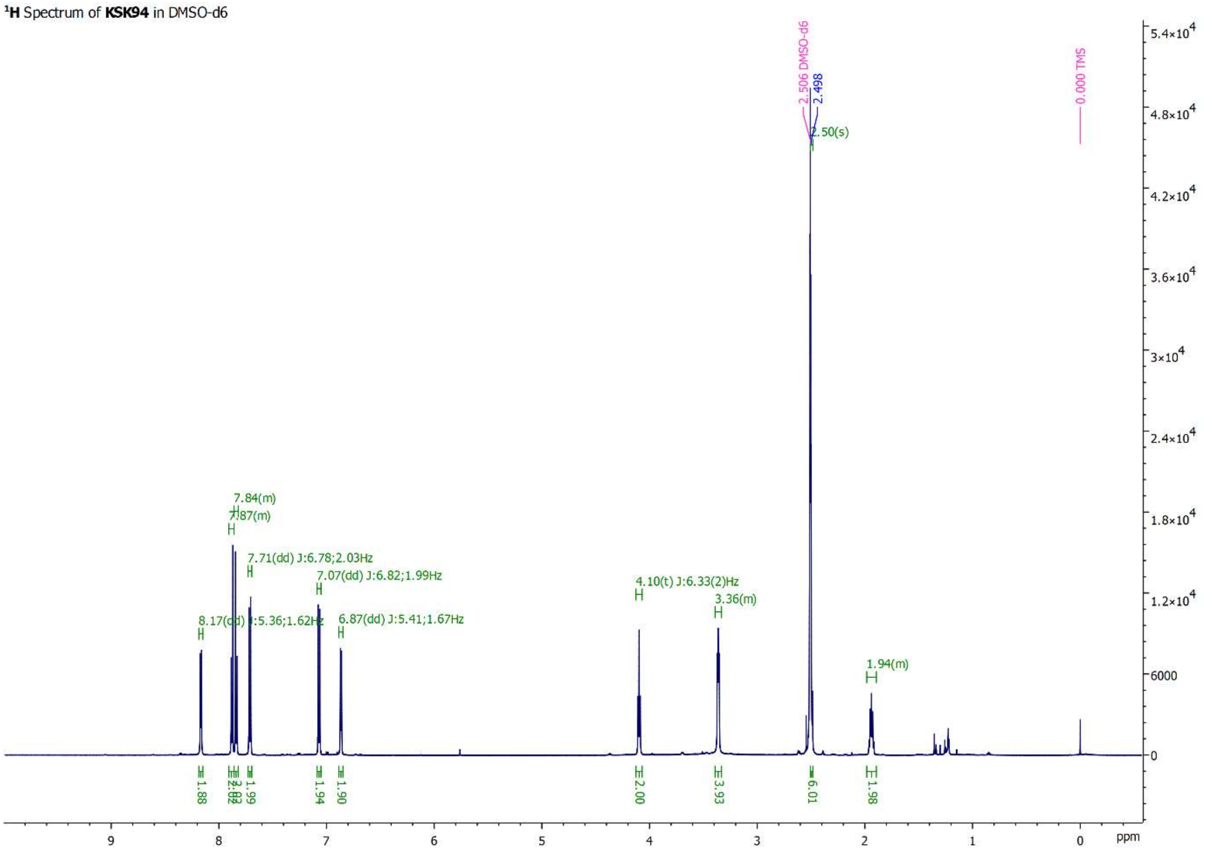

¹³C Spectrum of **KSK94** in DMSO-d6

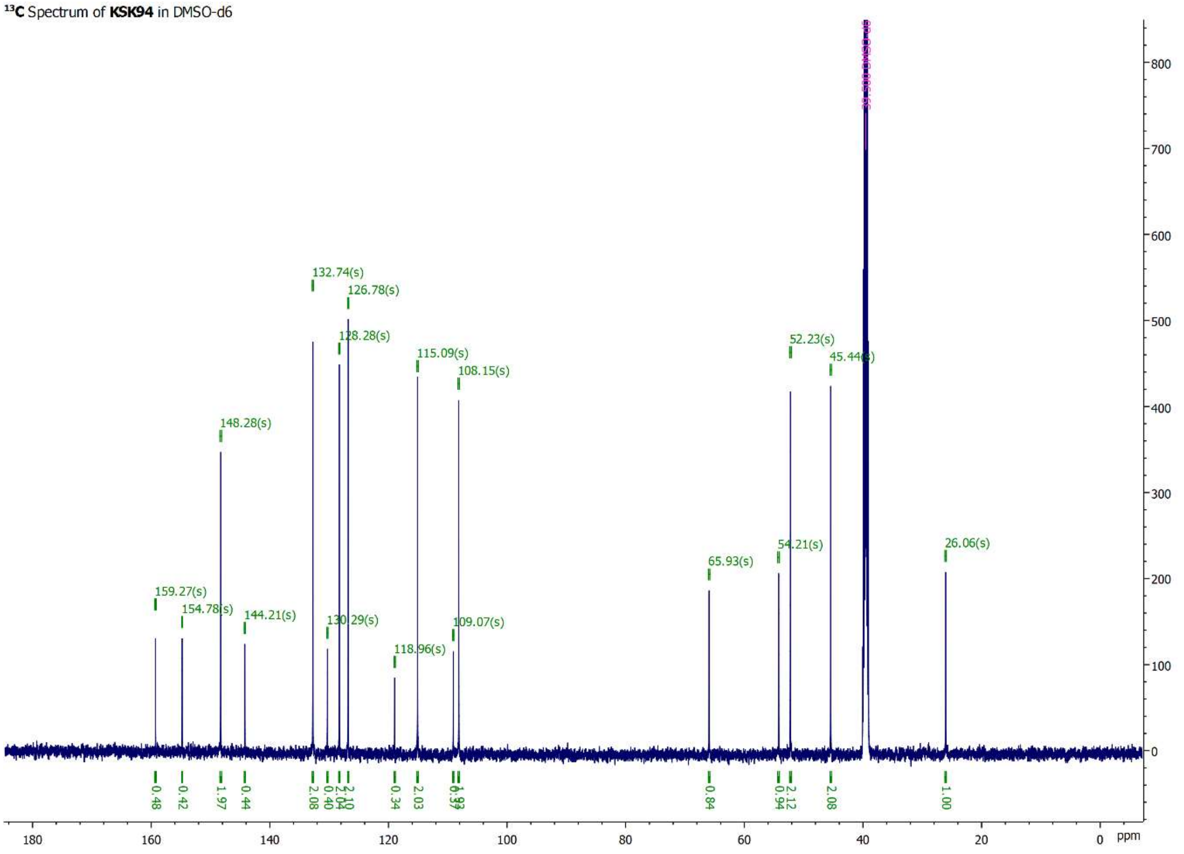

¹H-¹H DQF-COSY Spectrum of **KSK94** in DMSO-d6

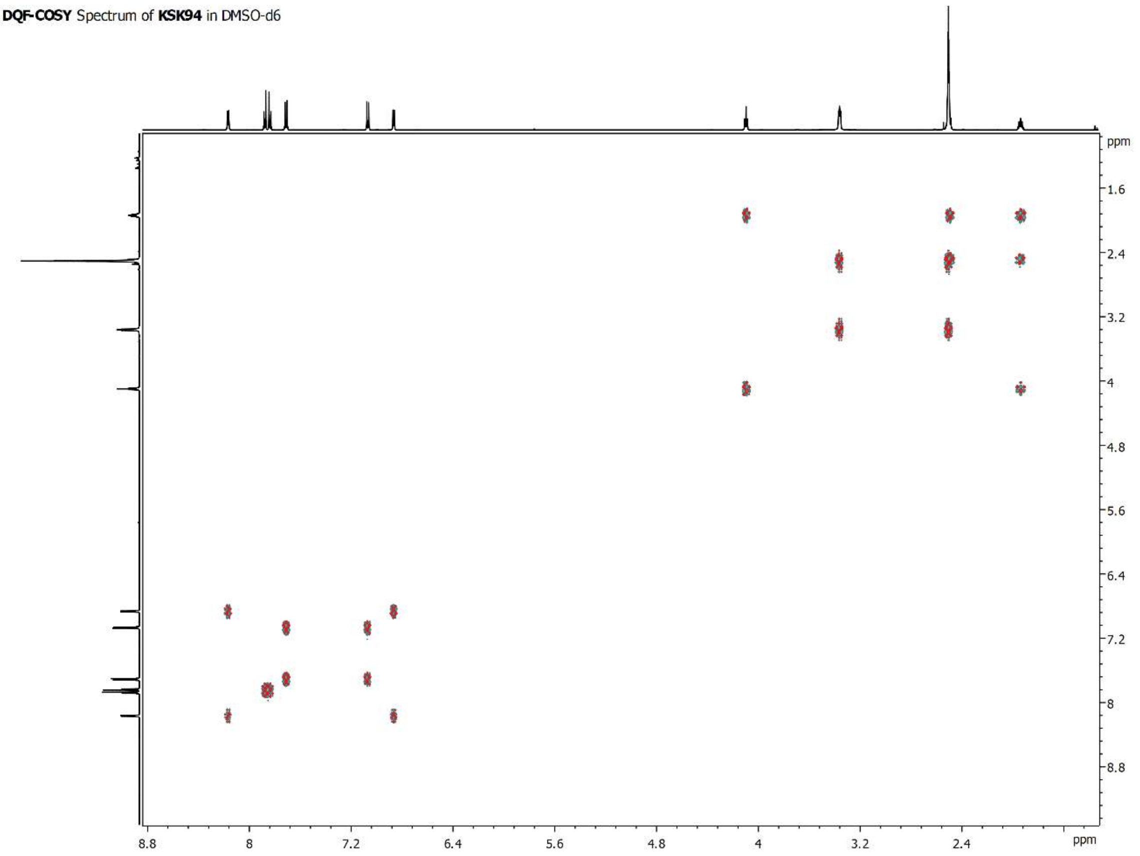

^{13}**C-HSQC** Spectrum of **KSK94** in DMSO-d6

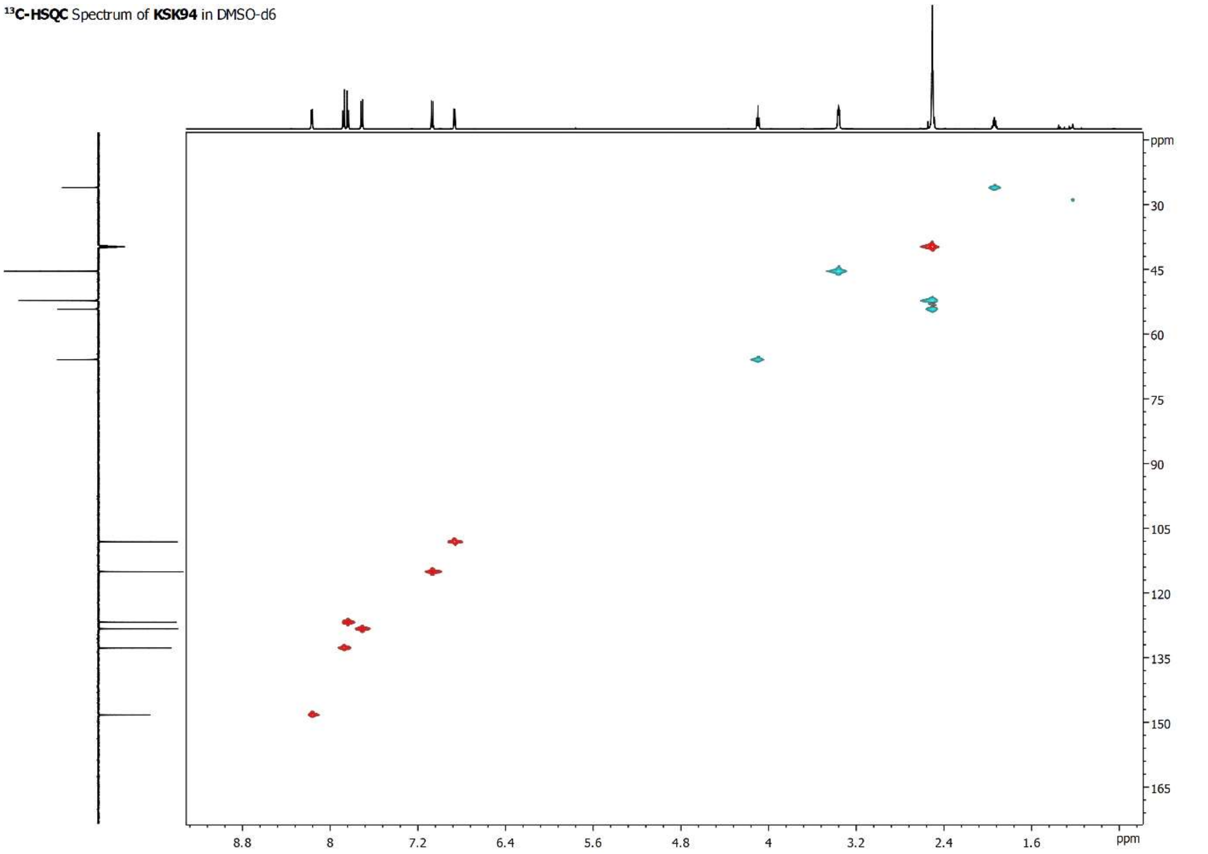

^{13}**C-HMBC** Spectrum of **KSK94** in DMSO-d6

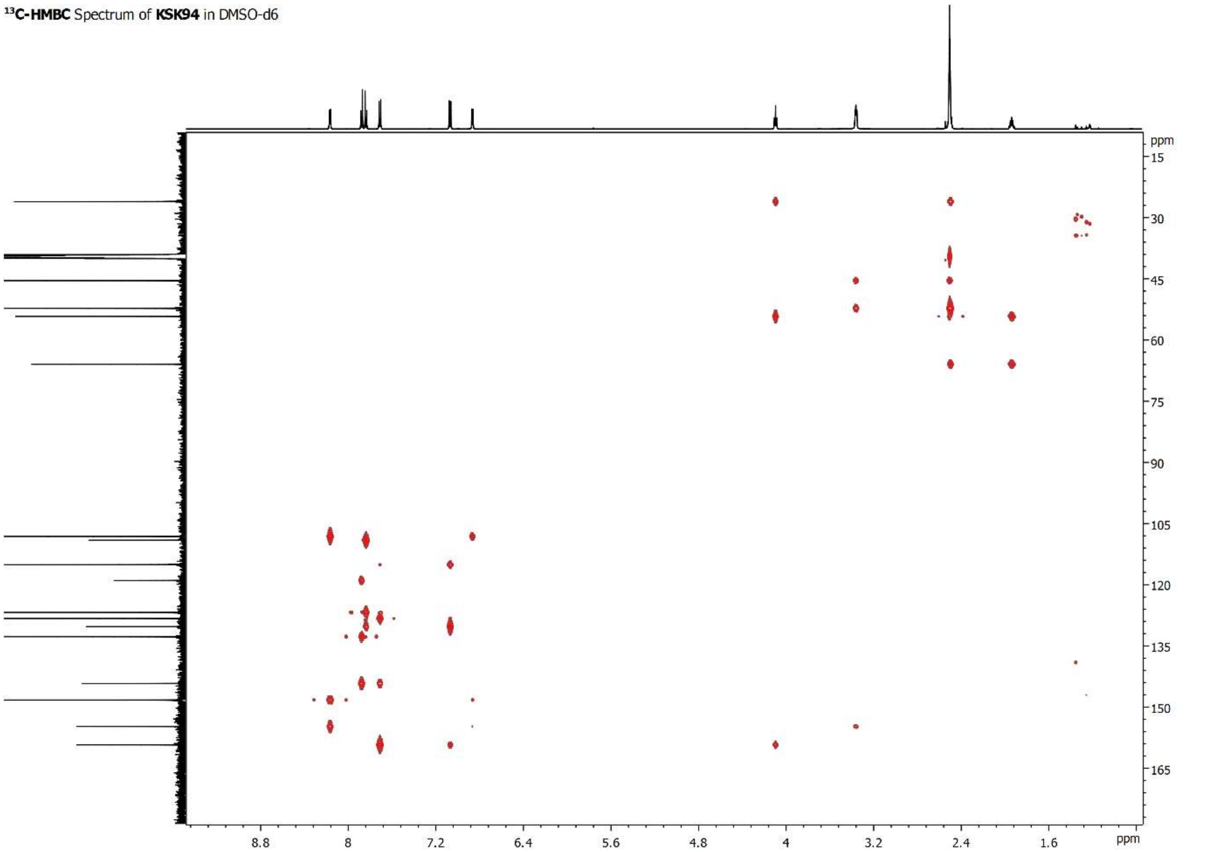

^{15}N HMQC Spectrum of **KSK94** in DMSO-d6
+ **0,0** molar equivalent of Triflic acid (**TrifA**)

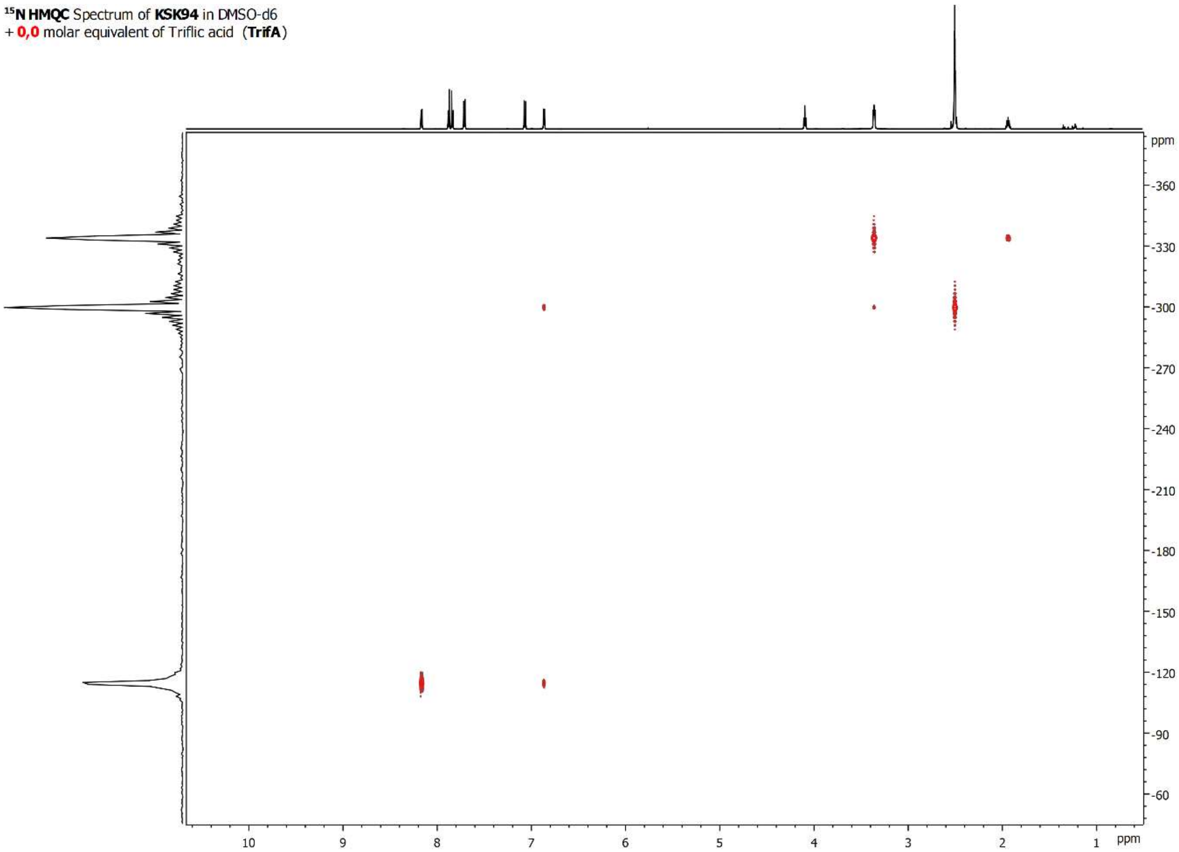

^{15}N HMQC Spectrum of **KSK94** in DMSO-d6
+ **3,0** molar equivalent of Triflic acid (**TrifA**)

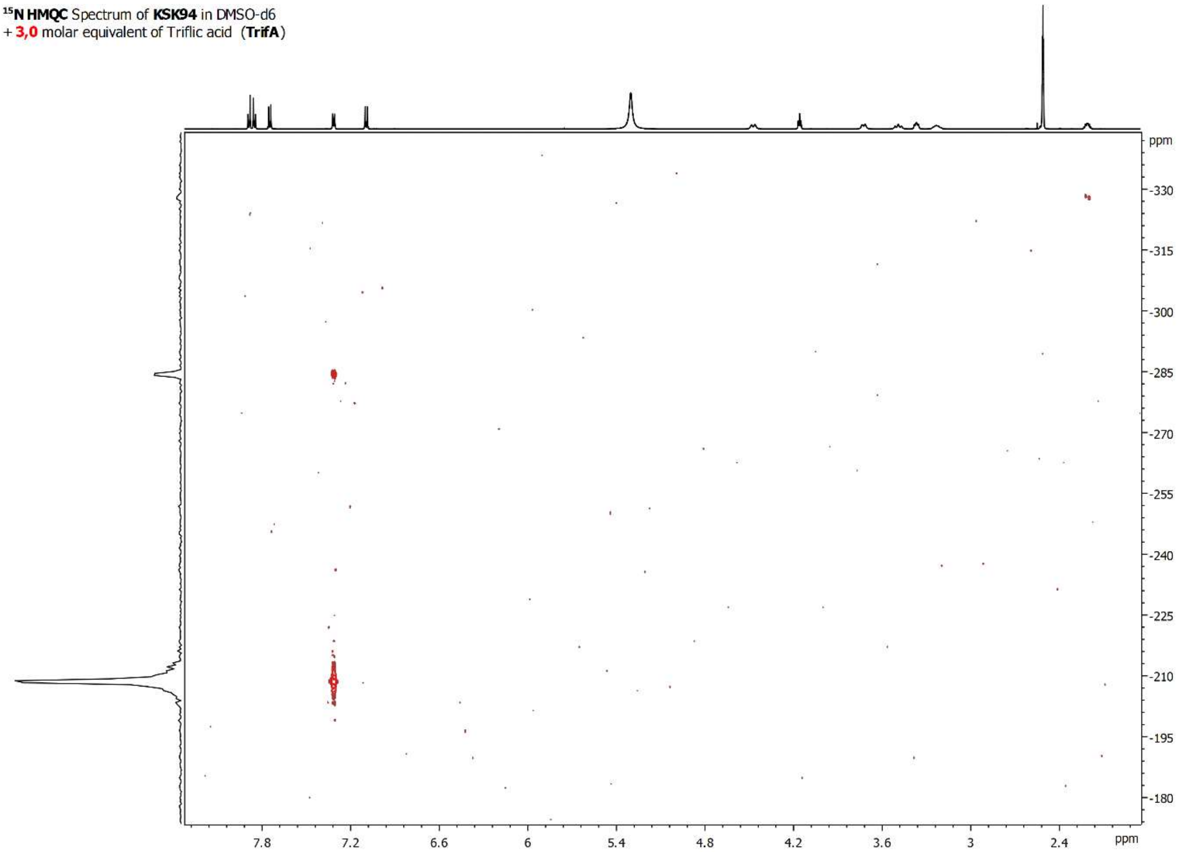

KSK94

Titration experiment

Table S4. Proton chemical shifts of signals of compound **KSK94** with increasing TfOH concentration during titration.

	Chemical shifts [ppm] in reference to TMS in DMSO-d_6												
**Atom label**	1	2	3a	3b	4a	4b	5	6	7	8	9	10	11
**Molarity of acid**													
0.0	8.168	6.865	3.362	3.362	2.500	2.500	2.500	1.939	4.097	7.069	7.711	7.837	7.877
0.2	8.190	6.975	3.470	3.470	2.527	2.527	2.517	1.945	4.101	7.070	7.714	7.839	7.881
0.4	8.211	7.081	3.577	3.577	2.551	2.551	2.540	1.952	4.106	7.070	7.712	7.837	7.876
0.6	8.235	7.177	3.680	3.680	2.604	2.604	2.587	1.969	4.112	7.072	7.715	7.839	7.878
0.8	8.272	7.226	3.769	3.769	2.805	2.805	2.771	2.028	4.124	7.077	7.722	7.841	7.879
1.0	8.313	7.261	3.849	3.849	3.055	3.055	2.990	2.096	4.139	7.084	7.732	7.844	7.882
1.2	8.359	7.297	3.927	3.927	3.323	3.323	3.215	2.166	4.152	7.091	7.745	7.851	7.890
1.4	8.386	7.318	3.498	4.474	3.231	3.720	3.365	2.212	4.161	7.095	7.750	7.853	7.892
1.6	8.386	7.318	3.496	4.475	3.230	3.725	3.367	2.213	4.161	7.095	7.750	7.852	7.891
1.8	8.385	7.318	3.493	4.475	3.232	3.727	3.368	2.212	4.161	7.095	7.750	7.853	7.891
2.0	8.385	7.318	3.497	4.475	3.232	3.728	3.368	2.213	4.161	7.096	7.749	7.854	7.892
2.2	8.383	7.316	3.494	4.475	3.232	3.728	3.368	2.214	4.160	7.095	7.750	7.854	7.892
2.4	8.383	7.316	3.494	4.475	3.232	3.728	3.368	2.214	4.160	7.095	7.750	7.854	7.892
2.6	8.383	7.316	3.494	4.475	3.232	3.728	3.368	2.214	4.160	7.095	7.750	7.854	7.892
2.8	8.383	7.316	3.494	4.475	3.232	3.728	3.368	2.214	4.160	7.095	7.750	7.854	7.892
3.0	8.383	7.316	3.494	4.475	3.232	3.728	3.368	2.214	4.160	7.095	7.750	7.854	7.892

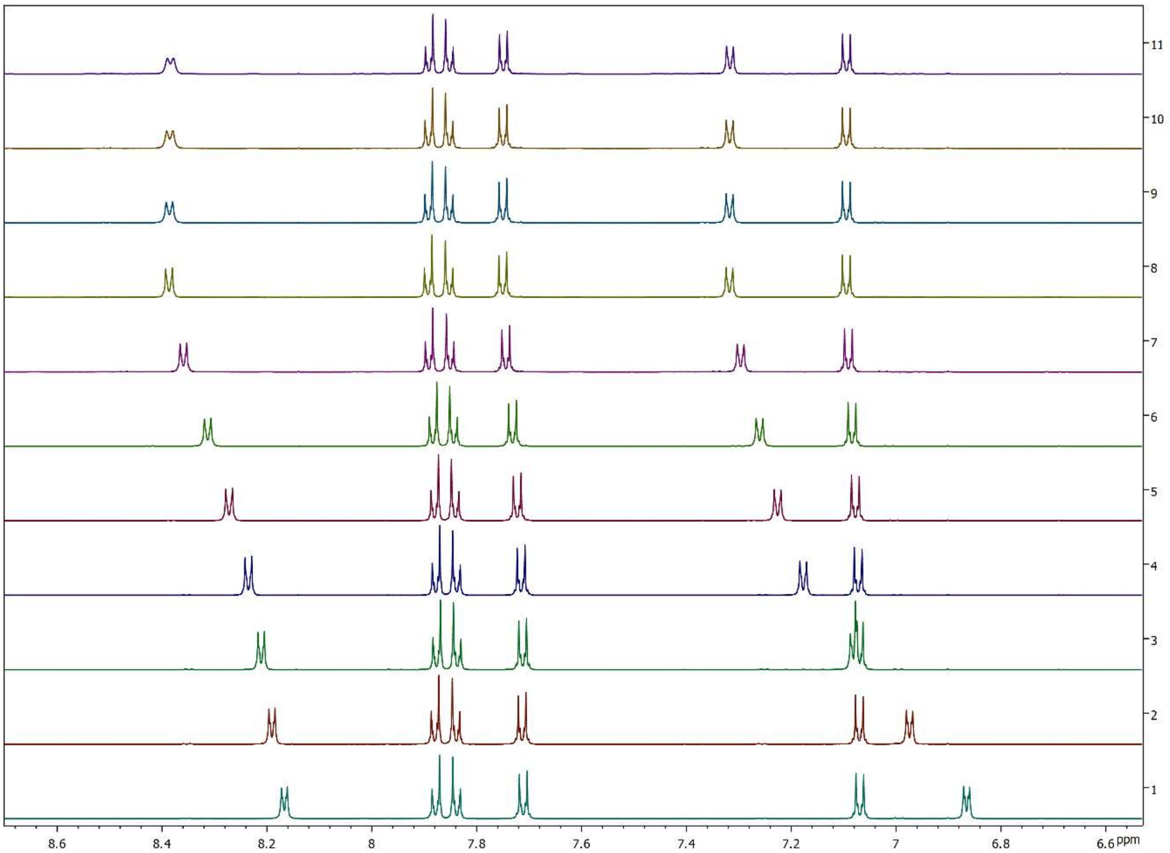

Figure S21. Stacked ^{1}H spectra of **KSK94** with increasing TfOH concentration. Aromatic region of spectrum. Acid concentration is increasing from the bottom spectrum (no acid) in steps of molar equivalents equal to 0.2.

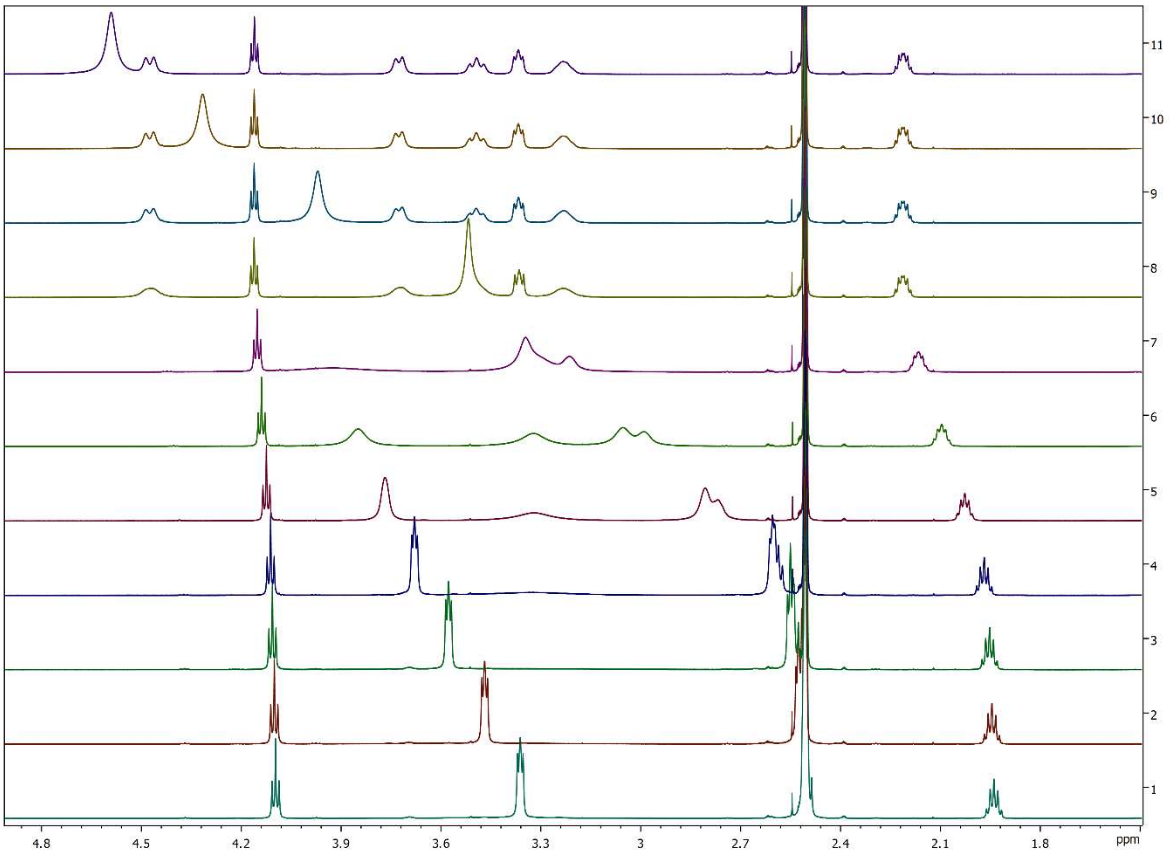

Figure S22. Stacked ^{1}H spectra of **KSK94** with increasing TfOH concentration. Aliphatic region of spectrum. Acid concentration is increasing from the bottom spectrum (no acid) in steps of molar equivalents equal to 0.2.

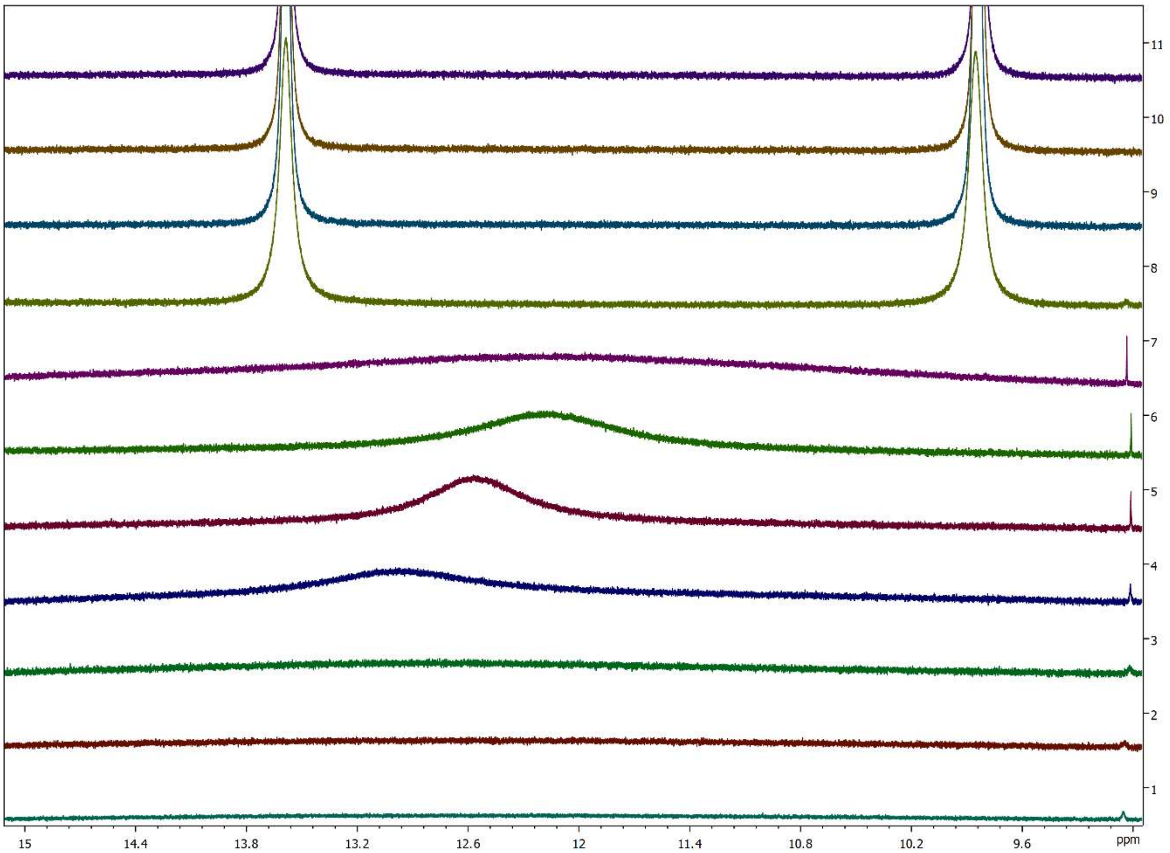

Figure S23. Stacked ^{1}H spectra of **KSK94** with increasing **TfOH** concentration. High frequency region of spectrum. Acid concentration is increasing from the bottom spectrum (no acid) in steps of molar equivalents equal to 0.2.

4. *In silico* studies. Molecular modeling: docking studies and molecular dynamics simulations

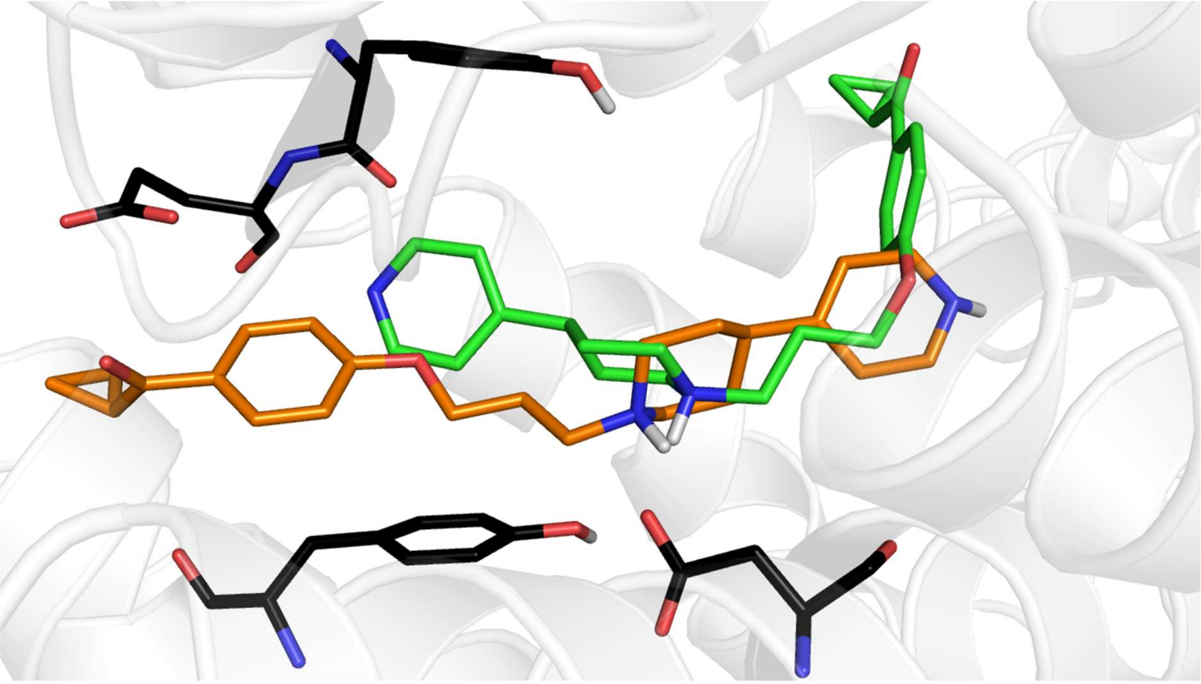

Figure S24. Comparison of the previously described **KSK68** binding mode (orange) and that obtained with induced-fit docking (green) at the H_3R binding site (homology model).

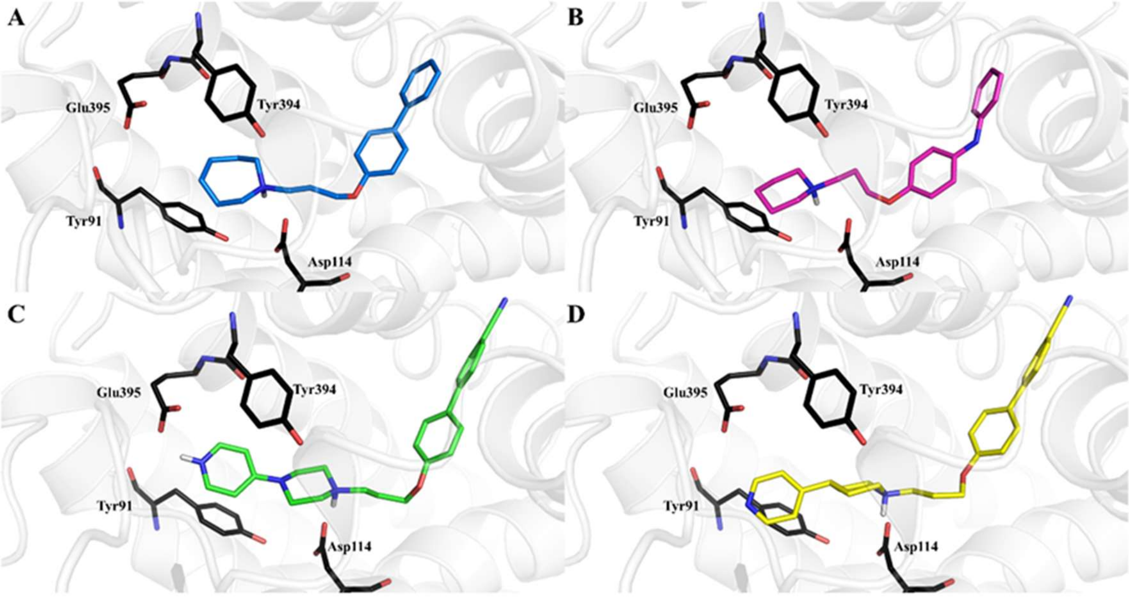

Figure S25. The binding mode of **3** (navy) (**A**), **7** (magenta) (**B**), **KSK94** (green) (**C**) in tautomeric monoprotonated state and **12** (yellow) (**D**) in the σ_1R (PDB ID: 6DJZ).

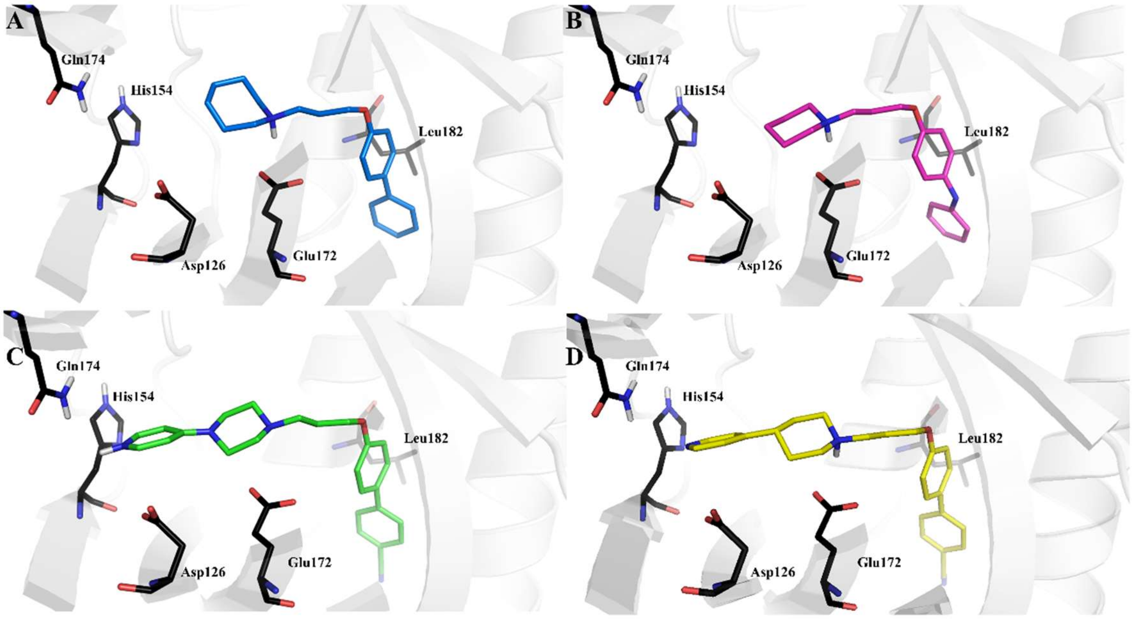

Figure S26. The binding mode of **3** (navy) (**A**), **7** (magenta) (**B**), **KSK94** (green) (**C**) in tautomeric monoprotonated state and **12** (yellow) (**D**) in H_3R binding site (homology model).

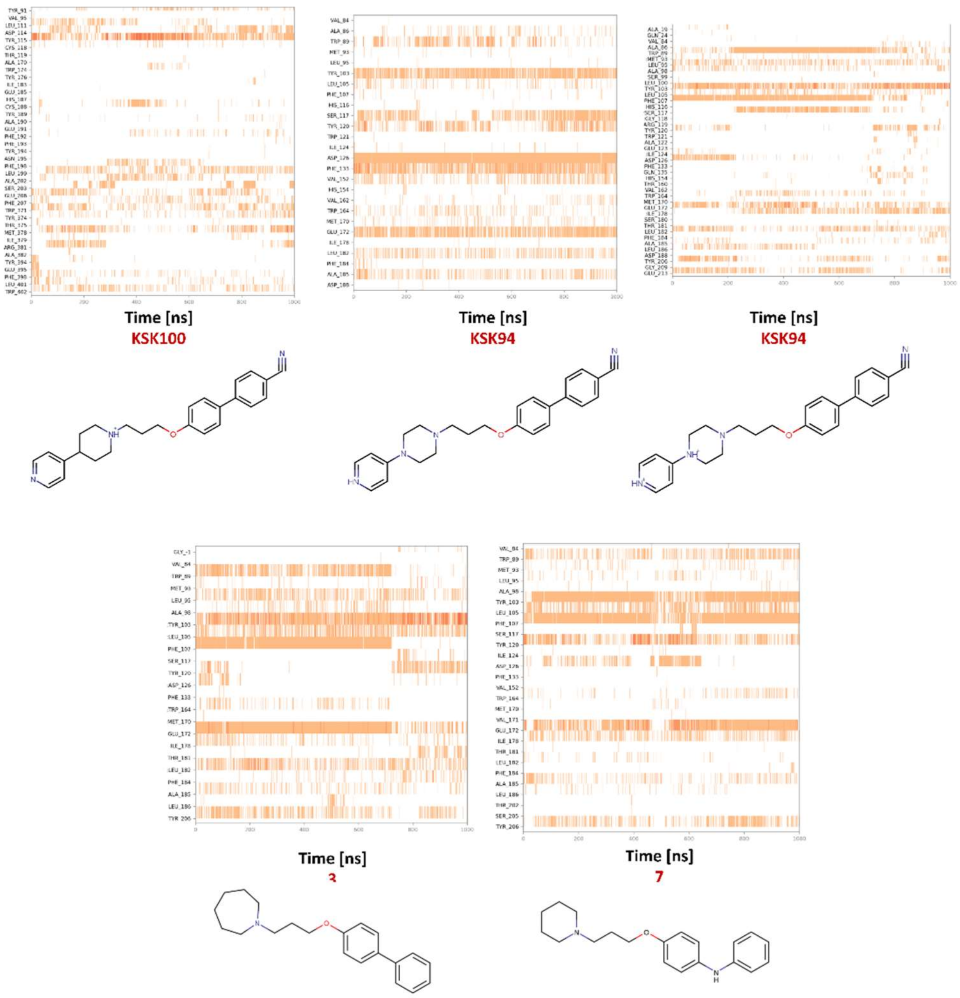

Figure S27. Ligand-protein interaction diagrams obtained during MD simulations studies for selected compounds in different protonation states docked to the σ_1R (PDB ID: 6DJZ).

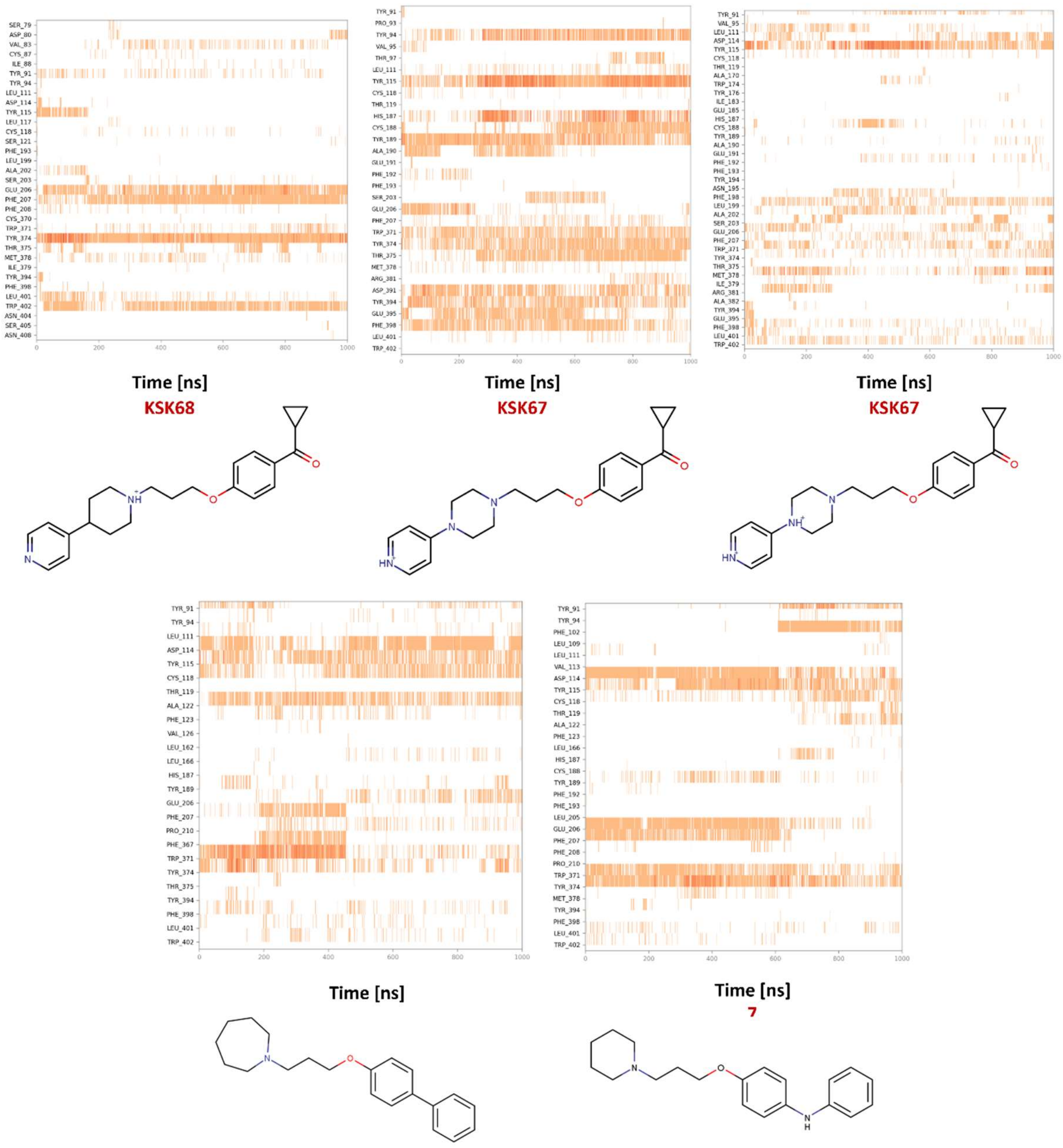

Figure S28. Ligand-protein interaction diagrams obtained during MD simulations studies for selected compounds in different protonation states docked to the H_3R binding site (homology model).

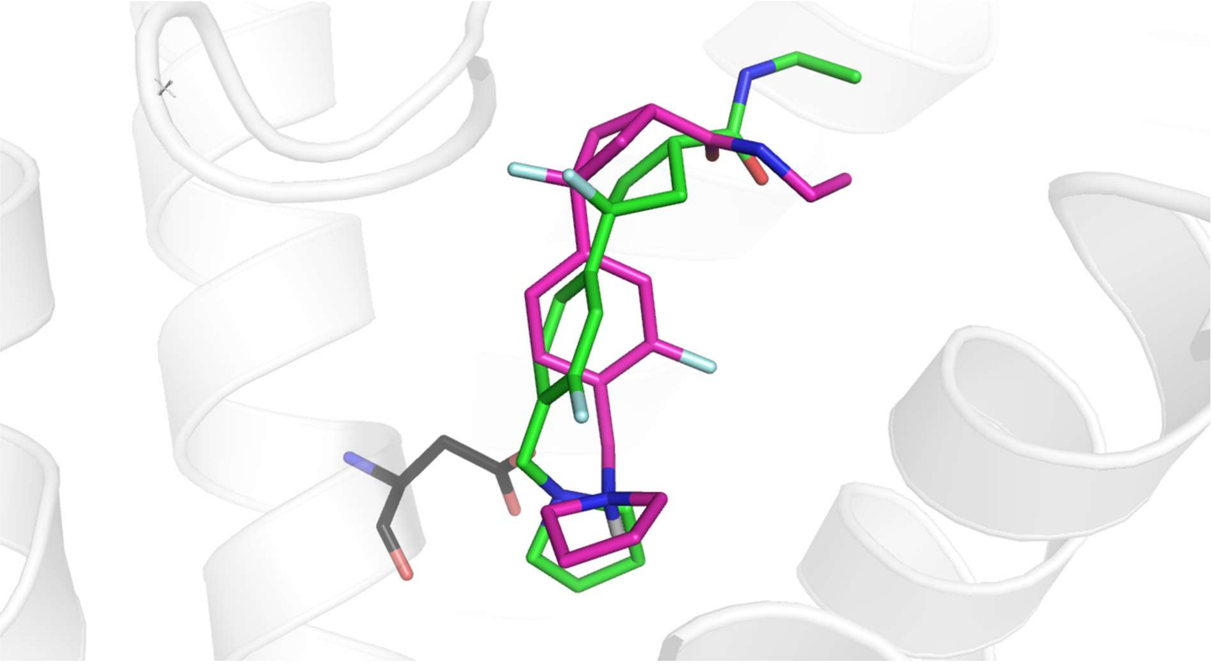

Figure S29. Superposition of the crystalized PF-03654746 (green) in crystal structure of H_3R (PDB ID: 7F61) with the binding mode (magenta) obtained with induced-fit docking to the described homology model.

Table S5. Comparison of binding free energies and docking scores (for all protonation states) with *in vitro* activity of **KSK67**, **KSK68**, **KSK94** and **12** with σ_1 and H_3 receptors. The calculated percentage of particular protonation state were published in our previous studies[1]. For the mono- and diprotonated tautomeric form of **KSK94** we couldn't estimate percentage of protonation state at physiological pH.

Form	Calc. % of protonation state	Compound	σ_1R			H_3R			Compound	σ_1R			H_3R		
			K_i [nM]	Docking score	ΔG [kcal/mol]	K_i [nM]	Docking score	ΔG [kcal/mol]		K_i [nM]	Docking score	ΔG [kcal/mol]	K_i [nM]	Docking score	ΔG [kcal/mol]
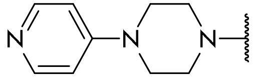	2.08			-7.56	-34.23		-8.63	-57.97			-9.32	-71.32		-9.64	-71.2
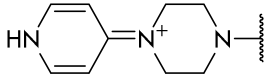		KSK67	1531	-8.04	-61.24	3.2	-9.67	-98.11	KSK94	2958	-10.45	-81.44	7.9	-10.22	-95.71
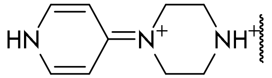				-10.53	-78.88		-10.71	-96.25			-12.52	-113.23		-10.58	-99.18
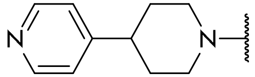	3.73			-7.66	-23.72		-8.65	-53.11			-9.31	-73.15		-9.58	-73.57
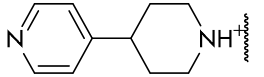	95.69	KSK68	3.6	-9.56	-99.29	7.7	-10.79	-98.69	12	4.5	-11.514	-99.47	7.7	-10.47	-97.64
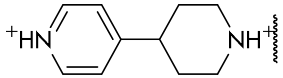	0.52			-10.278	-99.75		-11.08	-109.89			-12.23	-111.48		-10.94	-101.99

[1]Szczepańska K, Podlewska S, Dichiara M, Gentile D, Patamia V, Rosier N, Mönnich D, Ruiz Cantero MC, Karcz T, Łażewska D, Siwek A, Pockes S, Cobos EJ, Marrazzo A, Stark H, Rescifina A, Bojarski AJ, Amata E, Kieć-Kononowicz K. Structural and Molecular Insight into Piperazine and Piperidine Derivatives as Histamine H_3 and Sigma-1 Receptor Antagonists with Promising Antinociceptive Properties. *ACS Chem Neurosci*. 2022;13(1):1-15.

5. *In vivo* pharmacological activity

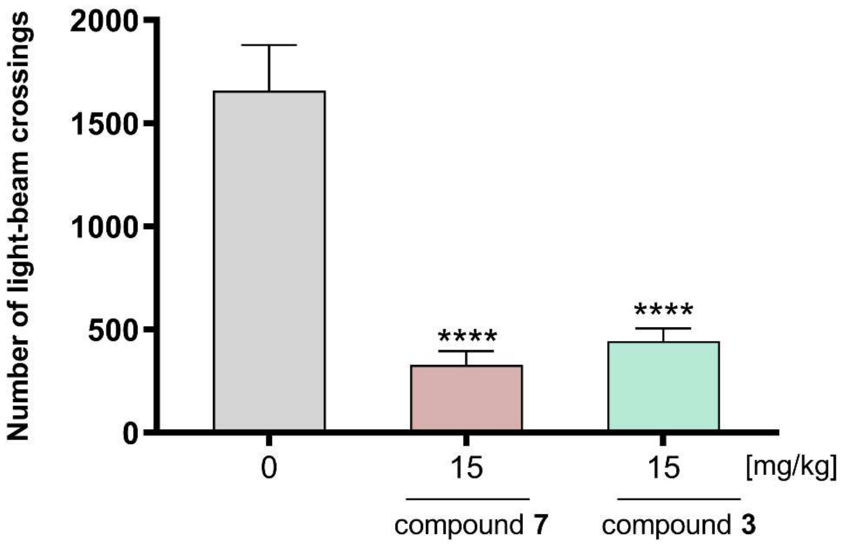

Figure S30. The influence of **3** and **7** on the spontaneous locomotor activity. Results are shown as number of light beam crossings during 30 min of observation beginning at 30 min after compound administration (i.p.). Each value represents the mean ± S.E.M. for 8-10 animals. 0 – vehicle (1% Tween 80). Statistical analysis: one-way ANOVA followed by post hoc Dunnett's test. Statistical significance compared to vehicle-treated animals: **** $p < 0.0001$.

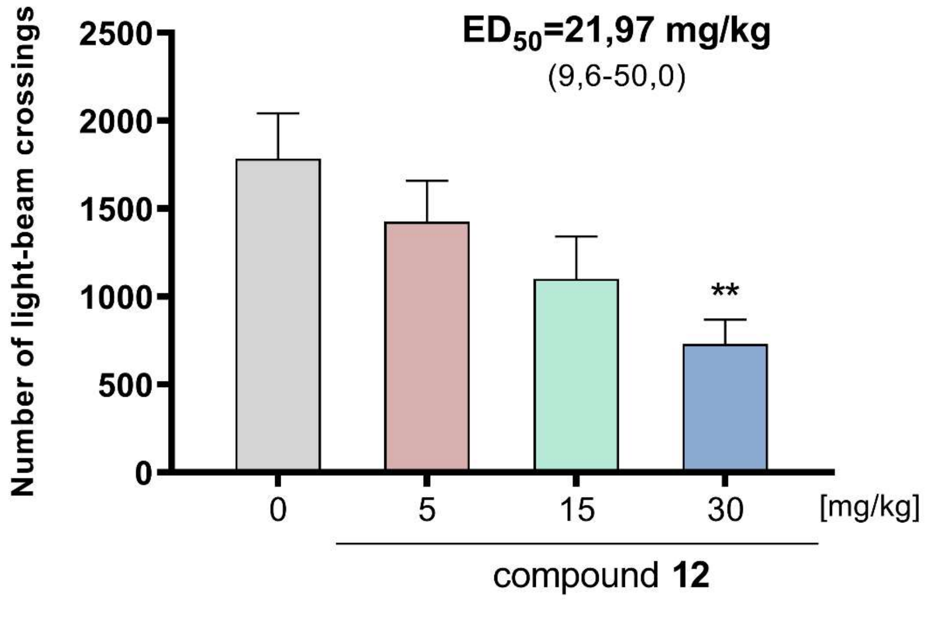

Figure S31. The influence of **12** on the spontaneous locomotor activity. Results are shown as number of light beam crossings during 30 min of observation beginning at 30 min after compound administration (i.p.). Each value represents the mean ± S.E.M. for 8-10 animals. 0 – vehicle (1% Tween 80). Statistical analysis: one-way ANOVA followed by post hoc Dunnett's test. Statistical significance compared to vehicle-treated animals: ** $p < 0.01$.

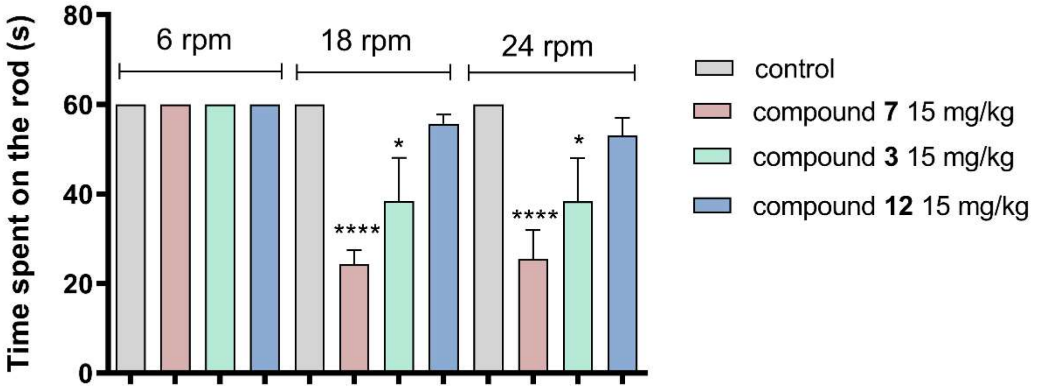

Figure S32. The influence of the test compounds **3**, **7** and **12** on rotarod test performance (measure of motor coordination). Bar graph plots the time (sec.) spent on the rotating rod when tested at different speed (6 rpm, 18 rpm and 24 rpm). Each value represents the mean ± S.E.M. for 6-8 animals. Control – mice treated with vehicle (1% Tween 80). Statistical analysis: one-way ANOVA followed by post hoc Dunnett's test. Statistical significance compared to vehicle-treated animals: * $p < 0.05$; **** $p < 0.0001$.

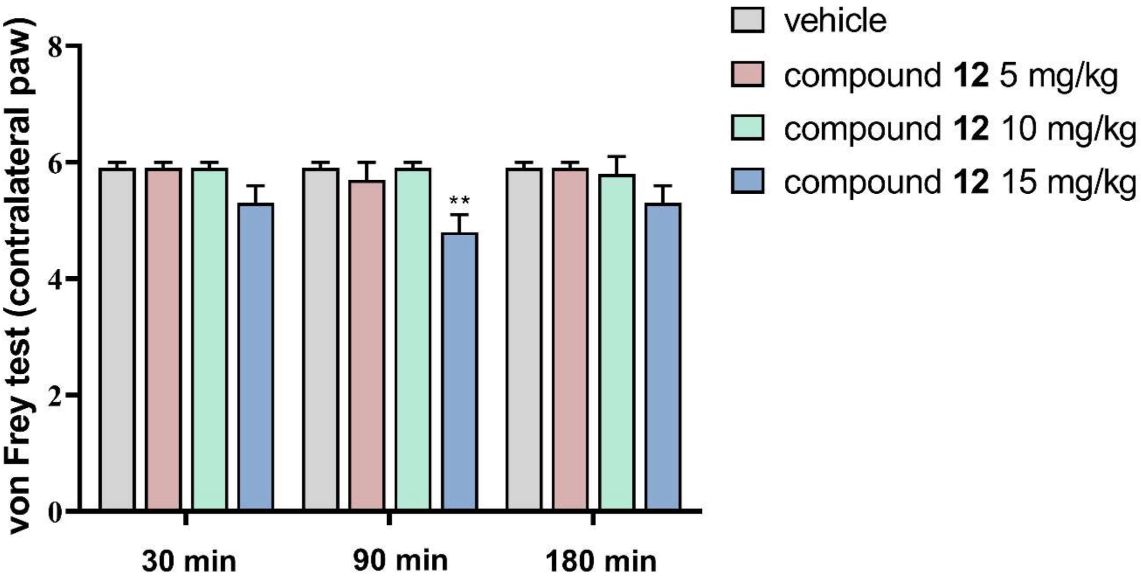

Figure S33. The effects of single i.p. administration of **12** (5, 10 and 15 mg/kg) on mechanical (von Frey test) stimulus on day 14 following CCI to the sciatic nerve, were evaluated (n = 7-8 animals per group). The results are presented as means ± SEM. Intergroup differences were analyzed by one-way ANOVA with Bonferroni's multiple comparison post-hoc test. **$p < 0.01$ vs vehicle-treated group.

6. ^{1}H-, ^{13}C-NMR spectra, HRMS analysis and HPLC traces

Compound 1

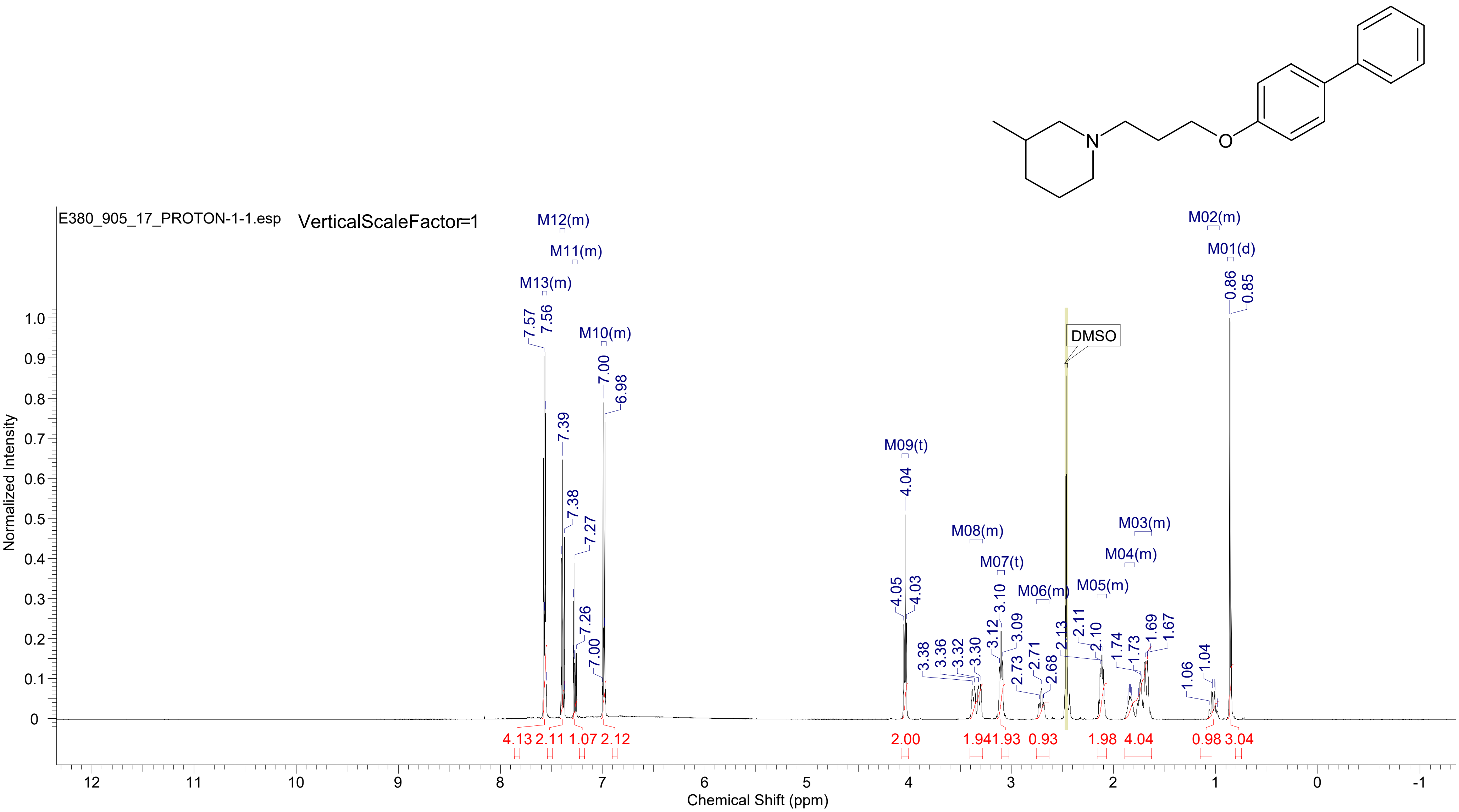

E380_905_17_PROTON-1-1.esp
VerticalScaleFactor=1
M13(m)
M12(m)
M11(m)
M10(m)
M09(t)
M08(m)
M07(t)
M06(m)
M05(m)
M04(m)
M03(m)
M02(m)
M01(d)
DMSO
4.13
2.11
1.07
2.12
2.00
1.94
1.93
0.93
1.98
4.04
0.98
3.04
Normalized Intensity
Chemical Shift (ppm)
N
O

Compound 2

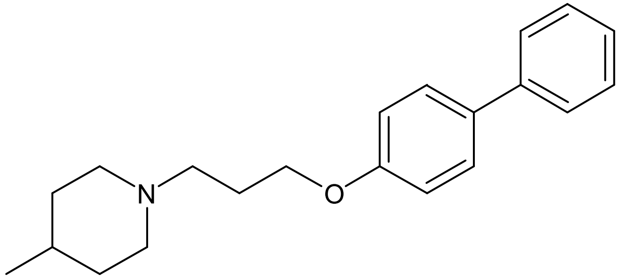

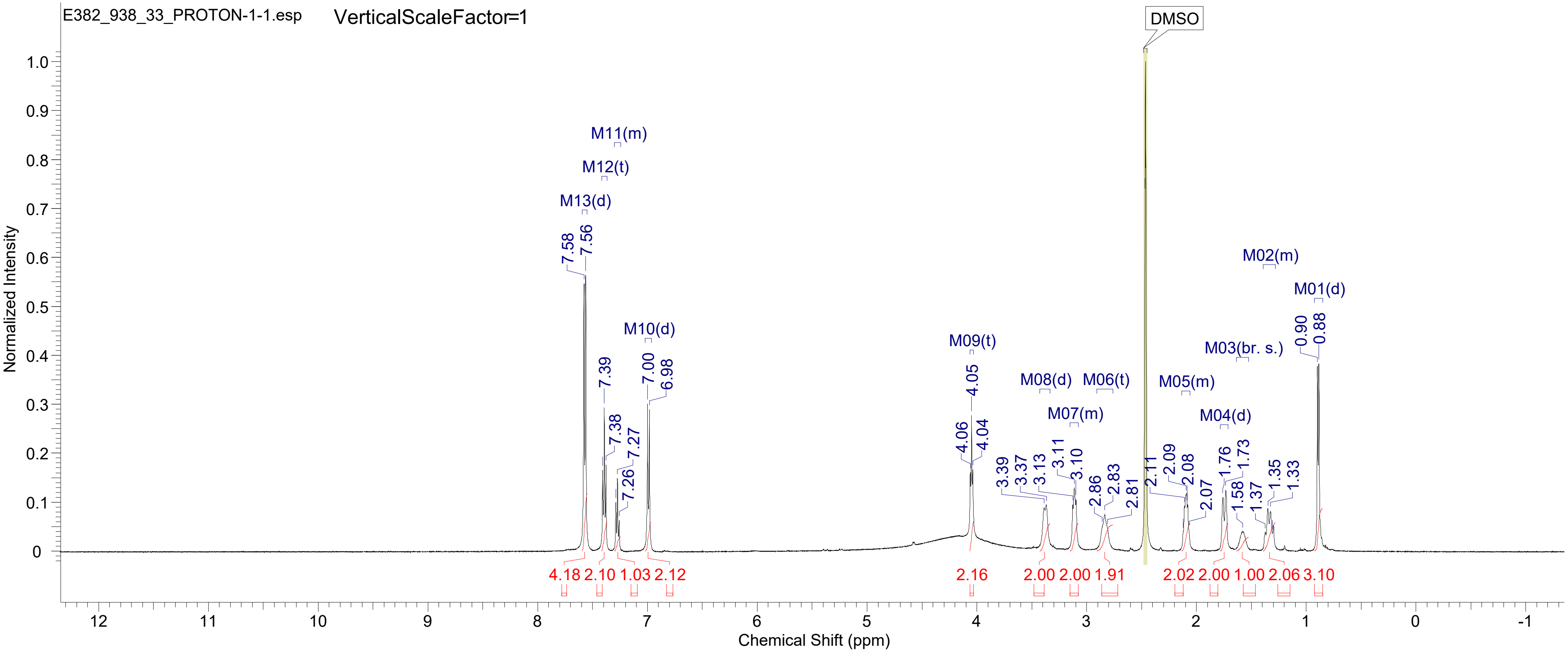
E382_938_33_PROTON-1-1.esp
VerticalScaleFactor=1
DMSO
M11(m)
M12(t)
M13(d)
M10(d)
M09(t)
M08(d)
M06(t)
M07(m)
M05(m)
M04(d)
M03(br. s.)
M02(m)
M01(d)
7.58
7.56
7.39
7.38
7.27
7.26
7.00
6.98
4.06
4.05
4.04
3.39
3.37
3.13
3.11
3.10
2.86
2.83
2.81
2.11
2.09
2.08
2.07
1.76
1.73
1.58
1.37
1.35
1.33
0.90
0.88
4.18
2.10
1.03
2.12
2.16
2.00
2.00
1.91
2.02
2.00
1.00
2.06
3.10
Normalized Intensity
Chemical Shift (ppm)

Compound 3

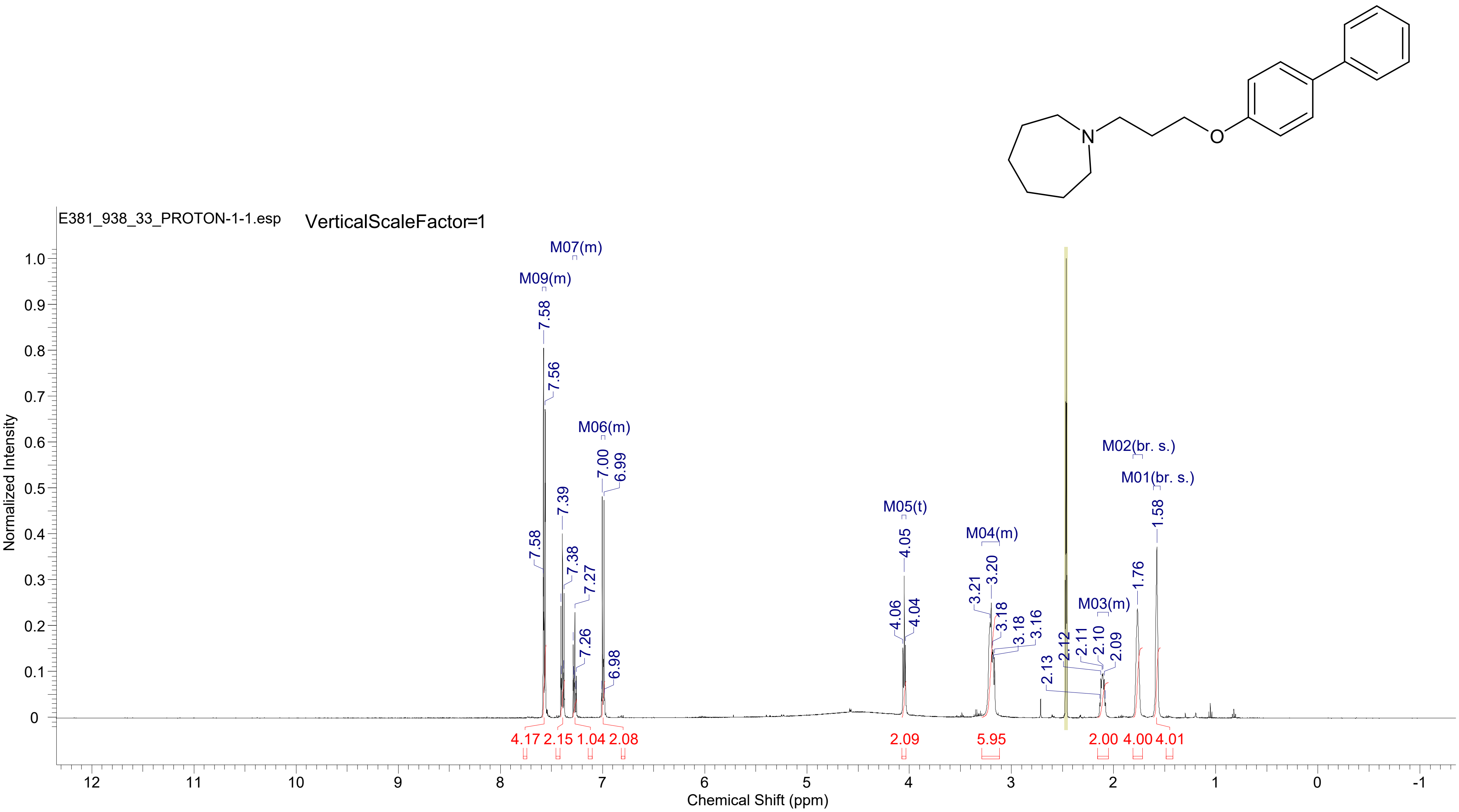

N
O
E381_938_33_PROTON-1-1.esp
VerticalScaleFactor=1
M07(m)
M09(m)
M06(m)
M05(t)
M04(m)
M03(m)
M02(br. s.)
M01(br. s.)
7.58
7.56
7.39
7.38
7.27
7.26
7.00
6.99
6.98
4.06
4.05
4.04
3.21
3.20
3.18
3.18
3.16
2.13
2.12
2.11
2.10
2.09
1.76
1.58
4.17
2.15
1.04
2.08
2.09
5.95
2.00
4.00
4.01
Normalized Intensity
Chemical Shift (ppm)

Compound 4

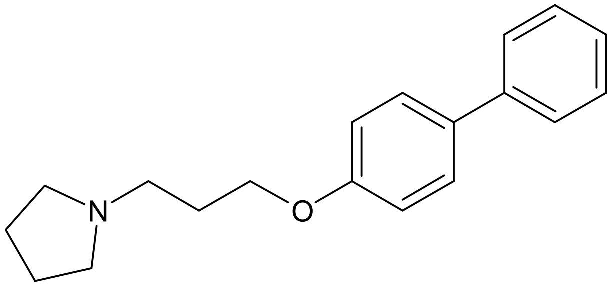

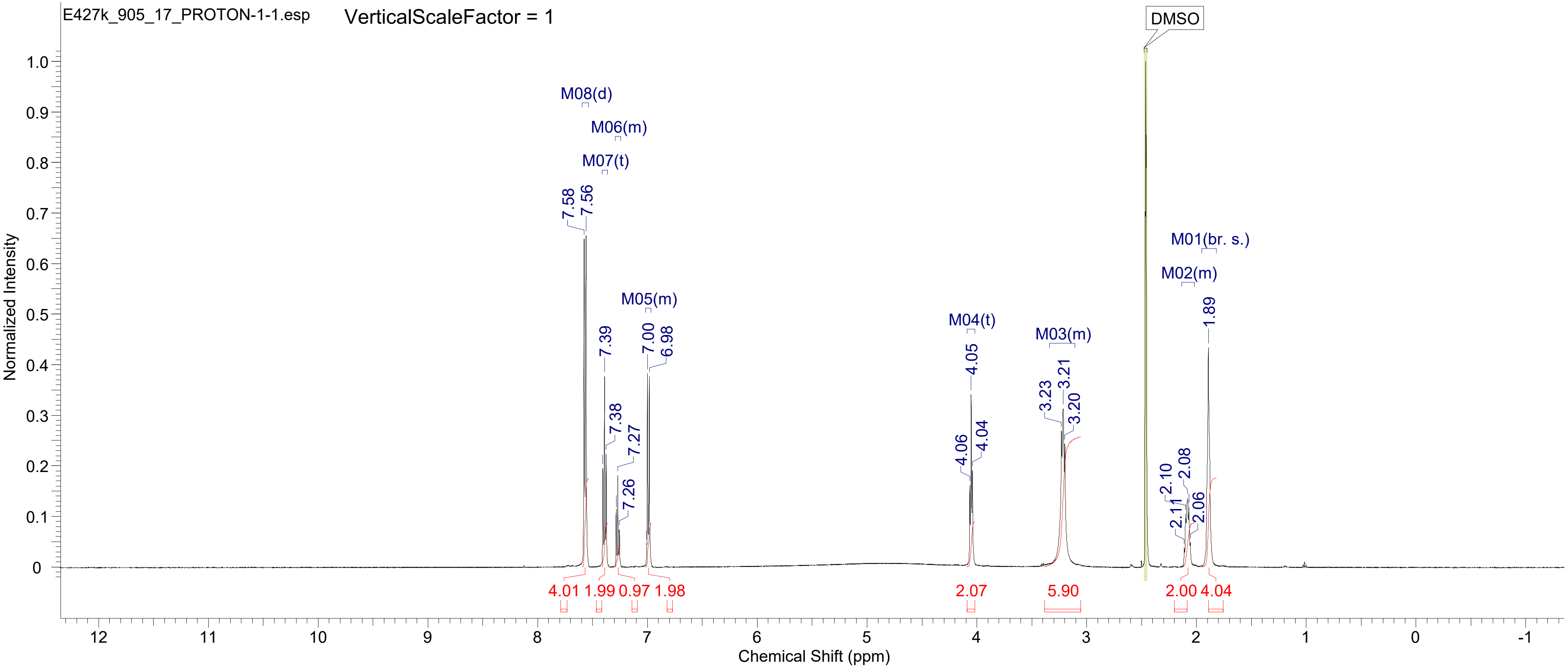

E427k_905_17_PROTON-1-1.esp
VerticalScaleFactor = 1
DMSO
M08(d)
M06(m)
M07(t)
7.58
7.56
7.39
7.38
7.27
7.26
M05(m)
7.00
6.98
M04(t)
4.05
4.06
4.04
M03(m)
3.23
3.21
3.20
M01(br. s.)
M02(m)
1.89
2.10
2.08
2.11
2.06
4.01
1.99
0.97
1.98
2.07
5.90
2.00
4.04
Normalized Intensity
Chemical Shift (ppm)

Compound 6

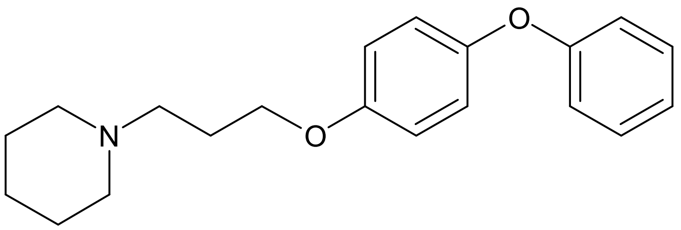

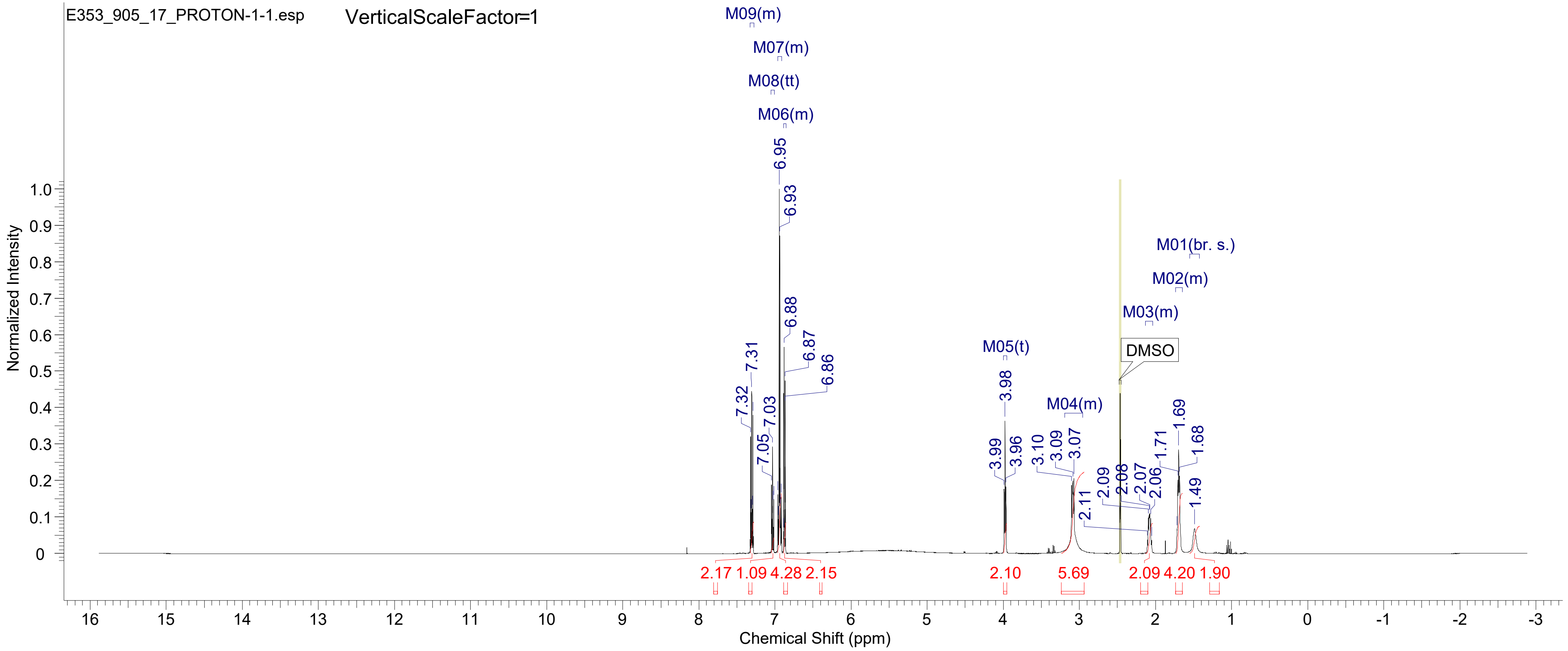

E353_905_17_PROTON-1-1.esp
VerticalScaleFactor=1
M09(m)
M07(m)
M08(tt)
M06(m)
M05(t)
M04(m)
M03(m)
M02(m)
M01(br. s.)
DMSO
6.95
6.93
6.88
6.87
6.86
7.32
7.31
7.05
7.03
3.99
3.98
3.96
3.10
3.09
3.07
2.11
2.09
2.08
2.07
2.06
1.71
1.69
1.68
1.49
2.17
1.09
4.28
2.15
2.10
5.69
2.09
4.20
1.90
Normalized Intensity
Chemical Shift (ppm)

Compound 7

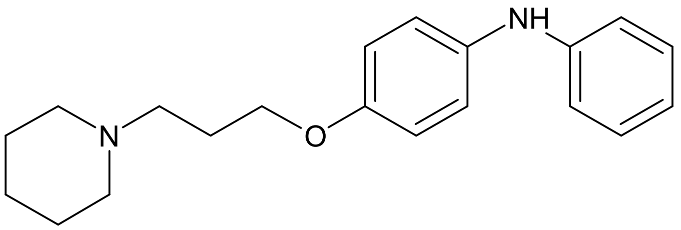

NH
N
O

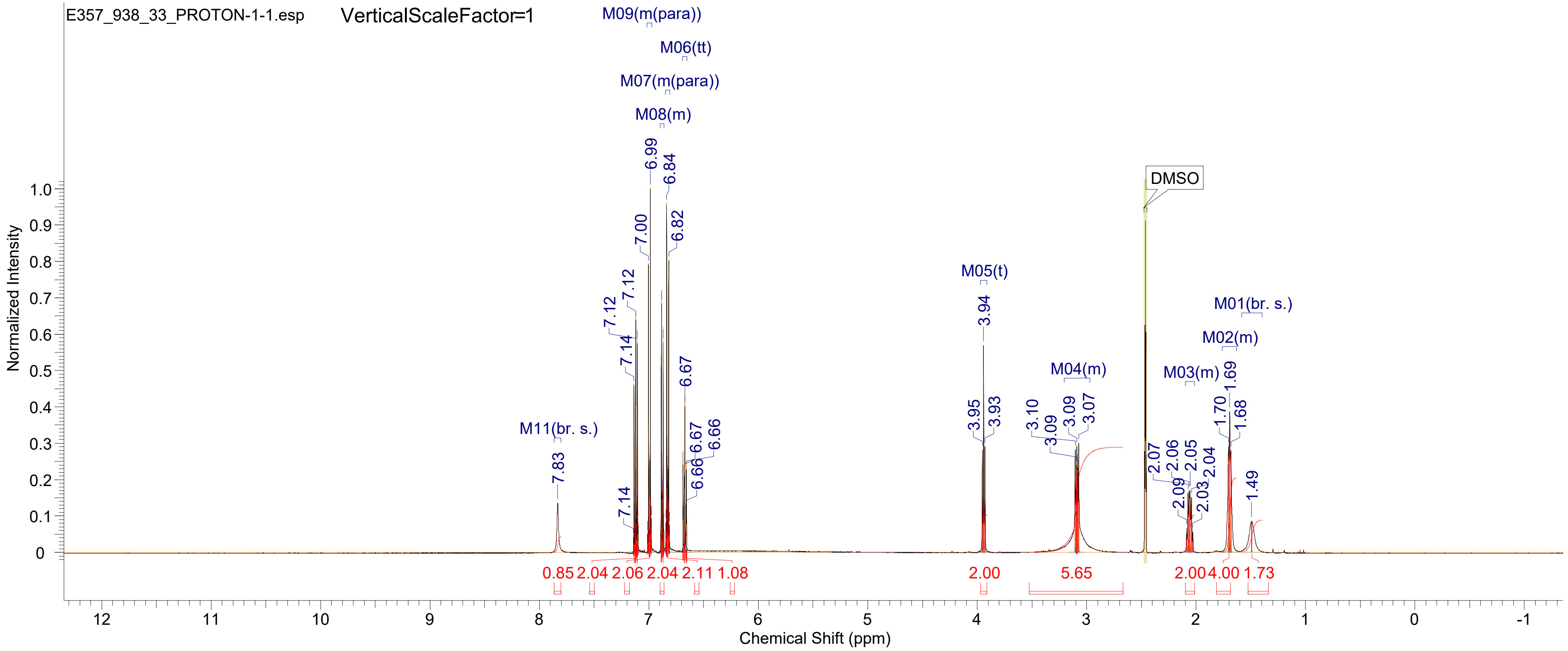

E357_938_33_PROTON-1-1.esp
VerticalScaleFactor=1
M09(m(para))
M06(tt)
M07(m(para))
M08(m)
M05(t)
M04(m)
M03(m)
M02(m)
M01(br. s.)
M11(br. s.)
DMSO
7.83
7.14
7.14
7.12
7.12
7.00
6.99
6.84
6.82
6.67
6.67
6.66
6.66
3.95
3.94
3.93
3.10
3.09
3.09
3.07
2.09
2.07
2.06
2.05
2.04
2.03
1.70
1.69
1.68
1.49
0.85
2.04
2.06
2.04
2.11
1.08
2.00
5.65
2.00
4.00
1.73
Normalized Intensity
Chemical Shift (ppm)

Compound 8

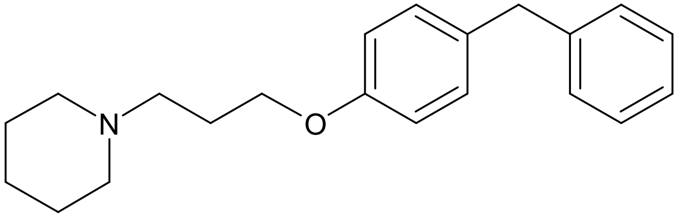

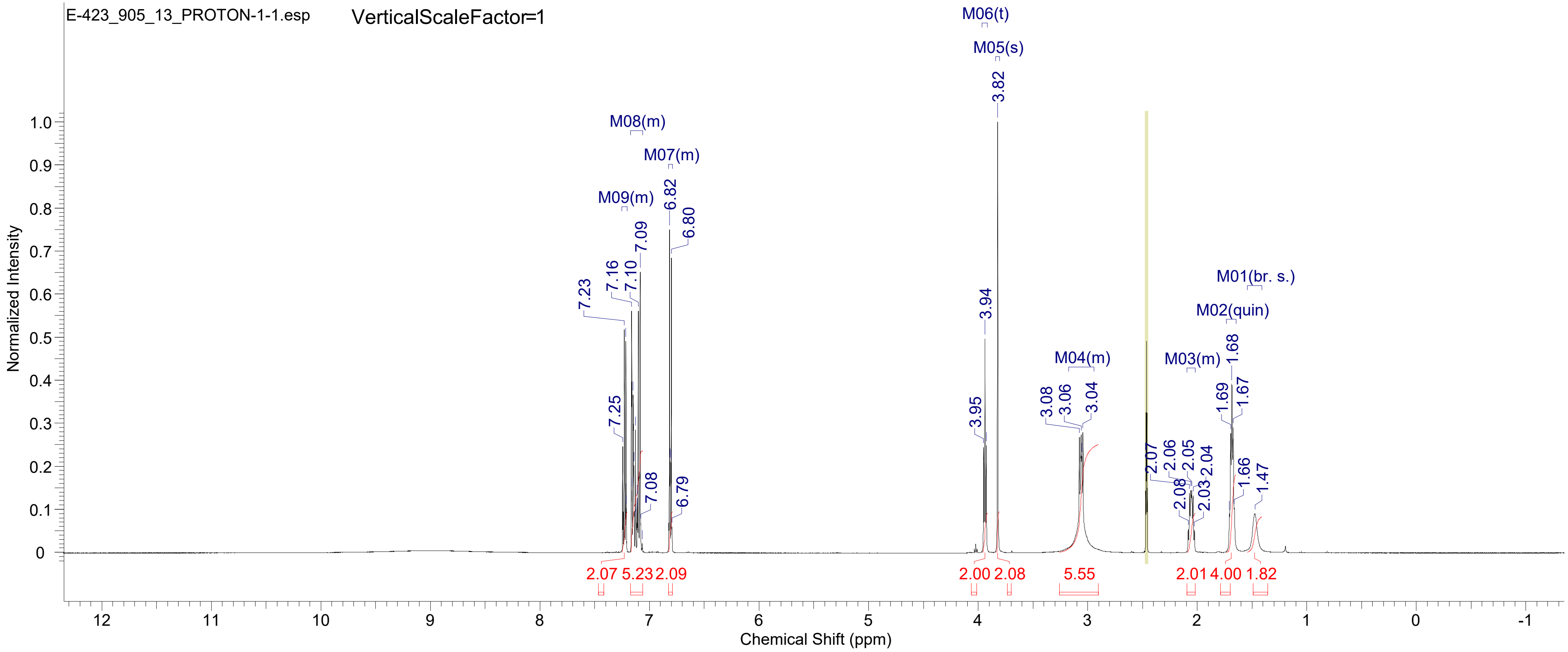
E-423_905_13_PROTON-1-1.esp
VerticalScaleFactor=1
M06(t)
M05(s)
3.82
M08(m)
M07(m)
M09(m)
6.82
6.80
7.09
7.16
7.10
7.23
3.94
M01(br. s.)
M02(quin)
M04(m)
M03(m)
1.68
7.25
3.08
3.06
3.04
3.95
1.69
1.67
2.07
2.06
2.05
2.04
2.08
2.03
1.66
1.47
7.08
6.79
2.07
5.23
2.09
2.00
2.08
5.55
2.01
4.00
1.82
Normalized Intensity
Chemical Shift (ppm)

Compound 9

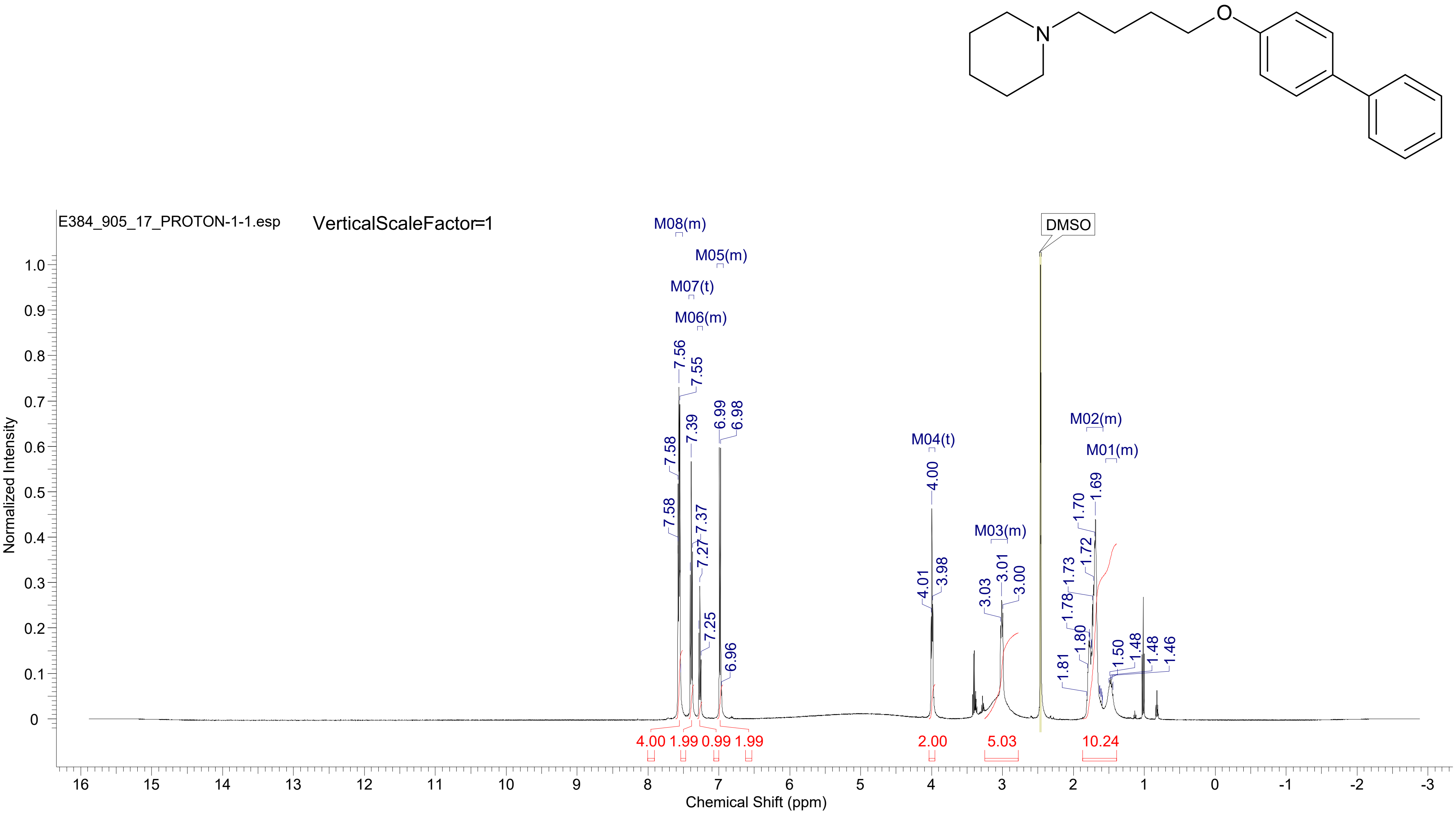

O
N
E384_905_17_PROTON-1-1.esp
VerticalScaleFactor=1
M08(m)
M05(m)
M07(t)
M06(m)
DMSO
M04(t)
M03(m)
M02(m)
M01(m)
4.00 1.99 0.99 1.99
2.00
5.03
10.24
Normalized Intensity
Chemical Shift (ppm)

Compound 12

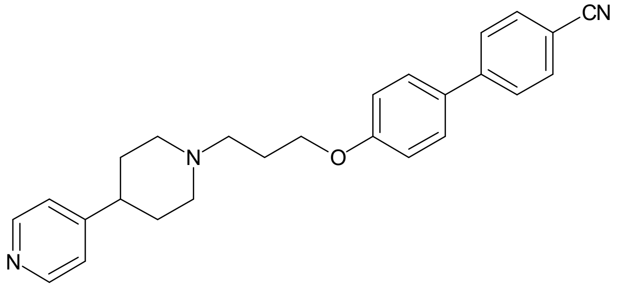

CN
N
O
N

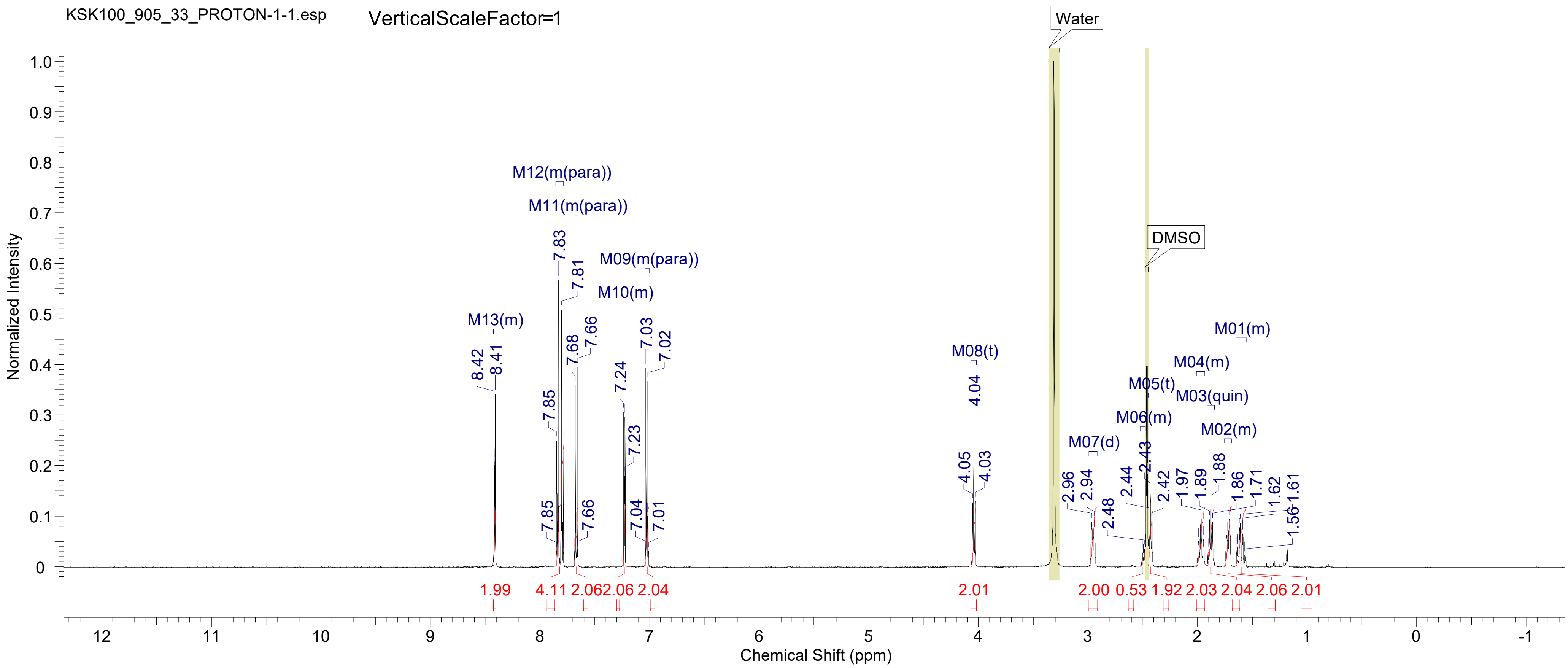

KSK100_905_33_PROTON-1-1.esp
VerticalScaleFactor=1
Water
DMSO
M12(m(para))
M11(m(para))
M09(m(para))
M10(m)
M13(m)
M08(t)
M07(d)
M06(m)
M05(t)
M04(m)
M03(quin)
M02(m)
M01(m)
8.42
8.41
7.85
7.83
7.81
7.85
7.68
7.66
7.66
7.24
7.23
7.04
7.03
7.02
7.01
4.05
4.04
4.03
2.96
2.94
2.48
2.44
2.43
2.42
1.97
1.89
1.88
1.86
1.71
1.62
1.61
1.56
1.99
4.11
2.06
2.06
2.04
2.01
2.00
0.53
1.92
2.03
2.04
2.06
2.01
Normalized Intensity
Chemical Shift (ppm)

Compound 13

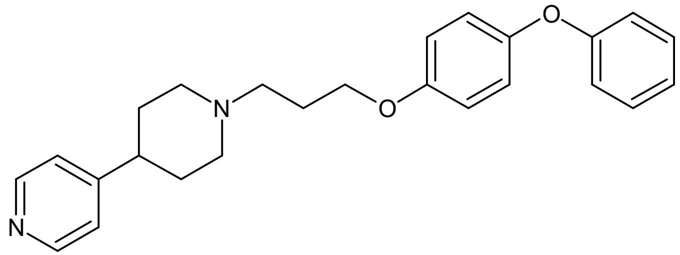

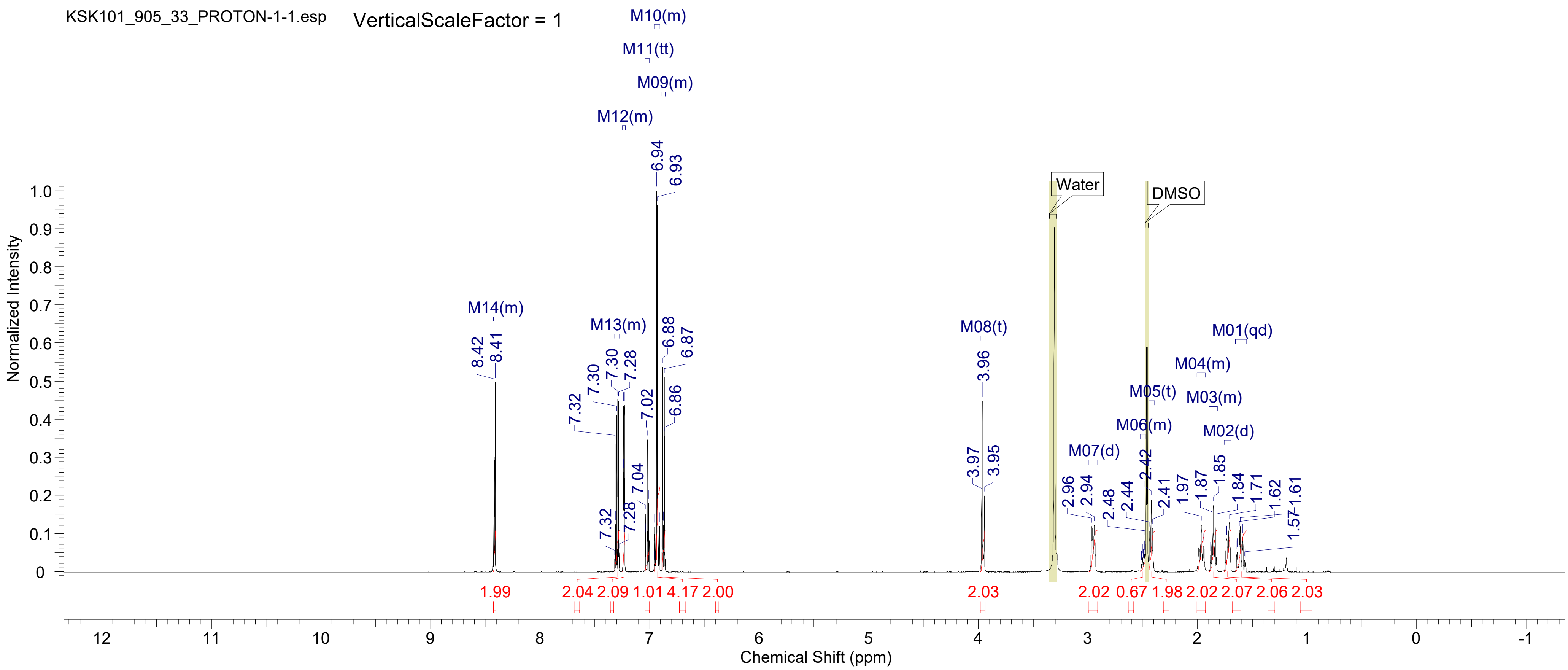
KSK101_905_33_PROTON-1-1.esp
VerticalScaleFactor = 1
Water
DMSO
M14(m)
M13(m)
M12(m)
M11(tt)
M10(m)
M09(m)
M08(t)
M07(d)
M06(m)
M05(t)
M04(m)
M03(m)
M02(d)
M01(qd)
Normalized Intensity
Chemical Shift (ppm)
1.99
2.04
2.09
1.01
4.17
2.00
2.03
2.02
0.67
1.98
2.02
2.07
2.06
2.03

Compound 14

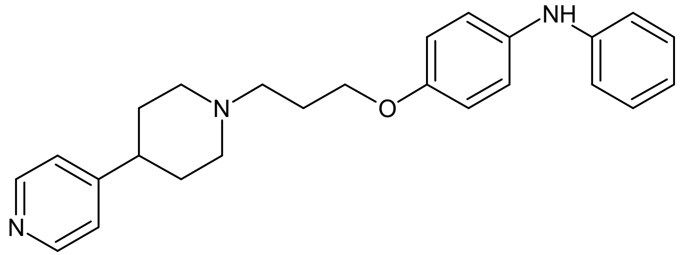

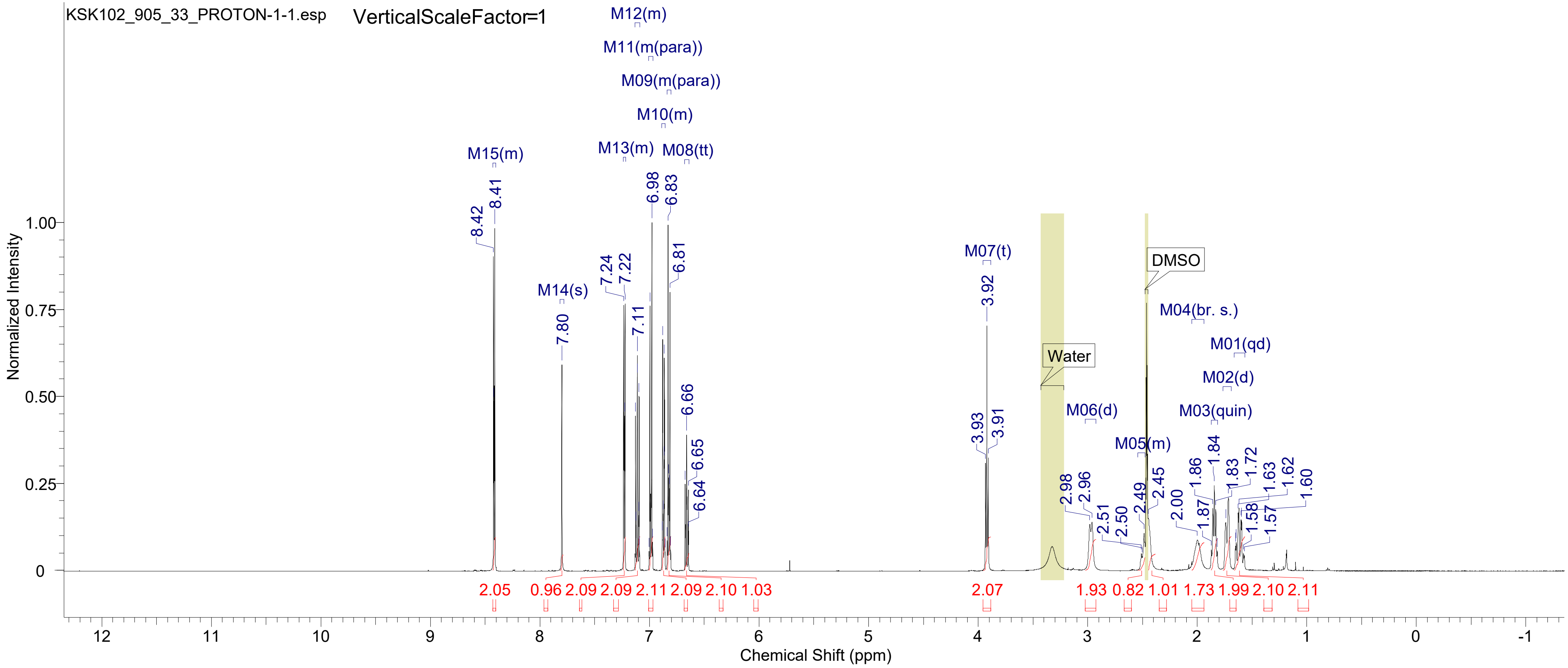

KSK102_905_33_PROTON-1-1.esp
VerticalScaleFactor=1
Normalized Intensity
Chemical Shift (ppm)
Water
DMSO

Compound 15

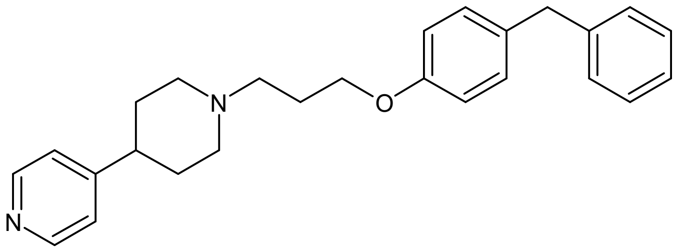

N
N
O

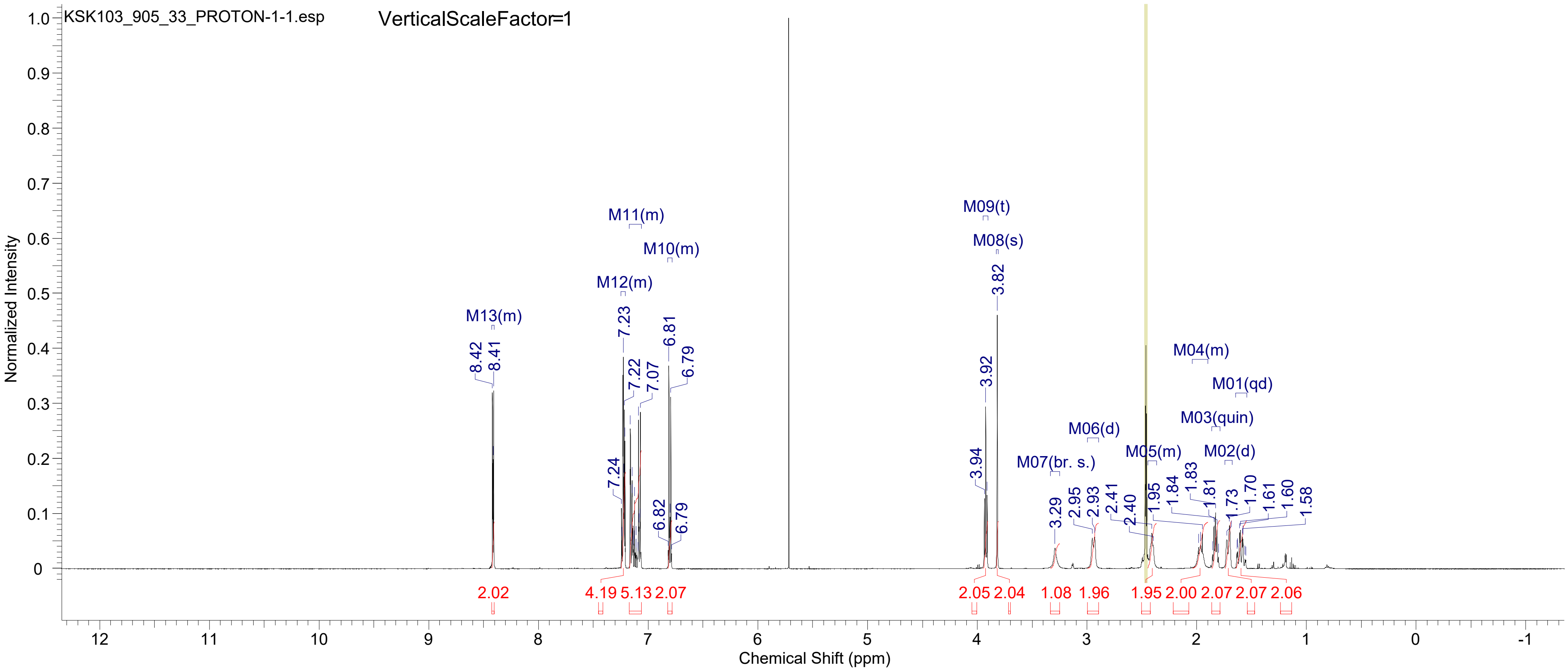

KSK103_905_33_PROTON-1-1.esp
VerticalScaleFactor=1
Normalized Intensity
Chemical Shift (ppm)
M13(m)
M12(m)
M11(m)
M10(m)
M09(t)
M08(s)
M07(br. s.)
M06(d)
M05(m)
M04(m)
M03(quin)
M02(d)
M01(qd)
8.42
8.41
7.24
7.23
7.22
7.07
6.82
6.81
6.79
6.79
3.94
3.92
3.82
3.29
2.95
2.93
2.41
2.40
1.95
1.84
1.83
1.81
1.73
1.70
1.61
1.60
1.58
2.02
4.19
5.13
2.07
2.05
2.04
1.08
1.96
1.95
2.00
2.07
2.07
2.06
1.0
0.9
0.8
0.7
0.6
0.5
0.4
0.3
0.2
0.1
0
12
11
10
9
8
7
6
5
4
3
2
1
0
-1

Compound 1

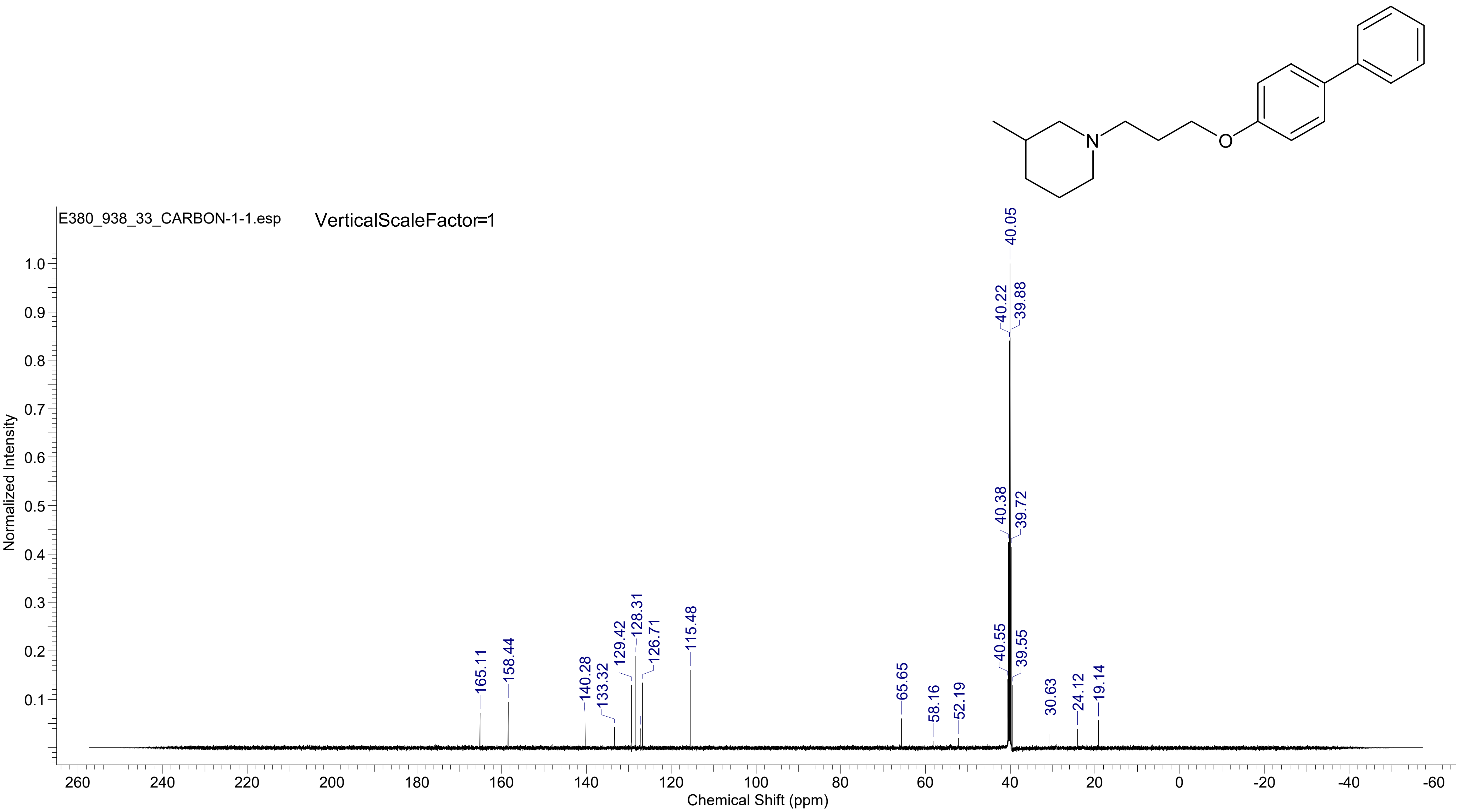

N
O
E380_938_33_CARBON-1-1.esp
VerticalScaleFactor=1
40.05
40.22
39.88
40.38
39.72
40.55
39.55
165.11
158.44
140.28
133.32
129.42
128.31
126.71
115.48
65.65
58.16
52.19
30.63
24.12
19.14
Normalized Intensity
Chemical Shift (ppm)

Compound 2

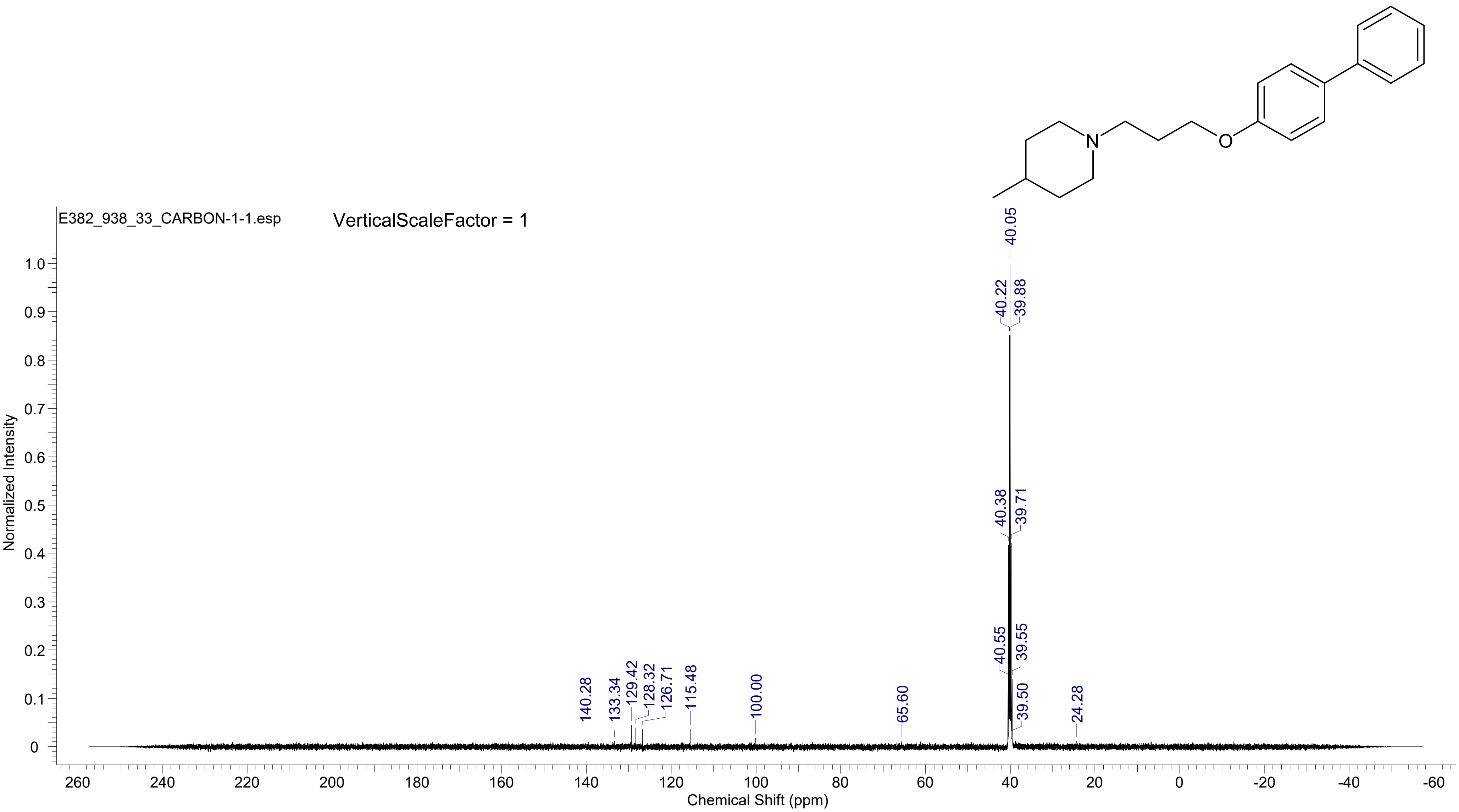

N
O
E382_938_33_CARBON-1-1.esp
VerticalScaleFactor = 1
40.05
40.22
39.88
40.38
39.71
40.55
39.55
39.50
140.28
133.34
129.42
128.32
126.71
115.48
100.00
65.60
24.28
Normalized Intensity
Chemical Shift (ppm)

Compound 3

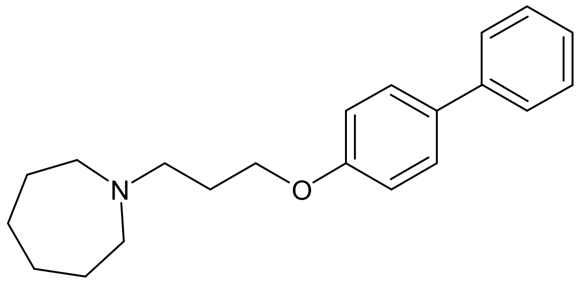

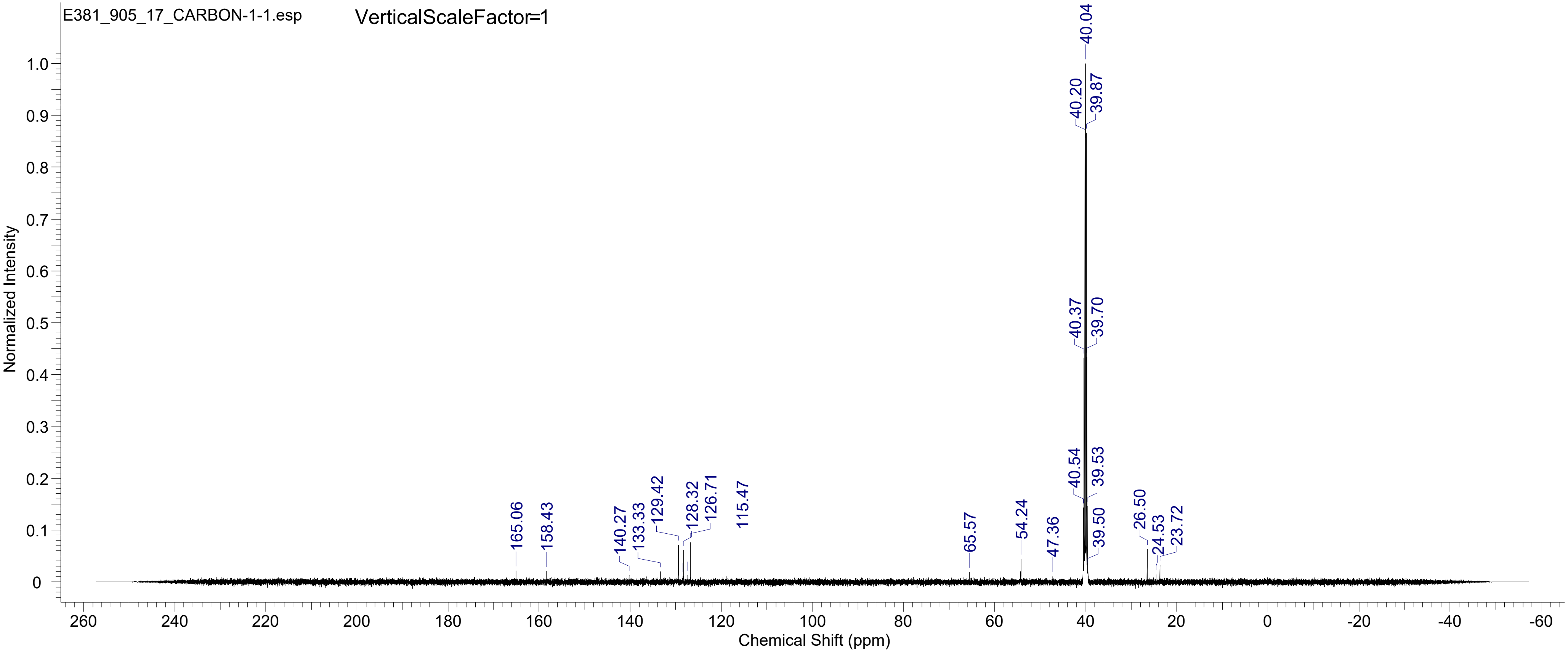
E381_905_17_CARBON-1-1.esp
VerticalScaleFactor=1
165.06
158.43
140.27
133.33
129.42
128.32
126.71
115.47
65.57
54.24
47.36
40.54
40.37
40.20
40.04
39.87
39.70
39.53
39.50
26.50
24.53
23.72
Normalized Intensity
Chemical Shift (ppm)

Compound 4

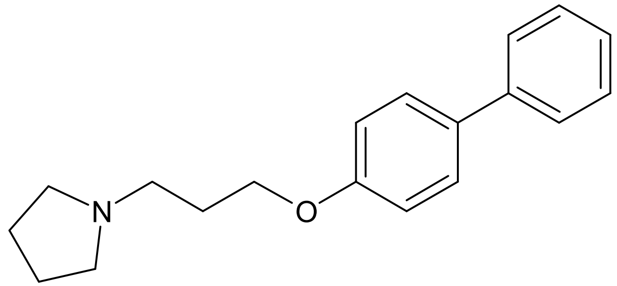

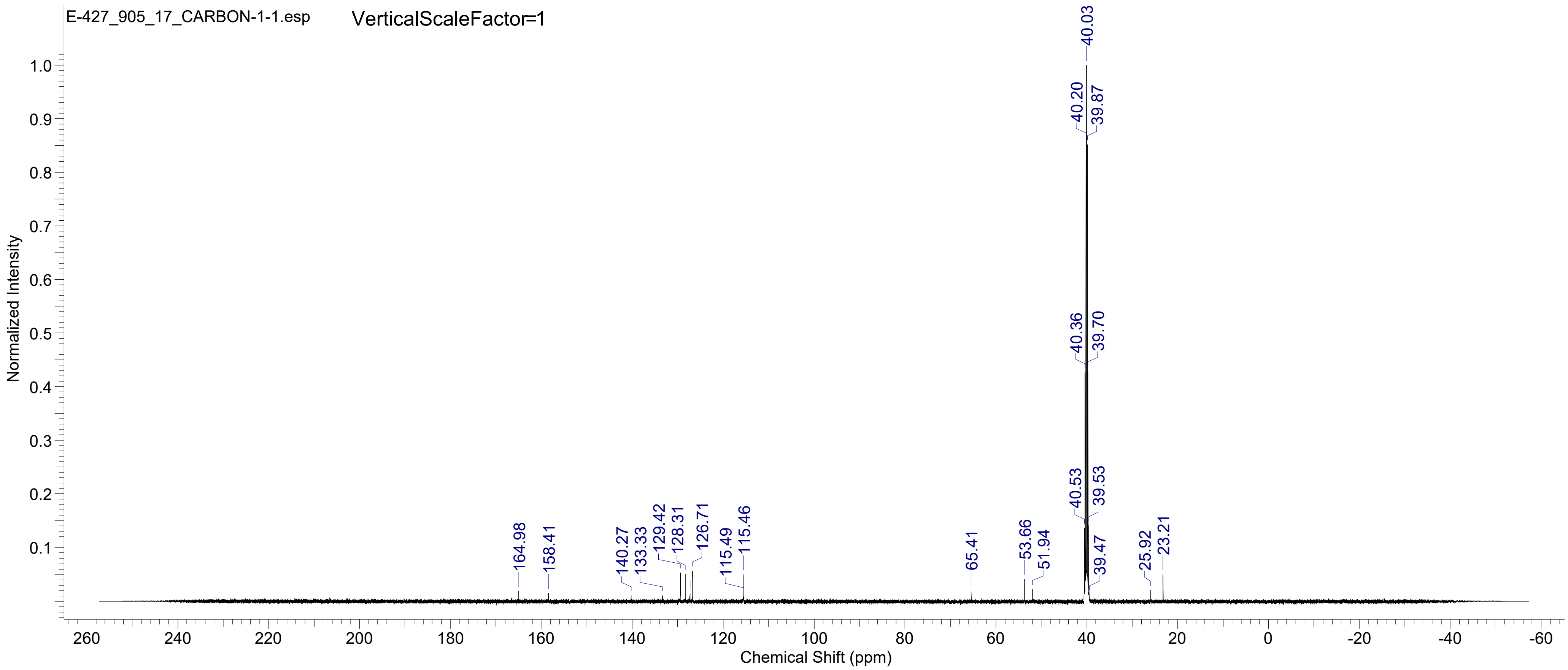

E-427_905_17_CARBON-1-1.esp
VerticalScaleFactor=1
164.98
158.41
140.27
133.33
129.42
128.31
126.71
115.49
115.46
65.41
53.66
51.94
40.53
40.36
40.20
40.03
39.87
39.70
39.53
39.47
25.92
23.21
Normalized Intensity
Chemical Shift (ppm)

Compound 6

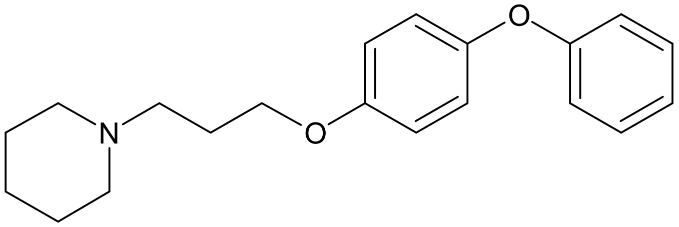

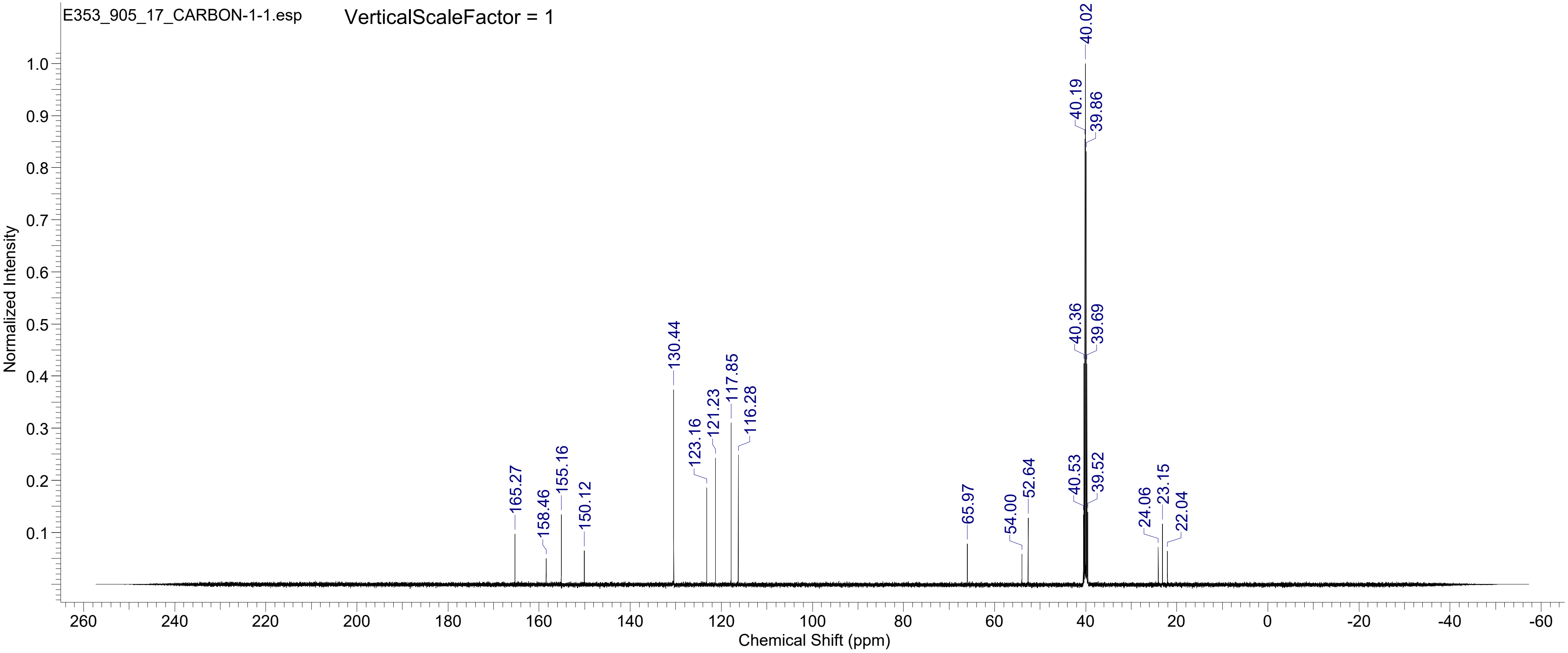

E353_905_17_CARBON-1-1.esp
VerticalScaleFactor = 1
Normalized Intensity
Chemical Shift (ppm)
165.27
158.46
155.16
150.12
130.44
123.16
121.23
117.85
116.28
65.97
54.00
52.64
40.53
40.36
40.19
40.02
39.86
39.69
39.52
24.06
23.15
22.04

Compound 7

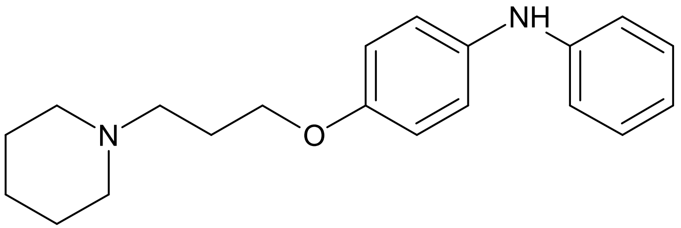
NH
N
O

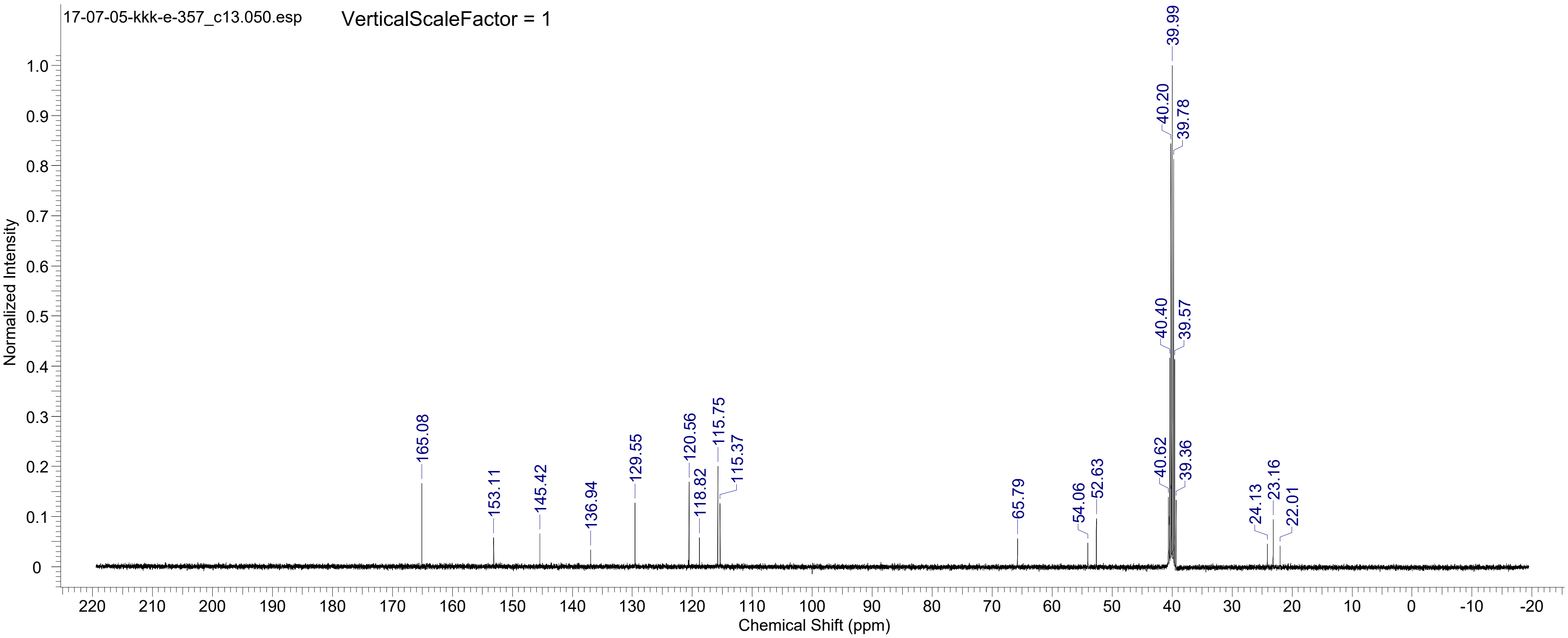
17-07-05-kkk-e-357_c13.050.esp
VerticalScaleFactor = 1
39.99
40.20
39.78
40.40
39.57
165.08
153.11
145.42
136.94
129.55
120.56
118.82
115.75
115.37
65.79
54.06
52.63
40.62
39.36
24.13
23.16
22.01
Normalized Intensity
Chemical Shift (ppm)

Compound 8

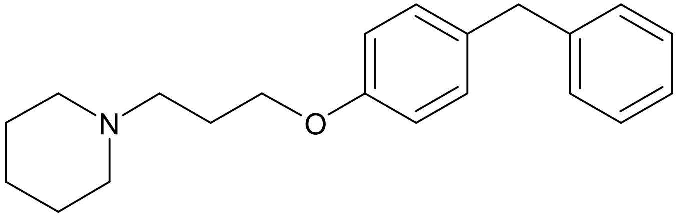

N
O

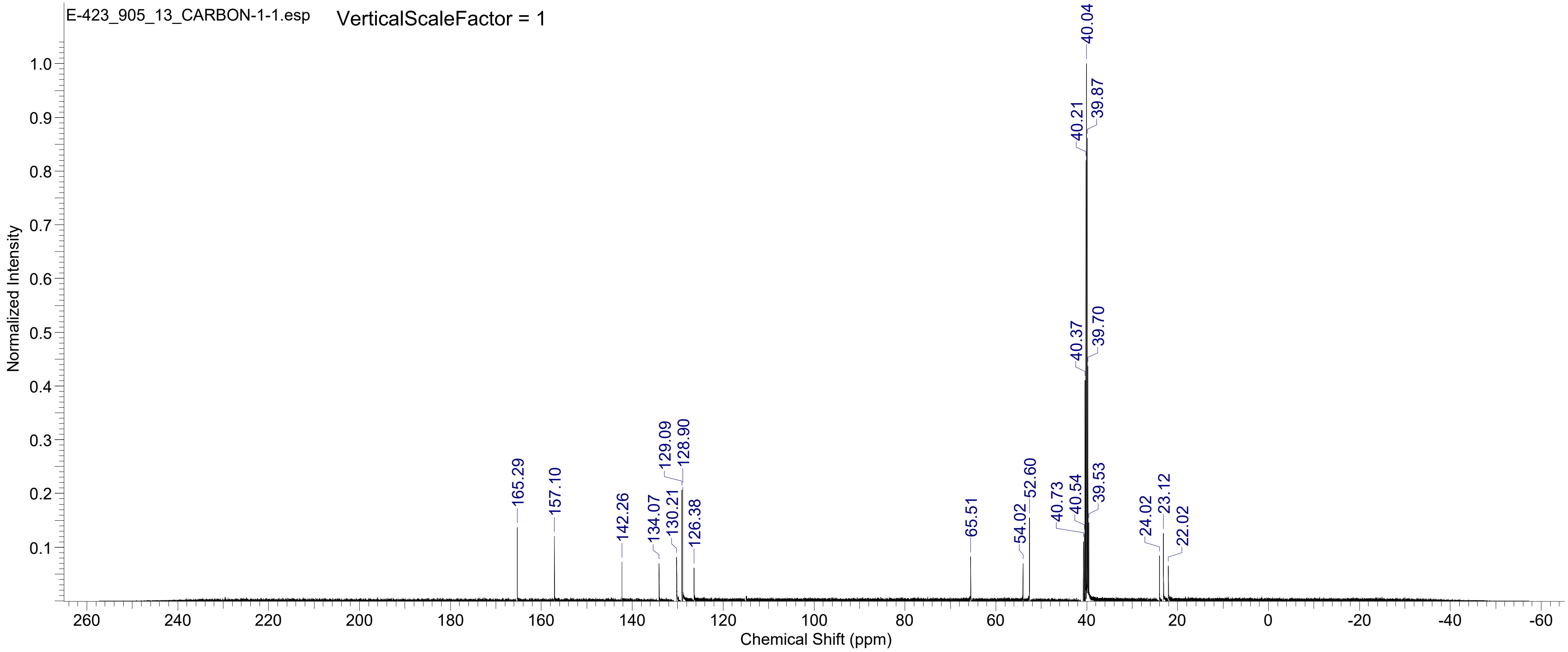

E-423_905_13_CARBON-1-1.esp
VerticalScaleFactor = 1
Normalized Intensity
Chemical Shift (ppm)
165.29
157.10
142.26
134.07
130.21
129.09
128.90
126.38
65.51
54.02
52.60
40.73
40.54
40.37
40.21
40.04
39.87
39.70
39.53
24.02
23.12
22.02

Compound 9

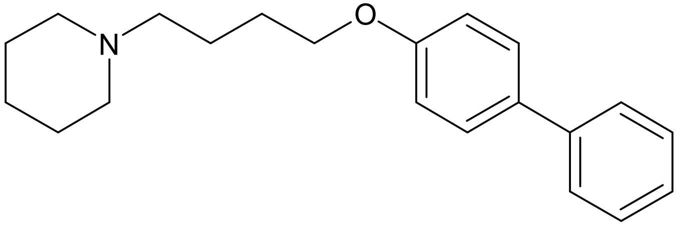
N
O

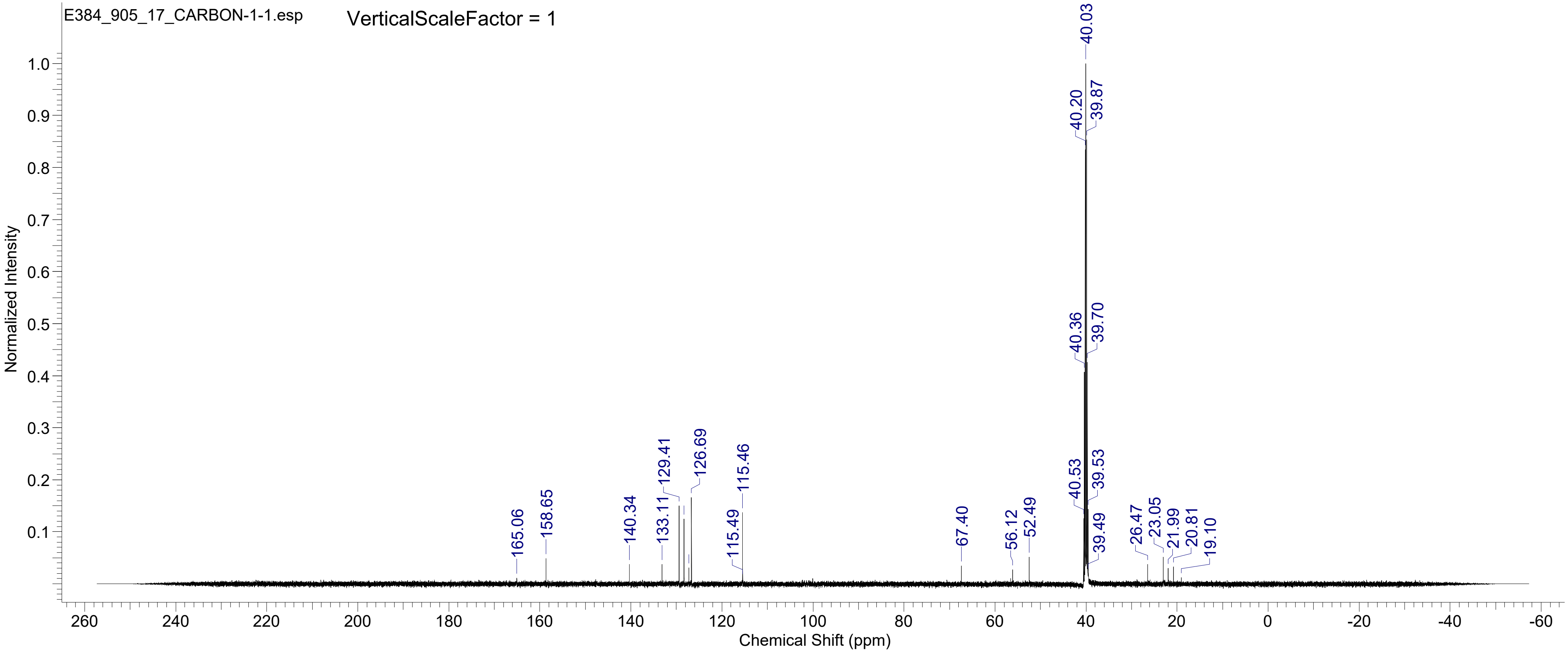
E384_905_17_CARBON-1-1.esp
VerticalScaleFactor = 1
Normalized Intensity
Chemical Shift (ppm)
165.06
158.65
140.34
133.11
129.41
126.69
115.49
115.46
67.40
56.12
52.49
40.53
40.36
40.20
40.03
39.87
39.70
39.53
39.49
26.47
23.05
21.99
20.81
19.10

Compound 12

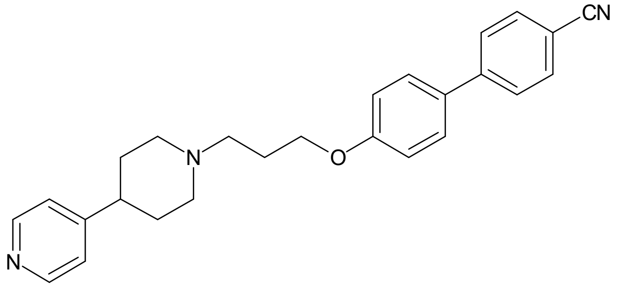

CN
N
O
N

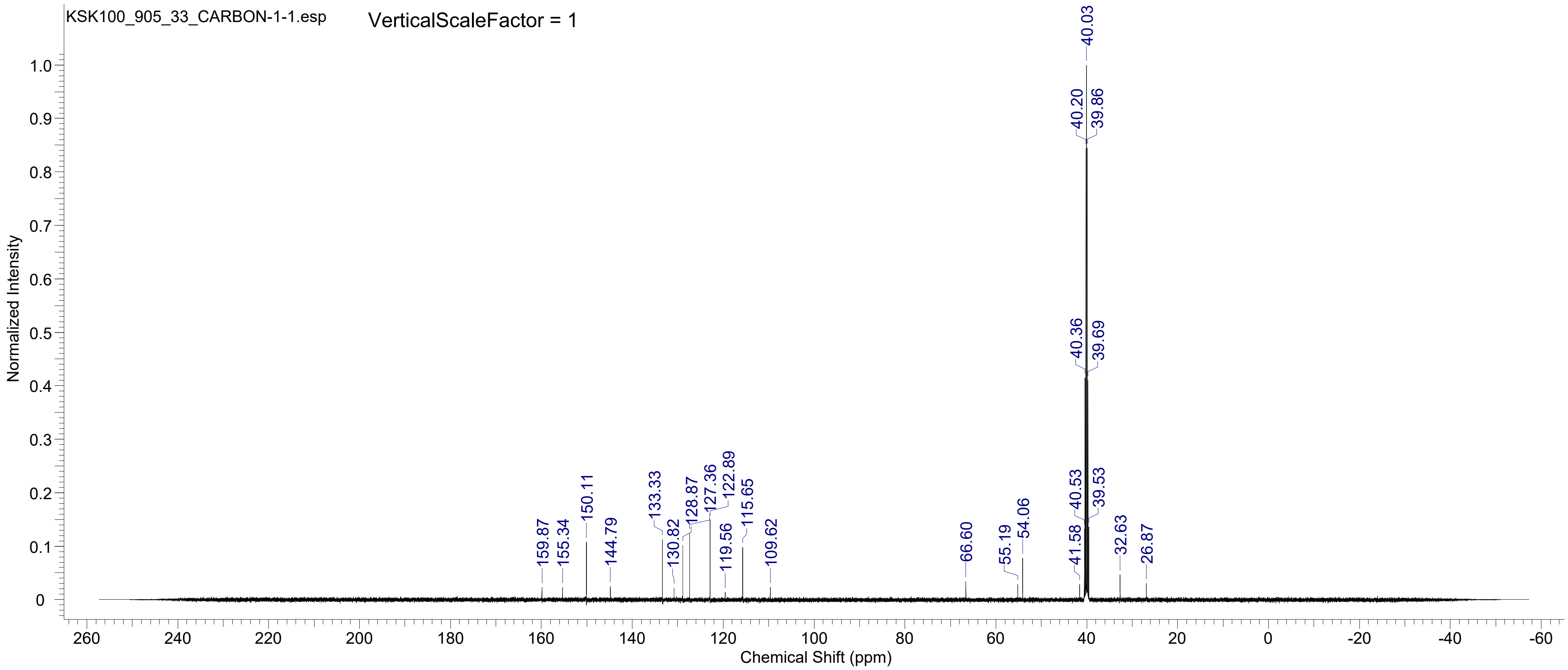

KSK100_905_33_CARBON-1-1.esp
VerticalScaleFactor = 1
40.03
40.20
39.86
40.36
39.69
159.87
155.34
150.11
144.79
133.33
130.82
128.87
127.36
122.89
119.56
115.65
109.62
66.60
55.19
54.06
41.58
40.53
39.53
32.63
26.87
Normalized Intensity
Chemical Shift (ppm)

Compound 13

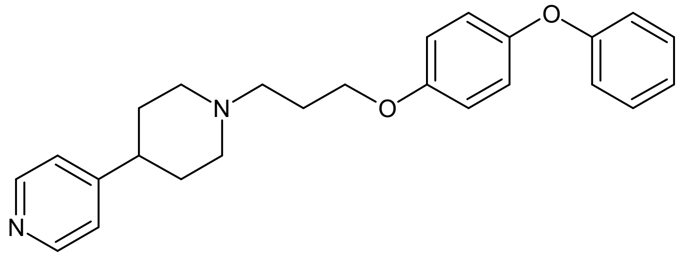

N
N
O
O

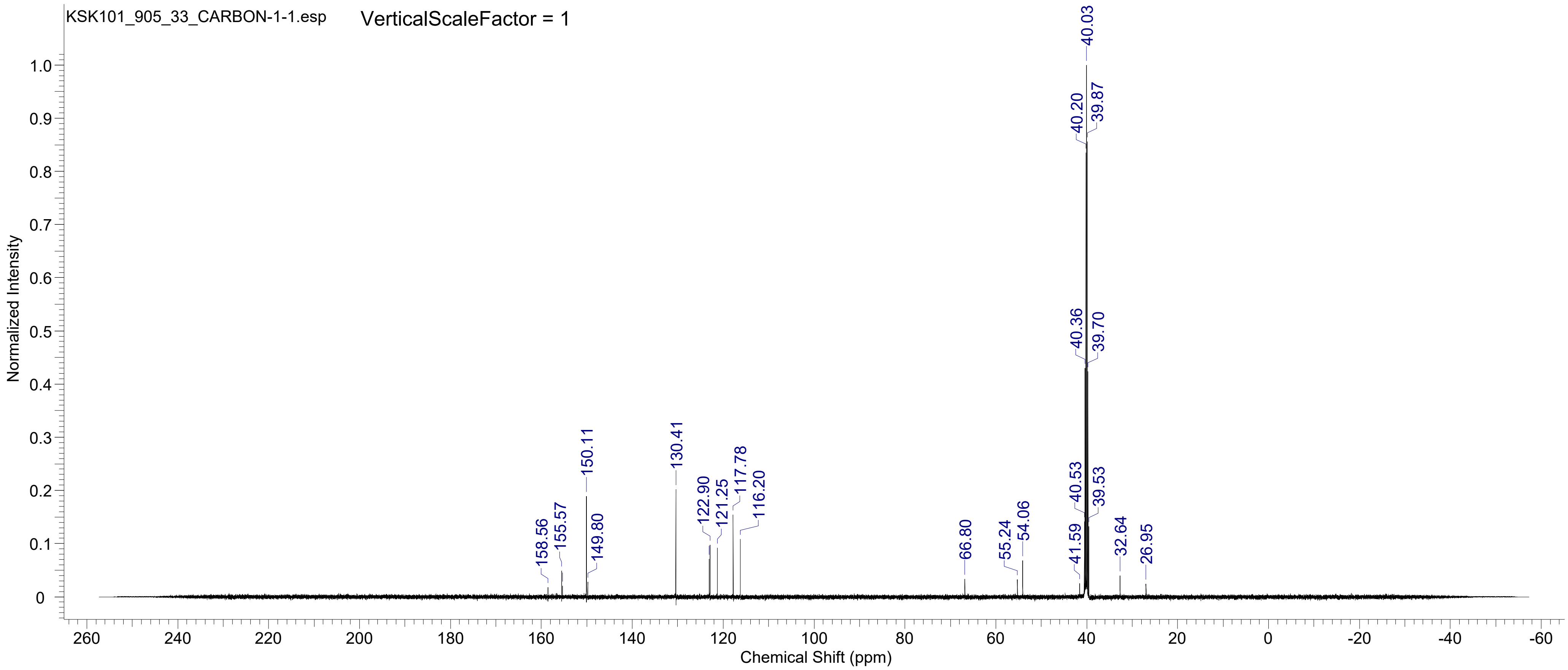

KSK101_905_33_CARBON-1-1.esp
VerticalScaleFactor = 1
Normalized Intensity
Chemical Shift (ppm)
158.56
155.57
150.11
149.80
130.41
122.90
121.25
117.78
116.20
66.80
55.24
54.06
41.59
40.53
40.36
40.20
40.03
39.87
39.70
39.53
32.64
26.95

Compound 14

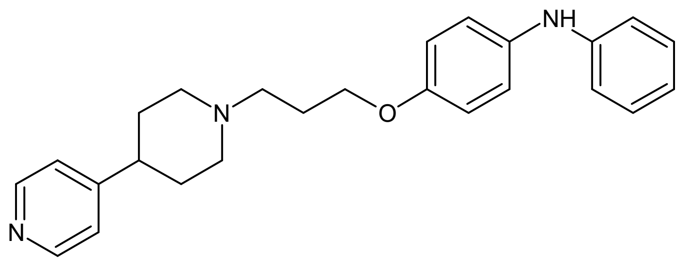

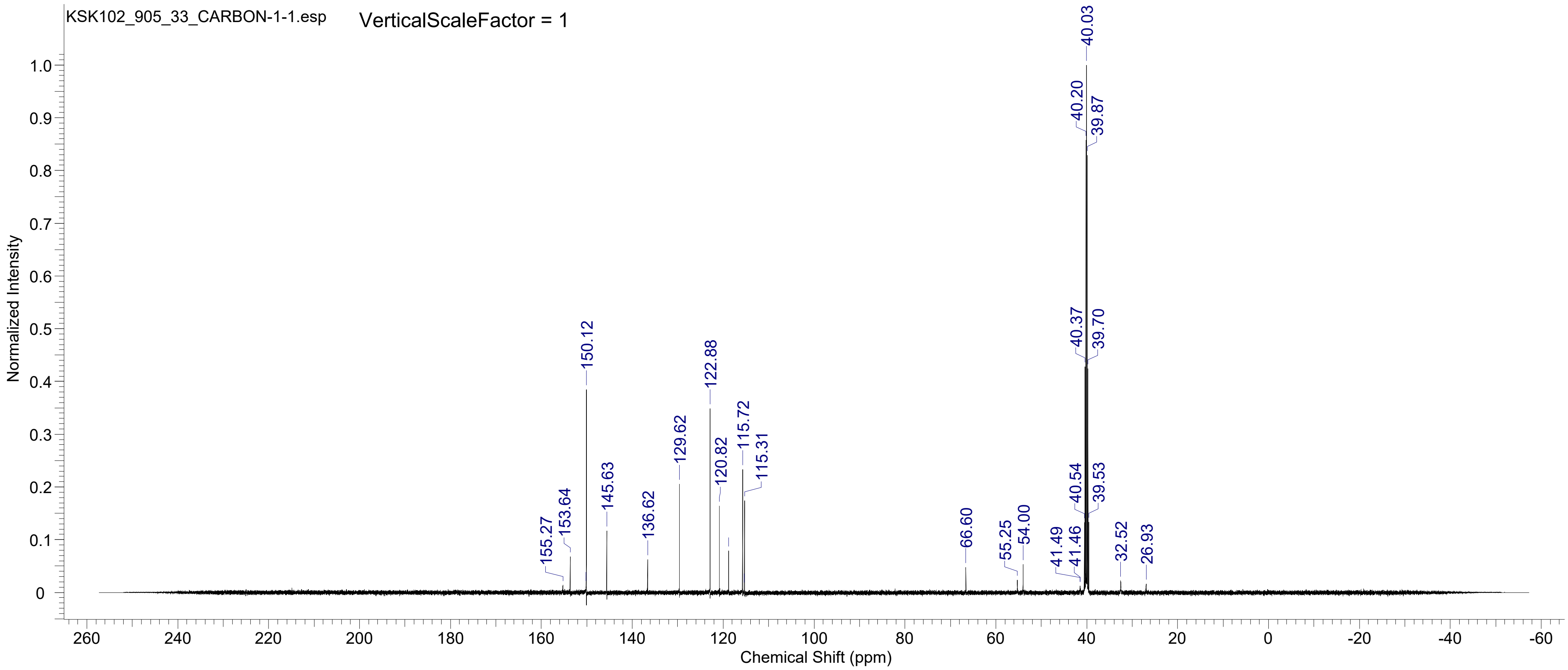

KSK102_905_33_CARBON-1-1.esp
VerticalScaleFactor = 1
Normalized Intensity
Chemical Shift (ppm)
155.27
153.64
150.12
145.63
136.62
129.62
122.88
120.82
115.72
115.31
66.60
55.25
54.00
41.49
41.46
40.54
40.37
40.20
40.03
39.87
39.70
39.53
32.52
26.93

Compound 15

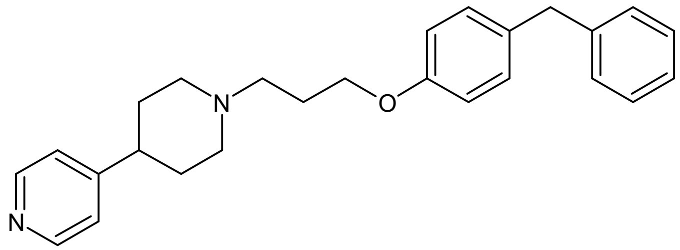

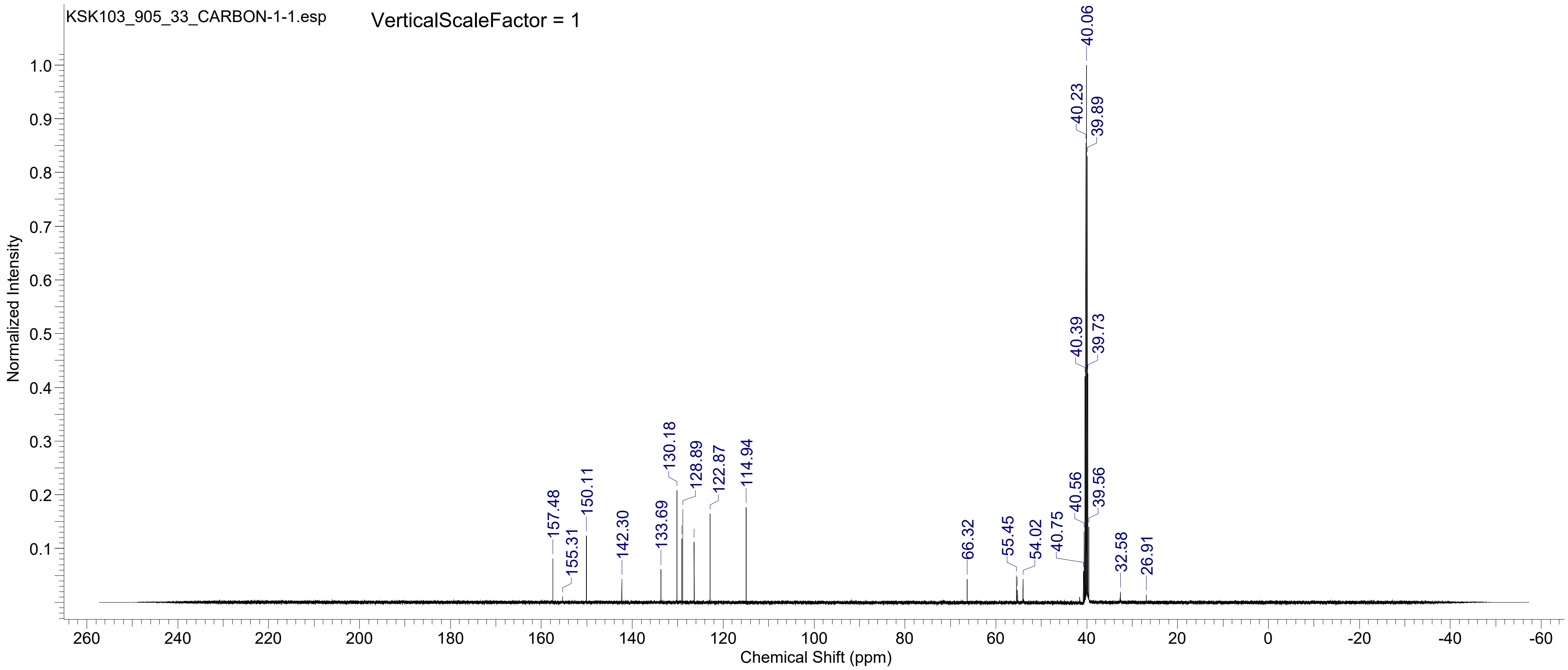

KSK103_905_33_CARBON-1-1.esp
VerticalScaleFactor = 1
157.48
155.31
150.11
142.30
133.69
130.18
128.89
122.87
114.94
66.32
55.45
54.02
40.75
40.56
40.39
40.23
40.06
39.89
39.73
39.56
32.58
26.91
Normalized Intensity
Chemical Shift (ppm)

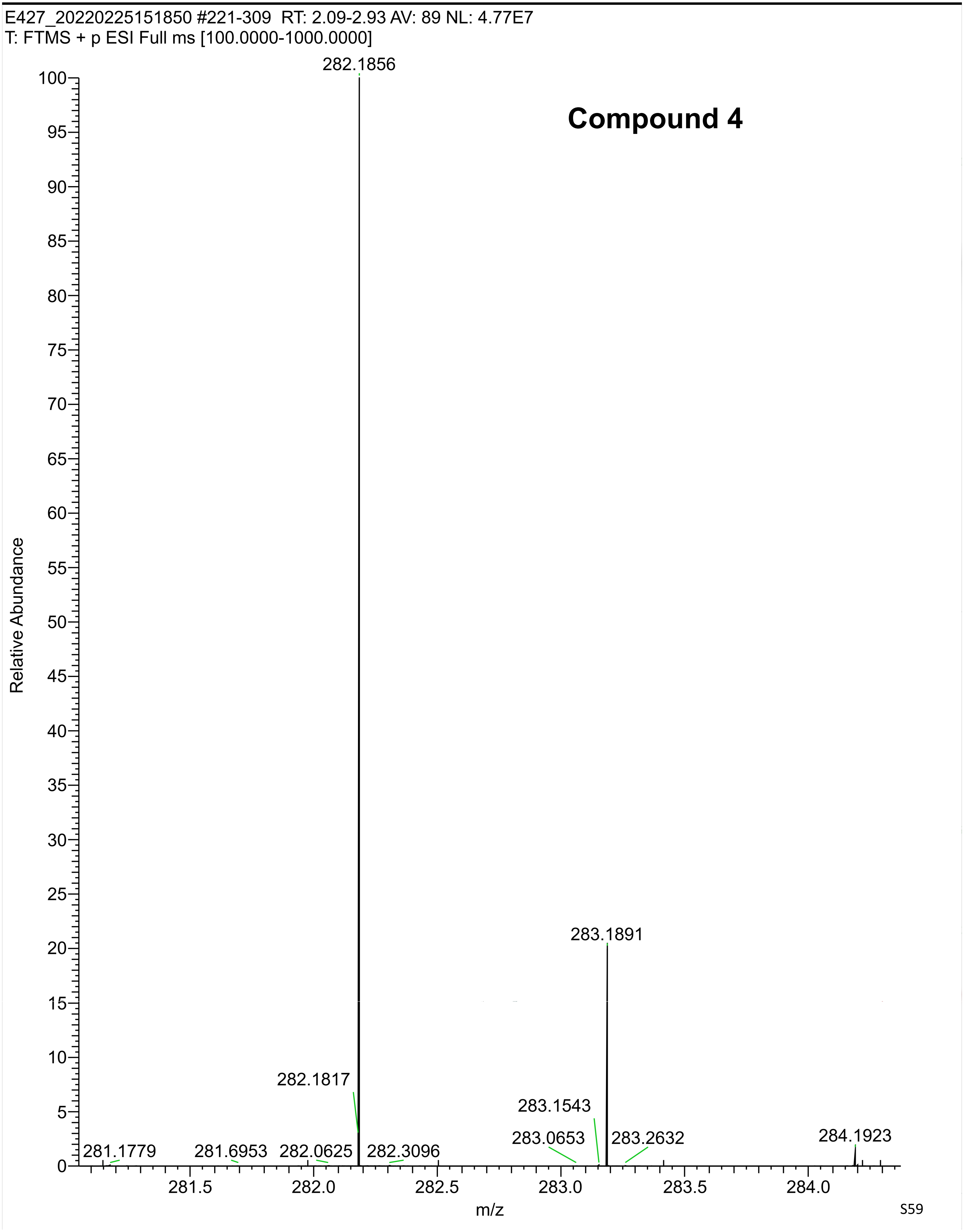
E427_20220225151850 #221-309 RT: 2.09-2.93 AV: 89 NL: 4.77E7
T: FTMS + p ESI Full ms [100.0000-1000.0000]
Compound 4
282.1856
283.1891
282.1817
283.1543
283.0653
283.2632
284.1923
281.1779
281.6953
282.0625
282.3096
Relative Abundance
m/z

E350_20220225151202 #275-359 RT: 2.59-3.39 AV: 85 NL: 3.55E6
T: FTMS + p ESI Full ms [100.0000-1000.0000]

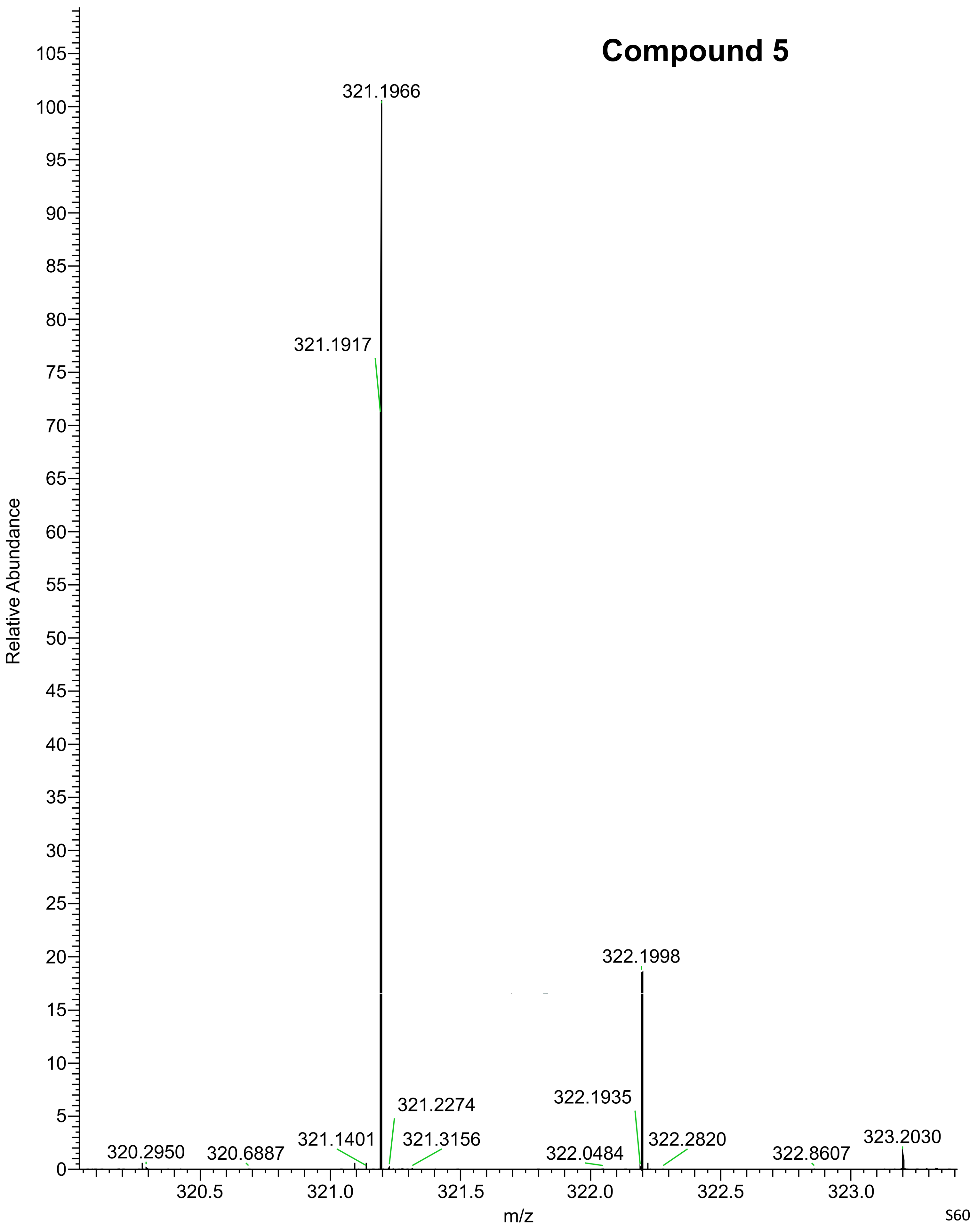

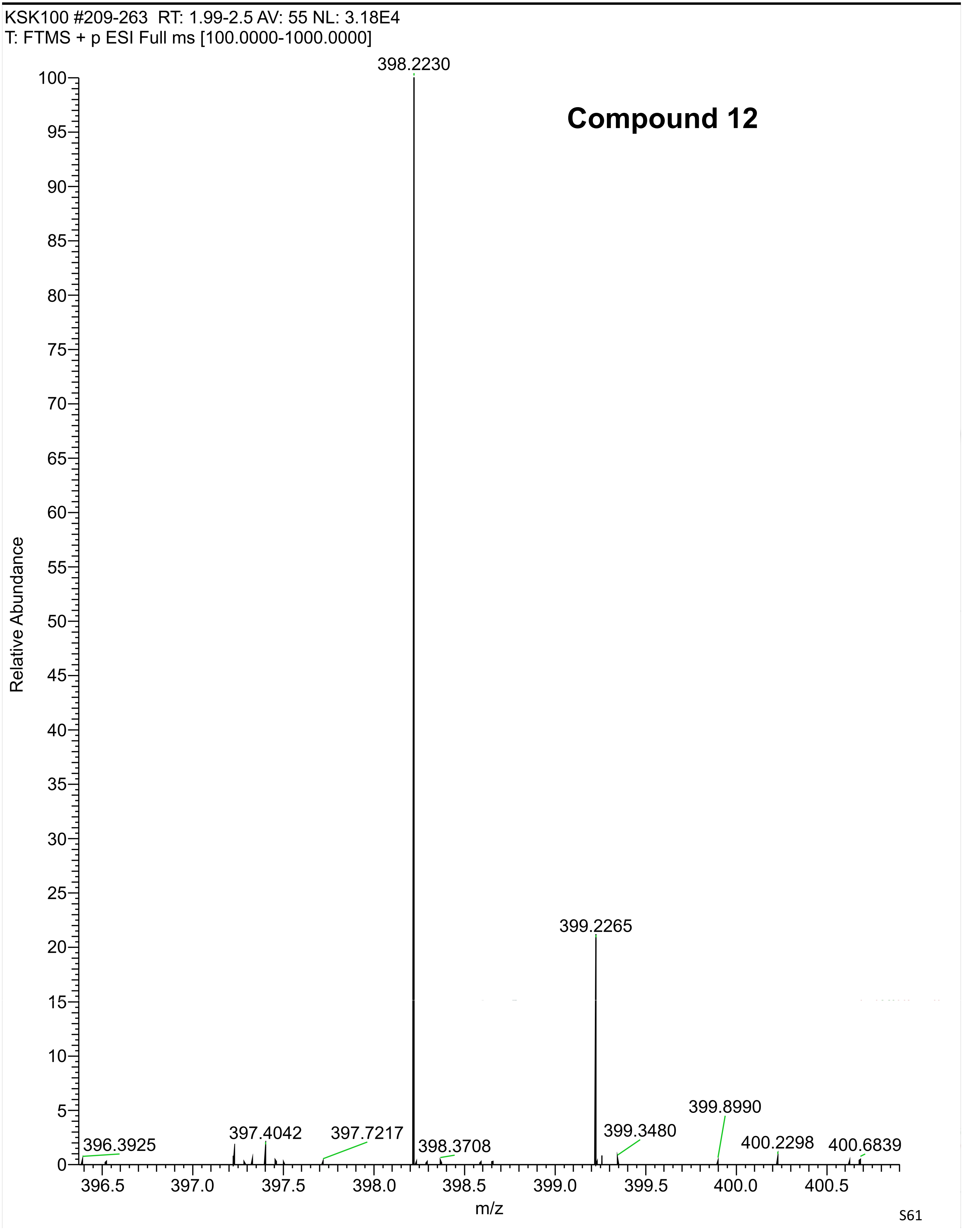
KSK100 #209-263 RT: 1.99-2.5 AV: 55 NL: 3.18E4
T: FTMS + p ESI Full ms [100.0000-1000.0000]
Compound 12
398.2230
399.2265
396.3925
397.4042
397.7217
398.3708
399.3480
399.8990
400.2298
400.6839
Relative Abundance
m/z

D:\DATA\Przemek\HRMS01\KSK100.raw

KSK101 #200-246 RT: 1.9-2.34 AV: 47 SB: 2 5.00 , 5.00 NL: 1.27E5
T: FTMS + p ESI Full ms [100.0000-1000.0000]

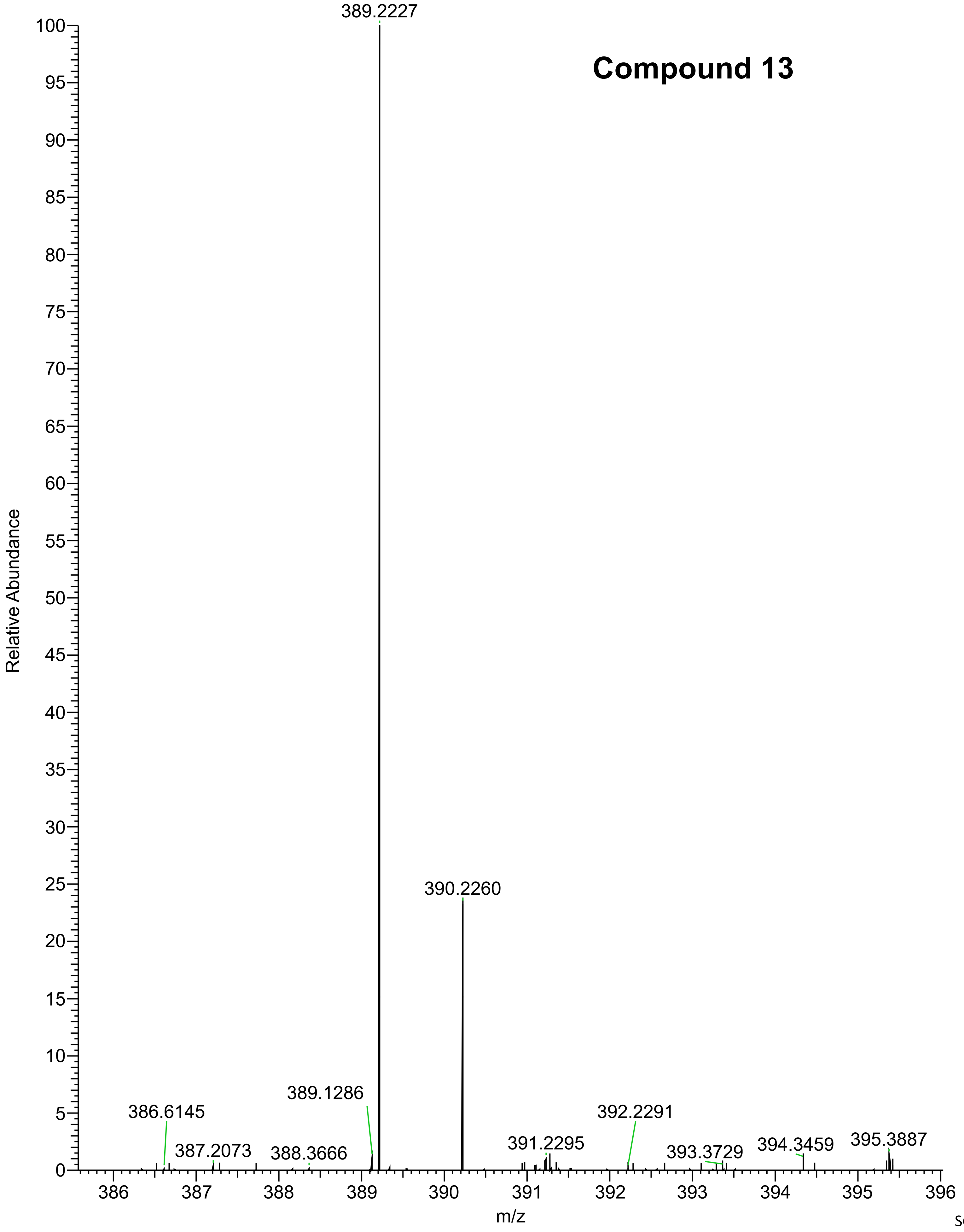

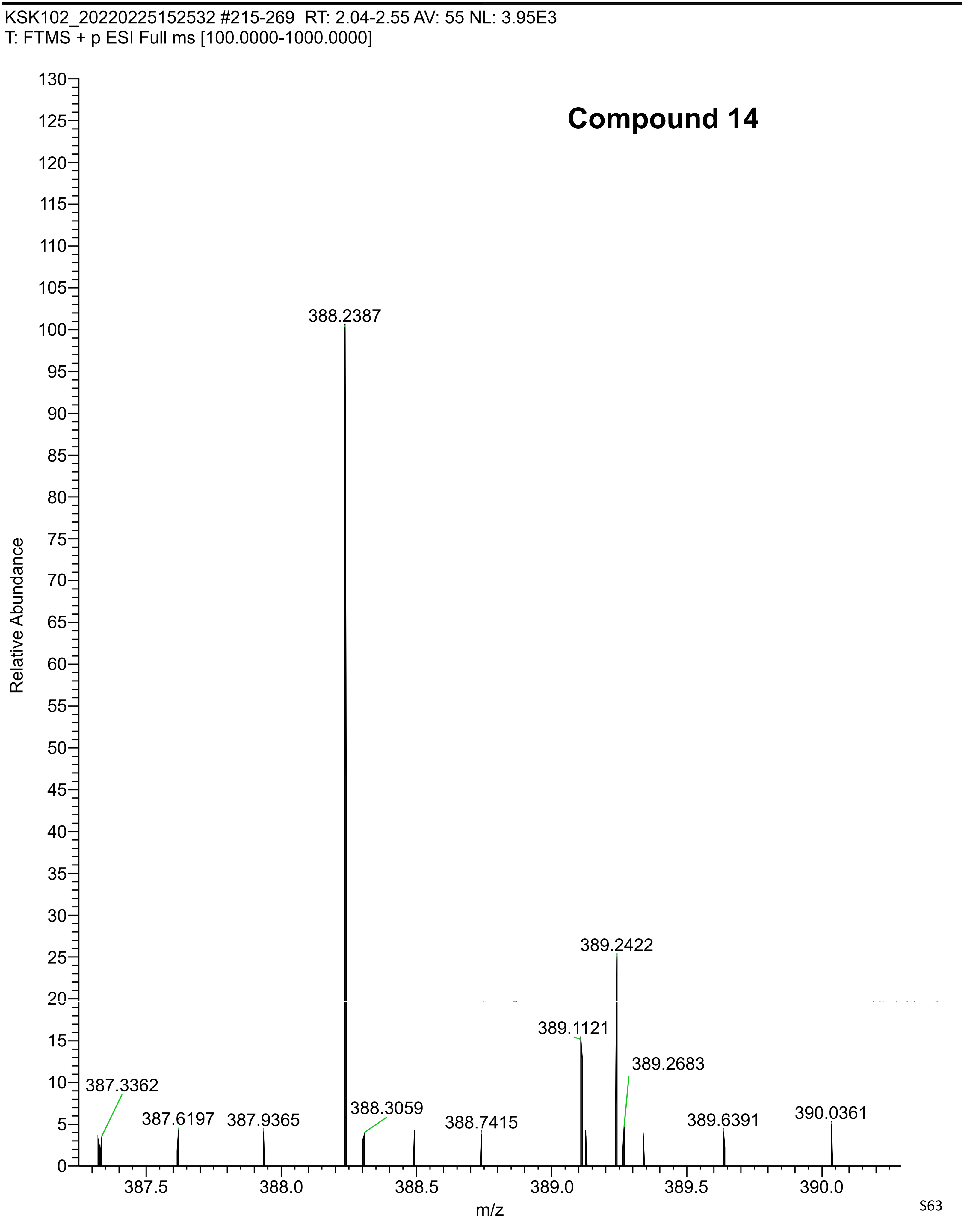
KSK102_20220225152532 #215-269 RT: 2.04-2.55 AV: 55 NL: 3.95E3
T: FTMS + p ESI Full ms [100.0000-1000.0000]
Compound 14
Relative Abundance
m/z
388.2387
389.2422
389.1121
389.2683
387.3362
387.6197
387.9365
388.3059
388.7415
389.6391
390.0361

KSK103 #213-286 RT: 2.03-2.72 AV: 74 NL: 5.36E4
T: FTMS + p ESI Full ms [100.0000-1000.0000]

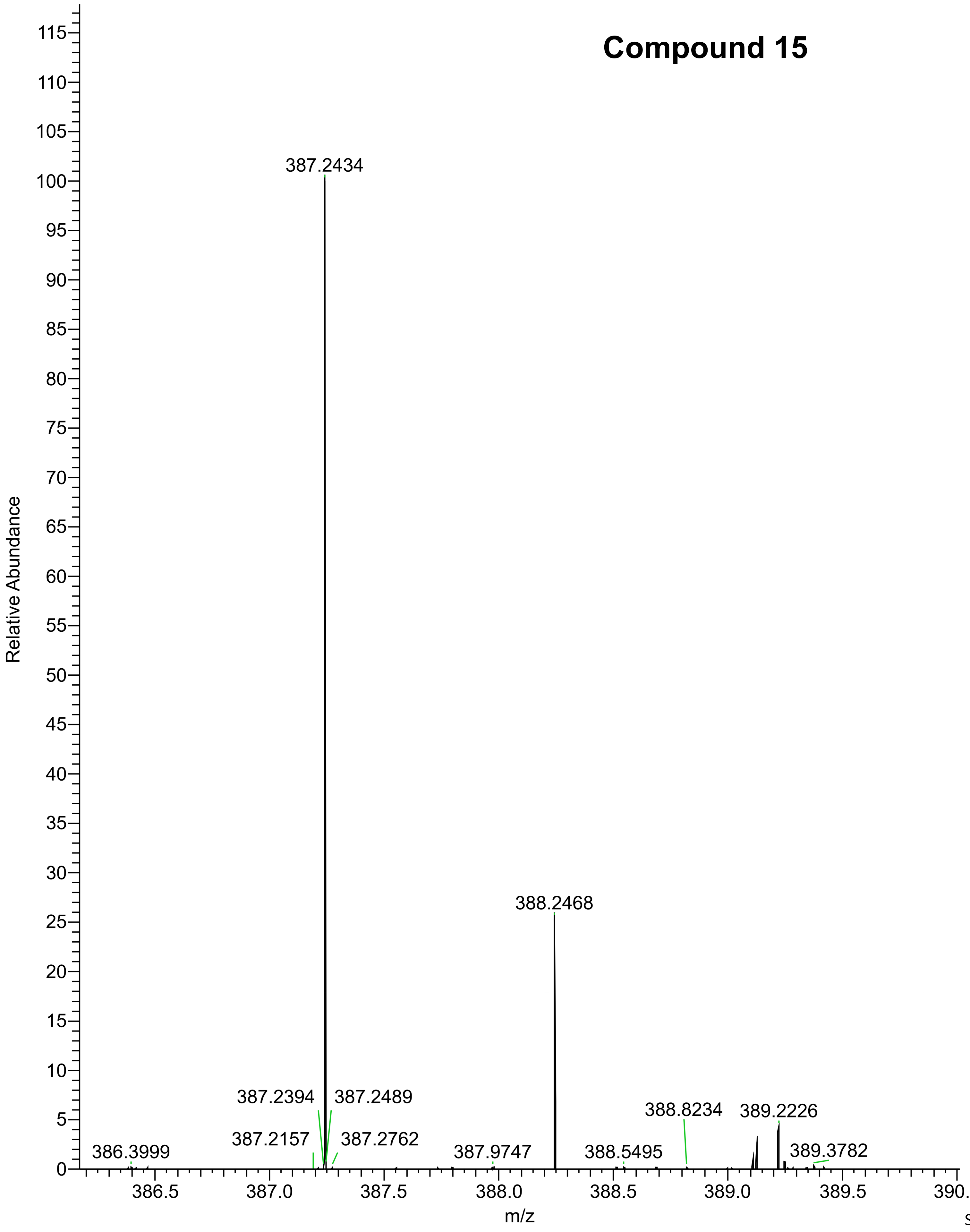

Screen

Compound 1

Data file: /LAF non-GMP/CPL LIBRARY/PURITY SCREENING/Results/2023/2023 04/2023 04 18/purity_screen_A2C_Monika Delis_20230418 144135_PHYSCHEM_03.rslt\2023-04-18 15-52-42+02-00-15.dx

Sample name: E380,FR18566015
Sample type: Sample
Instrument: PHYSCHEM_03
Location: D1F-D2
Injection date: 4/18/2023 3:53:23 PM
Injection: 1 of 1
Acq. method: PHYSCHEM5_CPL_0-100_A2C_C8_05_30_5.amx
Injection volume: 2.000
Analysis method: CPL_SKRIN_Zaktualizowany.pmx
Acq. operator: Monika Delis

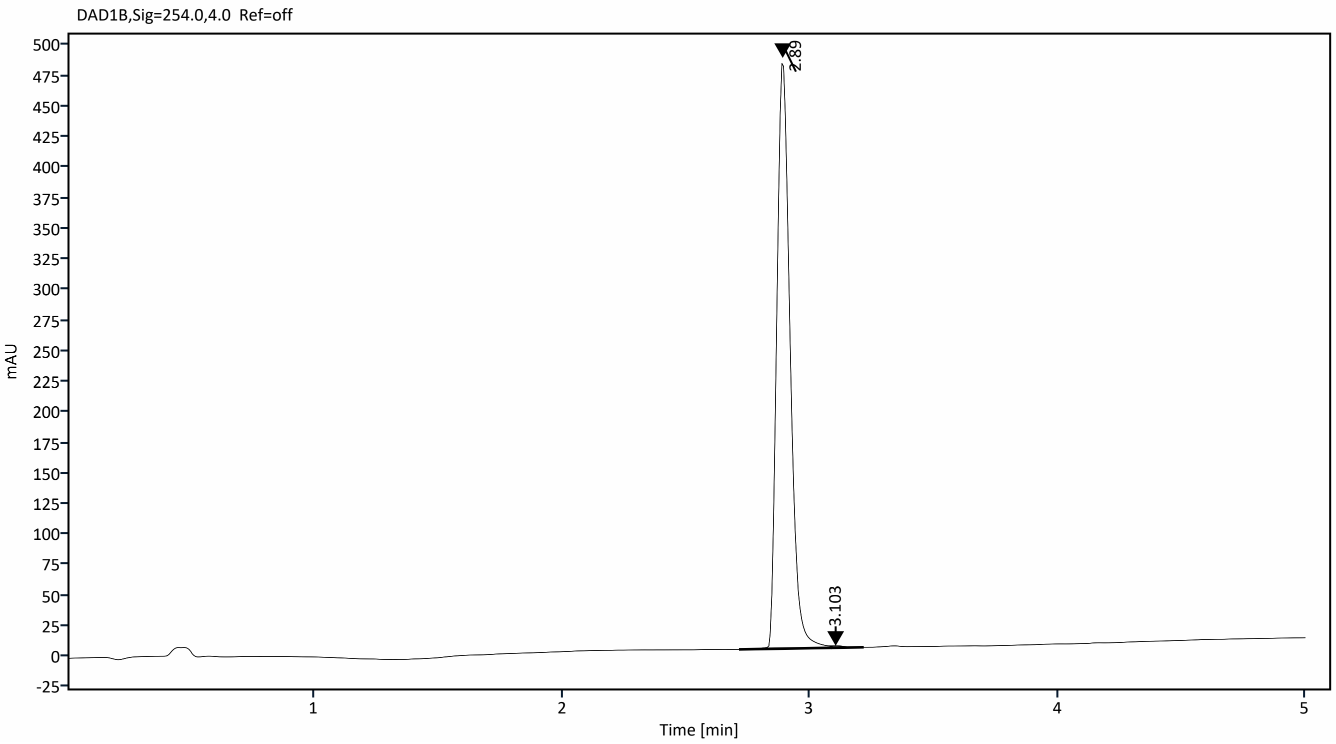

Signal: DAD1B,Sig=254.0,4.0 Ref=off

Name	RT [min]	RRT	Area	Area%	Height	Peak Tail Factor	Peak Asymmetry 10%
	2.890		1783.10 04	99.6652	483.25 16	1.20450	1.12280
	3.103		5.9890	0.3348	1.6104	3.14311	3.14311
		Sum	1789.08 94				

Screen
Compound 2

Data file: /LAF non-GMP/CPL LIBRARY/PURITY SCREENING/Results/2023/2023 04/2023 04 18/purity_screen_A2C_Monika Delis_20230418 144135_PHYSCHEM_03.rslt\2023-04-18 15-58-35+02-00-16.dx

Sample name: E382,FR16753640
Sample type: Sample
Instrument: PHYSCHEM_03
Location: D1F-E2
Injection date: 4/18/2023 3:59:17 PM
Injection: 1 of 1
Acq. method: PHYSCHEM5_CPL_0-100_A2C_C8_05_30_5.amx
Injection volume: 2.000
Analysis method: CPL_SKRIN_Zaktualizowany.pmx
Acq. operator: Monika Delis

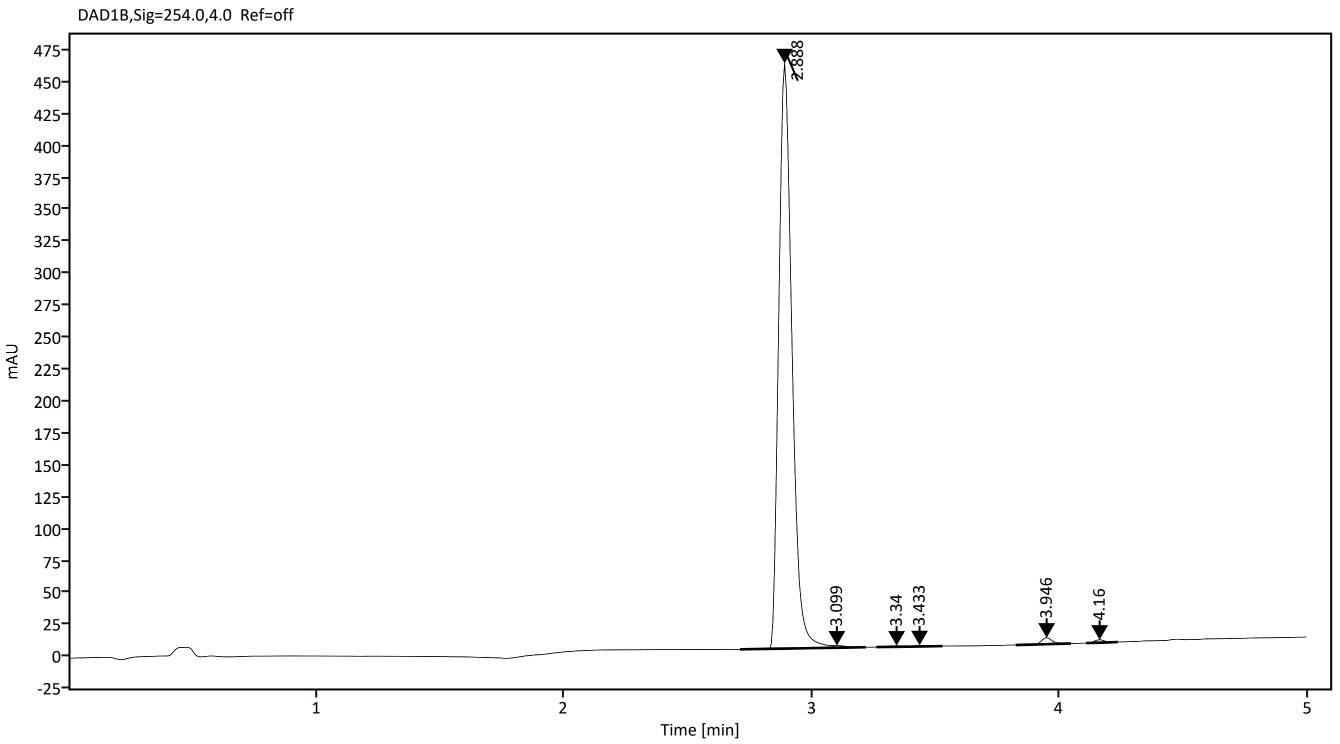

Signal: DAD1B,Sig=254.0,4.0 Ref=off

Name	RT [min]	RRT	Area	Area%	Height	Peak Tail Factor	Peak Asymmetry 10%
	2.888		1683.2402	97.9588	458.2108	1.29702	1.21696
	3.099		6.0788	0.3538	1.6592	3.60709	3.60709
	3.340		3.8460	0.2238	0.9198	0.93744	0.96397
	3.433		2.7404	0.1595	0.8983	1.63634	1.63634
	3.946		15.8049	0.9198	5.0312	1.63586	1.72263
	4.160		6.6044	0.3844	2.2855	1.47460	1.53033
		Sum	1718.3146				

Screen

Compound 3

Data file: /LAF non-GMP/CPL LIBRARY/PURITY SCREENING/Results/2023/2023 04/2023 04 18/purity_screen_A2C_Monika Delis_20230418 144135_PHYSCHEM_03.rslt\2023-04-18 15-17-22+02-00-09.dx

**Sample name:**	E381,FR18565972	**Sample type:**	Sample
**Instrument:**	PHYSCHEM_03	**Location:**	D1F-F1
**Injection date:**	4/18/2023 3:18:04 PM	**Injection:**	1 of 1
**Acq. method:**	PHYSCHEM5_CPL_0-100_A2C_C8_05_30_5.amx	**Injection volume:**	2.000
**Analysis method:**	CPL_SKRIN_Zaktualizowany.pmx	**Acq. operator:**	Monika Delis

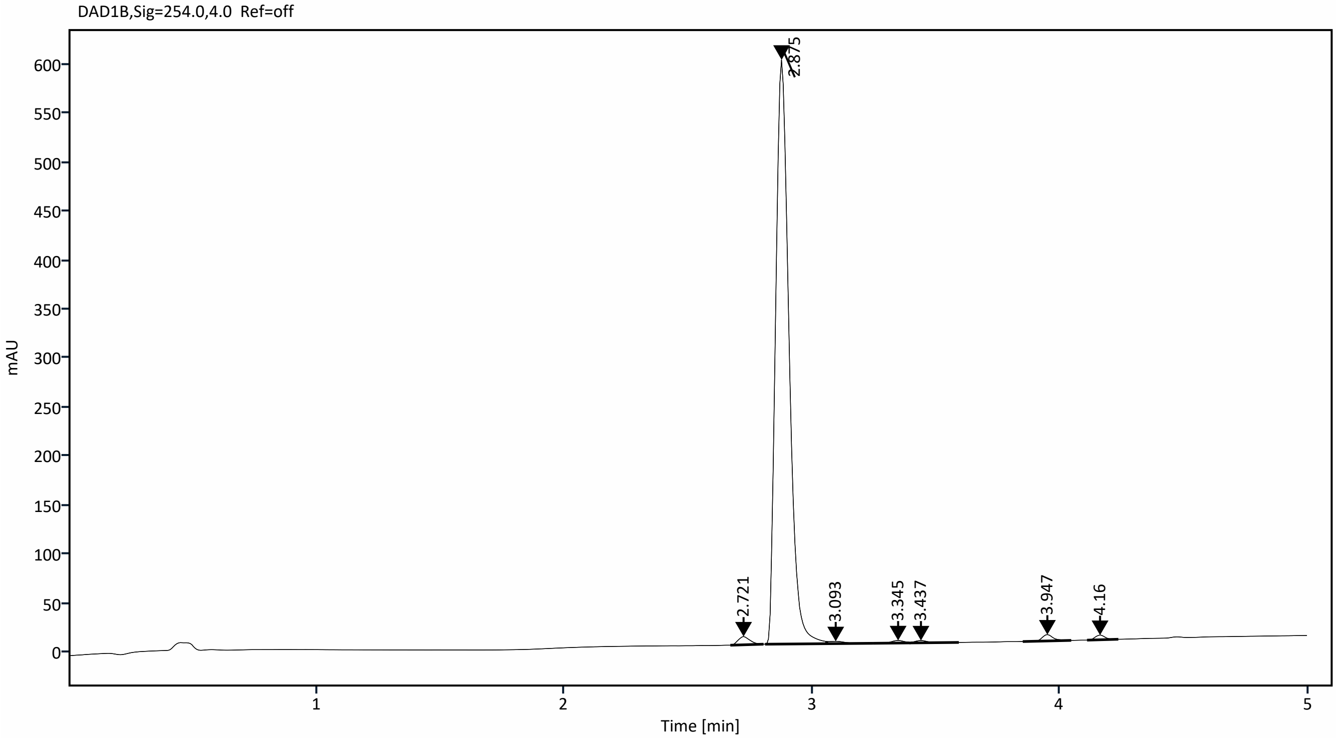

Signal: DAD1B,Sig=254.0,4.0 Ref=off

Name	RT [min]	RRT	Area	Area%	Height	Peak Tail Factor	Peak Asymmetry 10%
	2.721		27.8311	1.2043	8.4383	1.48125	1.36170
	2.875		2219.8825	96.0588	595.6153	1.30125	1.22261
	3.093		11.2036	0.4848	2.3268	2.45553	2.45553
	3.345		9.5539	0.4134	2.6734	0.86574	0.91957
	3.437		8.8692	0.3838	2.3819	2.36151	2.36151
	3.947		20.1335	0.8712	6.4586	1.69971	1.57023
	4.160		13.4888	0.5837	4.7635	1.45138	1.51246
		Sum	2310.9625				

Screen

Compound 4

Data file: /LAF non-GMP/CPL LIBRARY/PURITY SCREENING/Results/2023/2023 04/2023 04 18/purity_screen_A2C_Monika Delis_20230418 144135_PHYSCHEM_03.rslt\2023-04-18 16-04-29+02-00-17.dx

**Sample name:**	E427,FR18565374	**Sample type:**	Sample
**Instrument:**	PHYSCHEM_03	**Location:**	D1F-G2
**Injection date:**	4/18/2023 4:05:10 PM	**Injection:**	1 of 1
**Acq. method:**	PHYSCHEM5_CPL_0-100_A2C_C8_05_30_5.amx	**Injection volume:**	2.000
**Analysis method:**	CPL_SKRIN_Zaktualizowany.pmx	**Acq. operator:**	Monika Delis

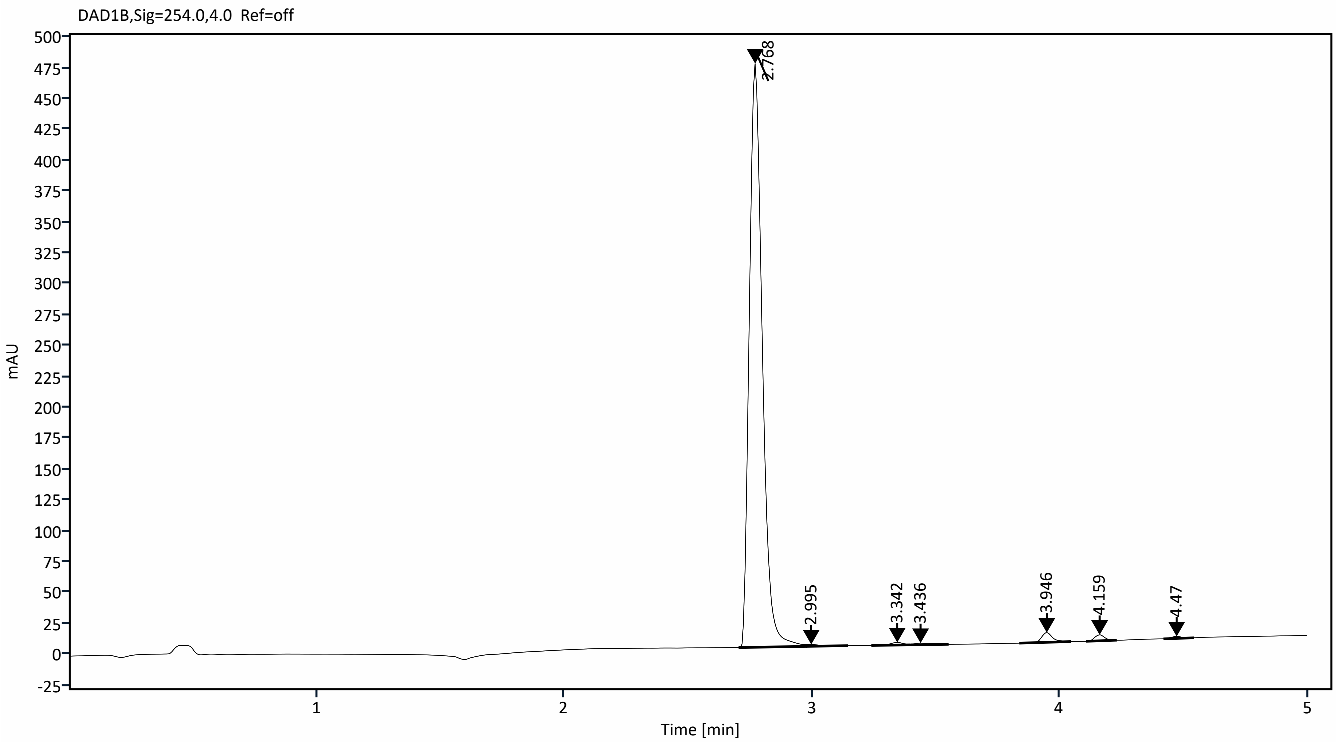

Signal: DAD1B,Sig=254.0,4.0 Ref=off

Name	RT [min]	RRT	Area	Area%	Height	Peak Tail Factor	Peak Asymmetry 10%
	2.768		1707.59 20	96.5794	472.45 71	1.29934	1.21149
	2.995		5.8079	0.3285	1.4781	6.63139	6.63139
	3.342		7.7897	0.4406	2.1418	0.95455	1.01526
	3.436		4.5628	0.2581	1.3469	1.93089	1.93089
	3.946		24.1283	1.3647	7.8147	1.59938	1.40547
	4.159		13.6605	0.7726	4.8310	1.35492	1.40293
	4.470		4.5292	0.2562	1.5861	1.60601	1.66066
		Sum	1768.07 04				

Screen   Compound 5

Data file: /LAF non-GMP/CPL LIBRARY/PURITY SCREENING/Results/2023/2023 04/2023 04 18/purity_screen_A2C_Monika Delis_20230418 144135_PHYSCHEM_03.rslt\2023-04-18 16-10-21+02-00-18.dx

Sample name: E350,FR18565917
Sample type: Sample
Instrument: PHYSCHEM_03
Location: D1F-H2
Injection date: 4/18/2023 4:11:03 PM
Injection: 1 of 1
Acq. method: PHYSCHEM5_CPL_0-100_A2C_C8_05_30_5.amx
Injection volume: 2.000
Analysis method: CPL_SKRIN_Zaktualizowany.pmx
Acq. operator: Monika Delis

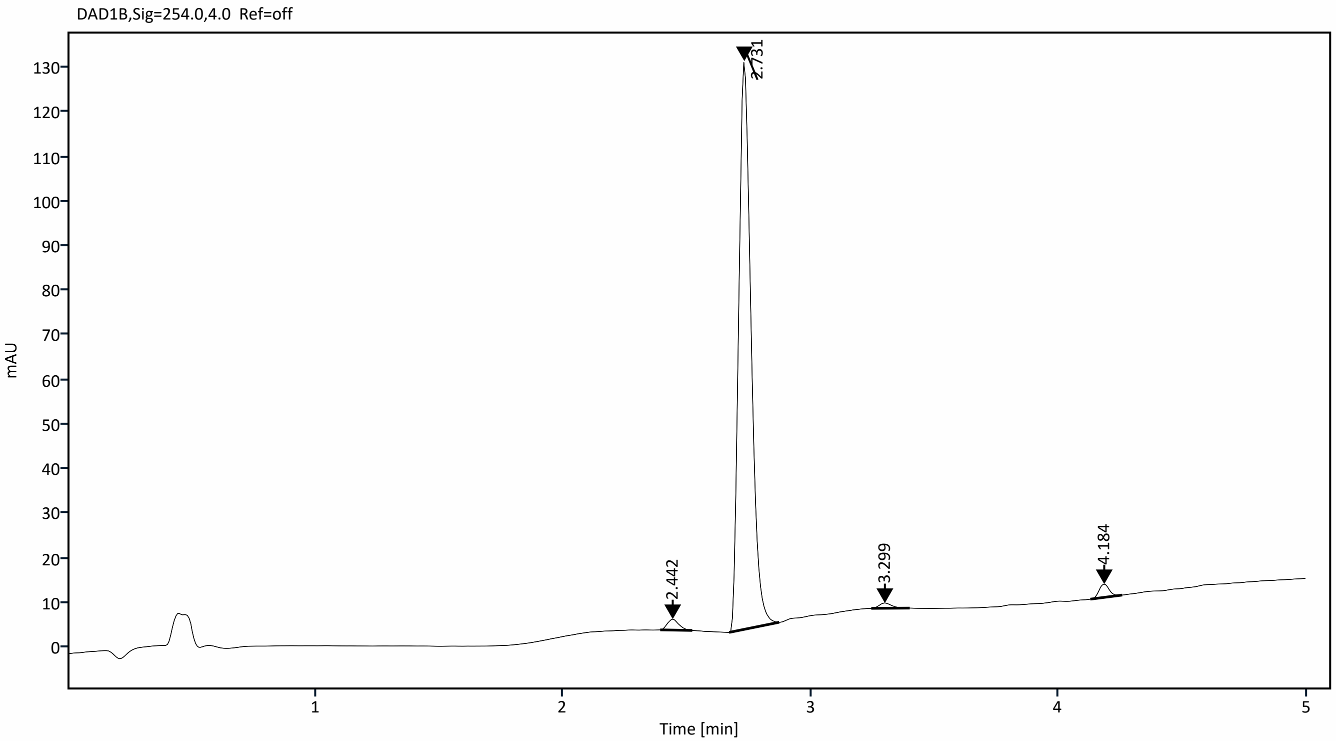

Signal: DAD1B,Sig=254.0,4.0 Ref=off

Name	RT [min]	RRT	Area	Area%	Height	Peak Tail Factor	Peak Asymmetry 10%
	2.442		7.7504	1.6674	2.4636	1.13326	1.12034
	2.731		444.4937	95.6279	127.4687	1.33268	1.24594
	3.299		4.1076	0.8837	1.1753	1.47010	1.34467
	4.184		8.4640	1.8209	2.9743	1.54846	1.62352
		Sum	464.8157				

Screen

Compound 6

Data file: /LAF non-GMP/CPL LIBRARY/PURITY SCREENING/Results/2023/2023 04/2023 04 18/purity_screen_A2C_Monika Delis_20230418 144135_PHYSCHEM_03.rslt\2023-04-18 16-16-13+02-00-19.dx

Method:

Sample name: E353,FR18565886

Sample type: Sample

Instrument: PHYSCHEM_03

Injection date: 4/18/2023 4:16:55 PM

Acq. method: PHYSCHEM5_CPL_0-100_A2C_C8_05_30_5.amx

Analysis method: CPL_SKRIN_Zaktualizowany.pmx

Location: D1F-A3

Injection: 1 of 1

Injection volume: 2.000

Acq. operator: Monika Delis

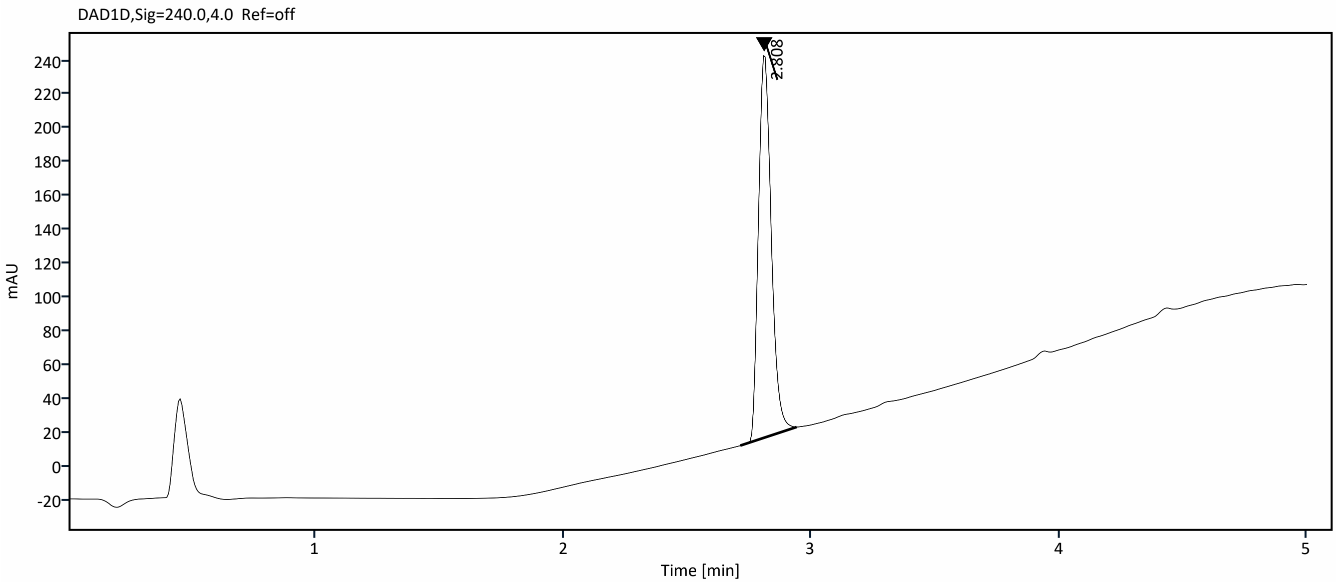

Signal: DAD1D,Sig=240.0,4.0 Ref=off

Name	RT [min]	RRT	Area	Area%	Height	Peak Tail Factor	Peak Asymmetry 10%
	2.808		812.6963	100.00 00	227.84 59	1.26230	1.14361
		Sum	812.6963				

Screen Compound 7

Data file: /LAF non-GMP/CPL LIBRARY/PURITY SCREENING/Results/2023/2023 04/2023 04 18/purity_screen_A2C_Monika Delis_20230418 144135_PHYSCHEM_03.rslt\2023-04-18 17-09-14+02-00-28.dx

Sample name: E357,FR16753598
Sample type: Sample
Instrument: PHYSCHEM_03
Location: D1F-B4
Injection date: 4/18/2023 5:09:55 PM
Injection: 1 of 1
Acq. method: PHYSCHEM5_CPL_0-100_A2C_C8_05_30_5.amx
Injection volume: 2.000
Analysis method: CPL_SKRIN_Zaktualizowany.pmx
Acq. operator: Monika Delis

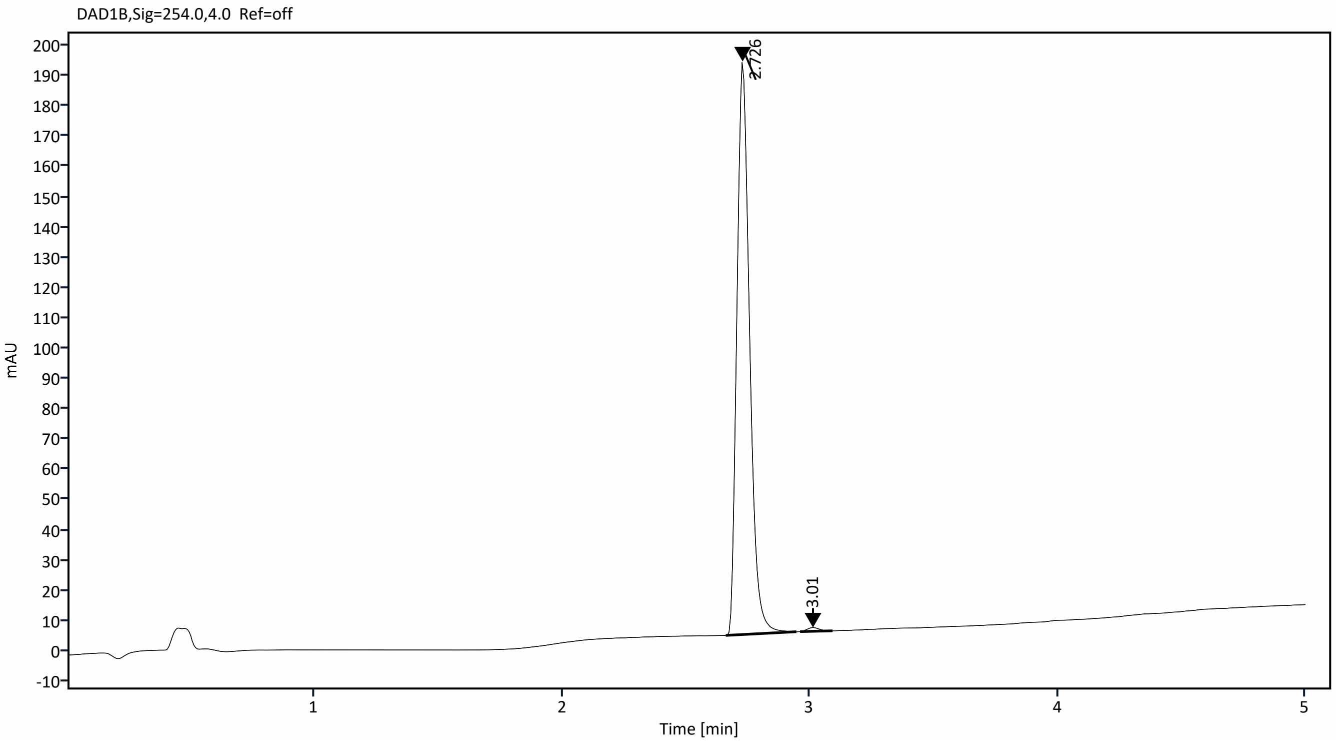

Signal: DAD1B,Sig=254.0,4.0 Ref=off

Name	RT [min]	RRT	Area	Area%	Height	Peak Tail Factor	Peak Asymmetry 10%
	2.726		681.9849	99.4057	189.30 11	1.31020	1.22891
	3.010		4.0771	0.5943	1.2555	1.42179	1.48166
		Sum	686.0621				

Screen

Compound 8

Data file: /LAF non-GMP/CPL LIBRARY/PURITY SCREENING/Results/2023/2023 04/2023 04 18/purity_screen_A2C_Monika Delis_20230418 144135_PHYSCHEM_03.rslt\2023-04-18 17-15-07+02-00-29.dx

Method:

Sample name: E423,FR18565878

Sample type: Sample

Instrument: PHYSCHEM_03

Location: D1F-C4

Injection date: 4/18/2023 5:15:49 PM

Injection: 1 of 1

Acq. method: PHYSCHEM5_CPL_0-100_A2C_C8_05_30_5.amx

Injection volume: 2.000

Analysis method: CPL_SKRIN_Zaktualizowany.pmx

Acq. operator: Monika Delis

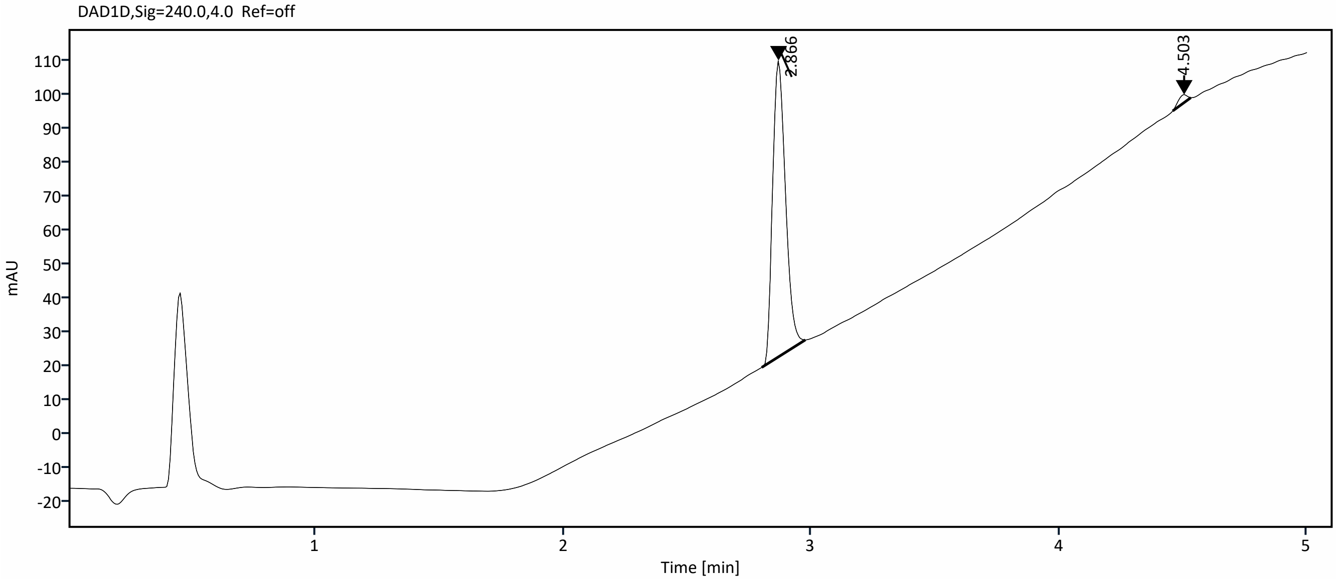

Signal: DAD1D,Sig=240.0,4.0 Ref=off

Name	RT [min]	RRT	Area	Area%	Height	Peak Tail Factor	Peak Asymmetry 10%
	2.866		314.0104	98.0164	87.4546	1.71589	1.33308
	4.503		6.3549	1.9836	2.3446	0.94636	0.95848
		Sum	320.3653				

Screen

Compound 9

Data file: /LAF non-GMP/CPL LIBRARY/PURITY SCREENING/Results/2023/2023 04/2023 04 18/purity_screen_A2C_Monika Delis_20230418 144135_PHYSCHEM_03.rslt\2023-04-18 17-21-01+02-00-30.dx

**Sample name:**	E384,FR18566075	**Sample type:**	Sample
**Instrument:**	PHYSCHEM_03	**Location:**	D1F-D4
**Injection date:**	4/18/2023 5:21:43 PM	**Injection:**	1 of 1
**Acq. method:**	PHYSCHEM5_CPL_0-100_A2C_C8_05_30_5.amx	**Injection volume:**	2.000
**Analysis method:**	CPL_SKRIN_Zaktualizowany.pmx	**Acq. operator:**	Monika Delis

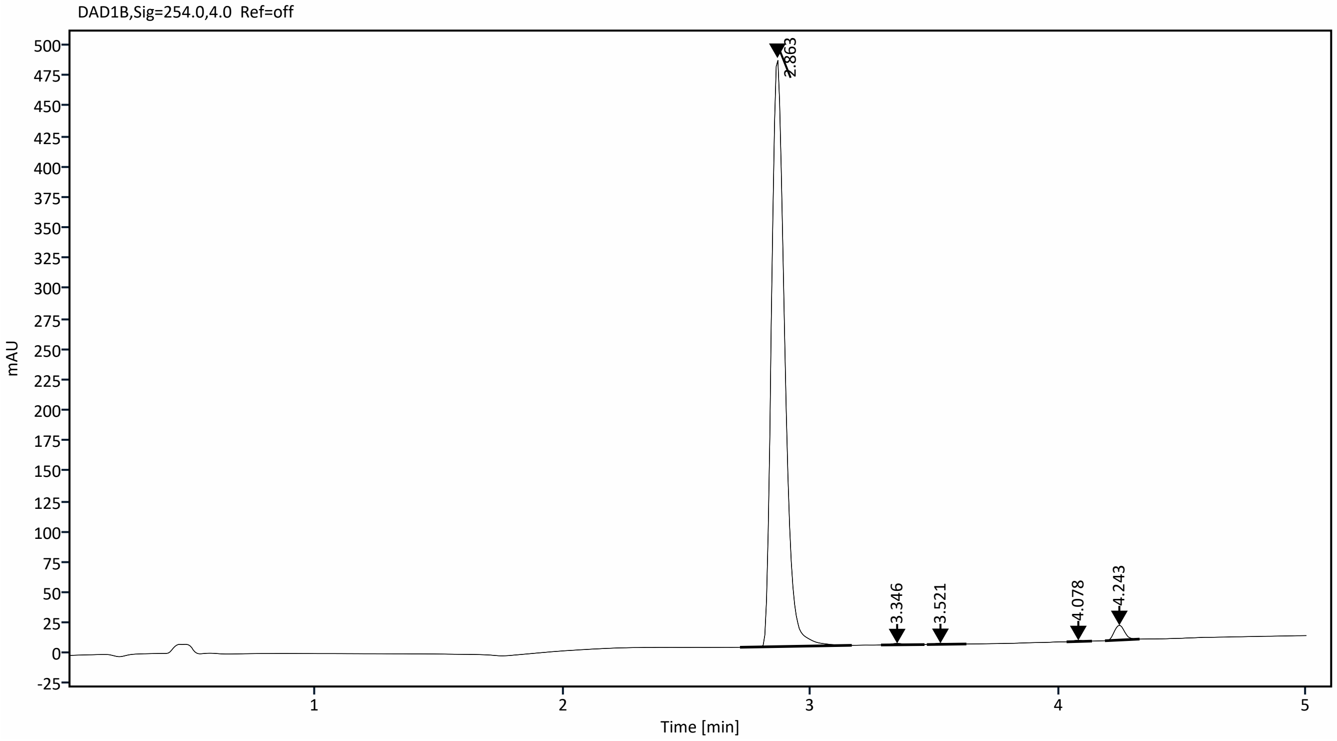

Signal: DAD1B,Sig=254.0,4.0 Ref=off

Name	RT [min]	RRT	Area	Area%	Height	Peak Tail Factor	Peak Asymmetry 10%
	2.863		1793.1006	97.5350	483.9614	1.22550	1.15001
	3.346		4.1350	0.2249	1.1740	1.84988	1.94717
	3.521		3.4922	0.1900	0.9594	2.01049	2.11218
	4.078		2.5077	0.1364	0.9463	1.33947	1.37348
	4.243		35.1822	1.9137	12.1591	1.49158	1.17291
		Sum	1838.4177				

Screen

Compound 10

Data file: /LAF non-GMP/CPL LIBRARY/PURITY SCREENING/Results/2023/2023 04/2023 04 18/purity_screen_A2C_Monika Delis_20230418 144135_PHYSCHEM_03.rslt\2023-04-18 17-26-54+02-00-31.dx

Sample name: E150,FR18565408
Sample type: Sample
Instrument: PHYSCHEM_03
Location: D1F-E4
Injection date: 4/18/2023 5:27:36 PM
Injection: 1 of 1
Acq. method: PHYSCHEM5_CPL_0-100_A2C_C8_05_30_5.amx
Injection volume: 2.000
Analysis method: CPL_SKRIN_Zaktualizowany.pmx
Acq. operator: Monika Delis

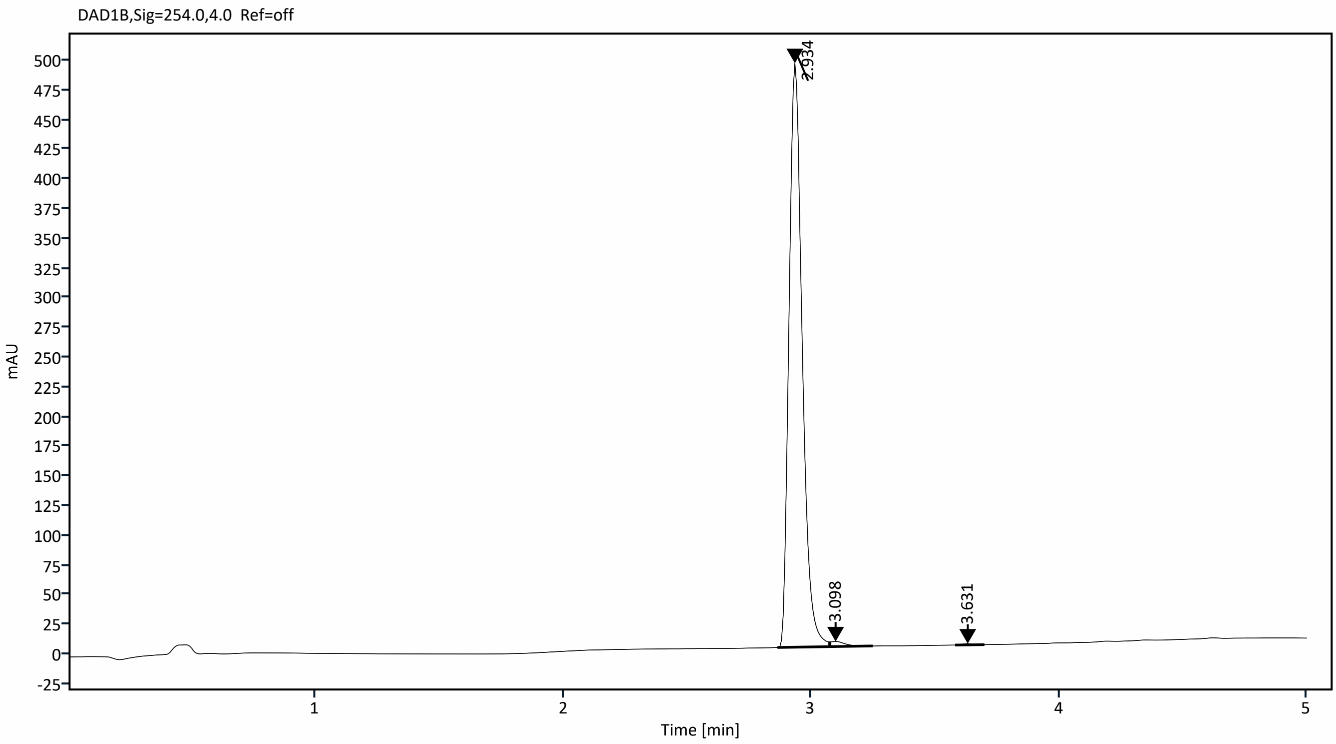

Signal: DAD1B,Sig=254.0,4.0 Ref=off

Name	RT [min]	RRT	Area	Area%	Height	Peak Tail Factor	Peak Asymmetry 10%
	2.934		1833.2958	98.8801	491.9770	1.29635	1.22229
	3.098		17.6976	0.9545	4.2801	3.36634	3.36634
	3.631		3.0658	0.1654	1.0476	1.36042	1.40396
		Sum	1854.0592				

Screen   Compound 11

Data file: /LAF non-GMP/CPL LIBRARY/PURITY SCREENING/Results/2023/2023 04/2023 04 18/purity_screen_A2C_Monika Delis_20230418 144135_PHYSCHEM_03.rslt\2023-04-18 16-45-41+02-00-24.dx

Sample name: E146,FR16753616
Sample type: Sample
Instrument: PHYSCHEM_03
Location: D1F-F3
Injection date: 4/18/2023 4:46:23 PM
Injection: 1 of 1
Acq. method: PHYSCHEM5_CPL_0-100_A2C_C8_05_30_5.amx
Injection volume: 2.000
Analysis method: CPL_SKRIN_Zaktualizowany.pmx
Acq. operator: Monika Delis

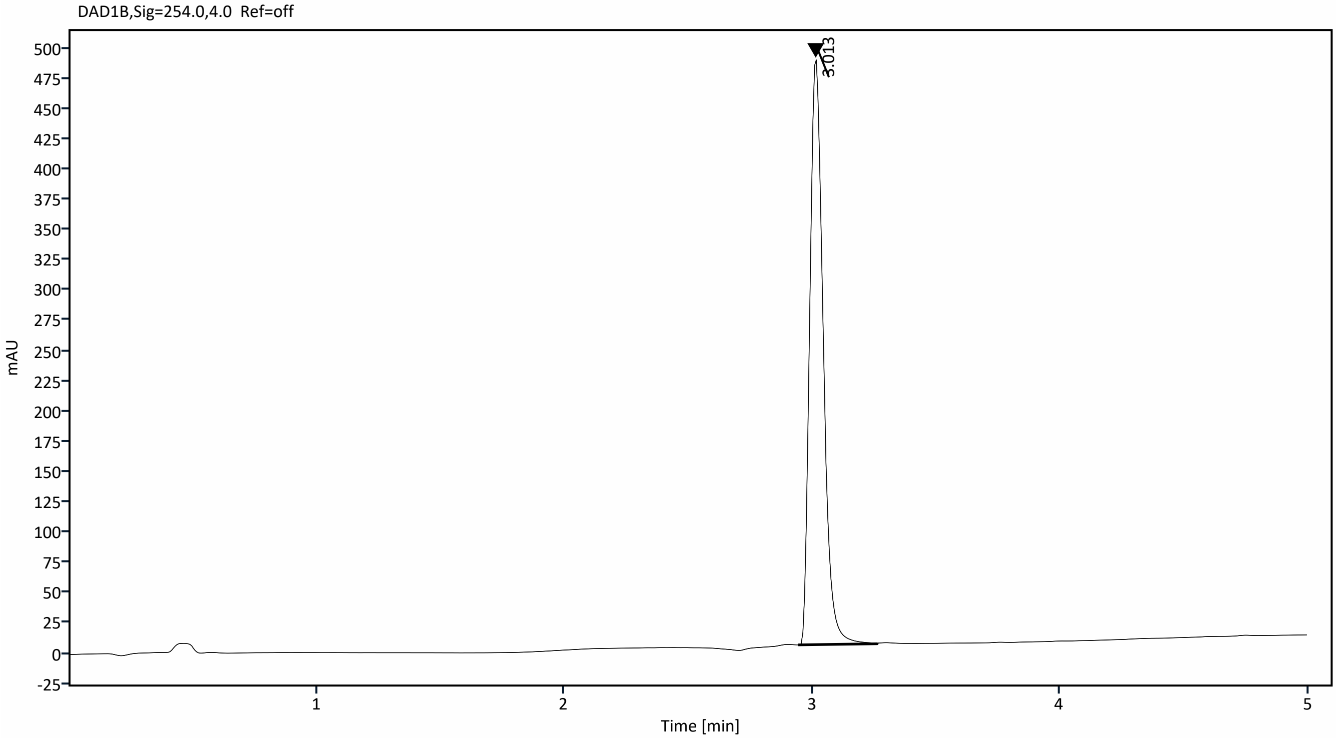

Signal: DAD1B,Sig=254.0,4.0 Ref=off

Name	RT [min]	RRT	Area	Area%	Height	Peak Tail Factor	Peak Asymmetry 10%
	3.013		1826.7414	100.0000	484.6352	1.24170	1.16470
		Sum	1826.7414				

Screen

Compound 12

Data file: /LAF non-GMP/CPL LIBRARY/PURITY SCREENING/Results/2023/2023 04/2023 04 18/purity_screen_A2C_Monika Delis_20230418 144135_PHYSCHEM_03.rslt\2023-04-18 17-38-40+02-00-33.dx

**Sample name:**	KSK100,FR18565835	**Sample type:**	Sample
**Instrument:**	PHYSCHEM_03	**Location:**	D1F-G4
**Injection date:**	4/18/2023 5:39:21 PM	**Injection:**	1 of 1
**Acq. method:**	PHYSCHEM5_CPL_0-100_A2C_C8_05_30_5.amx	**Injection volume:**	2.000
**Analysis method:**	CPL_SKRIN_Zaktualizowany.pmx	**Acq. operator:**	Monika Delis

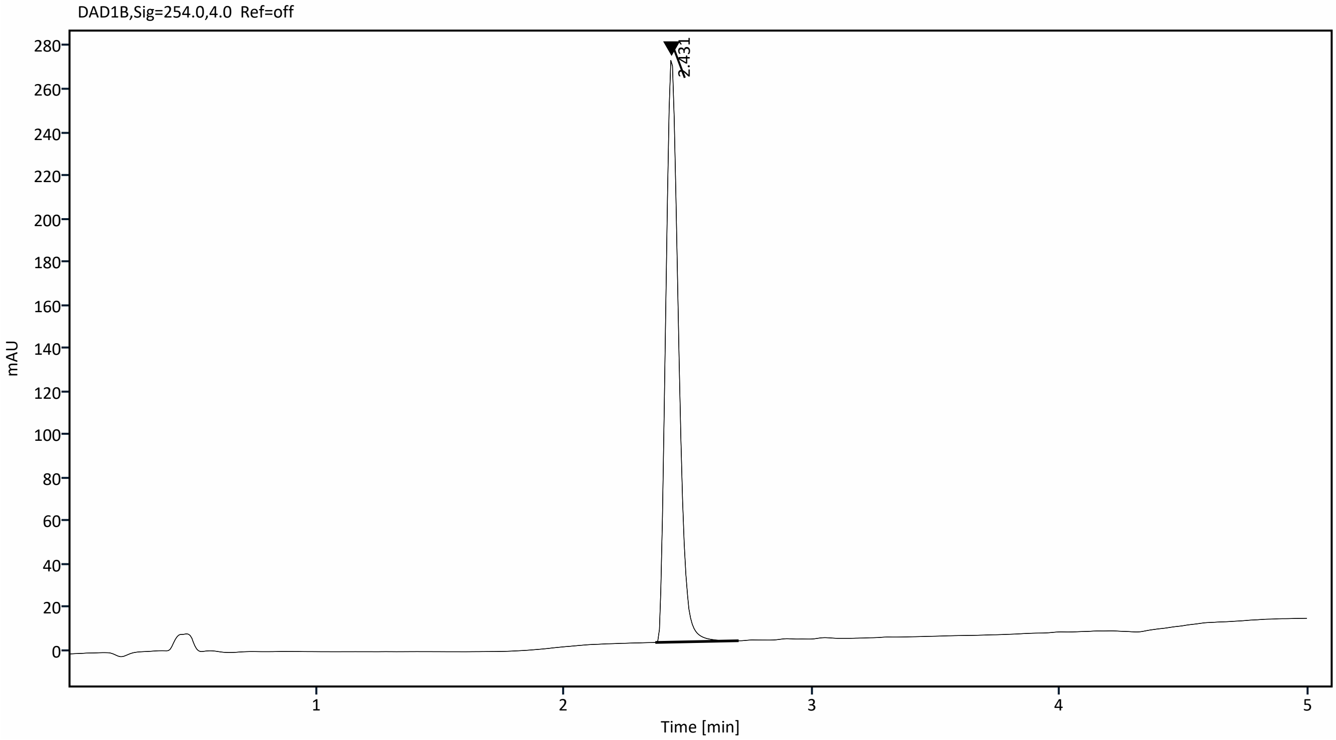

Signal: DAD1B,Sig=254.0,4.0 Ref=off

Name	RT [min]	RRT	Area	Area%	Height	Peak Tail Factor	Peak Asymmetry 10%
	2.431		965.5887	100.0000	271.3364	1.34161	1.27233
		Sum	965.5887				

Screen   Compound 13

Data file: /LAF non-GMP/CPL LIBRARY/PURITY SCREENING/Results/2023/2023 04/2023 04 18/purity_screen_A2C_Monika Delis_20230418 144135_PHYSCHEM_03.rslt\2023-04-18 16-57-27+02-00-26.dx

Method:

Sample name: KSK101,FR16753592

Sample type: Sample

Instrument: PHYSCHEM_03

Location: D1F-H3

Injection date: 4/18/2023 4:58:09 PM

Injection: 1 of 1

Acq. method: PHYSCHEM5_CPL_0-100_A2C_C8_05_30_5.amx

Injection volume: 2.000

Analysis method: CPL_SKRIN_Zaktualizowany.pmx

Acq. operator: Monika Delis

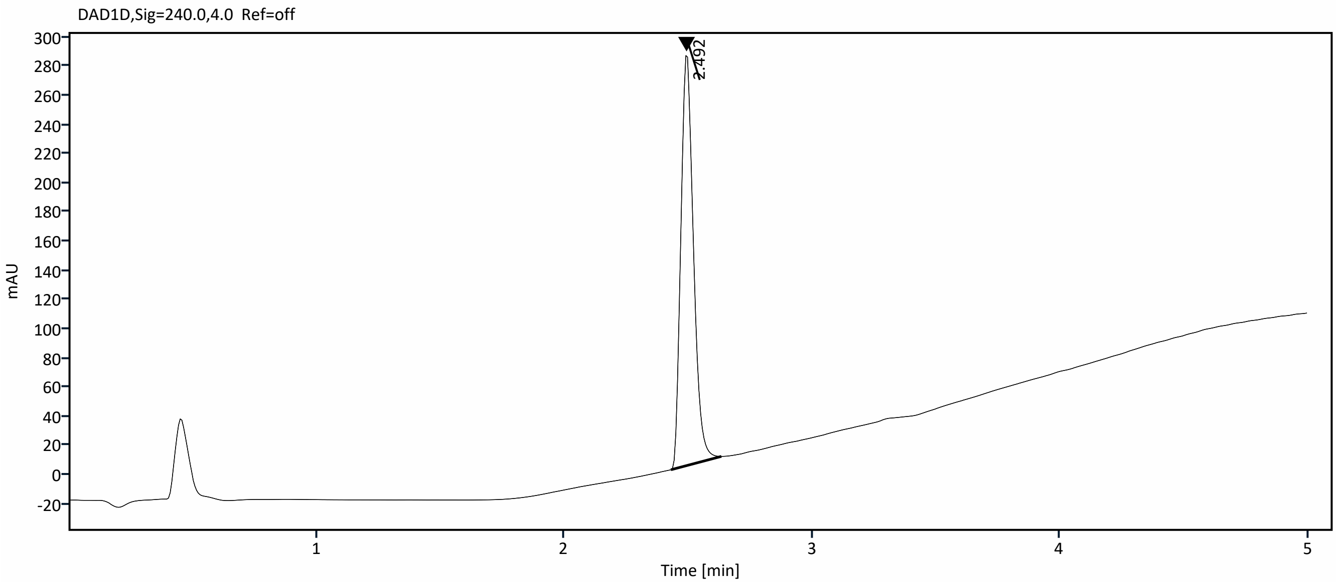

Signal: DAD1D,Sig=240.0,4.0 Ref=off

Name	RT [min]	RRT	Area	Area%	Height	Peak Tail Factor	Peak Asymmetry 10%
	2.492		1006.5670	100.0000	283.8744	1.22305	1.12666
		Sum	1006.5670				

Screen **Compound 14**

Data file: /LAF non-GMP/CPL LIBRARY/PURITY SCREENING/Results/2023/2023 04/2023 04 18/purity_screen_A2C_Monika Delis_20230418 144135_PHYSCHEM_03.rslt\2023-04-18 17-50-28+02-00-35.dx

Sample name: KSK102,FR18565868
Sample type: Sample
Instrument: PHYSCHEM_03
Location: D1F-A5
Injection date: 4/18/2023 5:51:09 PM
Injection: 1 of 1
Acq. method: PHYSCHEM5_CPL_0-100_A2C_C8_05_30_5.amx
Injection volume: 2.000
Analysis method: CPL_SKRIN_Zaktualizowany.pmx
Acq. operator: Monika Delis

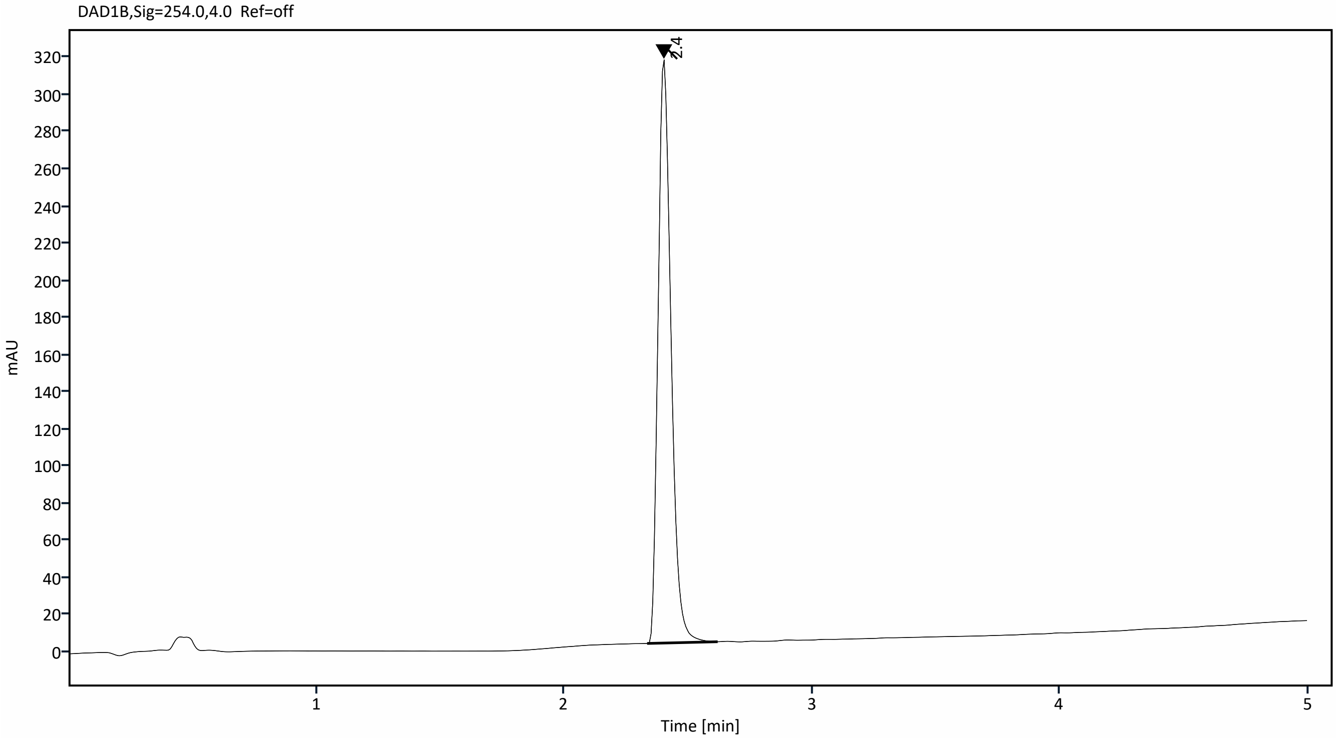

Signal: DAD1B,Sig=254.0,4.0 Ref=off

Name	RT [min]	RRT	Area	Area%	Height	Peak Tail Factor	Peak Asymmetry 10%
	2.400		1151.3182	100.0000	314.7400	1.22236	1.15516
		Sum	1151.3182				

Screen **Compound 15**

Data file: /LAF non-GMP/CPL LIBRARY/PURITY SCREENING/Results/2023/2023 04/2023 04 18/purity_screen_A2C_Monika Delis_20230418 144135_PHYSCHEM_03.rslt\2023-04-18 17-56-22+02-00-36.dx

**Sample name:**	KSK103,FR18565431	**Sample type:**	Sample
**Instrument:**	PHYSCHEM_03	**Location:**	D1F-B5
**Injection date:**	4/18/2023 5:57:04 PM	**Injection:**	1 of 1
**Acq. method:**	PHYSCHEM5_CPL_0-100_A2C_C8_05_30_5.amx	**Injection volume:**	2.000
**Analysis method:**	CPL_SKRIN_Zaktualizowany.pmx	**Acq. operator:**	Monika Delis

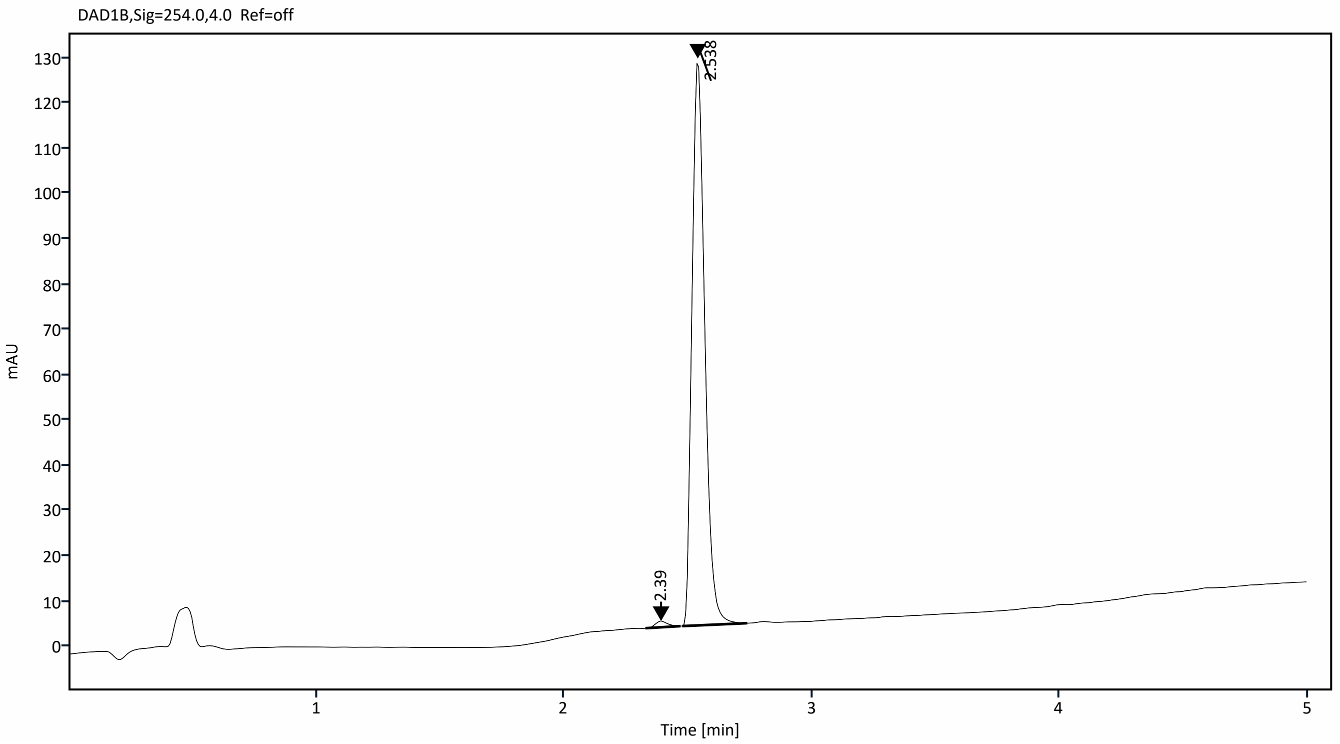

Signal: DAD1B,Sig=254.0,4.0 Ref=off

Name	RT [min]	RRT	Area	Area%	Height	Peak Tail Factor	Peak Asymmetry 10%
	2.390		4.6935	1.0464	1.3791	1.54834	1.61099
	2.538		443.8392	98.9536	125.2085	1.17372	1.10123
		Sum	448.5328				

Screen

Compound 16

Data file: /LAF non-GMP/CPL LIBRARY/PURITY SCREENING/Results/2023/2023 04/2023 04 18/purity_screen_A2C_Monika Delis_20230418 144135_PHYSCHEM_03.rslt\2023-04-18 18-02-16+02-00-37.dx

Method:

Sample name: KSK21,FR18565891

Sample type: Sample

Instrument: PHYSCHEM_03

Location: D1F-C5

Injection date: 4/18/2023 6:02:58 PM

Injection: 1 of 1

Acq. method: PHYSCHEM5_CPL_0-100_A2C_C8_05_30_5.amx

Injection volume: 2.000

Analysis method: CPL_SKRIN_Zaktualizowany.pmx

Acq. operator: Monika Delis

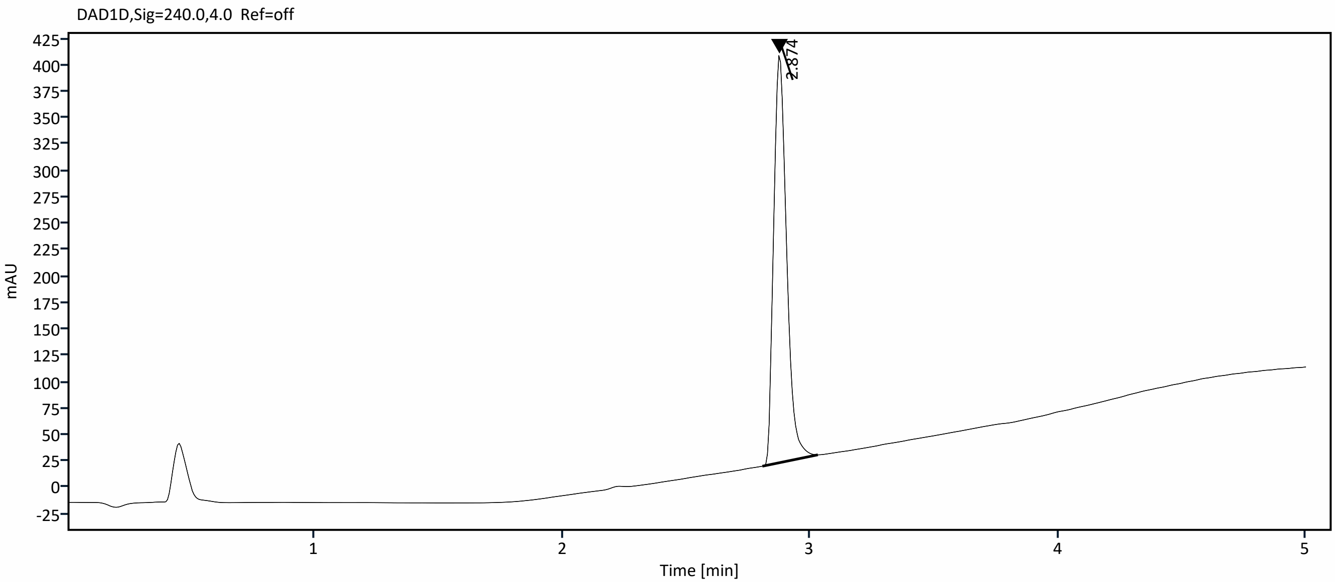

Signal: DAD1D,Sig=240.0,4.0 Ref=off

Name	RT [min]	RRT	Area	Area%	Height	Peak Tail Factor	Peak Asymmetry 10%
	2.874		1392.24 45	100.00 00	387.42 40	1.43738	1.28700
		Sum	1392.24 45				
